# Heteroaryl-Directed Iridium-Catalyzed Enantioselective C–H Alkenylations of Secondary Alcohols

**DOI:** 10.1021/jacs.4c16414

**Published:** 2024-12-23

**Authors:** Wenbin Mao, Craig M. Robertson, John F. Bower

**Affiliations:** Department of Chemistry, University of Liverpool, Crown Street, Liverpool L69 7ZD, United Kingdom

## Abstract

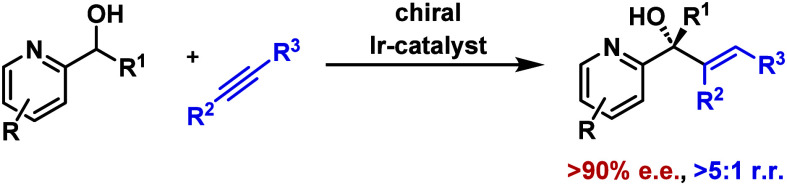

Under iridium-catalyzed conditions, 2-aza-aryl-substituted secondary alcohols undergo C(sp^3^)–H addition reactions to alkynes to provide alkenylated tertiary alcohols. The processes occur with very high regio- and enantioselectivity. An analogous addition to styrene is shown to provide a prototype C(sp^3^)–H alkylation process. A mechanism based on directed aza-enolization of the reactant alcohol is proposed.

We have recently outlined enantioselective alkylation processes that are predicated on the addition of catalytically generated Ir-enolates or aza-enolates to minimally activated alkenes (Scheme 1A).^1^ From the viewpoint of alkene functionalization, these methods are significant because (a) they are highly atom and step economical^2^ and (b) they offer complete branched selectivity for challenging C(sp^2^/sp^3^)–H additions.^3^ Indeed, there are only a handful of other methods available that can effect branched selective and enantioselective C–H additions to minimally polarized alkenes.^4^ Accordingly, further development of the C(sp^2^/sp^3^)–C(sp^3^) cross-coupling framework in Scheme 1A is warranted,^5^ and the unusual reactivity features that have been uncovered so far provide additional impetus. For example, the use of catalytically generated Ir-enolates and related species in reaction design is unusual, and the potential for leveraging their intermediacy in regiocontrolled enantioselective C–H addition processes is essentially unexplored.^6,7^

**Scheme 1:**
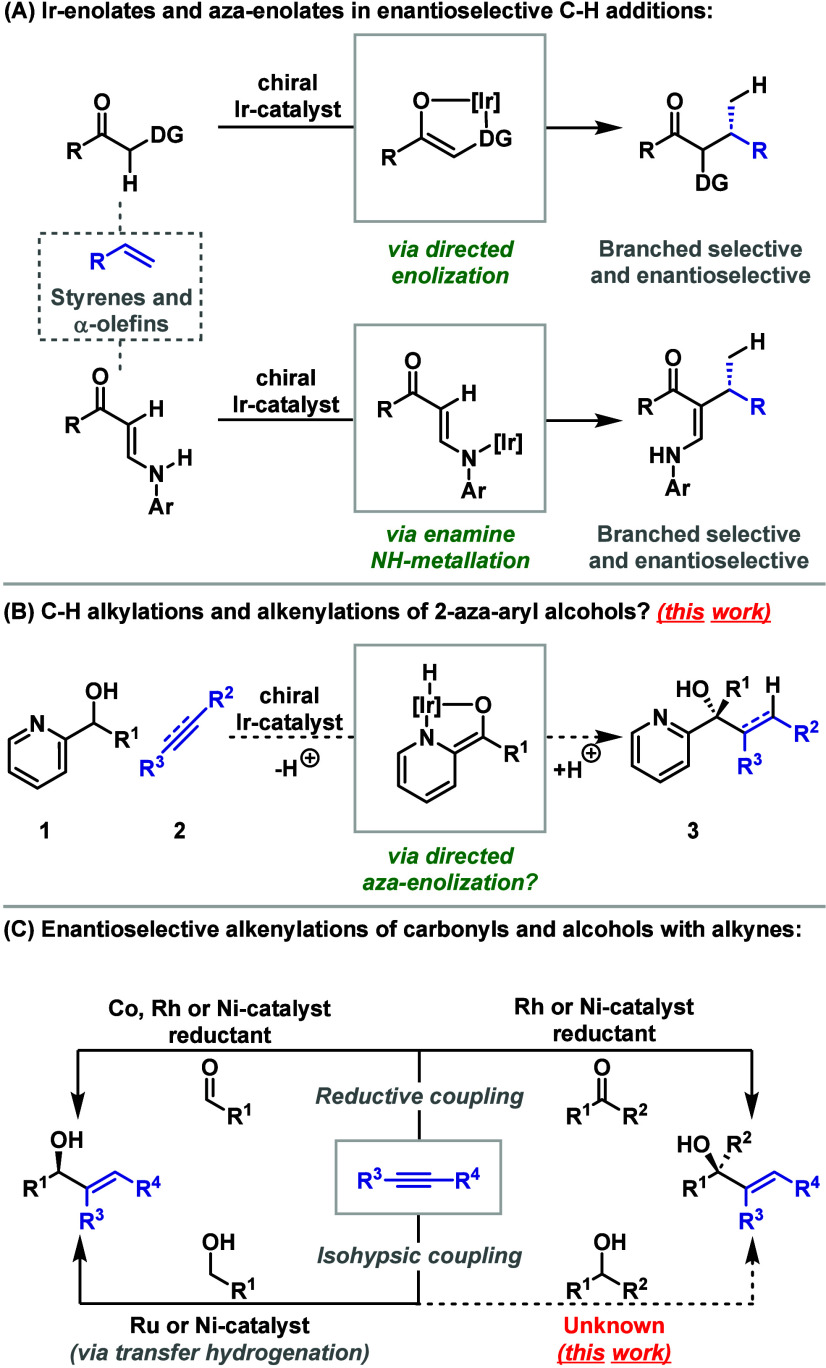
Introduction

The methods we have reported so far are based on either directing-group (DG) mediated “soft” enolization to give an Ir-enolate (Scheme 1A, top equation) or NH-metalation of a persistent enamine to give an Ir-aza-enolate (Scheme 1A, bottom equation).^1^ To extend the range of available initiating modes, we considered whether a DG might be able to facilitate “soft” *aza*-enolization. This led us to evaluate 2-aza-aryl alcohols of type **1**, where we envisaged that metalation of the OH unit and coordination of nitrogen would lead to the formation of an Ir-aza-enolate (Scheme 1B).^8^ Addition of this to an alkene or other π-unsaturated species would then lead to tertiary alcohols **3**, which possess a new tetrasubstituted stereocenter. Outlined below are our studies in this area, which have resulted in (1) a prototype alkylation process using styrene and (2) a highly efficient protocol for C–H alkenylation using alkynes. The latter complements existing enantioselective intermolecular reductive^9,10^ and isohypsic (transfer hydrogenation-based) carbonyl alkenylation methods^11,12^ by enabling the direct and enantioselective up conversion of secondary alcohols to tertiary derivatives (Scheme 1C). The method also offers distinct regioselectivities compared to nonenantioselective alkyne-based C–H alkenylations of secondary alcohols, which require Ru-catalyzed conditions.^12b,12c^

In early efforts toward the envisaged process, we exposed alcohol **1a** to styrene **4** in the presence of [Ir(cod)_2_]BARF/(*R*)-BINAP (**L1**) (5 mol %) and this led to the formation of **5** in 78% yield and a 1:1 mixture of diastereomers (Scheme 2A and vide infra). The process was completely selective for the branched product, with inefficiencies attributed to Ir-catalyzed transfer hydrogenative oxidation of the alcohol to the corresponding ketone (20% yield) with concomitant reduction of **4** to ethylbenzene. To recycle the ketone side product, the conditions were modified to include *i*-PrOH (250 mol %) as a sacrificial reductant, and this increased the yield of **5** to 93%. The hydroalkylation of **4** to **5** is unusual,^13^ but because we were unable to improve the diastereoselectivity, we instead evaluated the use of an alkyne as the coupling partner on the basis that this would generate a C–H alkenylation product possessing only one stereocenter. Indeed, Takeuchi has previously demonstrated that Ir-enolates derived from 1,3-dicarbonyls can add to alkynes to give achiral or racemic alkenylation products.^7a^ Under conditions analogous to those shown in Scheme 2A, reaction of **1a** with **2a** provided product **3aa** in 72% yield, 99% e.e. and 19:1 r.r., favoring functionalization at the β-position of the alkyne (Scheme 2B). A variety of other homochiral diphosphine ligands (**L2**–**L6**) were evaluated, but these offered no appreciable benefits in this case (vide infra). Accordingly, further optimization was undertaken with **(±)-L1**, as detailed in Scheme 2C. Ultimately, by using 3,3-dimethyl-2-butanol as the sacrificial reductant (Entry 11), conditions were identified that deliver **3aa** in 83% yield and with high regioselectivity at 110 °C.

**Scheme 2:**
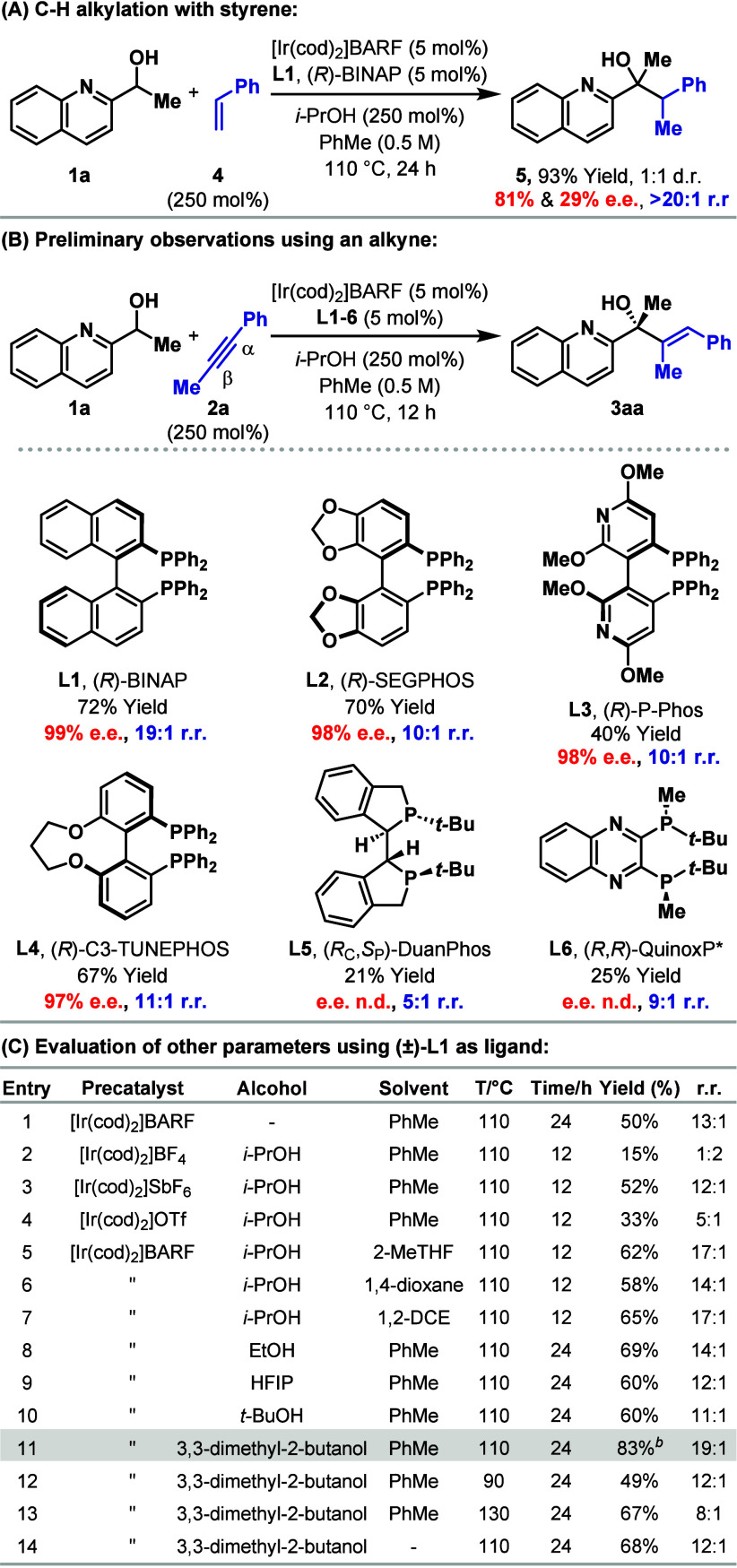
Reaction Development All reactions were conducted on a 0.10 mmol scale. Yields were determined by the ^1^H NMR analysis of the crude mixture using 1,3,5-trimethoxybenzene as an internal standard. r.r. is the ratio of β:α selectivity and was determined by ^1^H NMR analysis of the crude mixture. 1-(Quinolin-2-yl)ethan-1-one (i.e., the ketone of **1a**) was observed as a side product in all reactions. Further optimization results are given in the Supporting Information. Isolated yield.

The optimized conditions using homochiral **L1** were next used to explore the scope of the process with respect to the alkyne component (Tabel 1). Initially, this involved the exploration of the effects of substitution on the aromatic units of various aryl propynes (**2a**–**m**). In all cases, products **3aa**–**3am** were generated with very high levels of enantioselectivity and generally excellent levels of β:α regioselectivity. Notably, *ortho*-substitution is tolerated (**3ab**), halides remain intact (**3ag**, **3ah**), and strongly electron withdrawing substituents (e.g., **3al**) can be accommodated. The absolute stereochemistry of **3ag** was determined by single crystal X-ray diffraction, and this provided the basis for the other stereochemical assignments. Beyond simple aryl groups, it was also found that thienyl (**3an**) and alkyl propynes (**3ao**) could cross-couple with **1a**. Further scope exploration of alkylated propynes (750 mol %) was conducted using pyridyl alcohol **1b** with *i*-PrOH as the sacrificial reductant. Systems with primary (**3bo**, **3bp**), secondary (**3bq**, **3br**) and tertiary (**3bs**) alkyl groups all reacted efficiently and with high selectivity. The efficient formation of **3bt** demonstrates that methyl-substitution on the alkyne is not a requirement, whereas **3bu** and **3bv** demonstrate that useful functionality can be carried on the alkyne. Certain limitations have been identified, including terminal alkynes and the systems summarized at the bottom of Table 1. The reactions in Table 1 were run on 0.10 mmol scale, but we verified that similar efficiencies can be achieved on gram scale, as exemplified by the conversion of **1a** (5.8 mmol) to **3aa** in 76% yield and 99% e.e. (see the Supporting Information).

**Table 1:**
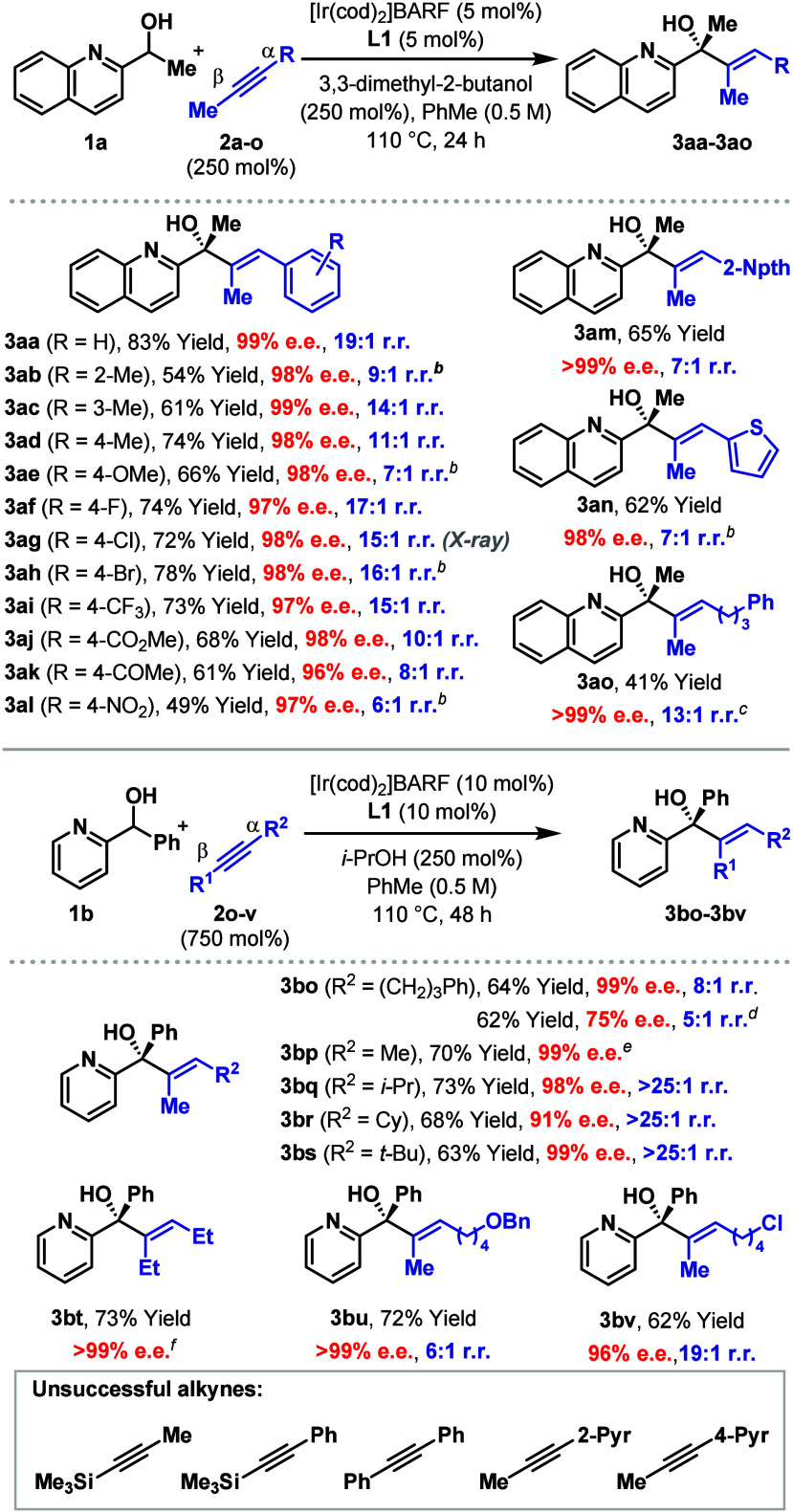
Scope of the Alkyne^a^

a See Table 1 footnotes. Isolated yields are given.

b [Ir(cod)_2_]BARF/**L1** (10 mol %), and alkyne (500 mol %) were used for 48 h, except for **3ah** where the reaction time was 24 h.

c [Ir(cod)_2_]BARF/**L1** (10 mol %) and *i-*PrOH (250 mol %) were used.

d [Ir(cod)_2_]BARF/**L6** (10 mol %), *i-*PrOH (250 mol %) and **2p** (250 mol %) were used.

e [Ir(cod)_2_]BARF/**L1** (5 mol %) were used.

f Alkyne (250 mol %) was used.

Table 2 outlines the scope of the process with respect to the heteroaryl alcohol component using alkynes **2p** or **2a** (750 mol %). Substituted pyridines are, in general, well tolerated, with electronically distinct systems **3gp**–**3kp** all offering good levels of efficiency. The most significant observation is that substitution at either C3 or C6 is not tolerated, such that very low yields were obtained for **3cp** and **3fp**. The failure of the latter is consistent with the notion that coordination of the N-center to the Ir-catalyst is an important factor. Variation of the R^1^ group of the alcohol was also explored and this revealed very good scope for primary (**3la**) and secondary substituents (**3mp**–**3pp**). **3qp**, where R^1^ = *t*-butyl, was formed in only low yield, but high enantioselectivity was still maintained. It was found that **L6** offered higher efficiencies than **L1** for the formation of **3ra**; this was not the case elsewhere, as highlighted by comparative results for **3bo** (see Table 1) and **3aa** (see Scheme 2B). Interestingly, bicyclic system **1s** cross-coupled smoothly with **2p** to provide **3sp** in 60% yield with 97% e.e. This result is notable because it contrasts with **3cp**, where a substituent at C3 of the pyridyl ring was not tolerated. An isoquinoline unit was also able to direct the process to form **3tp** in a satisfactory manner. So far, we have found that the heteroaryl unit is limited to pyridine-like systems: benzothiazole (**1u**) and pyrazinyl (**1v**) units were not able to promote cross-coupling (Table 2, box). Equally, 3- and 4-pyridyl substituents (**1w** and **1x**) and simple phenyl groups (**1y**) were ineffective. The lack of reactivity observed with *O***-Me-1r** highlights the importance of a free hydroxyl unit.

**Table 2:**
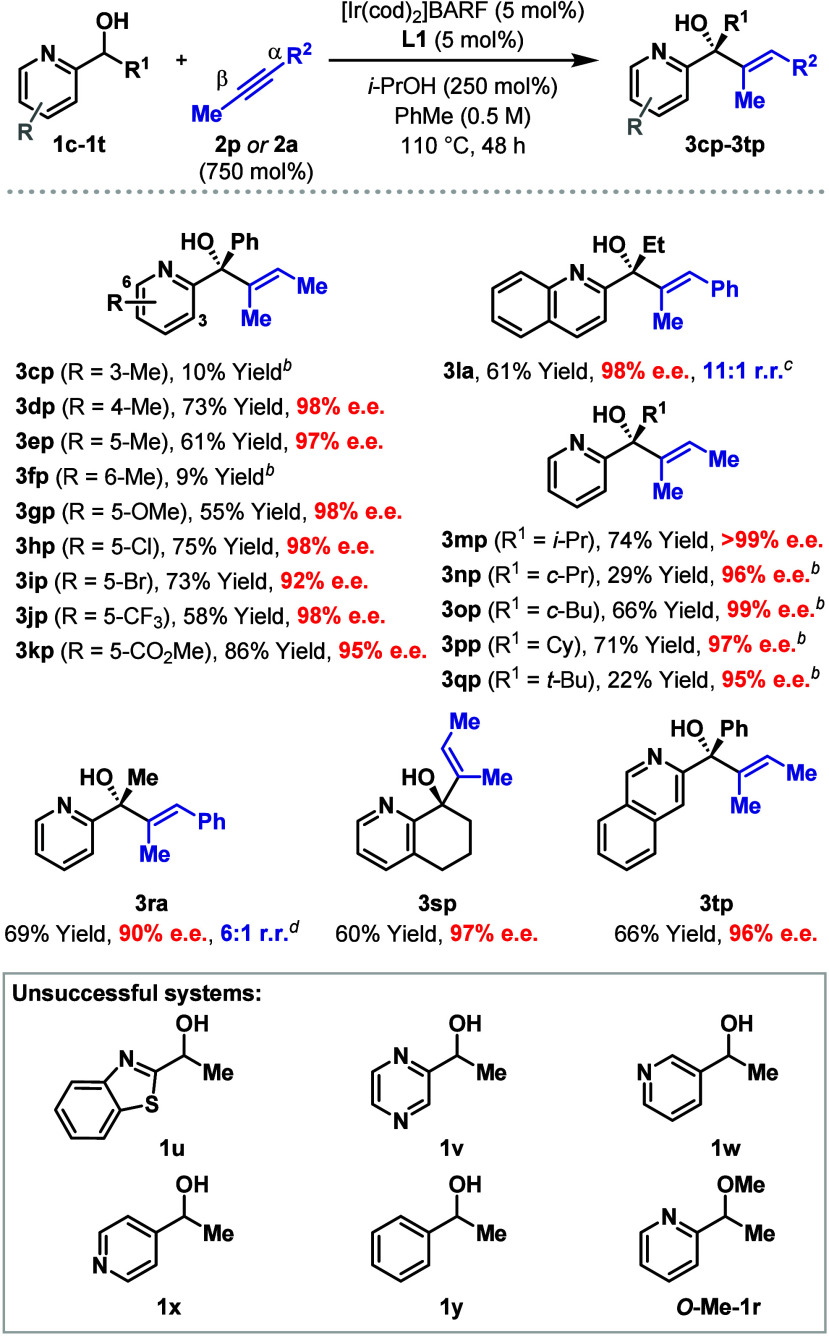
Scope of the Heteroaryl Alcohol^a^

a See Table 1 footnotes. Isolated yields are given.

b [Ir(cod)_2_]BARF/**L1** (10 mol %) were used for 48 h.

c [Ir(cod)_2_]BARF/**L1** (10 mol %), 3,3-dimethyl-2-butanol (250 mol %) and **2a** (250 mol %) were used.

d [Ir(cod)_2_]BARF/**L6** (10 mol %) and **2a** (250 mol %) were used.

We have explored derivatizations of the products, using **3aa** as a testbed, and found that diastereoselective processes are feasible (Scheme 3). For example, treatment of **3aa** with *m*-CPBA provided epoxide **6** in 67% yield and 7:1 d.r. The relative stereochemistry of the major diastereomer was assigned by single crystal X-ray diffraction analysis.^14^ We were especially interested in exploring manipulations of the heteroaryl unit, because this is a requirement for the alkenylation process. Pleasingly, we found that catalyst-controlled hydrogenation of the heterocyclic ring of **3aa** was efficient using an Ir-system modified with **L2**.^15^ This allowed the highly diastereoselective formation of tetrahydroquinoline **7a**.^16^ Diastereomeric system **7b** could be accessed from *ent***-3aa** under the same conditions, thereby demonstrating the ability to control the relative and absolute configurations of the vicinal stereocenters of these targets.

**Scheme 3:**
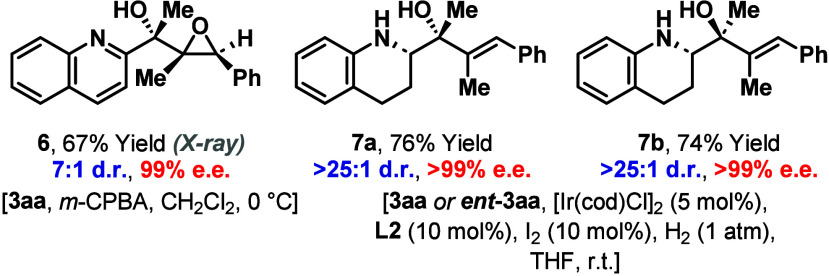
Derivatizations of 3aa

Based on the optimization studies and substrate scope, mechanisms proceeding via either the aza-enolate-based proposition from Scheme 1B, or an oxidative-cyclocoupling-based transfer hydrogenative pathway should be considered (Scheme 4A, *path a* vs *path b*). For *path a*, metalation of the hydroxyl group provides **Int-I**, where the Ir-center may or may not be protonated (the former option is depicted).^17^ Aza-enolization then provides **Int-II**, which engages the alkene/alkyne via an inner sphere *syn*-carbometalation process to provide **Int-III**.^18^ Alternate mechanisms for the conversion of **1** to **Int-II** can also be envisaged.^19^ The viability of the addition of an Ir-aza-enolate across an alkyne finds support from stoichiometric studies involving Ir-acac complexes.^20^ Nevertheless, alternate C–C bond forming options should also be considered;^21^ for example, NH reductive elimination to **Int-II′** may occur in advance of addition of the enamine and the Ir-center across the alkene/alkyne en route to **Int-III**. From here, C–H reductive elimination and protodemetalation provides **3**. The feasibility of (Lewis) acid-mediated aza-enolization from **Int-I** to provide **Int-II** is evidenced by the deuterium exchange experiment detailed in Scheme 4B, and related aza-enolization processes have been exploited in other contexts.^8^ For *path b*, Ir-catalyzed oxidation of **1** provides ketone **Int-IV** with concomitant reduction of the alkyne/alkene (or cod). Coordination of the Ir-catalyst to **Int-IV** then provides **Int-V**, which engages the alkene/alkyne in a chelation-assisted oxidative-cyclocoupling process to provide **Int-VI**.^12a^ Transfer hydrogenolysis of this by another equivalent of **1** leads to **3** and a further equivalent of **Int-IV**. Secondary coordination has been shown to facilitate oxidative-cyclocoupling processes in other contexts, most notably in Murai’s Ru-catalyzed (2 + 2 + 1) carbonylative cycloadditions, where carbonyl or heteroaryl directing groups were employed.^22^ This effect has also been exploited by Krische and co-workers in transfer hydrogenative C–C couplings.^23^ Nevertheless, to the best of our knowledge, transfer hydrogenative carbonyl additions using phosphine ligated cationic Ir(I)-systems require activated π-systems and do not tolerate styrenes, which contrasts with the result in Scheme 2A. Indeed, styrenes are recognized as a challenging substrate class, requiring catalysts based on other transition metals.^13,24,25^ Consequently, based on the substrate scope of the process described here, we currently favor *path a*. Efforts to unambiguously assign mechanism are complicated by the ability of cationic Ir-systems to rapidly and reversibly dehydrogenate alcohols.^26^ Indeed, ketones are also viable substrates under optimized conditions (Scheme 4C), but this process could be rationalized either via *path a* or *path b*, with the key issue being whether the alcohol additive (3,3-dimethyl-2-butanol or *i*-PrOH) effects reduction of **Int-IV** to **1**(27) or **Int-VI** to **3**. To distinguish these options, the coupling of **8** with **2a** was explored in the presence of stoichiometric [Ir], but in the absence of 3,3-dimethyl-2-butanol (Scheme 4D). This led to 78% recovery of **8**, with no evidence for C–C bond formation, which would appear to rule out *path b*.^28^

**Scheme 4:**
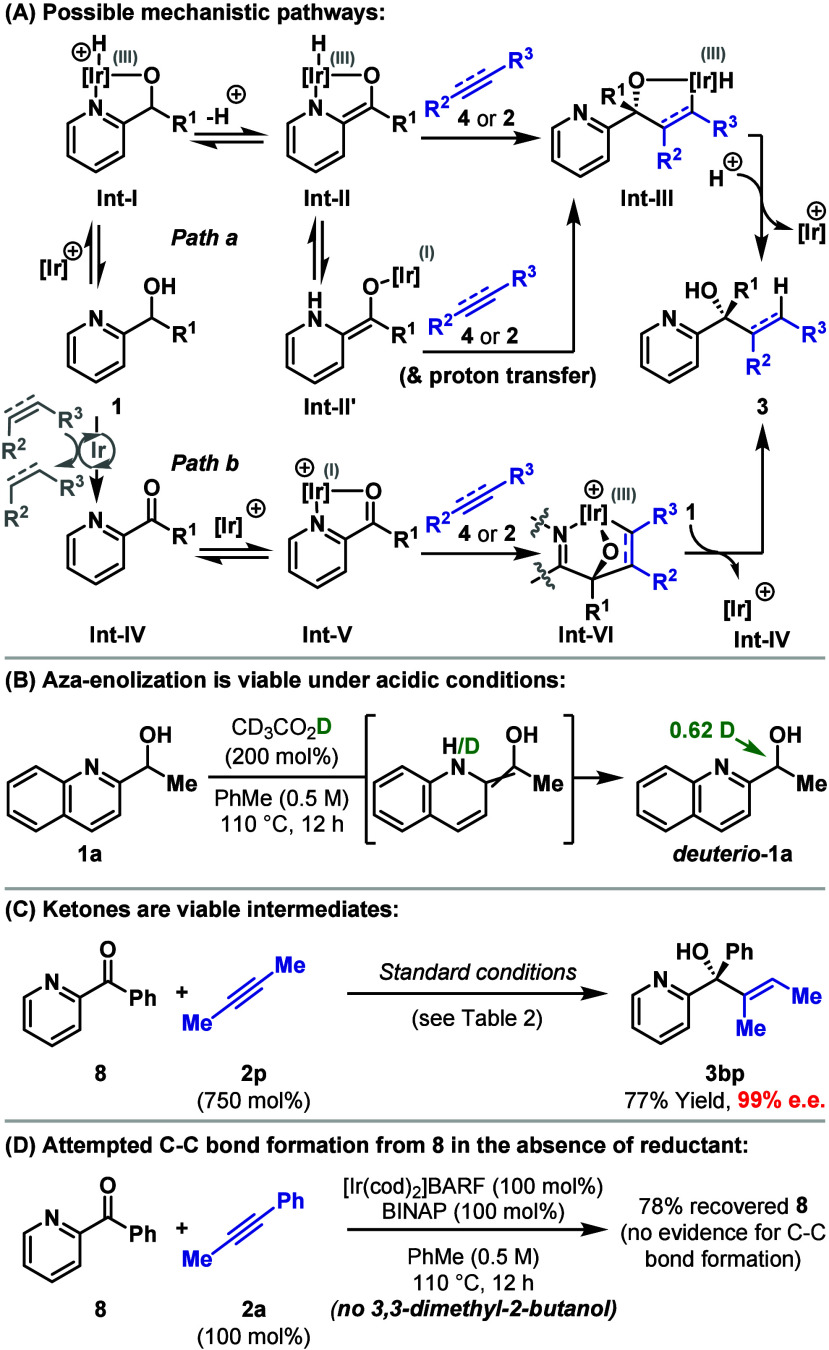
Mechanistic Considerations

In summary, we demonstrate that 2-aza-aryl-substituted secondary alcohols readily undergo C(sp^3^)–H addition reactions to styrene and alkynes under Ir-catalyzed conditions. These operationally simple C–C cross-coupling processes conform to green chemistry ideals because they are highly regioselective, highly enantioselective, and highly step/atom economical. The alkyne-based processes offer a unique C–H alkenylation method for upgrading secondary alcohols to tertiary derivatives. Our studies have highlighted the importance of both the 2-aza-aryl unit and the alcohol for inducing reactivity. Although alternate mechanistic pathways cannot be discounted, the collective evidence at this stage is suggestive of a pathway that proceeds via the alcohol-directed formation of an Ir-aza-enolate. Further efforts to expand this unusual mode of reactivity are ongoing and will be reported in due course.

## Supporting Information Available

Experimental details, characterization data, crystallographic data, and NMR spectra (PDF)

## References

[ref1] aHong F.; Aldhous T. P.; Kemmitt P. D.; Bower J. F. A Directed Enolization Strategy Enables Byproduct Free Construction of Contiguous Stereocentres en Route to Complex Amino Acids. Nat. Chem. 2024, 16, 1125–1132. 10.1038/s41557-024-01473-5. 38565976 PMC11230901

[ref2] aWender P. A.; Miller B. L. Synthesis at the Molecular Frontier. Nature 2009, 460, 197–201. 10.1038/460197a. 19587760 PMC2857510

[ref3] aDong Z.; Ren Z.; Thompson S. J.; Xu Y.; Dong G. Transition-Metal-Catalyzed C–H Alkylation Using Alkenes. Chem. Rev. 2017, 117, 9333–9403. 10.1021/acs.chemrev.6b00574. 28125210

[ref4] aRajanBabu T. V. Asymmetric Hydrovinylation Reaction. Chem. Rev. 2003, 103, 2845–2860. 10.1021/cr020040g. 12914483

[ref5] aMa X.; Murray B.; Biscoe M. R. Stereoselectivity in Pd-Catalysed Cross-Coupling Reactions of Enantioenriched Nucleophiles. Nat. Rev. Chem. 2020, 4, 584–599. 10.1038/s41570-020-00222-9. 33869786 PMC8049355

[ref6] aHuo H.; Wang C.; Harms K.; Meggers E. Enantioselective, Catalytic Trichloromethylation through Visible-Light-Activated Photoredox Catalysis with a Chiral Iridium Complex. J. Am. Chem. Soc. 2015, 137, 9551–9554. 10.1021/jacs.5b06010. 26193928

[ref7] aOnodera G.; Kato M.; Kawano R.; Kometani Y.; Takeuchi R. Highly Regio- and Stereoselective Addition of 1,3-Diketones to Internal Alkynes Catalyzed by Cationic Iridium Complex. Org. Lett. 2009, 11, 5038–5041. 10.1021/ol9020095. 19795904

[ref8] aJiang K.; Pi D.; Zhou H.; Liu S.; Zou K. Iron-Catalyzed C(sp^3^)-H Functionalization of Methyl Azaarenes with α-Oxoesters: a Facile Approach to Lactic Acid Derivatives. Tetrahedron 2014, 70, 3056–3060. 10.1016/j.tet.2014.02.069.

[ref9] For a review, see: Ortiz E.; Shezaf Z.; Chang Y.-H.; Krische M. J. Enantioselective Metal-Catalyzed Reductive Coupling of Alkynes with Carbonyl Compounds and Imines: Convergent Construction of Allylic Alcohols and Amines. ACS Catal. 2022, 12, 8164–8174. 10.1021/acscatal.2c02444. 37082110 PMC10112658

[ref10] aMiller K. M.; Huang W.-S.; Jamison T. F. Catalytic Asymmetric Reductive Coupling of Alkynes and Aldehydes: Enantioselective Synthesis of Allylic Alcohols and α-Hydroxy Ketones. J. Am. Chem. Soc. 2003, 125, 3442–3443. 10.1021/ja034366y. 12643701

[ref11] aCai Y.; Zhang J.-W.; Li F.; Liu J.-M.; Shi S.-L. Nickel/N-Heterocyclic Carbene Complex-Catalyzed Enantioselective Redox-Neutral Coupling of Benzyl Alcohols and Alkynes to Allylic Alcohols. ACS Catal. 2019, 9, 1–6. 10.1021/acscatal.8b04198.

[ref12] aNgai M.-Y.; Barchuk A.; Krische M. J. Iridium-Catalyzed C–C Bond Forming Hydrogenation: Direct Regioselective Reductive Coupling of Alkyl-Substituted Alkynes to Activated Ketones. J. Am. Chem. Soc. 2007, 129, 280–281. 10.1021/ja0670815. 17212400

[ref13] Control experiments have shown that the conditions in Scheme 2A can effect reduction of styrene to ethyl benzene but are less effective for the reduction of alkynes (see the Supporting Information). The only comparable hydrocarbonations of styrene we are aware of require 3-hydroxy-2-oxindoles: Yamaguchi E.; Mowat J.; Luong T.; Krische M. J. Regio- and Diastereoselective C–C Coupling of α-Olefins and Styrenes to 3-Hydroxy-2-oxindoles by Ru-Catalyzed Hydrohydroxyalkylation. Angew. Chem., Int. Ed. 2013, 52, 8428–8431. 10.1002/anie.201303552. PMC3816539 23832830

[ref14] A rationale for the diastereoselectivity is unclear at this stage.

[ref15] Wang W.-B.; Lu S.-M.; Yang P.-Y.; Han X.-W.; Zhou Y.-G. Highly Enantioselective Iridium-Catalyzed Hydrogenation of Heteroaromatic Compounds, Quinolines. J. Am. Chem. Soc. 2003, 125, 10536–10537. 10.1021/ja0353762. 12940733

[ref16] The Supporting Information details the relative stereochemical assignment of **7a** and **7b**.

[ref17] aGreen M.; Kuc T. A.; Taylor S. H. Cationic Transition-Metal Complexes. Part 1. Synthesis and Reactions of Bis(diene)-Rhodium and -Iridium Tetrafluoroborates. J. Chem. Soc. (A) 1971, 2334–2337. 10.1039/j19710002334.

[ref18] C–H acidification of an amido-DG stabilized analogue of **Int-I** en route to a chelate related to **Int-II** has been noted: Tejel C.; Ciriano M. A.; del Río M. P.; Hetterscheid D. G. H.; Tsichlis i Spithas N.; Smits J. M. M.; de Bruin B. Ligand Oxidation of a Deprotonated Bis(picolyl)amine Ir^I^(cod) Complex. Chem.—Eur. J. 2008, 14, 10932–10936. 10.1002/chem.200801162. 19016555

[ref19] **Int-II** could also form via β-hydride elimination from the Ir-alkoxide of **1**.

[ref20] aRussell D. R.; Tucker P. A. Crystal and Molecular Structure of *acb*[l,2-Bis(trifluoromethyl)-3-acetyl-4-oxopent-I-enyl-*O’*,*O*,*C*^*1*^]-*fde*{l,4–5,β-η-[bis(trifluoromethyl)-ethylene]oct-4-enyl)iridium(111): an Addition Product of Hexafluorobut-2-yne with Co-ordinated Cyclo-octa-I,5-diene. J. Chem. Soc., Dalton Trans. 1975, 1749–1752. 10.1039/dt9750001749.

[ref21] Another option is that O–H reductive elimination from **Int-II** generates a nonchelated Ir-aza-enolate that adds across the alkyne, via a mechanism akin to that proposed in ref (1c).

[ref22] Chatani N.; Tobisu M.; Asaumi T.; Fukumoto Y.; Murai S. Ruthenium Carbonyl-Catalyzed [2 + 2 + 1]-Cycloaddition of Ketones, Olefins, and Carbon Monoxide, Leading to Functionalized γ-Butyrolactones. J. Am. Chem. Soc. 1999, 121, 7160–7161. 10.1021/ja991223w.

[ref23] Park B. Y.; Montgomery T. P.; Garza V.; Krische M. J. Ruthenium Catalyzed Hydrohydroxyalkylation of Isoprene Employing Heteroaromatic Secondary Alcohols: Isolation and Reversible Formation of the Putative Metallacycle Intermediate. J. Am. Chem. Soc. 2013, 135, 16320–16323. 10.1021/ja4087193. 24156560 PMC3855325

[ref24] Nguyen K. D.; Park B. Y.; Luong T.; Sato H.; Garza V. J.; Krische M. J. Metal-Catalyzed Reductive Coupling of Olefin-Derived Nucleophiles: Reinventing Carbonyl Addition. Science 2016, 354, aah5133 10.1126/science.aah5133. 27846504 PMC5130112

[ref25] aXiao H.; Wang G.; Krische M. J. Regioselective Hydrohydroxyalkylation of Styrene with Primary Alcohols or Aldehydes via Ruthenium Catalyzed C-C Bond Forming Transfer Hydrogenation. Angew. Chem., Int. Ed. 2016, 55, 16119–16122. 10.1002/anie.201609056. PMC5189692 27910228

[ref26] Bower J. F.; Skucas E.; Patman R. L.; Krische M. J. Catalytic C-C Coupling via Transfer Hydrogenation: Reverse Prenylation, Crotylation and Allylation from the Alcohol or Aldehyde Oxidation Level. J. Am. Chem. Soc. 2007, 129, 15134–15135. 10.1021/ja077389b. 18020342

[ref27] Another entry point into *Path a* is via hydride transfer onto the Ir-center of **Int-V** to give **Int-II**.

[ref28] Additional mechanistic experiments, including kinetic experiments, and deuterium labelling and exchange studies, are detailed in the Supporting Information. These led us to investigate the use of cyclohexylallene as a coupling partner, but this participated in low yield. In addition to the mechanisms proposed in Scheme 4A, options based upon hydrometalation of the alkyne to generate an alkenyl-Ir species cannot be ruled out at this stage.

