# Supplementary material for: Heteroaryl-Directed Iridium-Catalyzed Enantioselective C–H Alkenylations of Secondary Alcohols
Source: J Am Chem Soc. 2024 Dec 23;147(1):118–24. doi: 10.1021/jacs.4c16414 (PMC11726574; doi:10.1021/jacs.4c16414)
Supplement: Supplementary file 1 — ja4c16414_si_001.pdf [file ja4c16414_si_001.pdf]

Heteroaryl-Directed Iridium-Catalyzed Enantioselective C-H Alkenylations of Secondary Alcohols

Wenbin Mao, Craig M. Robertson, and John F Bower*

Department of Chemistry, University of Liverpool, Crown Street, Liverpool, L69 7ZD, United Kingdom

John.Bower@liverpool.ac.uk

Supporting Information

Table of Contents

1 General Information S3

2 Optimization of Reaction Conditions S5

3 Experimental Details and Characterization Data S6

3.1 Starting Materials S6

3.2 Products S19

4 Iridium-Catalyzed Cross Coupling of Primary Alcohol with Styrene S72

5 Gram Scale Reaction S73

6 Derivatizations of **3aa** S74

6.1 Epoxidation S74

6.2 Stereodivergent Synthesis by Stereoselective Quinoline Reduction S77

7 Mechanistic Studies S84

7.1 Reaction of Alkyne/Alkene under Standard Conditions S84

7.2 Reaction of Ketone **8** under Standard Conditions S86

7.3 Deuterium Labelling and Exchange Studies. S87

7.4 Visual Kinetic Analysis S95

7.5 Attempted C-C Bond Formation from **8** in the Absence of Reductant S101

8 NMR Spectra S104

9 References S168

1 General Information

All chemicals were purchased from commercial suppliers and used as received unless otherwise stated. All solvents (CH_2Cl_2, *n*-hexane, toluene, Et_2O and THF) were dried and purified following standard procedures.

All reactions were performed in a flame-dried glassware using conventional Schlenk techniques under a static pressure of nitrogen unless otherwise stated. Liquids and solutions were transferred with syringes.

Analytical thin layer chromatography (TLC) was performed on aluminium backed 60 F_{254} silica plates.

Flash column chromatography (FCC) was performed on silica gel (*Aldrich* 40-63 µm, 230-400 mesh) using the indicated solvents.

^{1}H, ^{13}C and ^{19}F NMR spectra were recorded in $CDCl_3$ on *Bruker* AV400 and AV500 instruments. Chemical shifts are reported in parts per million (ppm) and are referenced to the residual solvent resonance as the internal standard ($CHCl_3$: δ = 7.26 ppm for ^{1}H NMR and $CDCl_3$: δ = 77.16 ppm for ^{13}C NMR). Data are reported as following: chemical shift, multiplicity (s = singlet, d = doublet, t = triplet, q = quartet, m = multiplet, m_c = centrosymmetric multiplet, br = broad signal), coupling constants (Hz), and integration. Assignments of ^{1}H NMR and ^{13}C NMR signals were made, where possible, using COSY, HSQC, HMBC, and NOE experiments. Numbering systems for NMR signal assignments are specified on the structure and are not related to those used for the compound names.

Enantiomeric excesses were determined using an *Agilent* 1290 Infinity chiral SFC, equipped with a diode array detector, under conditions stated for each compound.

Infrared (IR) spectra were recorded on on a *Perkin Elmer* Spectrum Two FTIR spectrometer as thin films or solids compressed on a diamond plate. Only selected absorption maxima ($\tilde{\nu}$) are reported in wavenumbers (cm^{-1})

Melting points were determined using *Reichert* melting point apparatus and are uncorrected.

High resolution mass spectra (HRMS) were obtained from the *University of Liverpool* mass spectrometry service, given to four decimal places. Mass spectra were recorded on *Agilent* 7200 Accurate Mass QTOF GC/MS (under condition of chemical ionization-CI) and *Agilent* 6540 UHD Accurate Mass Q-TOF LC/MS (under condition of electrospray ionization-ESI).

Optical rotations were measured on ADP440+ polarimeter at the concentration and temperature stated.

Compound names are generated by *ChemDraw* 16.0 software (*PerkinElmer*), following IUPAC nomenclature.

2 Optimization of Reaction Conditions[a]

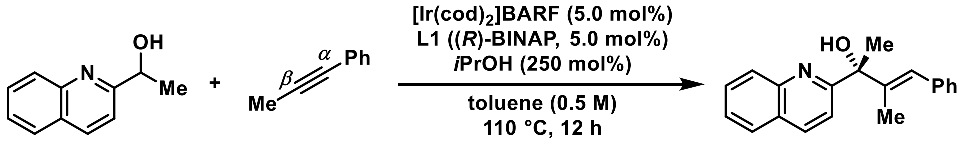

1a (0.10 mmol) **2a (250 mol%)** **3aa**

Evaluation of chiral ligands instead of L1

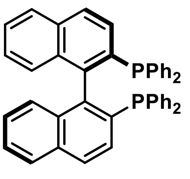

L1 ((*R*)-BINAP)
72% Yield
99% ee, 19:1 r.r.

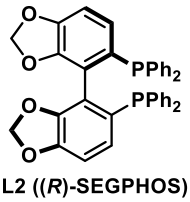

L2 ((*R*)-SEGPHOS)
70% Yield
98% ee, 10:1 r.r.

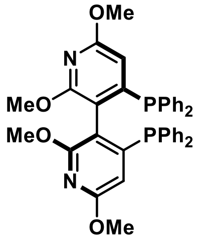

L3 ((*R*)-P-Phos)
40% Yield
98% ee, 10:1 r.r.

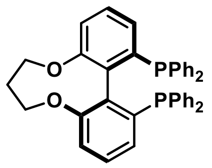

L4 ((*R*)-C3-TUNEPHOS)
67% Yield
97% ee, 11:1 r.r.

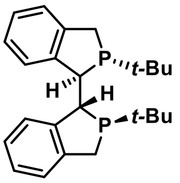

L5 ((R_C,S_P)-DuanPhos)
21% Yield
ee n.d., 5:1 r.r.

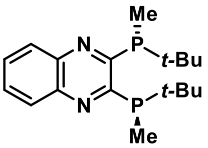

L6 ((*R*,*R*)-QuinoxP*)
25% Yield
ee n.d., 9:1 r.r.

Evaluation of other parameters

entry	cat.	L	alcohol	solvent	T/°C	t/h	yield	*r.r.*
1	$[Ir(cod)_2]BARF$	(±)-L1	*i*PrOH	toluene	110	12	72%	19:1
2	$[Ir(cod)_2]BF_4$	(±)-L1	*i*PrOH	toluene	110	12	15%	1:2
3	$[Ir(cod)_2]SbF_6$	(±)-L1	*i*PrOH	toluene	110	12	52%	12:1
4	$[Ir(cod)_2]OTf$	(±)-L1	*i*PrOH	toluene	110	12	33%	5:1
5	$[Ir(cod)_2]BARF$	(±)-L1	*i*PrOH	mesitylene	110	12	61%	16:1
6	$[Ir(cod)_2]BARF$	(±)-L1	*i*PrOH	2-MeTHF	110	12	62%	17:1
7	$[Ir(cod)_2]BARF$	(±)-L1	*i*PrOH	1,4-dioxane	110	12	58%	14:1
8	$[Ir(cod)_2]BARF$	(±)-L1	*i*PrOH	DCE	110	12	65%	17:1
9	$[Ir(cod)_2]BARF$	(±)-L1	MeOH	toluene	110	12	65%	15:1
10	$[Ir(cod)_2]BARF$	(±)-L1	EtOH	toluene	110	24	69%	14:1
11	$[Ir(cod)_2]BARF$	(±)-L1	HFIP	toluene	110	24	60%	12:1
12	$[Ir(cod)_2]BARF$	(±)-L1	*t*BuOH	toluene	110	24	60%	11:1
13	$[Ir(cod)_2]BARF$	(±)-L1	3,3-dimethyl-2-butanol	toluene	110	24	83%[b]	19:1
14	$[Ir(cod)_2]BARF$	(±)-L1	3,3-dimethyl-2-butanol	toluene	90	24	49%	12:1
15	$[Ir(cod)_2]BARF$	(±)-L1	3,3-dimethyl-2-butanol	toluene	130	24	67%	8:1
16	-	(±)-L1	3,3-dimethyl-2-butanol	toluene	110	24	n.d.	-
17	$[Ir(cod)_2]BARF$	-	3,3-dimethyl-2-butanol	toluene	110	24	n.d.	-
18	$[Ir(cod)_2]BARF$	(±)-L1	-	toluene	110	24	50%	13:1
19	$[Ir(cod)_2]BARF$	(±)-L1	3,3-dimethyl-2-butanol	-	110	24	68%	12:1
20	$[Ir(cod)_2]BARF$	(±)-L1	3,3-dimethyl-2-butanol (150 mol%)	toluene	110	24	70%	19:1
21	$[Ir(cod)_2]BARF$	(±)-L1	3,3-dimethyl-2-butanol (300 mol%)	toluene	110	24	82%	19:1
22	$[Ir(cod)_2]BARF$	(±)-L1	3,3-dimethyl-2-butanol	toluene (0.25 M)	110	24	70%	19:1

Scheme S1. Reaction Condition Screening. [a] All reactions were conducted on a 0.10 mmol scale. Yields were determined by the ^{1}H NMR of the crude mixture using 1,3,5-trimethoxybenzene as the internal standard. r.r. is the ratio of *β*:*α* selectivity and was determined by the ^{1}H NMR of the crude mixture. [b] Isolated yield.

3 Experimental Details and Characterization Data

3.1 Starting Materials

GP 1: General Procedure for the Preparation of Alcohols

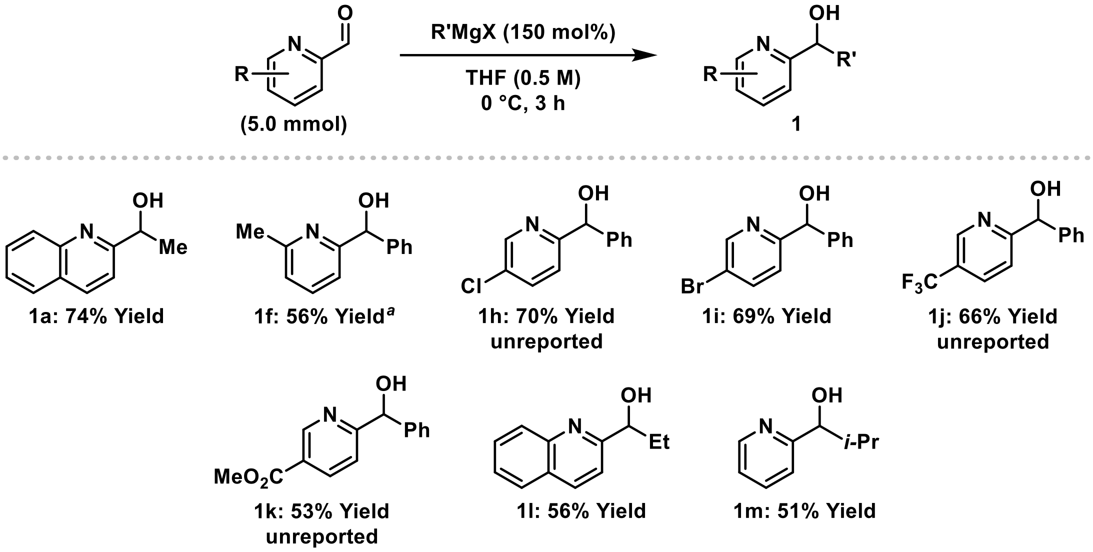

Scheme S2. Preparation of alcohols. [a] PhLi (150 mol% was used) at –78 °C for 3 h.

Alcohols were prepared according to the reported procedure.[1] A flame-dried 100 mL three-neck round-bottom flask equipped with a stirring bar was evacuated and backfilled with N_2 for 3 times. Then aldehyde (5.0 mmol, 100 mol%) and THF (10 mL, 0.5 M) were added. After cooling to 0 °C in an ice bath, Grignard reagent (7.5 mmol, 150 mol%) was added dropwise. Then the reaction mixture was stirred at this temperature for 3 hours. Water (20 mL) was added to quench the reaction and the reaction mixture was extracted with ethyl acetate (3 × 20 mL). The combined organic layer was washed with brine, dried with anhydrous $MgSO_4$, filtered, and concentrated under reduced pressure. Purification by flash column chromatography (hexane/ethyl acetate from 100/1 to 3/1) afforded the desired alcohol. Data for known alcohols were in accordance with those reported.[1,2] Data for unreported alcohols (**1h**, **1j** and **1k**) are listed below.

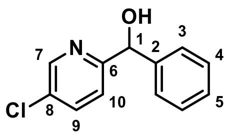

$C_{12}H_{10}ClNO$
219.66

(5-Chloropyridin-2-yl)(phenyl)methanol (**1h**, **GP 1**) was prepared from 5-chloropicolin-aldehyde (708 mg, 5.0 mmol, 100 mol%) and PhMgBr solution (2.5 mL, 3 M in Et_2O, 7.5 mmol, 150 mol%) in THF (10 mL) at 0 °C for 3 h. Purification by flash column chromatography on

silica gel (hexane/ethyl acetate from 100/1 to 3/1) afforded **1h** (769 mg, 70% yield) as a colorless oil.

R*f* = 0.25 (Hexane/EtOAc = 3/1).

^{1}H NMR (500 MHz, $CDCl_3$, 298 K): δ 8.47 (d, *J* = 2.4 Hz, 1H, H-7), 7.58 (dd, *J* = 8.5, 2.4 Hz, 1H, H-9), 7.38–7.30 (m, 4H, H-3 and H-4), 7.30–7.25 (m, 1H, H-5), 7.18 (d, *J* = 8.5 Hz, 1H, H-10), 5.75 (s, 1H, H-1), 4.97 (s, 1H, OH) ppm.

^{13}C NMR (125 MHz, $CDCl_3$, 298 K): δ 159.7 (C-6), 147.0 (C-7), 142.7 (C-2), 136.7 (C-9), 130.8 (C-8), 128.7 (C-4), 128.0 (C-5), 126.9 (C-3), 122.0 (C-7), 75.1 (C-1) ppm.

HRMS (ESI) m/z: $[M+H]^+$ calcd for $C_{12}H_{11}{}^{35}ClNO^+$ 220.0524, found 220.0521.

IR (thin film): ṽ 3340 (m), 1466 (s), 1369 (s), 1108 (s), 1013 (s) cm^{-1}.

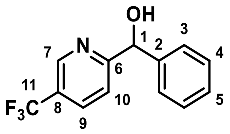

$C_{13}H_{10}F_3NO$
253.22

Phenyl(5-(trifluoromethyl)pyridin-2-yl)methanol (**1j**, **GP 1**) was prepared from 5-(trifluoromethyl)picolinaldehyde (876 mg, 5.0 mmol, 100 mol%) and PhMgBr solution (2.5 mL, 3 M in Et_2O, 7.5 mmol, 150 mol%) in THF (10 mL) at 0 °C for 3 h. Purification by flash column chromatography on silica gel (hexane/ethyl acetate from 100/1 to 5/1) afforded **1j** (836 mg, 66% yield) as a colorless oil.

R*f* = 0. 5 (Hexane/EtOAc = 3/1).

^{1}H NMR (500 MHz, $CDCl_3$, 298 K): δ 8.76 (s, 1H, H-7), 7.85 (d, *J* = 8.2 Hz, 1H, H-9), 7.43–7.32 (m, 5H, H-3, H-4, and H-10), 7.32–7.27 (m, 1H, H-5), 5.84 (s, 1H, H-1), 5.16 (s, 1H, OH) ppm.

^{13}C NMR (125 MHz, $CDCl_3$, 298 K): δ 165.5 (C-6), 145.2 (q, $J_{C–F}$ = 4.2 Hz, C-7), 142.3 (C-2), 134.1 (q, $J_{C–F}$ = 3.3 Hz, C-9), 128.8 (C-4), 128.2 (C-5), 127.0 (C-3), 125.5 (q, $J_{C–F}$ = 32.9 Hz, C-8), 123.5 (q, $J_{C–F}$ = 270.8 Hz, C-11), 121.1 (C-10), 75.5 (C-1) ppm.

^{19}F NMR (471 MHz, $CDCl_3$, 298 K): δ –62.3 (s, CF_3) ppm.

HRMS (ESI) m/z: $[M+H]^+$ calcd for $C_{13}H_{11}F_3NO^+$ 254.0787, found 254.0789.

IR (thin film): ṽ 3372 (m), 1324 (s), 1123 (s), 1077 (s) cm^{-1}.

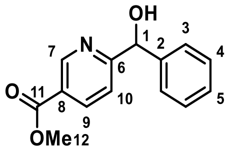

$C_{14}H_{13}NO_3$
243.26

Methyl 6-(hydroxy(phenyl)methyl)nicotinate (**1k**, **GP 1**) was prepared from methyl 6-formylnicotinate (826 mg, 5.0 mmol, 100 mol%) and PhMgBr solution (2.5 mL, 3 M in Et_2O, 7.5 mmol, 150 mol%) in THF (10 mL) at 0 °C for 3 h. Purification by flash column chromatography on silica gel (hexane/ethyl acetate from 100/1 to 3/1) afforded **1k** (645 mg, 53% yield) as a colorless solid.

R*f* = 0.25 (Hexane/EtOAc = 3/1).

M.**P**. 94–95 °C (Hexane/EtOAc).

^{1}H NMR (500 MHz, $CDCl_3$, 298 K): δ 9.17 (d, *J* = 2.3 Hz, 1H, H-7), 8.22 (dd, *J* = 8.3, 2.3 Hz, 1H, H-9), 7.39–7.31 (m, 4H, H-3 and H-4), 7.31–7.24 (m, 2H, H-5, and H-10), 5.81 (d, *J* = 4.4 Hz, 1H, H-1), 5.01 (d, *J* = 4.4 Hz, 1H, OH), 3.94 (s, 3H, H-12) ppm.

^{13}C NMR (125 MHz, $CDCl_3$, 298 K): δ 165.6 (C-11), 165.3 (C-6), 149.5 (C-7), 142.6 (C-2), 138.1 (C-9), 128.9 (C-4), 128.3 (C-5), 127.2 (C-3), 125.2 (C-8), 121.1 (C-7), 75.4 (C-1), 52.6 (C-12) ppm.

HRMS (ESI) m/z: $[M+H]^+$ calcd for $C_{14}H_{14}NO_3^+$ 244.0968, found 244.0968.

IR (thin film): ṽ 3233 (m), 1718 (s), 1597(s), 1431 (s), 1295 (s), 1115 (s), 1028 (s) cm^{-1}.

GP 2: General Procedure for the Preparation of Alcohols

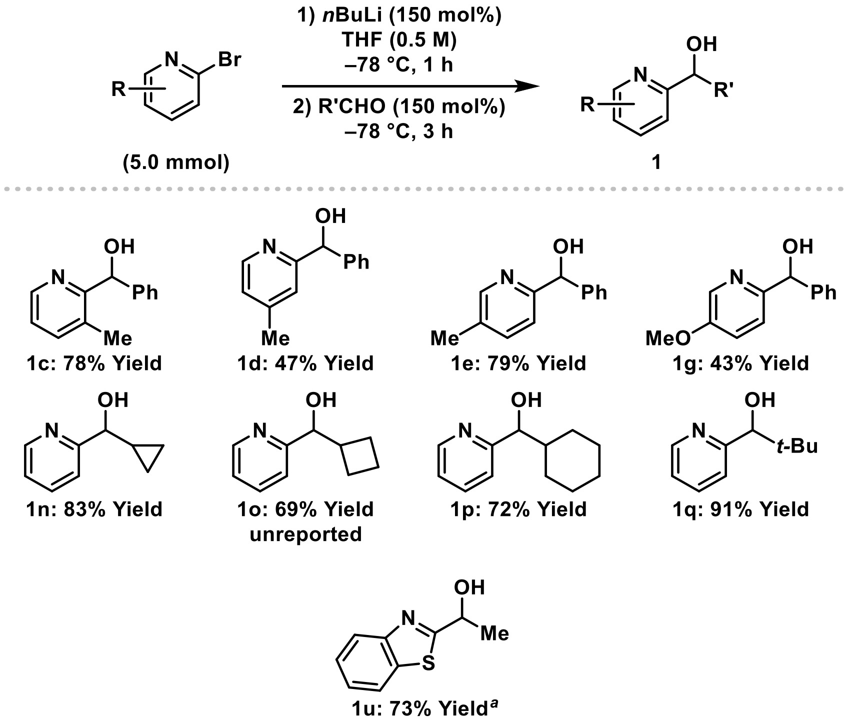

Scheme S3. Preparation of alcohols. [a] Benzothiazole or benzimidazole were used instead of bromide.

Alcohols were prepared according to the reported procedure.[3] A flame-dried 100 mL three-neck round-bottom flask equipped with a stirring bar was evacuated and backfilled with N_2 for 3 times. Then 2-bromopyridines (5.0 mmol, 100 mol%) and THF (10 mL, 0.5 M) were added. After cooling to –78 °C in a dry ice/acetone bath, *n*BuLi (3 mL, 2.5 M in hexane, 7.5 mmol, 150 mol%) was added dropwise. Then the reaction mixture was stirred at –78 °C for 1 hour. Aldehyde (0.75 mmol, 150 mol%) was added. After stirring at this temperature for 3 hours, water (20 mL) was added to quench the reaction and the reaction mixture was extracted with ethyl acetate (3 × 20 mL). The combined organic layer was washed with brine, dried with anhydrous $MgSO_4$, filtered, and concentrated under reduced pressure. Purification by flash column chromatography (hexane/ethyl acetate from 100/1 to 3/1) afforded the desired alcohol. Data for known alcohols were in accordance with those reported.[2a,4] Data for unreported alcohol **1o** are listed below.

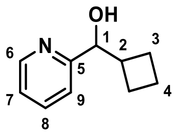

$C_{10}H_{13}NO$
163.22

Cyclobutyl(pyridin-2-yl)methanol (**1o**, **GP 2**) was prepared from 2-bromopyridines (0.48 mL, 790 mg, 5.0 mmol, 100 mol%) and cyclobutanecarbaldehyde (0.7 mL, 631 mg, 7.5 mmol, 150

mol%) in THF (10 mL) at –78 °C for 3 h. Purification by flash column chromatography on silica gel (hexane/ethyl acetate from 100/1 to 3/1) afforded **1o** (563 mg, 69% yield) as a colorless oil.

R_f = 0.2 (Hexane/EtOAc = 3/1).

^{1}H NMR (500 MHz, $CDCl_3$, 298 K): δ 8.41–8.37 (m, 1H, H-6), 7.58–7.52 (m, 1H, H-8), 7.17 (d, ^3J = 7.9 Hz, 1H, H-9), 7.09–7.04 (m, 1H, H-7), 4.70 (s, 1H, OH), 4.54 (d, ^3J = 6.7 Hz, 1H, H-1), 2.62–2.52 (m, 1H, H-2), 1.99–1.88 (m, 2H, H-3), 1.83–1.66 (m, 4H, H-3 and H-4) ppm.

^{13}C NMR (125 MHz, $CDCl_3$, 298 K): δ 161.4 (C-5), 148.1 (C-6), 136.5 (C-8), 122.2 (C-7), 120.6 (C-9), 75.9 (C-1), 41.9 (C-2), 24.1 (C-3), 23.5 (C-3), 17.9 (C-4) ppm.

HRMS (ESI) m/z: $[M+H]^+$ calcd for $C_{10}H_{14}NO^+$ 164.1070, found 164.1069.

IR (thin film): ṽ 3376 (m), 1594(s), 1435 (s), 999 (s) cm^{-1}.

GP 3: General Procedure for the Preparation of Alcohols

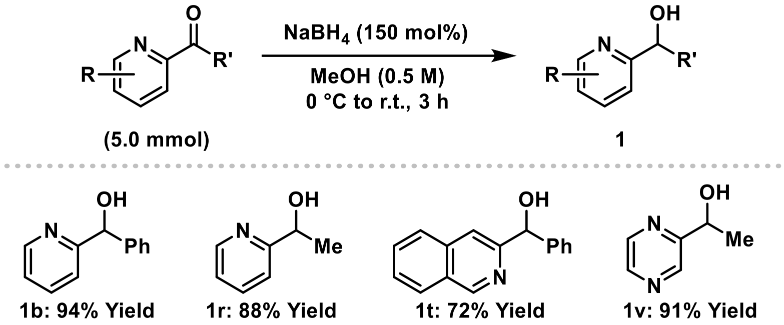

Scheme S4. Preparation of alcohols.

Alcohols were prepared according to a modified procedure.[5] A flame-dried 100 mL three-neck round-bottom flask equipped with a stirring bar was evacuated and backfilled with N_2 for 3 times. Then ketones (5.0 mmol, 100 mol%) and MeOH (10 mL, 0.5 M) were added. After cooling to 0 °C in an ice bath, $NaBH_4$ (567 mg, 0.75 mmol, 150 mol%) was added portionwise. Then the reaction mixture was stirred at room temperature for 3 hours. Water (20 mL) was added to quench the reaction at 0 °C in an ice bath and the reaction mixture was extracted with ethyl acetate (3 × 20 mL). The combined organic layer was washed with brine, dried with anhydrous $MgSO_4$, filtered, and concentrated under reduced pressure. Purification by flash column chromatography (hexane/ethyl acetate from 100/1 to 3/1) afforded the desired alcohol. Data for known alcohols were in accordance with those reported.[2d,4e,6]

GP 4: General Procedure for the Preparation of Internal Alkynes

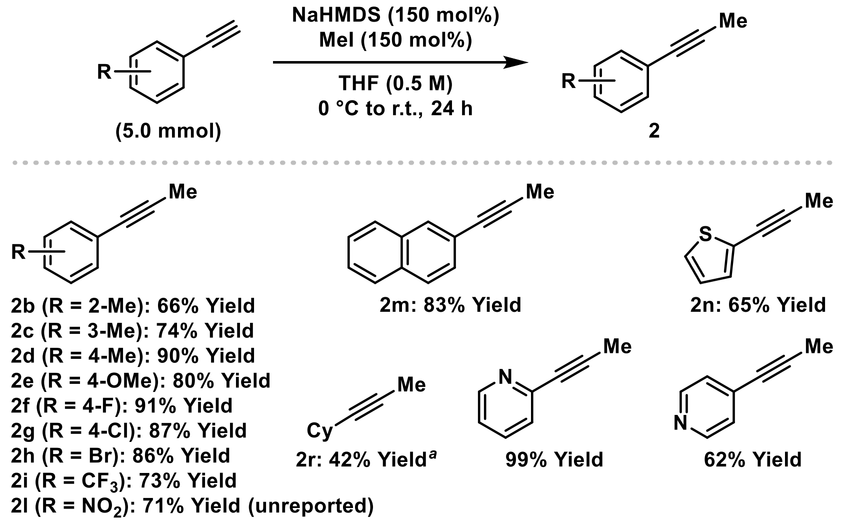

Scheme S5. Preparation of alkynes. [a] *n*-BuLi (150 mol%) was used instead of NaHMDS.

Alkynes were prepared according to the reported procedure.[7] A flame-dried 100 mL three-neck round-bottom flask equipped with a stirring bar was evacuated and backfilled with N_2 for 3 times. Then terminal alkyne (5.0 mmol, 100 mol%) and THF (10 mL, 0.5 M) were added. The resulting solution was cooled to 0 °C. NaHMDS (3.8 mL, 2 M in THF, 7.5 mmol, 150 mol%) was slowly added. The reaction mixture was then allowed to stir for 5 minutes at 0 °C. After the addition of MeI (0.5 mL, 1.07 g, 7.5 mmol, 150 mol%), the reaction mixture was warmed to room temperature and stirred for 24 hours. The reaction mixture was quenched with H_2O (20 mL) and extracted with ethyl acetate (3 × 20 mL). The combined organic layer was washed with brine, dried with anhydrous $MgSO_4$, filtered, and concentrated under reduced pressure. Purification by flash column chromatography afforded the desired internal alkyne. Data for known alkynes were in accordance with those reported.[7,8] Data for unreported alkyne **2l** are listed below.

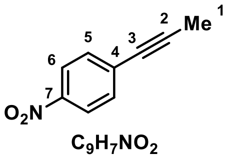

$C_9H_7NO_2$
M = 161.16

1-Nitro-4-(prop-1-yn-1-yl)benzene (**2l**, **GP 4**) was prepared from 1-ethynyl-4-nitrobenzene (736 mg, 5.0 mmol, 100 mol%) and iodomethane (0.467 mL, 1.06 g, 7.5 mmol, 150 mol%), using NaHMDS (3.8 mL, 2 M in THF, 7.5 mmol, 150 mol%) as the base in THF (10 mL, 0.5 M) at room temperature for 24 h. Purification by flash column chromatography on silica gel (hexane/ethyl acetate from 100/1 to 19/1) afforded **2l** (572 mg, 71% yield) as a brown solid.

$\mathbf{R_f}$ = 0.25 (Hexane/EtOAc = 15/1).

M.P. 103–104 °C (Hexane/EtOAc).

^{1}H NMR (500 MHz, $CDCl_3$, 298 K): δ 8.14 (d, *J* = 8.3 Hz, 2H, H-6), 7.49 (d, *J* = 8.3 Hz, 2H, H-5), 2.09 (s, 3H, H-3) ppm.

^{13}C NMR (125 MHz, $CDCl_3$, 298 K): δ 146.7 (C-7), 132.3 (C-4), 131.3 (C-5), 123.6 (C-6), 92.3 (C-2), 78.6 (C-3), 4.7 (C-1) ppm.

HRMS (ESI) m/z: $[M+H]^+$ calcd for $C_9H_8NO_2^+$ 162.0550, found 162.0553.

IR (thin film): ṽ 1715(s), 1590 (s), 1508 (s), 1337 (s), 1285 (s), 1106 (s) cm^{-1}.

GP 5: General Procedure for the Preparation of Internal Alkynes

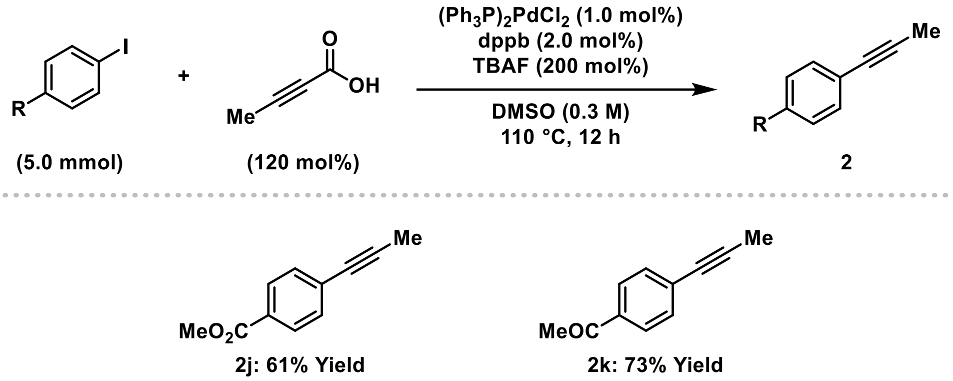

Scheme S6. Preparation of alkynes.

Alkynes were prepared according to the reported procedure.[8b] A flame-dried 100 mL three-neck round-bottom flask equipped with a stirring bar was evacuated and backfilled with N_2 for 3 times. Then $(Ph_3P)_2PdCl_2$ (35.1 mg, 50 μmol, 1.0 mol%), 2-butynoic acid (500 mg, 6.0 mmol, 120 mol%), 1,4-bis(diphenylphosphino)butane (dppb, 42.6 mg, 0.10 mmol, 2.0 mol%), ArI (5.0 mmol, 100 mol%), DMSO (15 mL, 0.3 M), and TBAF (10 mL, 1 M in THF, 10 mmol, 200 mol%) were added in senquence. The reaction mixture was degassed under N_2 for 30 min. The solution was stirred at 110 ˚C in a heating plate for 12 h. After cooling to room temperature, the reaction mixture was quenched by sat. NH_4Cl aq. (30 mL) and extracted with CH_2Cl_2 (3 × 20 mL). The combined organic layer was washed with brine, dried with anhydrous $MgSO_4$, filtered, and concentrated under reduced pressure. Purification by flash column chromatography afforded the desired internal alkyne. Data for known alkynes were in accordance with those reported.[8c]

Preparation of ((Hept-5-yn-1-yloxy)methyl)benzene

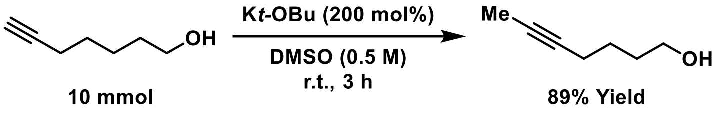

Scheme S7. Preparation of hept-5-yn-1-ol.

Hept-5-yn-1-ol was prepared according to the reported procedure.[9a] To a solution of hept-6-yn-1-ol (1.26 mL, 1.12 g, 10 mmol, 100 mol%) in DMSO (20 mL, 0.5 M) was added potassium *tert*-butoxide (2.24 g, 20 mmol, 200 mol%). The solution was stirred for 3 h at room temperature. Then the reaction was quenched by addition of HCl (5 mL, 2 M), and extracted with diethyl ether (3 × 20 mL). The combined organic layer was washed with brine, dried with anhydrous $MgSO_4$, filtered, and concentrated under reduced pressure. Column chromatography on silica (hexane/ethyl acetate from 100/1 to 6/1) afforded hept-5-yn-1-ol (998 mg, 89% yield) as a colorless oil. Data were in accordance with those reported.[9a]

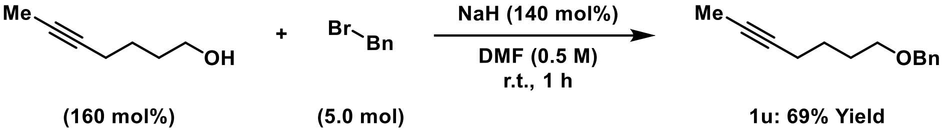

Scheme S8. Preparation of ((hept-5-yn-1-yloxy)methyl)benzene.

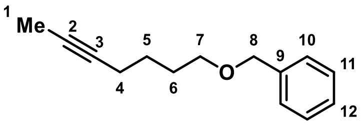

$C_{14}H_{18}O$
M = 202.30

((Hept-5-yn-1-yloxy)methyl)benzene was prepared according to the reported procedure.[9b] To a solution of hept-5-yn-1-ol (897 mg, 8.0 mmol, 160 mol%) in DMF (10 mL, 0.5 M) was added 60% NaH (280 mg, 7 mmol, 140 mol%). After 10 min, BnCl (595 μL, 855 mg, 5.0 mmol, 100 mol%) was added. The reaction was stirred for 1 h at room temperature and then quenched with water. The mixture was diluted with ether and then washed with water and brine. The organic layer was dried with anhydrous $MgSO_4$, filtered, and concentrated under reduced pressure. Column chromatography on silica (hexane/ethyl acetate from 100/1 to 19/1) afforded ((hept-5-yn-1-yloxy)methyl)benzene (**1u**, 701 mg, 69% yield) as a colorless oil.

$\mathbf{R_f}$ = 0.5 (Hexane/EtOAc = 15/1).

^{1}H NMR (500 MHz, $CDCl_3$, 298 K): δ 7.38–7.33 (m, 4H, H-10 and H-11), 7.32–7.26 (m, 1H, H-12), 4.51 (s, 2H, H-8), 3.50 (t, J = 6.5 Hz, 2H, H-7), 2.19–2.14 (m, 2H, H-4), 1.78 (t, J = 2.6 Hz, 3H, H-1), 1.76–1.70 (m, 2H, H-6), 1.62–1.55 (m, 2H, H-5) ppm.

^{13}C NMR (125 MHz, $CDCl_3$, 298 K): δ 138.7 (C-9), 128.5 (C-11), 127.7 (C-10), 127.6 (C-12), 79.1 (C-3), 75.8 (C-2), 73.0 (C-8), 70.0 (C-7), 29.0 (C-6), 25.9 (C-5), 18.7 (C-4), 3.6 (C-1) ppm.

HRMS (ESI) m/z: $[M+H]^+$ calcd for $C_{14}H_{19}O^+$ 203.1430, found 203.1429.

IR (thin film): ṽ 2918 (s), 2958 (s), 1453 (s), 1361 (s), 1204 (s), 1103 (s) cm^{-1}.

Preparation of 7-Chlorohept-2-yne

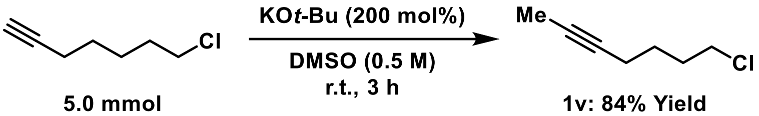

5.0 mmol

1v: 84% Yield

Scheme S9. Preparation of 7-Chlorohept-2-yne.

7-Chlorohept-2-yne was prepared according to the reported procedure.[9a] To a solution of 7-chlorohept-1-yne (1.39 mL, 1.31 g, 10 mmol, 100 mol%) in DMSO (20 mL, 0.5 M) was added potassium *tert*-butoxide (2.24 g, 20 mmol, 200 mol%). The solution was stirred for 3 h at room temperature. Then the reaction was quenched by addition of HCl (5 mL, 2 M), and extracted with diethyl ether (3 × 20 mL). The combined organic layer was washed with brine, dried with anhydrous $MgSO_4$, filtered, and concentrated under reduced pressure. Column chromatography on silica (hexane) afforded the desired 7-Chlorohept-2-yne (**1v**, 1.10 g, 84% yield) as a colorless oil. Data were in accordance with those reported.[9c]

Preparation of [Ir(cod)$_2$]BARF

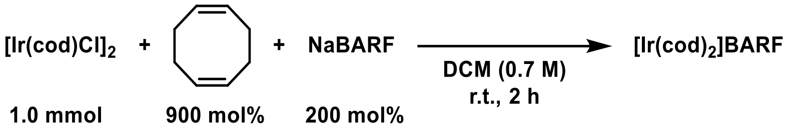

Scheme S10. Preparation of [Ir(cod)$_2$]BARF.

To a solution of chloro(1,5-cyclooctadiene)iridium(I) dimer (672 mg, 1.0 mmol, 100 mol%) and NaBARF (1.77 g, 2.0 mmol, 200 mol%) in CH_2Cl_2 (15 mL, 0.7 M) was added 1,5-cyclooctadiene (1.1 mL, 978 mg, 9.0 mmol, 900 mol%). The reaction mixture was stirred at r.t. for 2 hours and then filtered through Celite. Solvent was removed in vacuo and the residue was dried under vacuum (0.01 mmHg) overnight to afford the desired product (1.23 g, 97% yield) as a burgundy solid. This compound was stored under argon at –20 °C. Data were in accordance with those reported.[10]

^{1}H NMR ($CDCl_3$, 500 MHz): δ 7.70 (8H, s), 7.55 (4H, s), 4.99 (8H, s), 2.47–2.32 (m, 8H), 2.32–2.19 (m, 8H) ppm.

^{13}C NMR ($CDCl_3$, 125 MHz): 161.8 (1:1:1:1 pattern, $J_{C–B}$ = 49.2 Hz), 134.9, 129.1 (qq, $J_{C–F}$ = 31.3 Hz, $J_{C–F}$ = 2.9 Hz), 124.7 (q, $J_{C–F}$ = 270.8 Hz), 117.7, 101.2, 30.5.

^{19}F NMR ($CDCl_3$, 471 MHz): δ –62.3 ppm.

3.2 Products

GP 6: General Procedure for Iridium-Catalyzed Enantioselective C-H Alkylations/Alkenylations of Secondary Alcohols

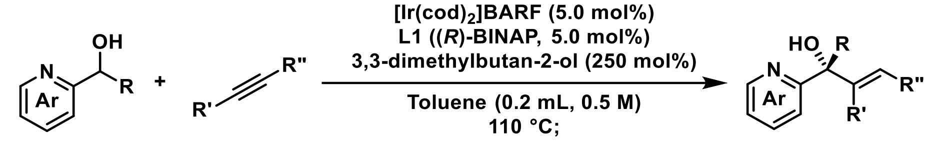

Scheme S11. Iridium-catalyzed enantioselective C-H alkenylations of secondary alcohols.

A flame-dried *Schlenk* tube was charged with alcohol (0.10 mmol, 100 mol%), [Ir(cod)$_2$]BARF (5.0 μmol, 5.0 mol%) and **L1** ((*R*)-BINAP, 5.0 μmol, 5.0 mol%). The *Schlenk* tube was evacuated and backfilled with N_2 for three times. Then toluene (0.2 mL, 0.5 M) was added, followed by the addition of styrene or alkyne (0.25 mmol, 250 mol%) and 3,3-dimethylbutan-2-ol (32 μL, 25.5 mg, 0.25 mmol, 250 mol%). The tube was sealed and stirred at 110 °C in a heating plate for the indicated time. After cooling to room temperature, the solvent was removed under reduced pressure and the crude reaction mixture was purified by flash column chromatography under the indicated conditions.

Note: All *racemic* compounds are synthesized according to the method outlined above, by using (±)-BINAP instead of (*R*)-BINAP.

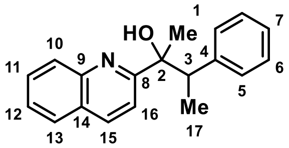

$C_{19}H_{19}NO$
M = 277.37

3-Phenyl-2-(quinolin-2-yl)butan-2-ol (**5**, **GP 6**) was prepared from 1-(quinolin-2-yl)ethan-1-ol (**1a**, 17.3 mg, 0.10 mmol, 100 mol%) and styrene (**4**, 28.7 μL, 26.0 mg, 0.25 mmol, 250 mol%), using [Ir(cod)$_2$]BARF (6.36 mg, 5.0 μmol, 5.0 mol%), **L1** (3.11 mg, 5.0 μmol, 5.0 mol%) and *i*-PrOH (19.1 μL, 15.0 mg, 0.25 mmol, 250 mol%) in toluene (0.2 mL, 0.5 M) at 110 °C for 24 h. Purification by flash column chromatography on silica gel (hexane/ethyl acetate from 100/1 to 19/1) afforded **5** as two separated diastereomers. Diastereomer and regiomer ratios (1:1 d.r., >20:1 r.r.) were determined from the ^{1}H NMR spectrum of crude material.

Diastereomers 1 (13.0 mg, 47% yield, 81% ee, >25:1 d.r., >25:1 r.r.) as a colorless solid

R*f* = 0.3 (Hexane/EtOAc = 15/1).

M.P. 102–103 °C (Hexane/EtOAc).

^{1}H NMR (500 MHz, $CDCl_3$, 298 K): δ 8.19 (d, *J* = 8.6 Hz, 1H, H-15), 8.12 (d, *J* = 8.4 Hz, 1H, H-10), 7.86 (d, *J* = 8.1 Hz, 1H, H-13), 7.78–7.73 (m, 1H, H-11), 7.59–7.55 (m, 1H, H-12), 7.46 (d, *J* = 8.6 Hz, 1H, H-16), 7.43 (d, *J* = 7.1 Hz, 2H, H-5), 7.35–7.30 (m, 2H, H-6), 7.28–7.23 (m, 1H, H-7), 6.04 (s, 1H, OH), 3.17 (q, *J* = 7.1 Hz, 1H, H-3), 1.37 (s, 3H, H-1), 1.01 (d, *J* = 7.1 Hz, 3H, H-17) ppm.

^{13}C NMR (125 MHz, $CDCl_3$, 298 K): δ 164.9 (C-8), 145.7 (C-9), 143.5 (C-4), 137.4 (C-15), 130.0 (C-11), 129.5 (C-5), 129.0 (C-10), 128.1 (C-6), 127.6 (C-13), 127.2 (C-14), 126.7 (C-7), 126.6 (C-12), 117.6 (C-16), 76.0 (C-2), 50.2 (C-3), 27.8 (C-1), 16.4 (C-17) ppm.

HRMS (ESI) m/z: $[M+H]^+$ calcd for $C_{19}H_{20}NO^+$ 278.1540, found 278.1550.

IR (thin film): ṽ 3338 (s), 2963 (s), 2926 (s), 1598 (s), 1364 (s), 1387 (s), 1152 (s), 1021 (s) cm^{-1}.

Specific rotation: $[\alpha]_D^{24}$ = −8.3 (*c* 0.5, CH_2Cl_2).

The **enantiomeric ratio** of **diastereomer 1** was determined by SFC analysis (CHIRALPACK SC (25 cm), column temperature 25 °C, solvent CO_2/MeOH = 95/5, flow rate = 2.0 mL/min): t_R = 6.3 min (major), t_R = 7.0 min (minor).

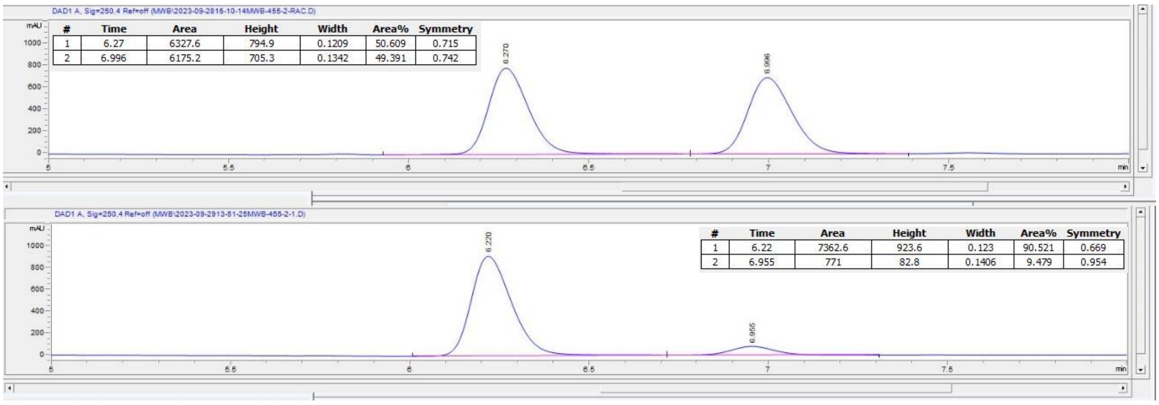

Diastereomer 2 (12.9 mg, 46% yield, 29% ee, >25:1 d.r., >25:1 r.r.) as a colorless oil

R_f = 0.3 (Hexane/EtOAc = 15/1).

^{1}H NMR (500 MHz, $CDCl_3$, 298 K): δ 8.06 (d, *J* = 8.6 Hz, 1H, H-15), 7.89 (d, *J* = 8.5 Hz, 1H, H-10), 7.76 (d, *J* = 8.1 Hz, 1H, H-13), 7.66–7.61 (m, 1H, H-11), 7.50–7.46 (m, 1H, H-12), 7.30 (d, *J* = 8.6 Hz, 1H, H-16), 7.03–6.96 (m, 5H, H-5, H-6 and H-7), 6.79 (s, 1H, OH), 3.28 (q, *J* = 7.1 Hz, 1H, H-3), 1.68 (s, 3H, H-1), 1.48 (d, *J* = 7.1 Hz, 3H, H-17) ppm.

^{13}C NMR (125 MHz, $CDCl_3$, 298 K): δ 164.0 (C-8), 145.6 (C-9), 143.0 (C-4), 136.5 (C-15), 129.6 (C-11), 129.0 (C-5), 128.9 (C-10), 127.5 (C-6), 127.4 (C-13), 126.9 (C-14), 126.4 (C-7), 126.2 (C-12), 118.4 (C-16), 76.1 (C-2), 50.1 (C-3), 27.1 (C-1), 15.9 (C-17) ppm.

HRMS (ESI) m/z: $[M+H]^+$ calcd for $C_{19}H_{20}NO^+$ 278.1540, found 278.1540.

IR (thin film): ṽ 3362 (br), 2973 (m), 1600 (s), 1504 (s), 1388 (s), 1363 (s), 1146 (s) cm^{-1}.

Specific rotation: $[\alpha]_D^{24}$ = +6.2 (c 0.5, CH_2Cl_2).

The **enantiomeric ratio** of **diastereomer 2** was determined by SFC analysis (CHIRALPACK SC (25 cm), column temperature 25 °C, solvent CO_2/MeOH = 95/5, flow rate = 2.0 mL/min): t_R = 5.8 min (major), t_R = 6.2 min (minor).

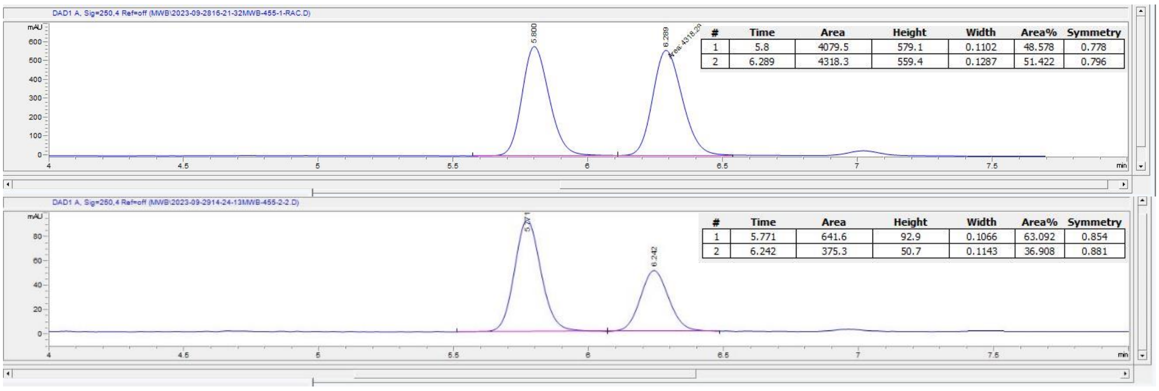

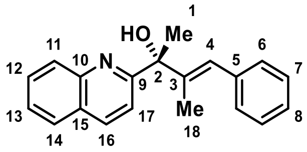

$C_{20}H_{19}NO$
M = 289.38

(*R*,*E*)-3-Methyl-4-phenyl-2-(quinolin-2-yl)but-3-en-2-ol (**3aa**, **GP 6**) was prepared from 1-(quinolin-2-yl)ethan-1-ol (**1a**, 17.3 mg, 0.10 mmol, 100 mol%) and 1-phenyl-1-propyne (**2a**, 31.6 µL, 29.0 mg, 0.25 mmol, 250 mol%), using $[Ir(cod)_2]$BARF (6.36 mg, 5.0 µmol, 5.0 mol%), **L1** (3.11 mg, 5.0 µmol, 5.0 mol%) and 3,3-dimethylbutan-2-ol (32 µL, 25.5 mg, 0.25 mmol, 250 mol%) in toluene (0.2 mL, 0.5 M) at 110 °C for 24 h. Purification by flash column chromatography on silica gel (hexane/ethyl acetate from 100/1 to 19/1) afforded **3aa** (24.0 mg, 83% yield, 99% ee, >25:1 r.r.) as a colorless solid. The regioisomer ratio of the process (19:1 r.r.) was determined from the ^{1}H NMR spectrum of crude material.

R_f = 0.4 (Hexane/EtOAc = 15/1).

M.P. 64–65 °C (Hexane/EtOAc).

^{1}H NMR (500 MHz, $CDCl_3$, 298 K): δ 8.16 (d, *J* = 8.6 Hz, 1H, H-16), 8.13 (d, *J* = 8.5 Hz, 1H, H-11), 7.85 (d, *J* = 8.1 Hz, 1H, H-14), 7.78–7.74 (m, 1H, H-12), 7.59–7.55 (m, 1H, H-13), 7.47 (d, *J* = 8.6 Hz, 1H, H-17), 7.37–7.31 (m, 4H, H-6 and H-7), 7.25–7.21 (m, 1H, H-8), 6.88 (s, 1H, H-4), 6.39 (s, 1H, OH), 1.84 (s, 3H, H-1), 1.67 (s, 3H, H-18) ppm.

^{13}C NMR (125 MHz, $CDCl_3$, 298 K): δ 163.7 (C-9), 145.9 (C-10), 142.5 (C-3), 138.1 (C-5), 137.5 (C-16), 130.0 (C-12), 129.3 (C-6), 128.9 (C-11), 128.2 (C-7), 127.6 (C-14), 127.4 (C-15), 126.7 (C-13), 126.6 (C-8), 126.2 (C-4), 118.0 (C-17), 77.0 (C-2), 27.8 (C-1), 14.4 (C-18) ppm.

HRMS (ESI) m/z: $[M+H]^+$ calcd for $C_{20}H_{20}NO^+$ 290.1539, found 290.1531.

IR (thin film): ṽ 3357 (br), 2970 (s), 2930 (s), 1617(s), 1598 (s), 1504 (s), 1364 (s), 1114 (s), 1076 (s) cm^{-1}.

Specific rotation: $[\alpha]_D^{25}$ = −245.7 (*c* 0.5, CH_2Cl_2).

The **enantiomeric ratio** of **3aa** was determined by SFC analysis (CHIRALPACK IE (25 cm), column temperature 25 °C, solvent CO_2/MeOH = 95/5, flow rate = 2.0 mL/min): t_R = 28.1 min (minor), t_R = 29.8 min (major).

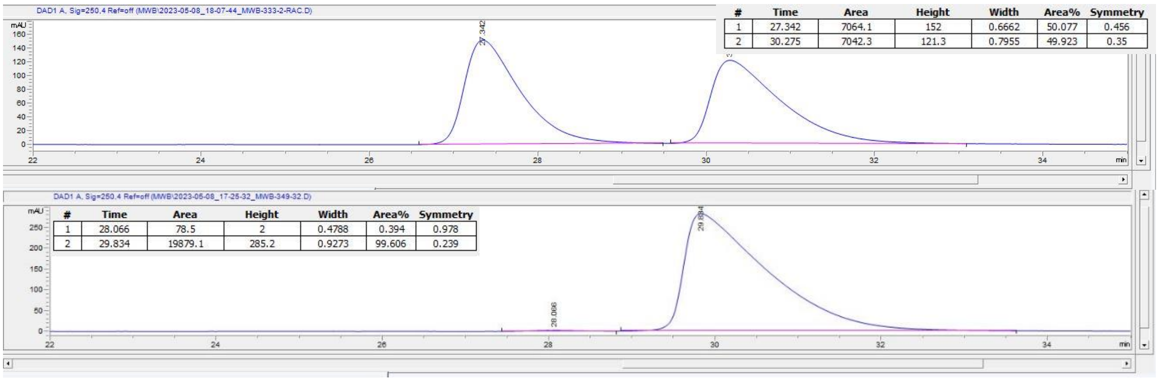

#	Time	Area	Height	Width	Area%	Symmetry
1	27.342	7064.1	152	0.6662	50.077	0.456
2	30.275	7042.3	121.3	0.7955	49.923	0.35

#	Time	Area	Height	Width	Area%	Symmetry
1	28.066	78.5	2	0.4788	0.394	0.978
2	29.834	19879.1	285.2	0.9273	99.606	0.239

(*S*,*E*)-3-Methyl-4-phenyl-2-(quinolin-2-yl)but-3-en-2-ol (***ent*-3aa**, **GP 6**) was prepared from 1-(quinolin-2-yl)ethan-1-ol (**1a**, 17.3 mg, 0.10 mmol, 100 mol%) and 1-phenyl-1-propyne (**2a**, 31.6 μL, 29.0 mg, 0.25 mmol, 250 mol%), using $[Ir(cod)_2]$BARF (6.36 mg, 5.0 μmol, 5.0 mol%), (*ent*)-**L1** ((*S*)-BINAP, 3.11 mg, 5.0 μmol, 5.0 mol%) and 3,3-dimethylbutan-2-ol (32 μL, 25.5 mg, 0.25 mmol, 250 mol%) in toluene (0.2 mL, 0.5 M) at 110 °C for 24 h. Purification by flash column chromatography on silica gel (hexane/ethyl acetate from 100/1 to 19/1) afforded **3aa** (24.0 mg, 82% yield, 98% ee, 19:1 r.r.) as a colorless solid.

Specific rotation: $[\alpha]_D^{23}$ = +231.9 (*c* 0.5, CH_2Cl_2).

The **enantiomeric ratio** of *ent*-**3aa** was determined by SFC analysis (CHIRALPACK IE (25 cm), column temperature 25 °C, solvent CO_2/MeOH = 95/5, flow rate = 2.0 mL/min): t_R = 26.5 min (major), t_R = 30.2 min (minor).

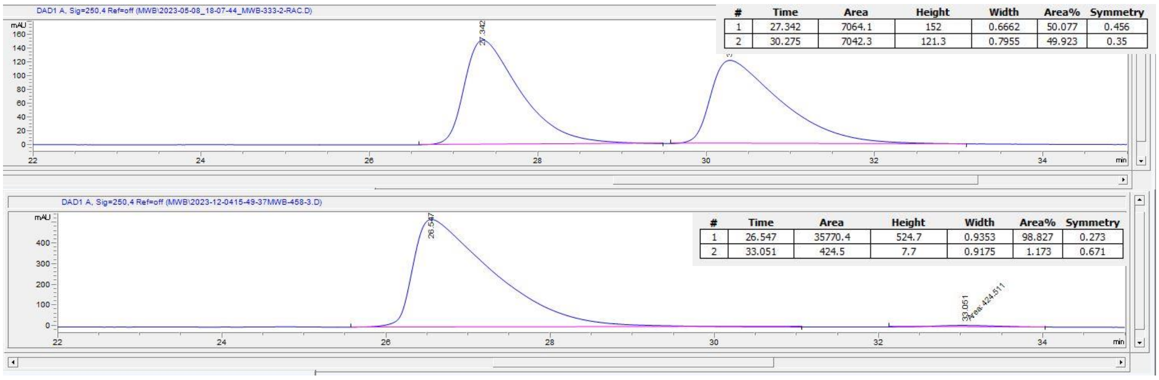

Characteristic signals for regioisomer *iso*-**3aa**

^{1}H NMR (500 MHz, $CDCl_3$, 298 K): 6.06 (q, J = 6.8 Hz, 1H), 1.47 (d, J = 6.8 Hz, 3H).

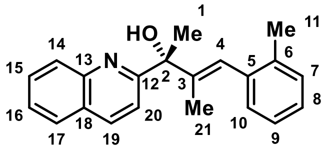

$C_{21}H_{21}NO$
M = 303.41

(*R*,*E*)-3-Methyl-2-(quinolin-2-yl)-4-(*o*-tolyl)but-3-en-2-ol (**3ab**, **GP 6**) was prepared from 1-(quinolin-2-yl)ethan-1-ol (**1a**, 17.3 mg, 0.10 mmol, 100 mol%) and 1-methyl-2-(prop-1-yn-1-yl)benzene (**2b**, 65.1 mg, 0.50 mmol, 500 mol%), using [Ir(cod)$_2$]BARF (12.7 mg, 10 μmol, 10 mol%), **L1** (6.23 mg, 10 μmol, 10 mol%) and 3,3-dimethylbutan-2-ol (32 μL, 25.5 mg, 0.25 mmol, 250 mol%) in toluene (0.2 mL, 0.5 M) at 110 °C for 48 h. Purification by flash column chromatography on silica gel (hexane/ethyl acetate from 100/1 to 19/1) afforded **3ab** (16.4 mg, 54% yield, 98% ee, >25:1 r.r.) as a colorless oil. The regioisomer ratio of the process (9:1 r.r.) was determined from the ^{1}H NMR spectrum of crude material.

R_f = 0.4 (Hexane/EtOAc = 15/1).

^{1}H NMR (500 MHz, $CDCl_3$, 298 K): δ 8.18 (d, J = 8.6 Hz, 1H, H-19), 8.13 (d, J = 8.5 Hz, 1H, H-14), 7.85 (d, J = 8.1 Hz, 1H, H-17), 7.78–7.73 (m, 1H, H-15), 7.59–7.55 (m, 1H, H-16), 7.50 (d, J = 8.6 Hz, 1H, H-20), 7.21–7.13 (m, 4H, H-7, H-8, H-9 and H-10), 6.84 (s, 1H, H-4), 6.37 (s, 1H, OH), 2.31 (s, 3H, H-11), 1.85 (s, 3H, H-1), 1.49 (s, 3H, H-21) ppm.

^{13}C NMR (125 MHz, $CDCl_3$, 298 K): δ 163.7 (C-12), 145.9 (C-13), 142.8 (C-3), 137.5 (C-5), 137.4 (C-19), 136.5 (C-6), 130.1 (C-15), 129.9 (C-7), 129.4 (C-10), 129.0 (C-14), 127.7 (C-17),

127.4 (C-18), 126.9 (Ar), 126.8 (C-16), 125.5 (Ar), 125.4 (C-4), 117.9 (C-20), 76.9 (C-2), 27.8 (C-1), 20.2 (C-11), 13.9 (C-21) ppm.

HRMS (ESI) m/z: $[M+H]^+$ calcd for $C_{21}H_{22}NO^+$ 304.1696, found 304.1697.

IR (thin film): ṽ 3371 (br), 1600 (s), 1504 (s), 1367 (s), 1122 (s), 1074 (s) cm^{-1}.

Specific rotation: $[\alpha]_D^{25}$ = −200.8 (*c* 0.5, CH_2Cl_2).

The **enantiomeric ratio** of **3ab** was determined by SFC analysis (CHIRALPACK IE (25 cm), column temperature 25 °C, solvent CO_2/MeOH (with 0.5% Et_3N) = 90/10, flow rate = 2.0 mL/min): t_R = 8.1 min (minor), t_R = 9.4 min (major).

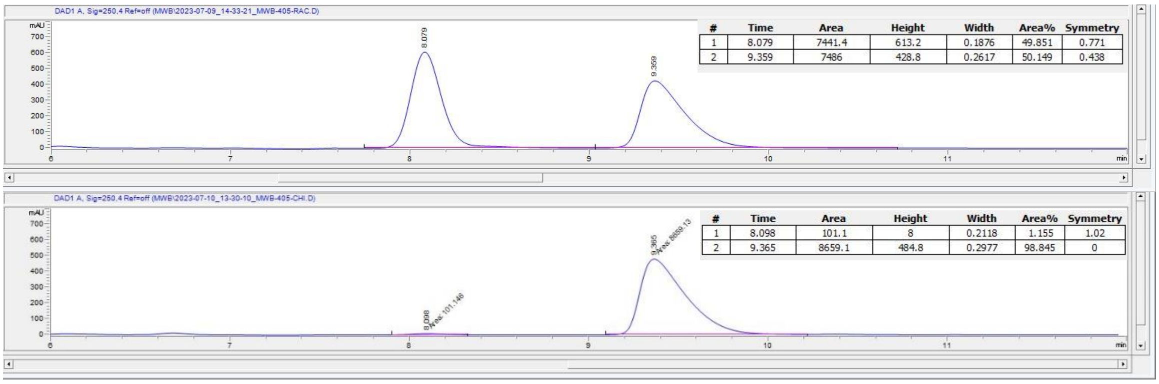

Characteristic signal for regioisomer *iso*-**3ab**

^{1}H NMR (500 MHz, $CDCl_3$, 298 K): 1.43 (d, *J* = 7.0 Hz, 3H).

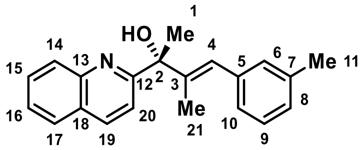

$C_{21}H_{21}NO$
M = 303.41

(*R*,*E*)-3-Methyl-2-(quinolin-2-yl)-4-(*m*-tolyl)but-3-en-2-ol (**3ac**, **GP 6**) was prepared from 1-(quinolin-2-yl)ethan-1-ol (**1a**, 17.3 mg, 0.10 mmol, 100 mol%) and 1-methyl-3-(prop-1-yn-1-yl)benzene (**2c**, 32.5 mg, 0.25 mmol, 250 mol%), using $[Ir(cod)_2]$BARF (6.36 mg, 5.0 µmol, 5.0 mol%), **L1** (3.11 mg, 5.0 µmol, 5.0 mol%) and 3,3-dimethylbutan-2-ol (32 µL, 25.5 mg, 0.25 mmol, 250 mol%) in toluene (0.2 mL, 0.5M) at 110 °C for 24 h. Purification by flash column chromatography on silica gel (hexane/ethyl acetate from 100/1 to 19/1) afforded **3ac** (18.6 mg, 61% yield, 99% ee, >25:1 r.r.) as a colorless solid. The regioisomer ratio of the process (14:1 r.r.) was determined from the ^{1}H NMR spectrum of crude material.

R_f = 0.4 (Hexane/EtOAc = 15/1).

M.P. 77–78 °C (Hexane/EtOAc).

^{1}H NMR (500 MHz, $CDCl_3$, 298 K): δ 8.15 (d, *J* = 8.6 Hz, 1H, H-19), 8.13 (d, *J* = 8.5 Hz, 1H, H-14), 7.84 (d, *J* = 8.2 Hz, 1H, H-17), 7.78–7.73 (m, 1H, H-15), 7.59–7.55 (m, 1H, H-16), 7.46 (d, *J* = 8.6 Hz, 1H, H-20), 7.26–7.21 (m, 1H, ArH), 7.15–7.11 (m, 2H, H-6 and ArH), 7.05 (d, *J* = 7.6 Hz, 1H, ArH), 6.84 (s, 1H, H-4), 6.36 (s, 1H, OH), 2.36 (s, 3H, H-11), 1.82 (s, 3H, H-1), 1.66 (s, 3H, H-21) ppm.

^{13}C NMR (125 MHz, $CDCl_3$, 298 K): δ 163.8 (C-12), 145.9 (C-13), 142.3 (C-3), 138.1 (C-5), 137.8 (C-7), 137.5 (C-19), 130.0 (C-15), 130.0 (C-6), 128.9 (C-14), 128.1 (Ar), 127.6 (C-18), 127.4 (C-17), 126.7 (C-16), 126.3 (Ar), 126.2 (C-4), 118.0 (C-20), 77.1 (C-2), 27.8 (C-1), 21.6 (C-11), 14.4 (C-21) ppm.

HRMS (ESI) m/z: $[M+H]^+$ calcd for $C_{21}H_{22}NO^+$ 304.1696, found 304.1695.

IR (thin film): ṽ 3306 (br), 1599 (s), 1504 (s), 1369 (s), 1120 (s), 1075 (s) cm^{-1}.

Specific rotation: $[\alpha]_D^{25}$ = −215.24 (*c* 0.5, CH_2Cl_2).

The **enantiomeric ratio** of **3ac** was determined by SFC analysis (CHIRALPACK SB (25 cm), column temperature 25 °C, solvent CO_2/MeOH (with 0.5% Et_3N) = 98/2, flow rate = 2.5 mL/min): t_R = 19.8 min (major), t_R = 21.8 min (minor).

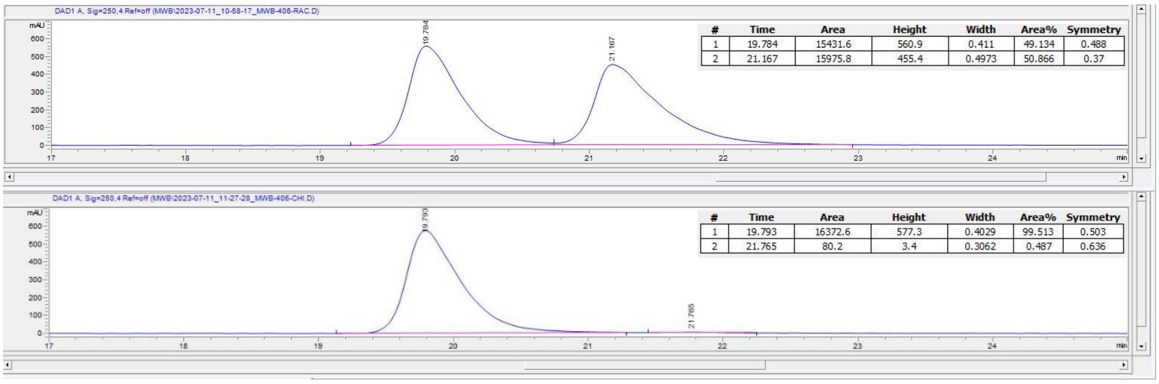

Characteristic signals for regioisomer *iso*-**3ac**

^{1} H NMR (500 MHz, $CDCl_3$, 298 K): 6.01 (q, *J* = 6.8 Hz, 1H), 1.46 (d, *J* = 6.8 Hz, 3H).

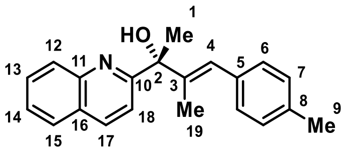

$C_{21}H_{21}NO$
M = 303.41

(*R*,*E*)-3-Methyl-2-(quinolin-2-yl)-4-(*p*-tolyl)but-3-en-2-ol (**3ad**, **GP 6**) was prepared from 1-(quinolin-2-yl)ethan-1-ol (**1a**, 17.3 mg, 0.10 mmol, 100 mol%) and 1-methyl-4-(prop-1-yn-1-yl)benzene (**2d**, 32.5 mg, 0.25 mmol, 250 mol%), using [Ir(cod)$_2$]BARF (6.36 mg, 5.0 μmol, 5.0 mol%), **L1** (3.11 mg, 5.0 μmol, 5.0 mol%) and 3,3-dimethylbutan-2-ol (32 μL, 25.5 mg, 0.25 mmol, 250 mol%) in toluene (0.2 mL, 0.5 M) at 110 °C for 24 h. Purification by flash column chromatography on silica gel (hexane/ethyl acetate from 100/1 to 19/1) afforded **3ad** (22.5 mg, 74% yield, 98% ee, >25:1:1 r.r.) as a brown solid. The regioisomer ratio of the process (11:1 r.r.) was determined from the ^{1}H NMR spectrum of crude material.

R*f* = 0.4 (Hexane/EtOAc = 15/1).

M.P. 67–68 °C (Hexane/EtOAc).

^{1}H NMR (500 MHz, $CDCl_3$, 298 K): δ 8.15 (d, *J* = 8.6 Hz, 1H, H-17), 8.12 (d, *J* = 8.5 Hz, 1H, H-12), 7.84 (d, *J* = 8.2 Hz, 1H, H-15), 7.78–7.73 (m, 1H, H-13), 7.59–7.54 (m, 1H, H-14), 7.45 (d, *J* = 8.6 Hz, 1H, H-18), 7.22 (d, *J* = 8.0 Hz, 2H, H-6), 7.15 (d, *J* = 8.0 Hz, 2H, H-7), 6.83 (s, 1H, H-4), 6.36 (s, 1H, OH), 2.35 (s, 3H, H-9), 1.82 (s, 3H, H-1), 1.65 (s, 3H, H-19) ppm.

^{13}C NMR (125 MHz, $CDCl_3$, 298 K): δ 163.8 (C-10), 145.9 (C-11), 141.7 (C-3), 137.5 (C-17), 136.3 (C-8), 135.2 (C-5), 130.0 (C-13), 129.2 (C-6), 128.9 (C-12), 128.9 (C-7), 127.7 (C-15), 127.4 (C-16), 126.7 (C-14), 126.1 (C-4), 118.0 (C-18), 77.1 (C-2), 27.8 (C-1), 21.3 (C-9), 14.4 (C-19) ppm.

HRMS (ESI) m/z: $[M+H]^+$ calcd for $C_{21}H_{22}NO^+$ 304.1696, found 304.1699.

IR (thin film): ṽ 3367 (br), 1599 (s), 1504 (s), 1367 (s), 1111 (s), 1074 (s) cm^{-1}.

Specific rotation: $[\alpha]_D^{25}$ = −266.3 (*c* 0.5, CH_2Cl_2).

The **enantiomeric ratio** of **3ad** was determined by SFC analysis (CHIRALPACK IE (25 cm), column temperature 25 °C, solvent CO_2/MeOH (with 0.5% Et_3N) = 95/5, flow rate = 2.0 mL/min): t_R = 11.7 min (minor), t_R = 13.0 min (major).

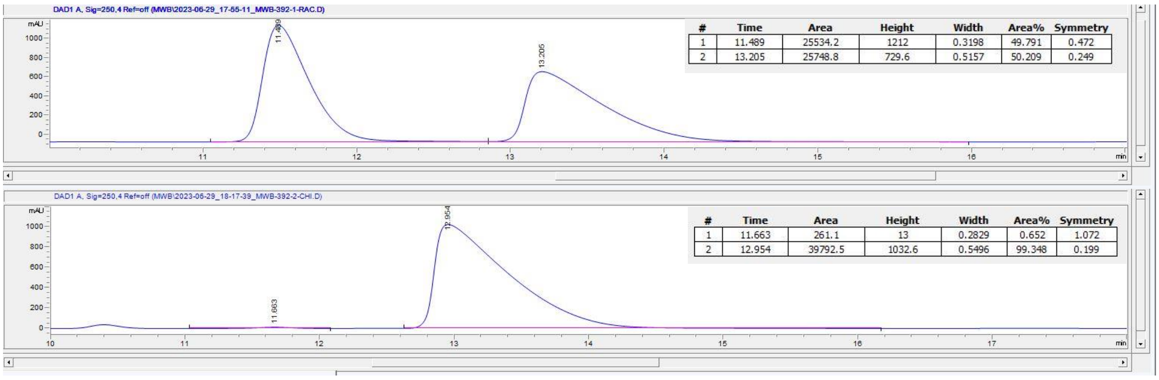

Characteristic signals for regioisomer *iso*-**3ad**

^{1} **H NMR** (500 MHz, $CDCl_3$, 298 K): 6.04 (q, *J* = 6.9 Hz, 1H), 1.46 (d, *J* = 6.9 Hz, 3H).

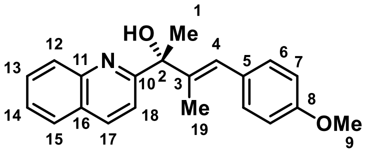

$C_{21}H_{21}NO_2$
M = 319.40

(*R*,*E*)-4-(4-Methoxyphenyl)-3-methyl-2-(quinolin-2-yl)but-3-en-2-ol (**3ae**, **GP 6**) was prepared from 1-(quinolin-2-yl)ethan-1-ol (**1a**, 17.3 mg, 0.10 mmol, 100 mol%) and 1-methoxy-4-(prop-1-yn-1-yl)benzene (**3e**, 73.1 mg, 0.50 mmol, 500 mol%), using [Ir(cod)$_2$]BARF (12.7 mg, 10 μmol, 10 mol%), **L1** (6.23 mg, 10 μmol, 10 mol%) and 3,3-dimethylbutan-2-ol (32 μL, 25.5 mg, 0.25 mmol, 250 mol%) in toluene (0.2 mL, 0.5 M) at 110 °C for 24 h. Purification by flash column chromatography on silica gel (hexane/ethyl acetate from 100/1 to 19/1) afforded **3ae** (21.1 mg, 66% yield, 98% ee, >25:1 r.r.) as a yellow solid. The regioisomer ratio of the process (7:1 r.r.) was determined from the ^{1}H NMR spectrum of crude material.

R*f* = 0.2 (Hexane/EtOAc = 15/1).

M.P. 100–101 °C (Hexane/EtOAc).

^{1}**H NMR** (500 MHz, $CDCl_3$, 298 K): δ 8.14 (d, *J* = 8.6 Hz, 1H, H-17), 8.12 (d, *J* = 8.5 Hz, 1H, H-12), 7.84 (d, *J* = 8.1 Hz, 1H, H-15), 7.78–7.73 (m, 1H, H-13), 7.59–7.54 (m, 1H, H-14), 7.44 (d, *J* = 8.6 Hz, 1H, H-18), 7.29–7.24 (m, 2H, H-6), 6.88 (d, *J* = 8.7 Hz, 2H, H-7), 6.80 (s, 1H, H-4), 6.35 (s, 1H, OH), 3.82 (s, 3H, H-9), 1.81 (s, 3H, H-1), 1.65 (s, 3H, H-19) ppm.

^{13}**C NMR** (125 MHz, $CDCl_3$, 298 K): δ 163.9 (C-10), 158.3 (C-8), 145.9 (C-11), 140.9 (C-3), 137.5 (C-17), 130.6 (C-6), 130.5 (C-13), 130.0 (C-5), 128.9 (C-12), 127.6 (C-15), 127.4 (C-16), 126.7 (C-14), 125.7 (C-4), 118.1 (C-18), 113.7 (C-7), 77.1 (C-2), 55.4 (C-9), 27.8 (C-1), 14.4 (C-19) ppm.

HRMS (ESI) m/z: $[M+H]^+$ calcd for $C_{21}H_{22}NO_2^+$ 320.1645, found 320.1640.

IR (thin film): ṽ 3310 (br), 1601 (s), 1501 (s), 1370 (s), 1246 (s), 1120 (s), 1023 (s) cm^{-1}.

Specific rotation: $[\alpha]_D^{23}$ = −226.9 (*c* 0.5, CH_2Cl_2).

The **enantiomeric ratio** of **3ae** was determined by SFC analysis (CHIRALPACK IE (25 cm), column temperature 25 °C, solvent CO_2/MeOH (with 0.5% Et_3N) = 90/10, flow rate = 2.0 mL/min): t_R = 16.1 min (minor), t_R = 18.3 min (major).

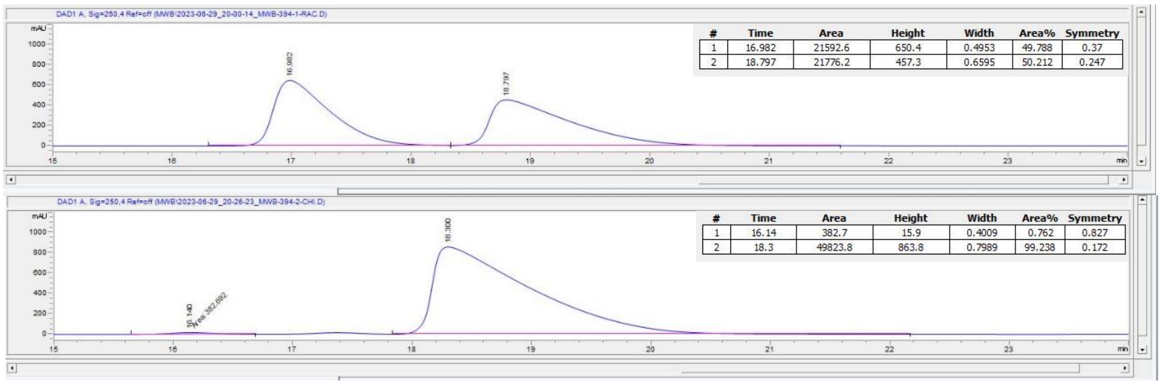

Characteristic signals for regioisomer *iso*-**3ae**

^{1}H NMR (500 MHz, $CDCl_3$, 298 K): 6.06 (q, *J* = 6.8 Hz, 1H), 1.47 (d, *J* = 6.8 Hz, 3H).

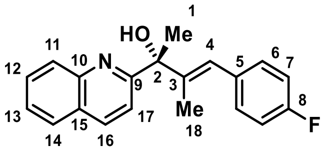

$C_{20}H_{18}FNO_2$
M = 307.37

(*R*,*E*)-4-(4-Fluorophenyl)-3-methyl-2-(quinolin-2-yl)but-3-en-2-ol (**3af**, **GP 6**) was prepared from 1-(quinolin-2-yl)ethan-1-ol (**1a**, 17.3 mg, 0.10 mmol, 100 mol%) and 1-fluoro-4-(prop-1-yn-1-yl)benzene (**2f**, 33.5 mg, 0.25 mmol, 250 mol%), using $[Ir(cod)_2]$BARF (6.36 mg, 5.0 μmol, 5.0 mol%), **L1** (3.11 mg, 5.0 μmol, 5.0 mol%) and 3,3-dimethylbutan-2-ol (32 μL, 25.5 mg, 0.25 mmol, 250 mol%) in toluene (0.2 mL, 0.5 M) at 110 °C for 48 h. Purification by flash column chromatography on silica gel (hexane/ethyl acetate from 100/1 to 19/1) afforded **3af** (22.7 mg, 74% yield, 97% *ee*, >25:1 r.r.) as a colorless solid. The regioisomer ratio of the process (17:1 r.r.) was determined from the ^{1}H NMR spectrum of crude material.

R_f = 0.4 (Hexane/EtOAc = 15/1).

M.P. 109–110 °C (Hexane/EtOAc).

^{1}H NMR (500 MHz, $CDCl_3$, 298 K): δ 8.16 (d, J = 8.6 Hz, 1H, H-16), 8.12 (d, J = 8.5 Hz, 1H, H-11), 7.85 (d, J = 8.1 Hz, 1H, H-14), 7.78–7.73 (m, 1H, H-12), 7.60–7.55 (m, 1H, H-13), 7.44 (d, J = 8.6 Hz, 1H, H-17), 7.29–7.25 (m, 2H, H-6), 7.06–7.00 (m, 2H, H-7), 6.82 (s, 1H, H-4), 6.37 (s, 1H, OH), 1.82 (s, 3H, H-1), 1.64 (s, 3H, H-18) ppm.

^{13}C NMR (125 MHz, $CDCl_3$, 298 K): δ 163.5 (C-9), 161.6 (d, $J_{C\text{–}F}$ = 243.8 Hz, C-8), 145.9 (C-10), 142.5 (C-3), 137.6 (C-16), 134.1 (d, $J_{C\text{–}F}$ = 2.5 Hz, C-5), 130.8 (d, $J_{C\text{–}F}$ = 7.5 Hz, C-6), 130.1 (C-12), 128.9 (C-11), 127.7 (C-14), 127.4 (C-15), 126.8 (C-13), 125.1 (C-4), 117.9 (C-17), 115.1 (d, $J_{C\text{–}F}$ = 21.3 Hz, C-7), 77.0 (C-2), 27.7 (C-1), 14.3 (C-18) ppm.

^{19}F NMR (471 MHz, $CDCl_3$, 298 K): δ –155.8—155.7 (m, 1F, ArF) ppm.

HRMS (ESI) m/z: $[M+H]^+$ calcd for $C_{20}H_{19}FNO^+$ 308.1444, found 308.1452.

IR (thin film): ṽ 3350 (br), 1598 (s), 1507 (s), 1366 (s), 1218 (s), 1099 (s) cm^{-1}.

Specific rotation: $[\alpha]_D^{23}$ = −227.15 (c 0.5, CH_2Cl_2).

The **enantiomeric ratio** of **3af** was determined by SFC analysis (CHIRALPACK IE (25 cm), column temperature 25 °C, solvent CO_2/MeOH (0.5% Et_3N) = 90/10, flow rate = 2.0 mL/min): t_R = 7.7 min (minor), t_R = 8.3 min (major).

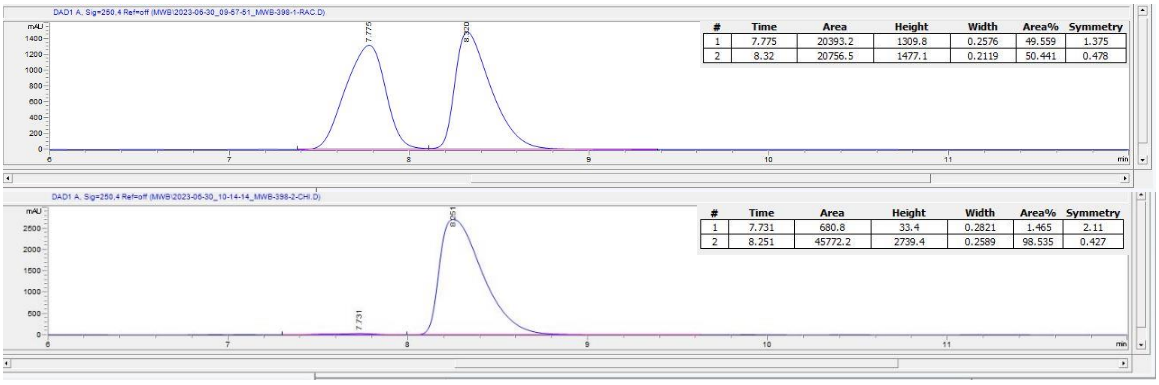

Characteristic signals for regioisomer *iso*-**3af**

^{1} H NMR (500 MHz, $CDCl_3$, 298 K): 6.07 (q, J = 6.8 Hz, 1H), 1.47 (d, J = 6.8 Hz, 3H).

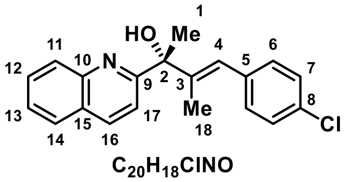

$C_{20}H_{18}ClNO$
M = 323.82

(*R*,*E*)-4-(4-Chlorophenyl)-3-methyl-2-(quinolin-2-yl)but-3-en-2-ol (**3ag**, **GP 6**) was prepared from 1-(quinolin-2-yl)ethan-1-ol (**1a**, 17.3 mg, 0.10 mmol, 100 mol%) and 1-chloro-4-(prop-1-yn-1-yl)benzene (**2g**, 37.7 mg, 0.25 mmol, 250 mol%), using [Ir(cod)$_2$]BARF (6.36 mg, 5.0 μmol, 5.0 mol%), **L1** (3.11 mg, 5.0 μmol, 5.0 mol%) and 3,3-dimethylbutan-2-ol (32 μL, 25.5 mg, 0.25 mmol, 250 mol%) in toluene (0.2 mL, 0.5 M) at 110 °C for 48 h. Purification by flash column chromatography on silica gel (hexane/ethyl acetate from 100/1 to 19/1) afforded **3ag** (23.3 mg, 72% yield, 98% ee, >25:1 r.r.) as a colorless solid. The regioisomer ratio of the process (15:1 r.r.) was determined from the ^{1}H NMR spectrum of crude material.

R*f* = 0.3 (Hexane/EtOAc = 15/1).

M.P. 112–113 °C (Hexane/EtOAc).

^{1}H NMR (500 MHz, $CDCl_3$, 298 K): δ 8.16 (d, *J* = 8.6 Hz, 1H, H-16), 8.12 (d, *J* = 8.5 Hz, 1H, H-11), 7.85 (d, *J* = 8.1 Hz, 1H, H-14), 7.78–7.74 (m, 1H, H-12), 7.60–7.55 (m, 1H, H-13), 7.44 (d, *J* = 8.6 Hz, 1H, H-17), 7.33–7.28 (m, 2H, H-7), 7.25–7.22 (m, 2H, H-6), 6.81 (s, 1H, H-4), 6.37 (s, 1H, OH), 1.82 (s, 3H, H-1), 1.64 (s, 3H, H-18) ppm.

^{13}C NMR (125 MHz, $CDCl_3$, 298 K): δ 163.4 (C-9), 145.9 (C-10), 143.4 (C-3), 137.6 (C-5), 136.5 (C-16), 132.4 (C-8), 130.6 (C-6), 130.1 (C-12), 128.9 (C-11), 128.4 (C-7), 127.7 (C-14), 127.4 (C-15), 126.8 (C-13), 125.0 (C-4), 117.9 (C-17), 77.0 (C-2), 27.7 (C-1), 14.4 (C-18) ppm.

HRMS (ESI) m/z: $[M+H]^+$ calcd for $C_{20}H_{19}{}^{35}ClNO^+$ 324.1149, found 324.1156.

IR (thin film): ṽ 3333 (br), 1598 (s), 1489 (s), 1384 (s), 1114 (s) cm^{-1}.

Specific rotation: $[\alpha]_D^{23}$ = −249.2 (*c* 0.5, CH_2Cl_2).

The **enantiomeric ratio** of **3ag** was determined by SFC analysis (CHIRALPACK IE (25 cm), column temperature 25 °C, solvent CO_2/MeOH (with 0.5% Et_3N) = 90/10, flow rate = 2.0 mL/min): t_R = 12.1 min (minor), t_R = 13.6 min (major).

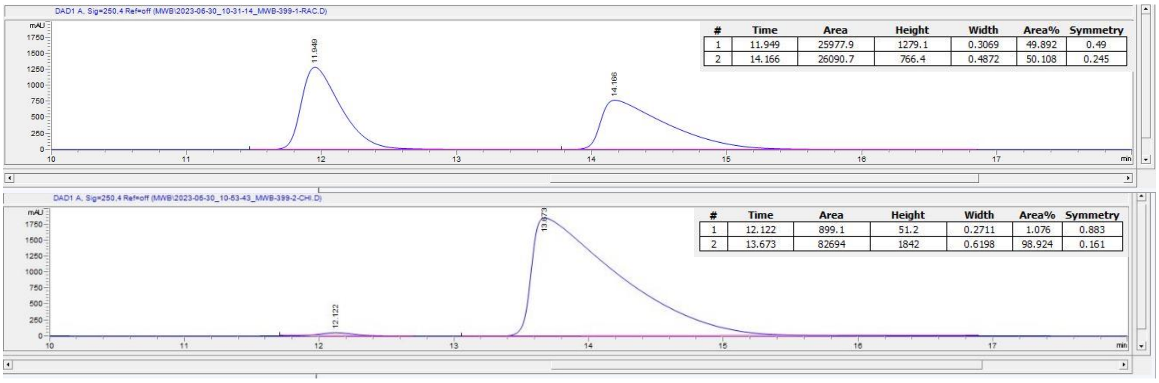

Crystal **3ag** was obtained through recrystallization in the solution of CH_2Cl_2 and methanol at room temperature. The absolute configuration of **3ag** was confirmed unambiguously by **X-ray diffraction analysis**, and other compounds were assigned by analogy. CCDC 2398065 contains the supplementary crystallographic data for this compound.

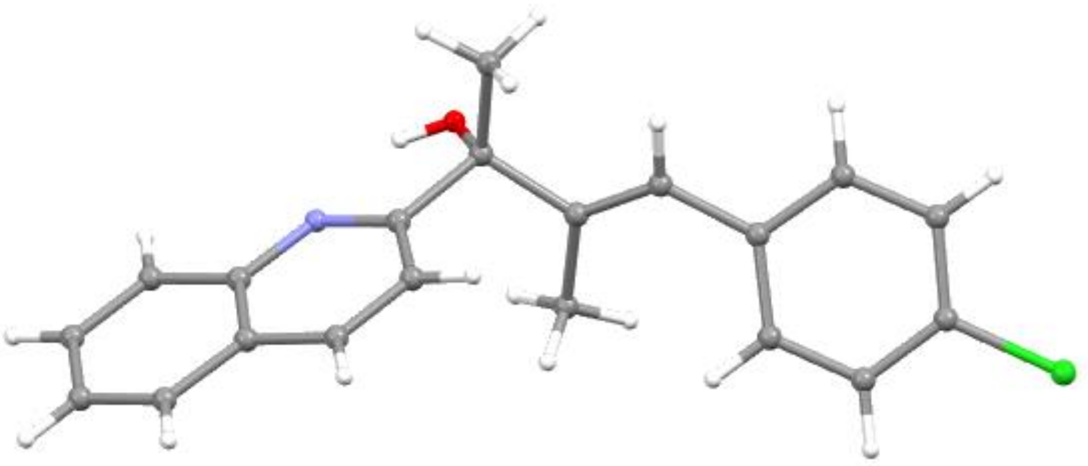

Figure S1. Molecular structure of **3ag**.

Characteristic signals for regioisomer *iso*-**3ag**

^{1} **H NMR** (500 MHz, $CDCl_3$, 298 K): 6.08 (q, J = 6.8 Hz, 1H), 1.47 (d, J = 6.8 Hz, 3H).

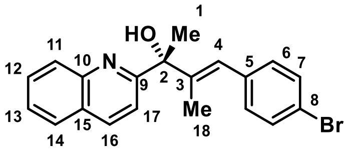

$C_{20}H_{18}BrNO$
M = 368.27

(*R*,*E*)-4-(4-Bromophenyl)-3-methyl-2-(quinolin-2-yl)but-3-en-2-ol (**3ah**, **GP 6**) was prepared from 1-(quinolin-2-yl)ethan-1-ol (**1a**, 17.3 mg, 0.10 mmol, 100 mol%) and 1-bromo-4-(prop-1-yn-1-yl)benzene (**2h**, 97.5 mg, 0.50 mmol, 500 mol%), using $[Ir(cod)_2]BARF$ (12.7 mg, 10 μmol, 10 mol%), **L1** (6.23 mg, 10 μmol, 10 mol%) and 3,3-dimethylbutan-2-ol (32 μL, 25.5 mg, 0.25 mmol, 250 mol%) in toluene (0.2 mL, 0.5 M) at 110 °C for 24 h. Purification by flash column chromatography on silica gel (hexane/ethyl acetate from 100/1 to 19/1) afforded **3ah** (28.7 mg, 78% yield, 98% ee, >25:1 r.r.) as a brown solid. The regioisomer ratio of the process (16:1 r.r.) was determined from the ^{1}H NMR spectrum of crude material.

R*f* = 0.2 (Hexane/EtOAc = 15/1).

M.**P**. 67–68 °C (Hexane/EtOAc).

^{1}H NMR (500 MHz, $CDCl_3$, 298 K): δ 8.16 (d, *J* = 8.6 Hz, 1H, H-16), 8.12 (d, *J* = 8.4 Hz, 1H, H-11), 7.85 (d, *J* = 8.1 Hz, 1H, H-14), 7.78–7.73 (m, 1H, H-12), 7.60–7.55 (m, 1H, H-13), 7.45 (d, *J* = 8.2 Hz, 2H, H-7), 7.43 (d, *J* = 8.6 Hz, 1H, H-17), 7.18 (d, *J* = 8.2 Hz, 2H, H-6), 6.78 (s, 1H, H-4), 6.37 (s, 1H, OH), 1.82 (s, 3H, H-1), 1.63 (s, 3H, H-18) ppm.

^{13}C NMR (125 MHz, $CDCl_3$, 298 K): δ 163.4 (C-9), 145.9 (C-10), 143.5 (C-3), 137.6 (C-5), 137.0 (C-16), 131.3 (C-7), 130.9 (C-6), 130.1 (C-12), 128.9 (C-11), 127.7 (C-14), 127.4 (C-15), 126.8 (C-13), 125.0 (C-4), 120.5 (C-8), 117.9 (C-17), 77.0 (C-2), 27.7 (C-1), 14.4 (C-18) ppm.

HRMS (ESI) m/z: $[M–H_2O+H]^+$ calcd for $C_{20}H_{17}{}^{79}BrN^+$ 350.0539, found 350.0537.

HRMS (ESI) m/z: $[M+H]^+$ calcd for $C_{20}H_{19}{}^{81}BrNO^+$ 370.0624, found 370.0616.

IR (thin film): ṽ 3332 (br), 1597 (s), 1487 (s), 1377 (s), 1113 (s) cm^{-1}.

Specific rotation: $[\alpha]_D^{23}$ = −239.89 (*c* 0.5, CH_2Cl_2).

The **enantiomeric ratio** of **3ah** was determined by SFC analysis (CHIRALPACK IE (25 cm), column temperature 25 °C, solvent CO_2/MeOH (with 0.5% Et_3N) = 90/10, flow rate = 2.0 mL/min): t_R = 16.2 min (minor), t_R = 19.6 min (major).

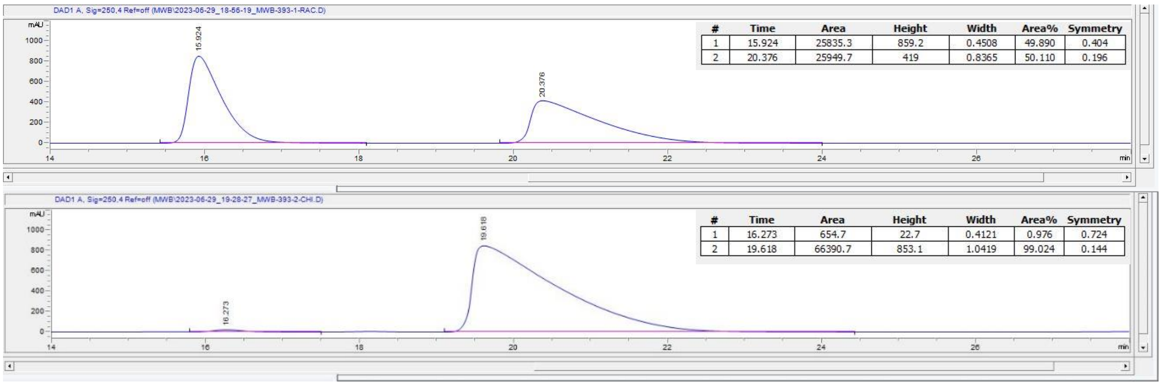

Characteristic signals for regioisomer *iso*-**3ah**

^{1} H NMR (500 MHz, $CDCl_3$, 298 K): 6.07 (q, *J* = 6.8 Hz, 1H), 1.46 (d, *J* = 6.8 Hz, 3H).

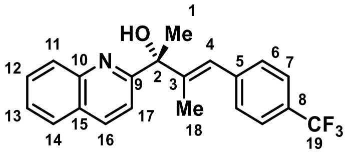

$C_{21}H_{18}F_3NO$
M = 357.38

(*R*,*E*)-3-Methyl-2-(quinolin-2-yl)-4-(4-(trifluoromethyl)phenyl)but-3-en-2-ol (**3ai**, **GP 6**) was prepared from 1-(quinolin-2-yl)ethan-1-ol (**1a**, 17.3 mg, 0.10 mmol, 100 mol%) and 1-

(prop-1-yn-1-yl)-4-(trifluoromethyl)benzene (**2i**, 45.0 mg, 0.25 mmol, 250 mol%), using $[Ir(cod)_2]$BARF (6.36 mg, 5.0 μmol, 5.0 mol%), **L1** (3.11 mg, 5.0 μmol, 5.0 mol%) and 3,3-dimethylbutan-2-ol (32 μL, 25.5 mg, 0.25 mmol, 250 mol%) in toluene (0.2 mL, 0.5 M) at 110 °C for 48 h. Purification by flash column chromatography on silica gel (hexane/ethyl acetate from 100/1 to 19/1) afforded **3ai** (26.0 mg, 73% yield, 97% ee, >25:1 r.r.) as a yellow solid. The regioisomer ratio of the process (15:1 r.r.) was determined from the ^{1}H NMR spectrum of crude material.

R*f* = 0.2 (Hexane/EtOAc = 15/1).

M.P. 75–76 °C (Hexane/EtOAc).

^{1}H NMR (500 MHz, $CDCl_3$, 298 K): δ 8.18 (d, *J* = 8.6 Hz, 1H, H-16), 8.13 (d, *J* = 8.4 Hz, 1H, H-11), 7.85 (d, *J* = 8.2 Hz, 1H, H-14), 7.79–7.74 (m, 1H, H-12), 7.61–7.55 (m, 3H, H-7 and H-13), 7.45 (d, *J* = 8.6 Hz, 1H, H-17), 7.41 (d, *J* = 8.1 Hz, 2H, H-6), 6.89 (s, 1H, H-4), 6.40 (s, 1H, OH), 1.84 (s, 3H, H-1), 1.67 (s, 3H, H-18) ppm.

^{13}C NMR (125 MHz, $CDCl_3$, 298 K): δ 163.2 (C-9), 145.9 (C-10), 144.9 (C-3), 141.8 (C-5), 137.7 (C-16), 130.2 (C-12), 129.5 (C-6), 128.9 (C-11), 128.6 ($J_{C–F}$ = 32.5 Hz, C-8), 127.7 (C-14), 127.4 (C-15), 126.9 (C-13), 125.2 ($J_{C–F}$ = 4.2 Hz, C-7), 124.9 (C-4), 124.4 ($J_{C–F}$ = 271.3 Hz, C-19), 117.8 (C-17), 76.9 (C-2), 27.6 (C-1), 14.5 (C-18) ppm.

^{19}F NMR (471 MHz, $CDCl_3$, 298 K): δ –62.4 (s, 3F, $ArCF_3$) ppm.

HRMS (ESI) m/z: $[M–H_2O+H]^+$ calcd for $C_{21}H_{17}F_3N^+$ 340.1308, found 340.1311.

IR (thin film): ṽ 3365 (br), 1600 (s), 1505 (s), 1321 (s), 1108 (s), 1065 (s) cm^{-1}.

Specific rotation: $[\alpha]_D^{24}$ = −125.48 (*c* 0.5, CH_2Cl_2).

The **enantiomeric ratio** of **3ai** was determined by SFC analysis (CHIRALPACK IE (25 cm), column temperature 25 °C, solvent CO_2/MeOH (with 0.5% Et_3N) = 90/10, flow rate = 2.0 mL/min): t_R = 4.8 min (minor), t_R = 5.2 min (major).

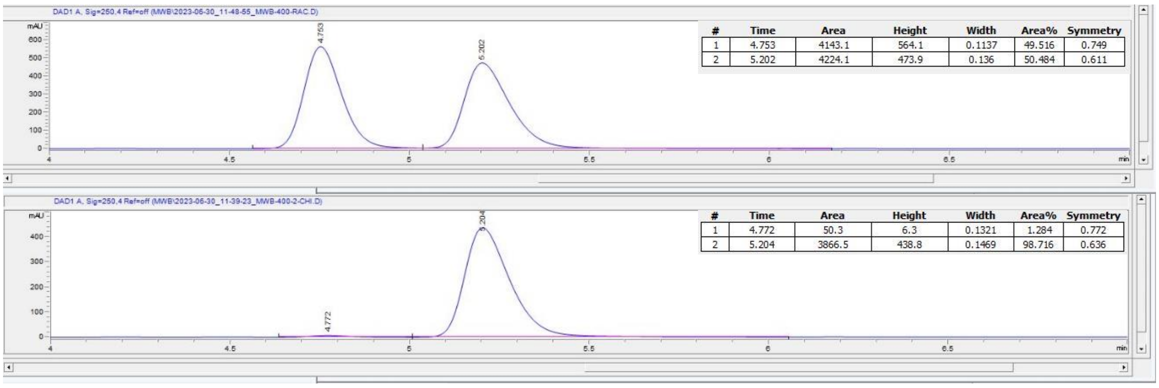

#	Time	Area	Height	Width	Area%	Symmetry
1	4.753	4143.1	564.1	0.1137	49.516	0.749
2	5.202	4224.1	473.9	0.136	50.484	0.611

#	Time	Area	Height	Width	Area%	Symmetry
1	4.772	50.3	6.3	0.1321	1.284	0.772
2	5.204	3866.5	438.8	0.1469	98.716	0.636

Characteristic signals for regioisomer *iso*-**3ai**

^{1} H NMR (500 MHz, $CDCl_3$, 298 K): 6.08 (q, *J* = 6.9 Hz, 1H), 1.46 (d, *J* = 6.8 Hz, 3H).

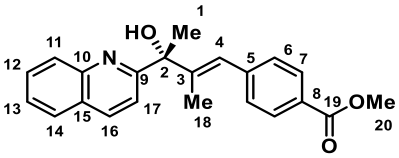

$C_{22}H_{21}NO_3$
M = 347.41

Methyl (*R*,*E*)-4-(3-hydroxy-2-methyl-3-(quinolin-2-yl)but-1-en-1-yl)benzoate (**3aj**, **GP 6**) was prepared from 1-(quinolin-2-yl)ethan-1-ol (**1a**, 17.3 mg, 0.10 mmol, 100 mol%) and methyl 4-(prop-1-yn-1-yl)benzoate (**2j**, 43.6 mg, 0.25 mmol, 250 mol%), using $[Ir(cod)_2]BARF$ (6.36 mg, 5.0 μmol, 5.0 mol%), **L1** (3.11 mg, 5.0 μmol, 5.0 mol%) and 3,3-dimethylbutan-2-ol (32 μL, 25.5 mg, 0.25 mmol, 250 mol%) in toluene (0.2 mL, 0.5 M) at 110 °C for 48 h. Purification by flash column chromatography on silica gel (hexane/ethyl acetate from 100/1 to 19/1) afforded **3aj** (23.5 mg, 68% yield, 98% ee, >25:1 r.r.) as a colorless solid. The regioisomer ratio of the process (10:1 r.r.) was determined from the ^{1}H NMR spectrum of crude material.

R*f* = 0.3 (Hexane/EtOAc = 9/1).

M.**P**. 122–123 °C (Hexane/EtOAc).

^{1}H NMR (400 MHz, $CDCl_3$, 298 K): δ 8.17 (d, *J* = 8.6 Hz, 1H, H-16), 8.12 (d, *J* = 8.5 Hz, 1H, H-11), 8.00 (d, *J* = 8.2 Hz, 2H, H-7), 7.85 (d, *J* = 8.2 Hz, 1H, H-14), 7.79–7.73 (m, 1H, H-12), 7.60–7.55 (m, 1H, H-13), 7.45 (d, *J* = 8.6 Hz, 1H, H-17), 7.37 (d, *J* = 8.2 Hz, 2H, H-6), 6.88 (s, 1H, H-4), 6.38 (s, 1H, OH), 3.91 (s, 3H, H-20), 1.84 (s, 3H, H-1), 1.68 (s, 3H, H-18) ppm.

^{13}C NMR (100 MHz, $CDCl_3$, 298 K): δ 167.1 (C-19), 163.2 (C-9), 145.9 (C-10), 144.8 (C-3), 143.0 (C-5), 137.6 (C-16), 130.1 (C-12), 129.5 (C-7), 129.2 (C-6), 128.9 (C-11), 128.2 (C-8), 127.7 (C-14), 127.4 (C-15), 126.9 (C-13), 125.4 (C-4), 117.9 (C-17), 77.0 (C-2), 52.2 (C-20), 27.6 (C-1), 14.6 (C-18) ppm.

HRMS (ESI) m/z: $[M+H]^+$ calcd for $C_{22}H_{22}NO_3^+$ 348.1594, found 348.1605.

IR (thin film): ṽ 3375 (br), 1705 (s), 1600 (s), 1504 (s), 1431 (s), 1276 (s), 1108 (s), 1079 (s) cm^{-1}.

Specific rotation: $[\alpha]_D^{23}$ = −276.5 (*c* 0.5, CH_2Cl_2).

The **enantiomeric ratio** of **3aj** was determined by SFC analysis (CHIRALPACK IE (25 cm), column temperature 25 °C, solvent CO_2/MeOH (with 0.5% Et_3N) = 60/40, flow rate = 2.0 mL/min): t_R = 7.4 min (minor), t_R = 10.7 min (major).

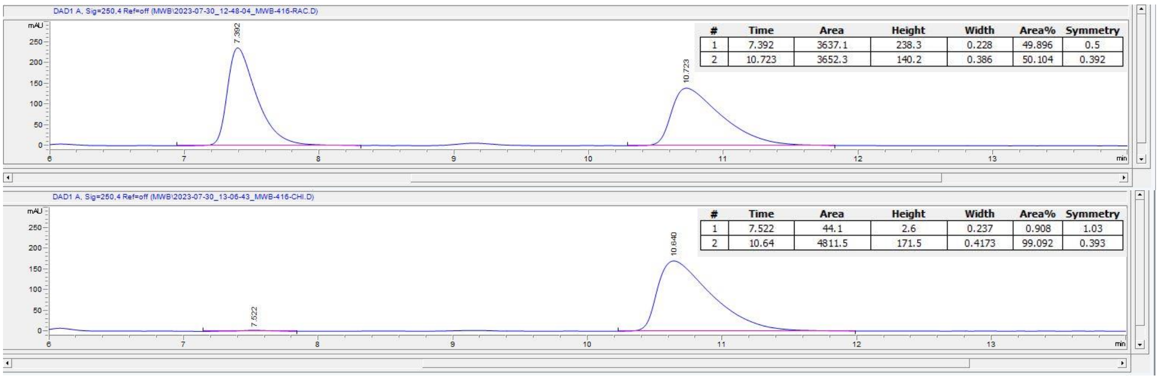

Characteristic signals for regioisomer *iso*-**3aj**

[1] H NMR (400 MHz, $CDCl_3$, 298 K): 6.09 (q, *J* = 6.8 Hz, 1H), 1.46 (d, *J* = 6.8 Hz, 3H).

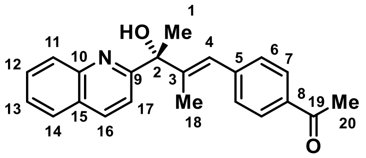

$C_{22}H_{21}NO_2$
M = 331.42

(*R*,*E*)-1-(4-(3-Hydroxy-2-methyl-3-(quinolin-2-yl)but-1-en-1-yl)phenyl)ethan-1-one (**3ak**, **GP 6**) was prepared from 1-(quinolin-2-yl)ethan-1-ol (**1a**, 17.3 mg, 0.10 mmol, 100 mol%) and 1-(4-(prop-1-yn-1-yl)phenyl)ethan-1-one (**2k**, 39.6 mg, 0.25 mmol, 250 mol%), using $[Ir(cod)_2]$BARF (6.36 mg, 5.0 µmol, 5.0 mol%), **L1** (3.11 mg, 5.0 µmol, 5.0 mol%) and 3,3-dimethylbutan-2-ol (32 µL, 25.5 mg, 0.25 mmol, 250 mol%) in toluene (0.2 mL, 0.5 M) at 110 °C for 48 h. Purification by flash column chromatography on silica gel (hexane/acetone from 100/1 to 19/1) afforded **3ak** (20.1 mg, 61% yield, 96% ee, >25:1 r.r.) as a colorless solid. The regioisomer ratio of the process (8:1 r.r.) was determined from the ^{1}H NMR spectrum of crude material.

R_f = 0.3 (Hexane/acetone = 5/1).

M.P. 98–99 °C (Hexane/Acetone).

^{1}H NMR (400 MHz, $CDCl_3$, 298 K): δ 8.18 (d, *J* = 8.6 Hz, 1H, H-16), 8.12 (d, *J* = 8.4 Hz, 1H, H-11), 7.93 (d, *J* = 8.4 Hz, 2H, H-7), 7.85 (d, *J* = 8.0 Hz, 1H, H-14), 7.79–7.73 (m, 1H, H-12), 7.60–7.55 (m, 1H, H-13), 7.45 (d, *J* = 8.6 Hz, 1H, H-17), 7.40 (d, *J* = 8.4 Hz, 2H, H-6), 6.89 (s, 1H, H-4), 6.39 (s, 1H, OH), 2.60 (s, 3H, H-20), 1.84 (s, 3H, H-1), 1.69 (s, 3H, H-18) ppm.

^{13}C NMR (100 MHz, $CDCl_3$, 298 K): δ 197.8 (C-19), 163.2 (C-9), 145.9 (C-10), 145.0 (C-3), 143.2 (C-5), 137.7 (C-16), 135.3 (C-8), 130.1 (C-12), 129.4 (C-6), 128.9 (C-11), 128.4 (C-7), 127.7 (C-14), 127.4 (C-15), 126.9 (C-13), 125.3 (C-4), 117.9 (C-17), 77.0 (C-2), 27.6 (C-1), 26.7 (C-20), 14.6 (C-18) ppm.

HRMS (ESI) m/z: $[M+H]^+$ calcd for $C_{22}H_{22}NO_2^+$ 332.1645, found 332.1650.

IR (thin film): ṽ 3338 (br), 1675 (s), 1599 (s), 1504 (s), 1366 (s), 1270 (s), 1108 (s), 1076 (s) cm^{-1}.

Specific rotation: $[\alpha]_D^{22}$ = −274.8 (*c* 0.5, CH_2Cl_2).

The **enantiomeric ratio** of **3ak** was determined by SFC analysis (CHIRALPACK IE (25 cm), column temperature 25 °C, solvent CO_2/MeOH (with 0.5% Et_3N) = 60/40, flow rate = 2.0 mL/min): t_R = 7.4 min (minor), t_R = 8.8 min (major).

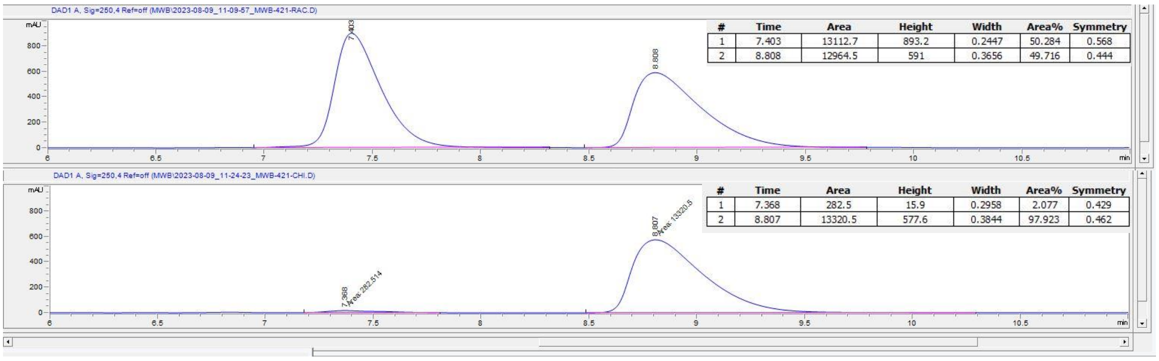

Characteristic signal for regioisomer *iso*-**3ak**

^{1} H NMR (500 MHz, $CDCl_3$, 298 K): 1.46 (d, *J* = 6.5 Hz, 3H).

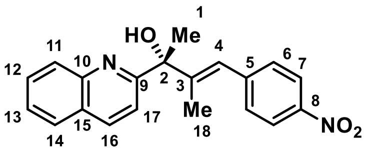

$C_{20}H_{18}N_2O_3$
M = 334.38

(*R*,*E*)-3-Methyl-4-(4-nitrophenyl)-2-(quinolin-2-yl)but-3-en-2-ol (**3al**, **GP 6**) was prepared from 1-(quinolin-2-yl)ethan-1-ol (**1a**, 17.3 mg, 0.10 mmol, 100 mol%) and 1-nitro-4-(prop-1-yn-1-yl)benzene (**2l**, 80.6 mg, 0.50 mmol, 500 mol%), using [Ir(cod)$_2$]BARF (12.7 mg, 10 μmol, 10 mol%), **L1** (6.23 mg, 10 μmol, 10 mol%) and 3,3-dimethylbutan-2-ol (32 μL, 25.5 mg, 0.25 mmol, 250 mol%) in toluene (0.2 mL, 0.5 M) at 110 °C for 48 h. Purification by flash column chromatography on silica gel (hexane/acetone from 100/1 to 19/1) afforded **3al** (16.2 mg, 49% yield, 97% ee, >25:1 r.r.) as a yellow solid. The regioisomer ratio of the process (6:1 r.r.) was determined from the ^{1}H NMR spectrum of crude material.

R*f* = 0.1 (Hexane/ethyl acetate = 15/1).

M.P. 118–119 °C (Hexane/Acetone).

^{1}H NMR (400 MHz, $CDCl_3$, 298 K): δ 8.22–8.17 (m, 3H, H-7 and H-16), 8.13 (d, *J* = 8.5 Hz, 1H, H-11), 7.86 (d, *J* = 8.1 Hz, 1H, H-14), 7.80–7.74 (m, 1H, H-12), 7.62–7.56 (m, 1H, H-13), 7.47–7.42 (m, 3H, H-6 and H-17), 6.90 (s, 1H, H-4), 6.40 (s, 1H, OH), 1.85 (s, 3H, H-1), 1.70 (s, 3H, H-18) ppm.

^{13}C NMR (100 MHz, $CDCl_3$, 298 K): δ 162.8 (C-9), 146.8 (C-3), 146.3 (C-5), 145.9 (C-10), 145.1 (C-8), 137.8 (C-16), 130.3 (C-12), 129.9 (C-6), 128.9 (C-11), 127.7 (C-14), 127.4 (C-15), 127.0 (C-13), 124.3 (C-4), 123.6 (C-7), 117.7 (C-17), 76.9 (C-2), 27.5 (C-1), 14.7 (C-18) ppm.

HRMS (ESI) m/z: $[M+H]^+$ calcd for $C_{20}H_{19}N_2O_3^+$ 335.1389, found 335.1390.

IR (thin film): ṽ 3325 (br), 1593 (s), 1514 (s), 1339 (s), 1109 (s), 1079 (s) cm^{-1}.

Specific rotation: $[\alpha]_D^{24}$ = −262.6 (*c* 0.5, CH_2Cl_2).

The **enantiomeric ratio** of **3al** was determined by SFC analysis (CHIRALPACK IE (25 cm), column temperature 25 °C, solvent CO_2/MeOH (with 0.5% Et_3N) = 75/25, flow rate = 2.0 mL/min): t_R = 10.3 min (minor), t_R = 11.9 min (major).

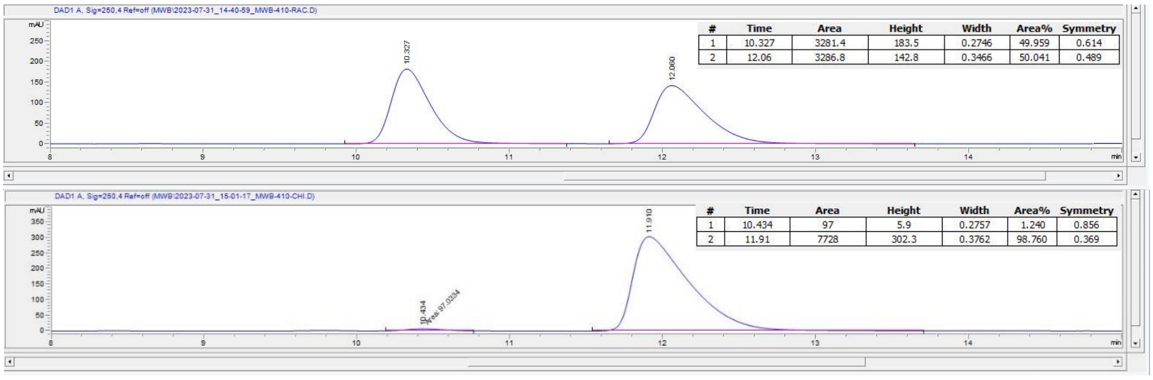

Characteristic signal for regioisomer *iso*-**3al**

^{1} H NMR (500 MHz, $CDCl_3$, 298 K): 1.48 (d, *J* = 6.8 Hz, 3H).

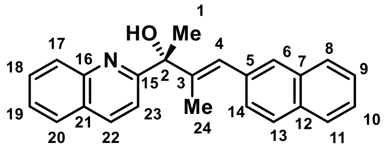

$C_{24}H_{21}NO$
M = 339.44

(*R*,*E*)-3-Methyl-4-(naphthalen-2-yl)-2-(quinolin-2-yl)but-3-en-2-ol (**3am**, **GP 6**) was prepared from 1-(quinolin-2-yl)ethan-1-ol (**1a**, 17.3 mg, 0.10 mmol, 100 mol%) and 2-(prop-1-yn-1-yl)naphthalene (**2m**, 41.6 mg, 0.25 mmol, 250 mol%), using $[Ir(cod)_2]BARF$ (6.36 mg, 5.0 µmol, 5.0 mol%), **L1** (3.11 mg, 5.0 µmol, 5.0 mol%) and 3,3-dimethylbutan-2-ol (32 µL, 25.5 mg, 0.25 mmol, 250 mol%) in toluene (0.2 mL, 0.5 M) at 110 °C for 24 h. Purification by flash column chromatography on silica gel (hexane/acetone from 100/1 to 19/1) afforded **3am** (22.1 mg, 65% yield, >99% ee, >25:1 r.r.) as a yellow solid. The regioisomer ratio of the process (7:1 r.r.) was determined from the ^{1}H NMR spectrum of crude material.

R$_f$ = 0.2 (Hexane/ethyl acetate = 15/1).

M.P. 107–108 °C (Hexane/Acetone).

^{1}H NMR (500 MHz, $CDCl_3$, 298 K): δ 8.18 (d, *J* = 8.6 Hz, 1H, H-22), 8.14 (d, *J* = 8.5 Hz, 1H, H-17), 7.86 (d, *J* = 8.2 Hz, 1H, H-20), 7.83–7.79 (m, 3H, ArH), 7.79–7.74 (m, 2H, ArH and H-18), 7.61–7.55 (m, 1H, H-19), 7.51 (d, *J* = 8.6 Hz, 1H, H-23), 7.49–7.42 (m, 3H, ArH), 7.02 (s, 1H, H-4), 6.40 (s, 1H, OH), 1.88 (s, 3H, H-1), 1.74 (s, 3H, H-24) ppm.

^{13}C NMR (125 MHz, $CDCl_3$, 298 K): δ 163.7 (C-15), 145.9 (C-16), 143.0 (C-3), 137.6 (C-22), 135.7 (C-5), 133.4 (C-7 or C-12), 132.3 (C-7 or C-12), 130.1 (C-18), 129.0 (C-17), 128.0 (Ar), 128.0 (Ar), 127.7 (Ar), 127.7 (C-20), 127.4 (C-21), 126.8 (C-19), 126.2 (Ar), 125.9 (C-4), 118.0 (C-23), 77.1 (C-2), 27.8 (C-1), 14.5 (C-24) ppm.

HRMS (ESI) m/z: $[M+H]^+$ calcd for $C_{24}H_{22}NO^+$ 340.1695, found 340.1687.

IR (thin film): ṽ 3330 (br), 1598 (s), 1503 (s), 1380 (s), 1110 (s), 1076 (s) cm^{-1}.

Specific rotation: $[\alpha]_D^{24}$ = −205.5 (*c* 0.5, CH_2Cl_2).

The **enantiomeric ratio** of **3am** was determined by SFC analysis (CHIRALPACK IE (25 cm), column temperature 25 °C, solvent CO_2/MeOH (with 0.5% Et_3N) = 90/10, flow rate = 2.0 mL/min): t_R = 26.7 min (minor), t_R = 28.3 min (major).

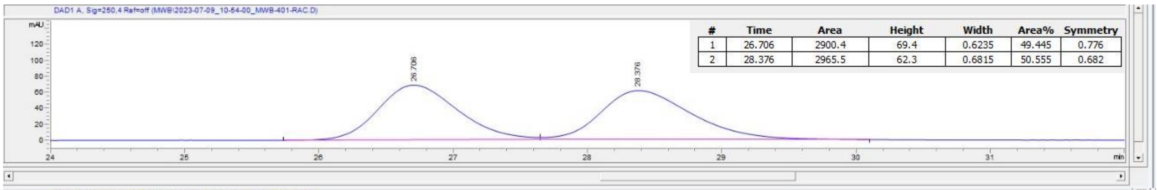

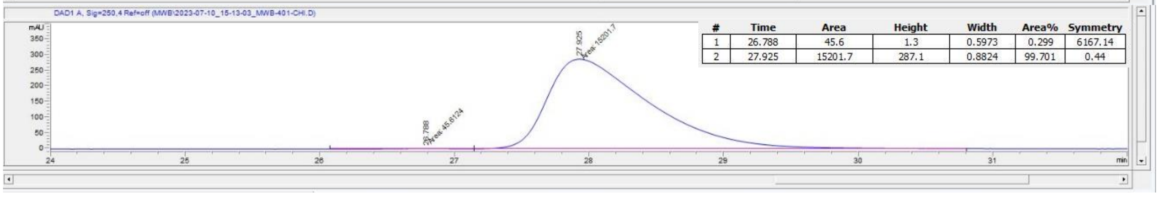

Characteristic signals for regioisomer *iso*-**3am**

[1] **H NMR** (500 MHz, $CDCl_3$, 298 K): 6.15 (q, *J* = 6.8 Hz, 1H), 1.50 (d, *J* = 6.8 Hz, 3H).

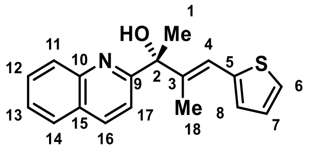

$C_{18}H_{17}NOS$
M = 295.40

(*R*,*E*)-3-Methyl-2-(quinolin-2-yl)-4-(thiophen-2-yl)but-3-en-2-ol (**3an**, **GP 6**) was prepared from 1-(quinolin-2-yl)ethan-1-ol (**1a**, 17.3 mg, 0.10 mmol, 100 mol%) and 2-(prop-1-yn-1-yl)thiophene (**2n**, 61.1 mg, 0.50 mmol, 500 mol%), using $[Ir(cod)_2]$BARF (12.7 mg, 10 μmol, 10 mol%), **L1** (6.23 mg, 10 μmol, 10 mol%) and 3,3-dimethylbutan-2-ol (32 μL, 25.5 mg, 0.25 mmol, 250 mol%) in toluene (0.2 mL, 0.5 M) at 110 °C for 48 h. Purification by flash column chromatography on silica gel (hexane/ethyl acetate from 100/1 to 19/1) afforded **3an** (18.2 mg, 62% yield, 98% ee, >25:1 r.r.) as a brown solid. The regioisomer ratio of the process (7:1 r.r.) was determined from the ^{1}H NMR spectrum of crude material.

R*f* = 0.3 (Hexane/EtOAc = 15/1).

M.P. 71–72 °C (Hexane/EtOAc).

^{1}H NMR (500 MHz, $CDCl_3$, 298 K): δ 8.13 (d, *J* = 8.6 Hz, 1H, H-16), 8.12 (d, *J* = 8.5 Hz, 1H, H-11), 7.84 (d, *J* = 8.1 Hz, 1H, H-14), 7.78–7.73 (m, 1H, H-12), 7.59–7.54 (m, 1H, H-13), 7.39 (d, *J* = 8.6 Hz, 1H, H-17), 7.28 (d, *J* = 5.1 Hz, 1H, H-6), 7.10–7.08 (m, 1H, H-8), 7.07–7.03 (m, 1H, H-7), 7.02 (s, 1H, H-4), 6.37 (s, 1H, OH), 1.82 (s, 3H, H-1), 1.79 (s, 3H, H-18) ppm.

^{13}C NMR (125 MHz, $CDCl_3$, 298 K): δ 163.4 (C-9), 145.9 (C-10), 141.2 (C-5), 140.6 (C-3), 137.6 (C-16), 130.1 (C-12), 129.0 (C-11), 127.7 (C-8), 127.7 (C-14), 127.4 (C-15), 127.0 (C-13), 126.8 (C-7), 125.3 (C-6), 119.4 (C-4), 118.1 (C-17), 77.0 (C-2), 27.7 (C-1), 15.4 (C-18) ppm.

HRMS (ESI) m/z: $[M+H]^+$ calcd for $C_{18}H_{18}NOS^+$ 296.1104, found 296.1099.

IR (thin film): ṽ 3336 (br), 1600 (s), 1505 (s), 1367 (s), 1121 (s), 1072 (s) cm^{-1}.

Specific rotation: $[\alpha]_D^{25}$ = −253.9 (*c* 0.5, CH_2Cl_2).

The **enantiomeric ratio** of **3an** was determined by SFC analysis (CHIRALPACK IE (25 cm), column temperature 25 °C, solvent CO_2/MeOH (with 0.5% Et_3N) = 90/10, flow rate = 2.0 mL/min): t_R = 15.2 min (minor), t_R = 16.4 min (major).

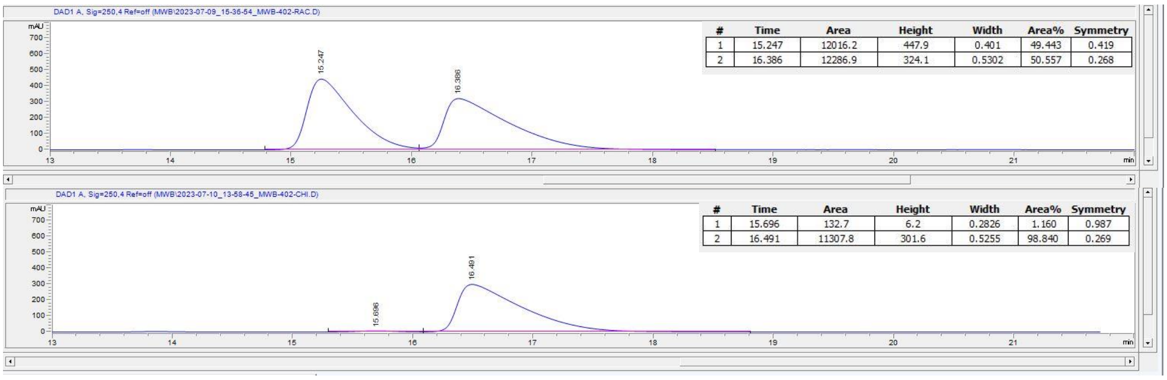

Characteristic signals for regioisomer *iso*-**3an**

^{1} H NMR (500 MHz, $CDCl_3$, 298 K): 6.24 (q, *J* = 6.8 Hz, 1H), 1.70 (d, *J* = 6.8 Hz, 3H).

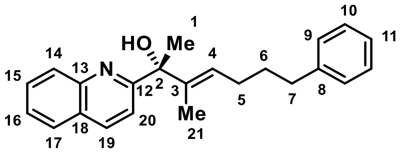

$C_{23}H_{25}NO$
M = 331.46

(*R*,*E*)-3-Methyl-7-phenyl-2-(quinolin-2-yl)hept-3-en-2-ol (**3ao**, **GP 6**) was prepared from 1-(quinolin-2-yl)ethan-1-ol (**1a**, 17.3 mg, 0.10 mmol, 100 mol%) and hex-4-yn-1-ylbenzene (**2o**, 43 μL, 39.6 mg, 0.25 mmol, 250 mol%), using [Ir(cod)$_2$]BARF (12.7 mg, 10 μmol, 10 mol%), **L1** (6.23 mg, 10 μmol, 10 mol%) and *i*-PrOH (19.1 μL, 15.0 mg, 0.25 mmol, 250 mol%) in toluene (0.2 mL, 0.5 M) at 110 °C for 24 h. Purification by flash column chromatography on silica gel (hexane/ethyl acetate from 100/1 to 19/1) afforded **3ao** (13.7 mg, 41% yield, >99% ee, >25:1 r.r.) as a brown oil. The regioisomer ratio of the process (13:1 r.r.) was determined from the ^{1}H NMR spectrum of crude material.

R_f = 0.2 (Hexane/EtOAc = 19/1).

^{1}H NMR (500 MHz, $CDCl_3$, 298 K): δ 8.12 (d, *J* = 8.6 Hz, 1H, H-19), 8.11 (d, *J* = 8.0 Hz, 1H, H-14), 7.83 (d, *J* = 8.1 Hz, 1H, H-17), 7.77–7.71 (m, 1H, H-15), 7.58–7.53 (m, 1H, H-16), 7.34

(d, J = 8.6 Hz, 1H, H-20), 7.32–7.27 (m, 2H, H-10), 7.23–7.17 (m, 3H, H-9 and H-11), 6.25 (s, 1H, OH), 5.80 (t, J = 7.1 Hz, 1H, H-4), 2.70 (t, J = 7.8 Hz, 2H, H-7), 2.21–2.11 (m, 2H, H-5), 1.84–1.74 (m, 2H, H-6), 1.69 (s, 3H, H-1), 1.42 (s, 3H, H-21) ppm.

^{13}C NMR (125 MHz, $CDCl_3$, 298 K): δ 164.2 (C-12), 145.8 (C-13), 142.6 (C-3), 139.6 (C-8), 137.3 (C-19), 129.9 (C-15), 128.9 (C-14), 128.6 (C-9), 128.4 (C-10), 127.6 (C-17), 127.3 (C-18), 126.6 (C-16), 126.2 (C-11), 125.8 (C-4), 118.0 (C-20), 76.6 (C-2), 35.8 (C-7), 31.4 (C-6), 27.9 (C-5), 27.7 (C-1), 12.7 (C-21) ppm.

HRMS (ESI) m/z: $[M+H]^+$ calcd for $C_{23}H_{26}NO^+$ 332.2008, found 332.2009.

IR (thin film): ṽ 3366 (br), 1696 (s), 1600 (s), 1505 (s), 1367 (s), 1124 (s), 1078 (s) cm^{-1}.

Specific rotation: $[\alpha]_D^{25}$ = −214.1 (c 0.5, CH_2Cl_2).

The **enantiomeric ratio** of **3ao** was determined by SFC analysis (CHIRALPACK SB (25 cm), column temperature 25 °C, solvent CO_2/MeOH (with 0.5% Et_3N) = 98/2, flow rate = 2.0 mL/min): t_R = 35.8 min (minor), t_R = 38.4 min (major).

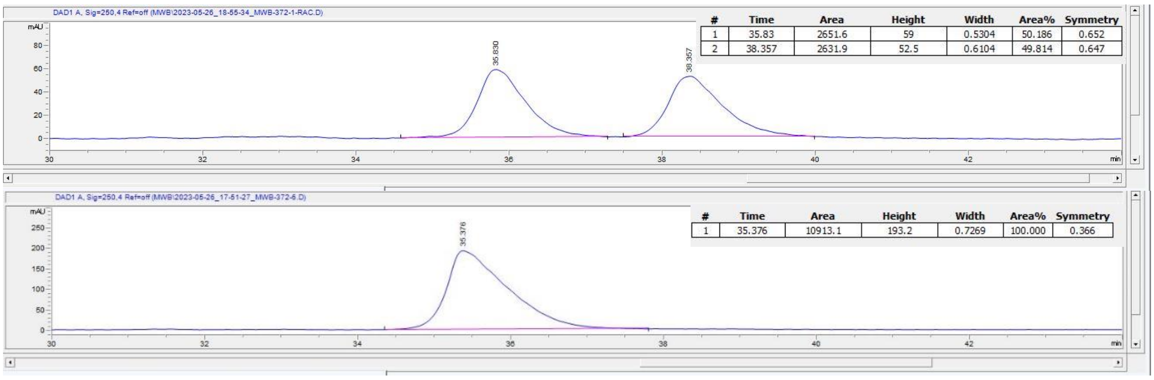

Characteristic signals for regioisomer *iso*-**3ao**

^{1}H NMR (500 MHz, $CDCl_3$, 298 K): 5.05 (q, J = 6.6 Hz, 1H), 1.59 (d, J = 6.6 Hz, 3H).

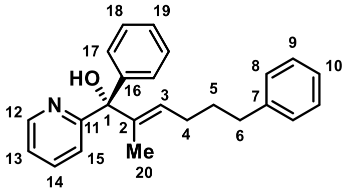

$C_{24}H_{25}NO$
M = 343.47

(*S*,*E*)-2-Methyl-1,6-diphenyl-1-(pyridin-2-yl)hex-2-en-1-ol (**3bo**, **GP 6**) was prepared from phenyl(pyridin-2-yl)methanol (**1b**, 18.5 mg, 0.10 mmol, 100 mol%) and hex-4-yn-1-ylbenzene (**2o**, 129 µL, 119 mg, 0.75 mmol, 750 mol%), using [Ir(cod)$_2$]BARF (12.7 mg, 10 µmol, 10

mol%), **L1** (6.23 mg, 10 μmol, 10 mol%) and *i*-PrOH (19.1 μL, 15.0 mg, 0.25 mmol, 250 mol%) in toluene (0.2 mL) at 110 °C for 48 h. Purification by flash column chromatography on silica gel (hexane/ethyl acetate from 100/1 to 19/1) afforded **3bo** (21.9 mg, 64% yield, 99% ee, >25:1 r.r.) as a colorless oil. The regioisomer ratio of the process (8:1 r.r.) was determined from the ^{1}H NMR spectrum of crude material.

$\mathbf{R}_{\boldsymbol{f}}$ = 0.2 (Hexane/EtOAc = 15/1).

^{1}H NMR (500 MHz, $CDCl_3$, 298 K): δ 8.57 (d, *J* = 4.9 Hz, 1H, H-12), 7.65–7.60 (m, 1H, H-14), 7.45–7.41 (m, 2H, H-17), 7.35–7.30 (m, 2H, H-18), 7.30–7.23 (m, 3H, H-9 and H-19 or H-10), 7.22–7.12 (m, 5H, H-8, H-10 or H-19, H-13 and H-15), 5.98 (s, 1H, OH), 4.93 (t, *J* = 7.1 Hz, 1H, H-3), 2.59 (t, *J* = 5.2 Hz, 2H, H-6), 2.17–2.04 (m, 2H, H-4), 1.68 (s, 3H, H-20), 1.67–1.60 (m, 2H, H-5) ppm.

^{13}C NMR (125 MHz, $CDCl_3$, 298 K): δ 162.3 (C-5), 147.9 (C-12), 144.6 (C-16), 142.5 (C-7), 140.3 (C-2), 136.3 (C-14), 129.9 (C-3), 128.5 (Ar), 128.4 (Ar), 128.1 (Ar), 127.8 (C-18), 127.2 (C-17), 125.8 (Ar), 122.9 (Ar), 122.3 (Ar), 82.6 (C-1), 35.8 (C-6), 31.3 (C-5), 27.9 (C-4), 14.3 (C-20) ppm.

HRMS (ESI) m/z: $[M+H]^+$ calcd for $C_{24}H_{26}NO^+$ 344.2009, found 344.2014.

IR (thin film): ṽ 3371 (br), 1590 (s), 1431 (s), 1374 (s), 1025 (s) cm^{-1}.

Specific rotation: $[\alpha]_D^{23}$ = +25.5 (*c* 0.5, CH_2Cl_2).

The **enantiomeric ratio** of **3bo** was determined by SFC analysis (CHIRALPACK SC (25 cm), column temperature 25 °C, solvent CO_2/MeOH (with 0.5% Et_3N) = 96/4, flow rate = 2.0 mL/min): t_R = 10.4 min (minor), t_R = 11.3 min (major).

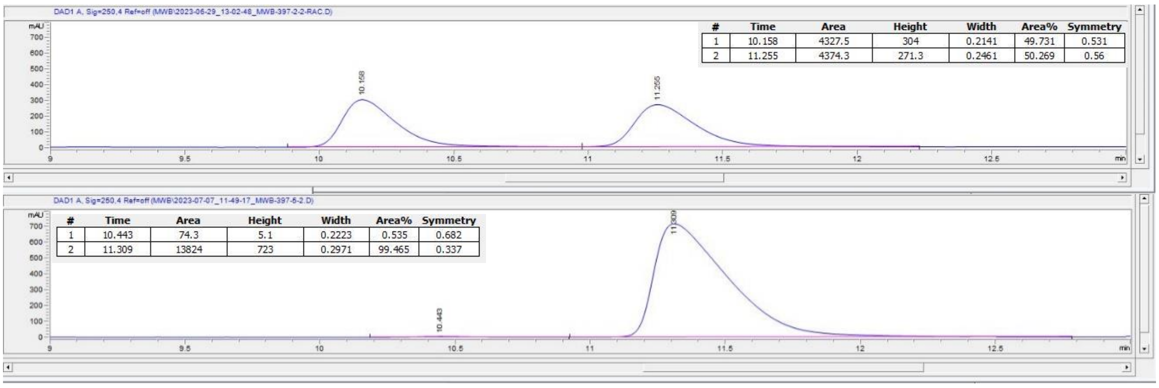

Characteristic signals for regioisomer *iso*-**3bo**

^{1}H NMR (500 MHz, $CDCl_3$, 298 K): 4.97 (q, *J* = 6.9 Hz, 1H), 1.58 (d, *J* = 6.9 Hz, 3H).

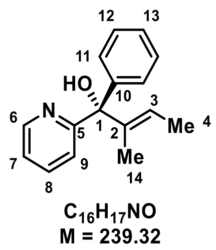

(S,E)-2-Methyl-1-phenyl-1-(pyridin-2-yl)but-2-en-1-ol (**3bp**, **GP 6**) was prepared from phenyl(pyridin-2-yl)methanol (**1b**, 18.5 mg, 0.10 mmol, 100 mol%) and but-2-yne (**2p**, 58.7 µL, 40.6 mg, 0.75 mmol, 750 mol%), using [Ir(cod)$_2$]BARF (6.36 mg, 5.0 µmol, 5.0 mol%), **L1** (3.11 mg, 5.0 µmol, 5.0 mol%) and *i*-PrOH (19.1 µL, 15.0 mg, 0.25 mmol, 250 mol%) in toluene (0.2 mL, 0.5 M) at 110 °C for 48 h. Purification by flash column chromatography on silica gel (hexane/ethyl acetate from 100/1 to 19/1) afforded **3bp** (16.8 mg, 70% yield, 99% ee) as a colorless oil.

R$_f$ = 0.3 (Hexane/EtOAc = 15/1).

^{1}H NMR (500 MHz, CDCl$_3$, 298 K): δ 8.57 (d, J = 4.9 Hz, 1H, H-6), 7.65–7.60 (m, 1H, H-8), 7.44–7.41 (m, 2H, H-11), 7.34–7.29 (m, 2H, H-12), 7.27–7.22 (m, 1H, H-13), 7.21–7.15 (m, 2H, H-7 and H-9), 5.95 (s, 1H, OH), 4.98 (q, J = 6.7 Hz, 1H, H-3), 1.63 (s, 3H, H-14), 1.42 (d, J = 6.7 Hz, 3H, H-4) ppm.

^{13}C NMR (125 MHz, CDCl$_3$, 298 K): δ 162.5 (C-5), 147.9 (C-6), 144.7 (C-10), 140.8 (C-2), 136.3 (C-8), 128.1 (C-12), 127.8 (C-11), 127.2 (C-13), 124.4 (C-3), 122.9 (C-7), 122.3 (C-9), 82.6 (C-1), 14.0 (C-14), 12.8 (C-4) ppm.

HRMS (ESI) m/z: [M+H]$^+$ calcd for $C_{16}H_{18}NO^+$ 240.1383, found 240.1389.

IR (thin film): ṽ 3371 (br), 1590 (s), 1431 (s), 1372 (s), 1173 (s), 1025 (s) cm^{-1}.

Specific rotation: $[\alpha]_D^{23}$ = +62.7 (c 0.5, CH_2Cl_2).

The **enantiomeric ratio** of **3bp** was determined by SFC analysis (CHIRALPACK IC (25 cm), column temperature 25 °C, solvent CO_2/MeOH (with 0.5% Et_3N) = 99/1, flow rate = 1.5 mL/min): t_R = 16.6 min (minor), t_R = 17.6 min (major).

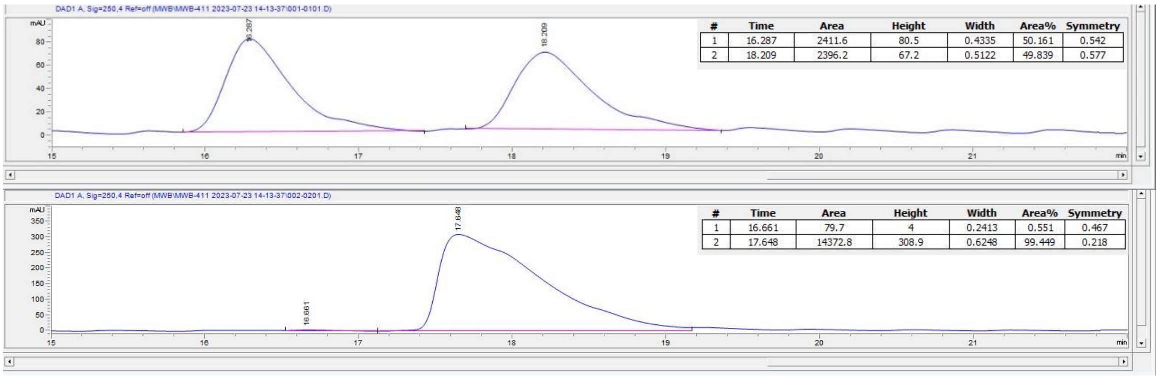

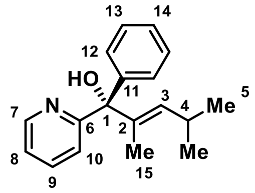

$C_{18}H_{21}NO$
M = 267.37

(*S*,*E*)-2,4-dimethyl-1-phenyl-1-(pyridin-2-yl)pent-2-en-1-ol (**3bq**, **GP 6**) was prepared from phenyl(pyridin-2-yl)methanol (**1b**, 18.5 mg, 0.10 mmol, 100 mol%) and 4-methyl-2-pentyne (**2q**, 88 µL, 61.6 mg, 0.75 mmol, 750 mol%), using [Ir(cod)$_2$]BARF (12.7 mg, 10 µmol, 10 mol%), **L1** (6.23 mg, 10 µmol, 10 mol%) and *i*-PrOH (19.1 µL, 15.0 mg, 0.25 mmol, 250 mol%) in toluene (0.2 mL, 0.5 M) at 110 °C for 48 h. Purification by flash column chromatography on silica gel (hexane/ethyl acetate from 100/1 to 19/1) afforded **3bq** (19.4 mg, 73% yield, 98% ee, >25:1 r.r.) as a colorless solid.

R*f* = 0.2 (Hexane/EtOAc = 15/1).

M.P. 58–59 °C (Hexane/EtOAc).

^{1}H NMR (400 MHz, $CDCl_3$, 298 K): δ 8.56 (d, *J* = 4.9 Hz, 1H, H-7), 7.65–7.60 (m, 1H, H-9), 7.43–7.39 (m, 2H, H-12), 7.34–7.28 (m, 2H, H-13), 7.27–7.22 (m, 1H, H-14), 7.22–7.15 (m, 2H, H-8 and H-10), 5.99 (s, 1H, OH), 4.76–4.71 (m, 1H, H-3), 2.65–2.54 (m, 1H, H-4), 1.69 (s, 3H, H-15), 0.93 (d, *J* = 6.6 Hz, 3H, H-5), 0.89 (d, *J* = 6.6 Hz, 3H, H-5) ppm.

^{13}C NMR (100 MHz, $CDCl_3$, 298 K): δ 162.4 (C-6), 147.8 (C-7), 144.8 (C-11), 137.7 (C-3), 137.5 (C-2), 136.2 (C-9), 128.0 (C-13), 127.8 (C-12), 127.1 (C-14), 122.9 (C-8), 122.2 (C-10), 82.4 (C-1), 27.5 (C-4), 22.9 (C-5), 22.7 (C-5), 14.1 (C-15) ppm.

HRMS (ESI) m/z: $[M+H]^+$ calcd for $C_{18}H_{22}NO^+$ 268.1696, found 268.1698.

IR (thin film): ṽ 3376 (br), 2956 (s), 1591 (s), 1431 (s), 1374 (s), 1205 (s), 1150 (s), 1028 (s) cm^{-1}.

Specific rotation: $[\alpha]_D^{25}$ = +50.1 (*c* 0.5, CH_2Cl_2).

The **enantiomeric ratio** of **3bq** was determined by SFC analysis (CHIRALPACK SC (25 cm), column temperature 25 °C, solvent CO_2/MeOH (with 0.5% Et_3N) = 97/3, flow rate = 2.0 mL/min): t_R = 4.2 min (minor), t_R = 4.5 min (major).

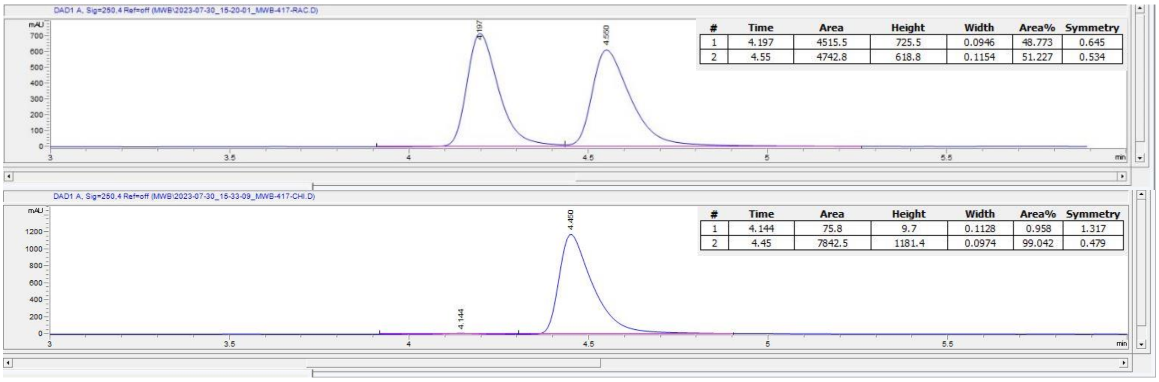

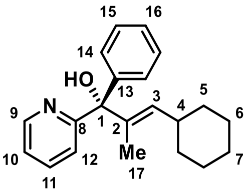

$C_{21}H_{25}NO$
M = 307.44

(*S*,*E*)-3-cyclohexyl-2-methyl-1-phenyl-1-(pyridin-2-yl)prop-2-en-1-ol (**3br**, **GP 6**) was prepared from phenyl(pyridin-2-yl)methanol (**1b**, 18.5 mg, 0.10 mmol, 100 mol%) and prop-1-yn-1-ylcyclohexane (**2r**, 91.7 mg, 0.75 mmol, 750 mol%), using [Ir(cod)$_2$]BARF (12.7 mg, 10 µmol, 10 mol%), **L1** (6.23 mg, 10 µmol, 10 mol%) and *i*-PrOH (19.1 µL, 15.0 mg, 0.25 mmol, 250 mol%) in toluene (0.2 mL, 0.5 M) at 110 °C for 48 h. Purification by flash column chromatography on silica gel (hexane/ethyl acetate from 100/1 to 19/1) afforded **3br** (20.8 mg, 68% yield, 91% ee, >25:1 r.r.) as a colorless solid.

R*f* = 0.2 (Hexane/EtOAc = 15/1).

M.P. 71–72 °C (Hexane/EtOAc).

^{1}H NMR (400 MHz, $CDCl_3$, 298 K): δ 8.56 (d, *J* = 4.9 Hz, 1H, H-9), 7.65–7.59 (m, 1H, H-11), 7.43–7.37 (m, 2H, H-14), 7.33–7.27 (m, 2H, H-15), 7.27–7.22 (m, 1H, H-16), 7.22–7.15 (m, 2H, H-10 and H-12), 5.98 (s, 1H, OH), 4.74 (d, *J* = 9.0 Hz, 1H, H-3), 2.31–2.19 (m, 1H, H-4), 1.69 (s, 3H, H-17), 1.71–1.54 (m, 5H, H-5 or CyH), 1.35–1.19 (m, 2H, CyH), 1.19–1.05 (m, 1H, CyH), 1.05–0.86 (m, 2H, H-5) ppm.

^{13}C NMR (100 MHz, $CDCl_3$, 298 K): δ 162.4 (C-8), 147.8 (C-9), 144.8 (C-13), 137.9 (C-2), 136.2 (C-3 and C-11), 128.0 (C-15), 127.9 (C-14), 127.1 (C-16), 122.9 (C-10), 122.2 (C-12), 82.5 (C-1), 37.3 (C-4), 33.0 (C-5), 32.8 (C-5), 26.2 (C-6 or C-7), 26.1 (C-6 or C-7), 26.0 (C-6 or C-7), 14.2 (C-17) ppm.

HRMS (ESI) m/z: $[M–H_2O+H]^+$ calcd for $C_{21}H_{24}N^+$ 290.1903, found 290.1906.

IR (thin film): ṽ 3388 (br), 2921 (s), 2849 (s), 1590 (s), 1431 (s), 1373 (s), 1028 (s) cm^{-1}.

Specific rotation: $[\alpha]_D^{24}$ = +51.7 (*c* 0.5, CH_2Cl_2).

The **enantiomeric ratio** of **3br** was determined by SFC analysis (CHIRALPACK SC (25 cm), column temperature 25 °C, solvent CO_2/MeOH (with 0.5% Et_3N) = 97/3, flow rate = 2.0 mL/min): t_R = 7.0 min (minor), t_R = 7.9 min (major).

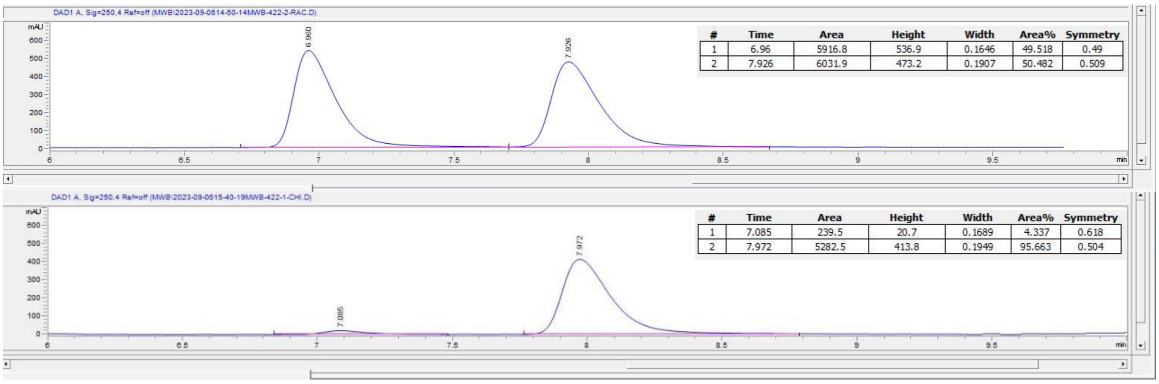

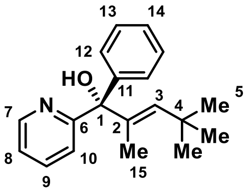

$C_{19}H_{23}NO$
M = 281.40

(*S*,*E*)-2,4,4-trimethyl-1-phenyl-1-(pyridin-2-yl)pent-2-en-1-ol (**3bs**, **GP 6**) was prepared from phenyl(pyridin-2-yl)methanol (**1b**, 18.5 mg, 0.10 mmol, 100 mol%) and 4,4-dimethyl-2-pentyne (**2s**, 100 µL, 72.1 mg, 0.75 mmol, 750 mol%), using $[Ir(cod)_2]$BARF (12.7 mg, 10 µmol, 10 mol%), **L1** (6.23 mg, 10 µmol, 10 mol%) and *i*-PrOH (19.1 µL, 15.0 mg, 0.25 mmol, 250 mol%) in toluene (0.2 mL, 0.5 M) at 110 °C for 48 h. Purification by flash column chromatography on silica gel (hexane/ethyl acetate from 100/1 to 19/1) afforded **3bs** (16.8 mg, 63% yield, 99% ee, >25:1 r.r.) as a colorless solid.

R*f* = 0.4 (Hexane/EtOAc = 15/1).

M.P. 43–44 °C (Hexane/EtOAc).

^{1}H NMR (400 MHz, $CDCl_3$, 298 K): δ 8.56 (d, *J* = 4.8 Hz, 1H, H-7), 7.64–7.60 (m, 1H, H-9), 7.42–7.39 (m, 2H, H-12), 7.33–7.28 (m, 2H, H-13), 7.26–7.21 (m, 1H, H-14), 7.21–7.15 (m, 2H, H-8 and H-10), 5.98 (s, 1H, OH), 4.94 (s, 1H, H-3), 1.78 (s, 3H, H-15), 1.08 (s, 9H, H-5) ppm.

^{13}C NMR (100 MHz, $CDCl_3$, 298 K): δ 162.6 (C-6), 147.8 (C-7), 145.0 (C-11), 140.2 (C-3), 138.9 (C-2), 136.2 (C-9), 128.0 (C-13), 127.9 (C-12), 127.1 (C-14), 123.1 (C-8), 122.1 (C-10), 83.4 (C-1), 32.5 (C-4), 30.9 (C-5), 15.3 (C-15) ppm.

HRMS (ESI) m/z: $[M+H]^+$ calcd for $C_{19}H_{24}NO^+$ 282.1853, found 282.1856.

IR (thin film): ṽ 3378 (br), 2955 (s), 1590 (s), 1432 (s), 1362 (s), 1041 (s) cm^{-1}.

Specific rotation: $[\alpha]_D^{22}$ = +38.5 (*c* 0.5, CH_2Cl_2).

The **enantiomeric ratio** of **3bs** was determined by SFC analysis (CHIRALPACK IC (25 cm), column temperature 25 °C, solvent CO_2/MeOH (with 0.5% Et_3N) = 99/1, flow rate = 1.5 mL/min): t_R = 10.3 min (minor), t_R = 11.0 min (major).

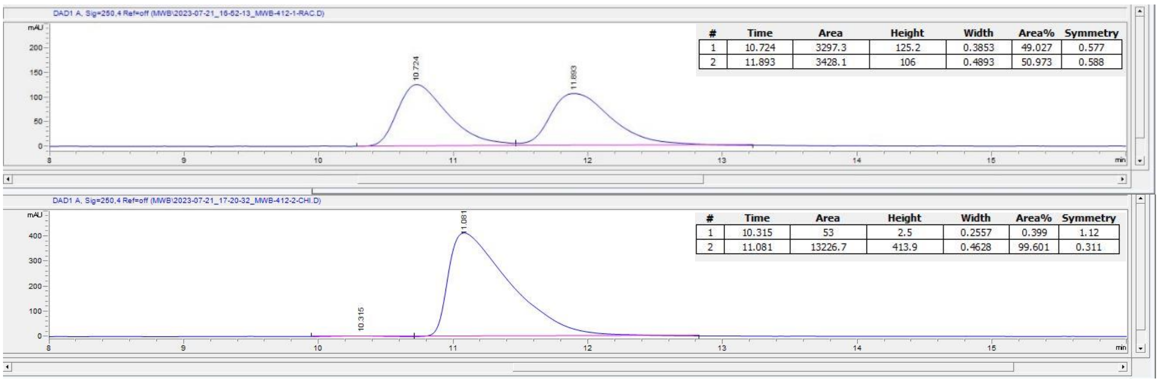

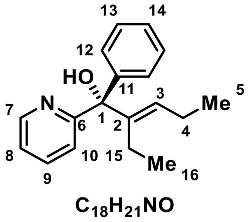

$C_{18}H_{21}NO$
M = 267.37

(*S*,*E*)-2-Ethyl-1-phenyl-1-(pyridin-2-yl)pent-2-en-1-ol (**3bt**, **GP 6**) was prepared from phenyl(pyridin-2-yl)methanol (**1b**, 18.5 mg, 0.10 mmol, 100 mol%) and hex-3-yne (**2t**, 85.4 µL, 61.6 mg, 0.75 mmol, 750 mol%), using $[Ir(cod)_2]$BARF (12.7 mg, 10 µmol, 10 mol%), **L1** (6.23 mg, 10 µmol, 10 mol%) and *i*-PrOH (19.1 µL, 15.0 mg, 0.25 mmol, 250 mol%) in toluene (0.2 mL, 0.5 M) at 110 °C for 48 h. Purification by flash column chromatography on silica gel

(hexane/ethyl acetate from 100/1 to 19/1) afforded **3bt** (19.5 mg, 73% yield, >99% ee) as a colorless oil.

R*f* = 0.3 (Hexane/EtOAc = 15/1).

^{1}H NMR (400 MHz, $CDCl_3$, 298 K): δ 8.58 (d, *J* = 4.7 Hz, 1H, H-7), 7.64–7.58 (m, 1H, H-9), 7.43–7.39 (m, 2H, H-12), 7.33–7.28 (m, 2H, H-13), 7.27–7.23 (m, 1H, H-14), 7.23–7.18 (m, 1H, H-8), 7.18–7.14 (m, 1H, H-10), 5.90 (s, 1H, OH), 4.87 (t, *J* = 7.2 Hz, 1H, H-3), 2.20–2.05 (m, 4H, H-4 and H-15), 1.01 (t, *J* = 7.5 Hz, 3H, H-16), 0.94 (t, *J* = 7.5 Hz, 3H, H-5) ppm.

^{13}C NMR (100 MHz, $CDCl_3$, 298 K): δ 162.7 (C-6), 147.8 (C-7), 145.3 (C-2 or C-11), 145.2 (C-2 or C-11), 136.1 (C-9), 132.1 (C-3), 128.0 (C-12 or C-13), 128.0 (C-12 or C-13), 127.2 (C-14), 123.1 (C-8), 122.2 (C-10), 83.3 (C-1), 22.7 (C-15), 21.5 (C-4), 15.4 (C-16), 14.4 (C-5) ppm.

HRMS (ESI) m/z: $[M–H_2O+H]^+$ calcd for $C_{18}H_{20}N^+$ 250.1590, found 250.1593.

IR (thin film): ṽ 3386 (br), 2962 (s), 1590 (s), 1432 (s), 1371 (s), 1173 (s), 1038 (s) cm^{-1}.

Specific rotation: $[\alpha]_D^{23}$ = +61.1 (*c* 0.5, CH_2Cl_2).

The **enantiomeric ratio** of **3bt** was determined by SFC analysis (CHIRALPACK IC (25 cm), column temperature 25 °C, solvent CO_2/MeOH (with 0.5% Et_3N) = 99/1, flow rate = 2.0 mL/min): t_R = 11.3 min (major), t_R = 12.5 min (minor).

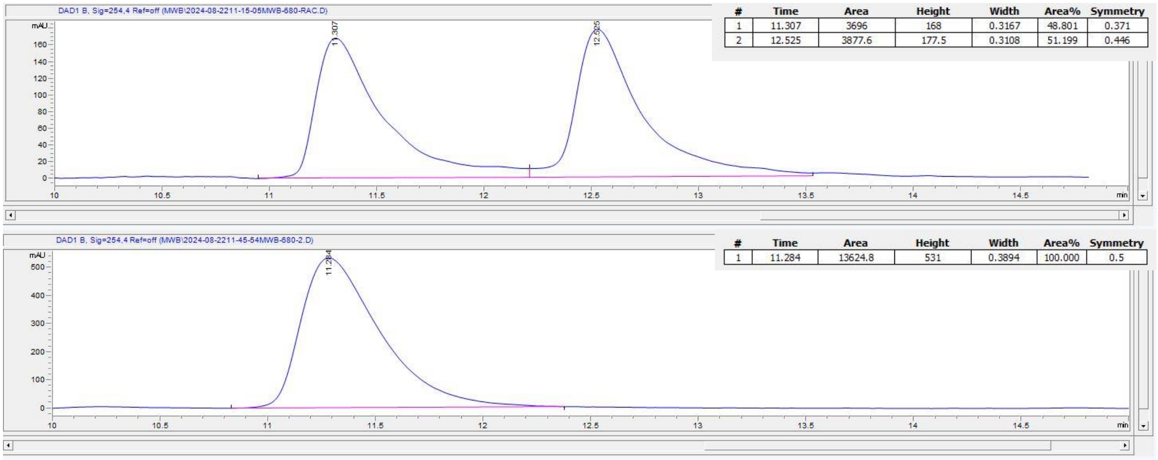

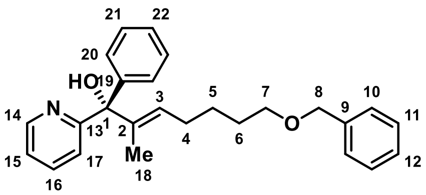

$C_{26}H_{29}NO_2$
M = 387.52

(*S*,*E*)-7-(benzyloxy)-2-methyl-1-phenyl-1-(pyridin-2-yl)hept-2-en-1-ol (**3bu**, **GP 6**) was prepared from phenyl(pyridin-2-yl)methanol (**1b**, 18.5 mg, 0.10 mmol, 100 mol%) and ((hept-5-yn-1-yloxy)methyl)benzene (**2u**, 152 mg, 0.75 mmol, 750 mol%), using [Ir(cod)$_2$]BARF (12.7 mg, 10 μmol, 10 mol%), **L1** (6.23 mg, 10 μmol, 10 mol%) and *i*-PrOH (19.1 μL, 15.0 mg, 0.25 mmol, 250 mol%) in toluene (0.2 mL) at 110 °C for 48 h. Purification by flash column chromatography on silica gel (hexane/ethyl acetate from 100/1 to 19/1) afforded **3bu** (28.1 mg, 72% yield, >99% ee, >25:1 r.r.) as a colorless oil. The regioisomer ratio of the process (6:1 r.r.) was determined from the ^{1}H NMR spectrum of crude material.

R*f* = 0.2 (Hexane/EtOAc = 9/1).

^{1}H NMR (500 MHz, $CDCl_3$, 298 K): δ 8.56 (d, *J* = 5.0 Hz, 1H, H-14), 7.64–7.59 (m, 1H, H-16), 7.42 (d, *J* = 7.3 Hz, 2H, H-20), 7.37–7.22 (m, 8H, H-10, H-11, H-12, H-21 and H-22), 7.22–7.14 (m, 2H, H-15 and H-17), 5.98 (s, 1H, OH), 4.90 (t, *J* = 7.1 Hz, 1H, H-3), 4.49 (s, 2H, H-8), 3.45 (t, *J* = 6.6 Hz, 2H, H-7), 2.12–2.03 (m, 2H, H-4), 1.68 (s, 3H, H-18), 1.64–1.56 (m, 2H, H-6), 1.44–1.36 (m, 2H, H-5) ppm.

^{13}C NMR (125 MHz, $CDCl_3$, 298 K): δ 162.3 (C-13), 147.8 (C-14), 144.6 (C-19), 140.0 (C-2), 138.7 (C-9), 136.3 (C-16), 130.1 (C-3), 128.5 (Ar), 128.1 (Ar), 127.8 (Ar), 127.8 (Ar), 127.6 (Ar), 127.2 (Ar), 122.9 (C-15 or C-17), 122.3 (C-15 or C-17), 82.6 (C-1), 73.0 (C-8), 70.4 (C-7), 29.6 (C-6), 28.1 (C-4), 26.1 (C-5), 14.2 (C-18) ppm.

HRMS (ESI) m/z: $[M+H]^+$ calcd for $C_{26}H_{30}NO_2^+$ 388.2271, found 388.2266.

IR (thin film): ṽ 3357 (br), 1739 (s), 1590 (s), 1432 (s), 1370 (s), 1027 (s) cm^{-1}.

Specific rotation: $[\alpha]_D^{24}$ = +32.3 (*c* 0.5, CH_2Cl_2).

The **enantiomeric ratio** of **3bu** was determined by SFC analysis (CHIRALPACK IC (25 cm), column temperature 25 °C, solvent CO_2/MeOH (with 0.5% Et_3N) = 95/5, flow rate = 2.0 mL/min): t_R = 16.4 min (minor), t_R = 18.1 min (major).

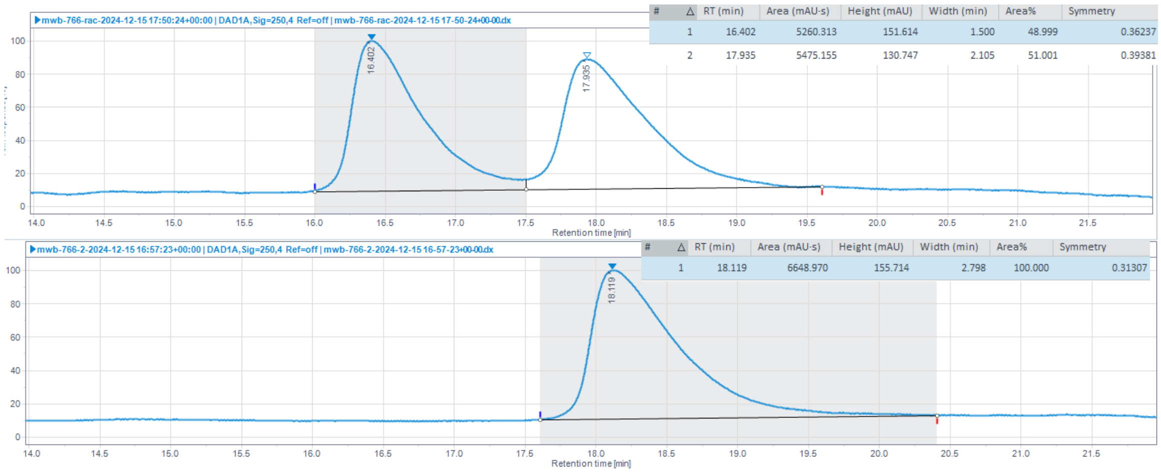

Characteristic signals for regioisomer *iso*-**3bu**

[1] **H NMR** (500 MHz, $CDCl_3$, 298 K): 4.98 (q, *J* = 6.8 Hz, 1H), 4.46 (s, 2H), 1.65 (d, *J* = 6.8 Hz, 3H).

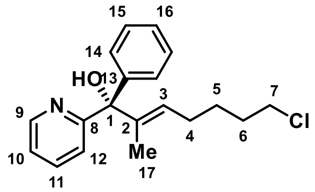

$C_{19}H_{22}ClNO$
M = 315.84

(*S*,*E*)-6-Chloro-2-methyl-1-phenyl-1-(pyridin-2-yl)hex-2-en-1-ol (**3bv**, **GP 6**) was prepared from phenyl(pyridin-2-yl)methanol (**1b**, 18.5 mg, 0.10 mmol, 100 mol%) and 6-chlorohex-2-yne (**2v**, 87.4 mg, 0.75 mmol, 750 mol%), using $[Ir(cod)_2]$BARF (12.7 mg, 10 µmol, 10 mol%), **L1** (6.23 mg, 10 µmol, 10 mol%) and *i*-PrOH (19.1 µL, 15.0 mg, 0.25 mmol, 250 mol%) in toluene (0.2 mL) at 110 °C for 48 h. Purification by flash column chromatography on silica gel (hexane/ethyl acetate from 100/1 to 19/1) afforded **3bv** (19.6 mg, 62% yield, 96% ee, >25:1 r.r.) as a colorless oil. The regioisomer ratio of the process (19:1 r.r.) was determined from the ^{1}H NMR spectrum of crude material.

R*f* = 0.5 (Hexane/EtOAc = 9/1).

^{1}H NMR (400 MHz, $CDCl_3$, 298 K): δ 8.57 (d, *J* = 4.5 Hz, 1H, H-9), 7.66–7.61 (m, 1H, H-11), 7.41 (d, *J* = 7.6 Hz, 2H, H-14), 7.35–7.28 (m, 2H, H-15), 7.28–7.24 (m, 1H, H-16), 7.24–7.15 (m, 2H, H-10 and H-12), 5.99 (s, 1H, OH), 4.90 (t, *J* = 7.1 Hz, 1H, H-3), 3.51 (t, *J* = 6.6 Hz, 2H, H-7), 2.15–2.05 (m, 2H, H-4), 1.79–1.72 (m, 2H, H-6), 1.69 (s, 3H, H-17), 1.51–1.41 (m, 2H, H-5) ppm.

^{13}C NMR (100 MHz, $CDCl_3$, 298 K): δ 162.3 (C-8), 147.8 (C-9), 144.5 (C-13), 140.6 (C-2), 136.4 (C-11), 129.4 (C-3), 128.1 (C-14 or C-15), 127.8 (C-14 or C-15), 127.2 (16), 122.9 (C-10 or C-12), 122.3 (C-10 or C-12), 82.5 (C-1), 45.1 (C-7), 32.4 (C-6), 27.6 (C-4), 26.7 (C-5), 14.3 (C-17) ppm.

HRMS (ESI) m/z: $[M–H_2O+H]^+$ calcd for $C_{19}H_{21}{}^{35}ClN^+$ 298.1357, found 298.1361.

IR (thin film): ṽ 3375 (br), 1590 (s), 1431 (s), 1373 (s), 1027 (s) cm^{-1}.

Specific rotation: $[\alpha]_D^{23}$ = +40.5 (*c* 1.0, CH_2Cl_2).

The **enantiomeric ratio** of **3bv** was determined by SFC analysis (CHIRALPACK IC (25 cm), column temperature 25 °C, solvent CO_2/MeOH (with 0.5% Et_3N) = 95/5, flow rate = 2.0 mL/min): t_R = 7.0 min (minor), t_R = 7.6 min (major).

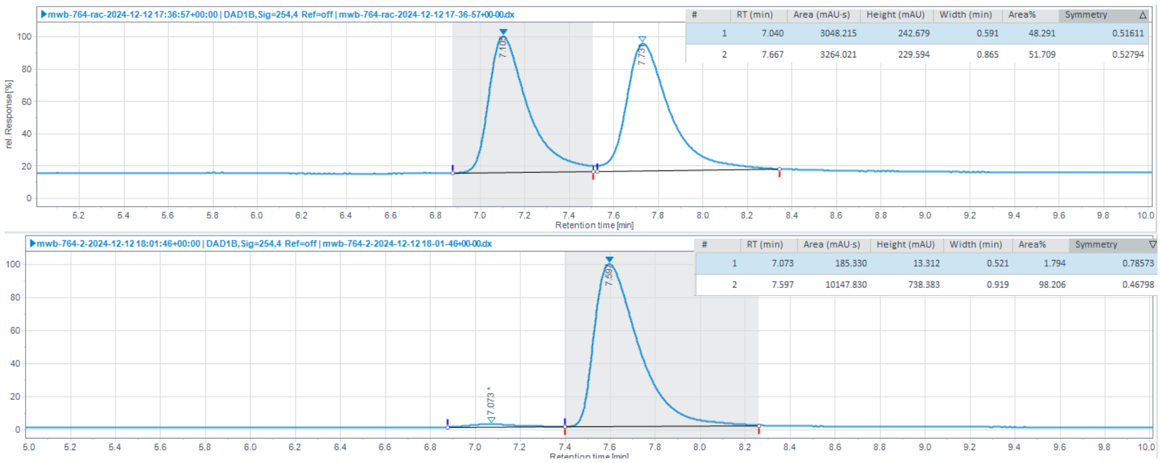

Characteristic signals for regioisomer *iso*-**3bv**

^{1} H NMR (500 MHz, $CDCl_3$, 298 K): 5.90 (S, 1H), 4.97 (q, *J* = 6.9 Hz, 1H), 3.48 (t, *J* = 6.8 Hz, 2H), 1.66 (d, *J* = 6.9 Hz, 3H).

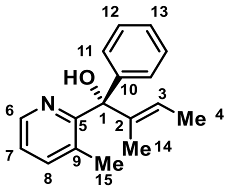

$C_{17}H_{19}NO$
M = 253.35

(*S*,*E*)-2-methyl-1-(3-methylpyridin-2-yl)-1-phenylbut-2-en-1-ol (**3cp**, **GP 6**) was prepared from (3-methylpyridin-2-yl)(phenyl)methanol (**1c**, 19.9 mg, 0.10 mmol, 100 mol%) and but-2-yne (**2p**, 58.7 µL, 40.6 mg, 0.75 mmol, 750 mol%), using $[Ir(cod)_2]$BARF (12.7 mg, 10 µmol, 10 mol%), **L1** (6.23 mg, 10 µmol, 10 mol%) and *i*-PrOH (19.1 µL, 15.0 mg, 0.25 mmol, 250

mol%) in toluene (0.2 mL, 0.5 M) at 110 °C for 48 h. Purification by flash column chromatography on silica gel (hexane/ethyl acetate from 100/1 to 19/1) afforded **3cp** (3.00 mg, 12% yield) as a colorless solid.

R*f* = 0.4 (Hexane/EtOAc = 15/1).

M.P. 84–85 °C (Hexane/EtOAc).

^{1}H NMR (500 MHz, $CDCl_3$, 298 K): δ 8.44 (d, *J* = 4.8 Hz, 1H, H-6), 7.47–7.44 (m, 1H, H-8), 7.28–7.25 (m, 4H, H-11 and H-12), 7.24–7.18 (m, 2H, H-7 and H-13), 7.17 (s, 1H, OH), 5.11 (q, *J* = 6.7 Hz, 1H, H-3), 2.05 (s, 3H, H-15), 1.73 (s, 3H, H-14), 1.64 (d, *J* = 6.7 Hz, 3H, H-4) ppm.

^{13}C NMR (125 MHz, $CDCl_3$, 298 K): δ 159.5 (C-5), 144.7 (C-6), 144.1 (C-10), 140.4 (C-8), 138.6 (C-2), 132.7 (C-9), 129.0 (C-12), 127.8 (C-11), 127.1 (C-13), 124.6 (C-3), 123.0 (C-7), 82.2 (C-1), 20.7 (C-15), 14.1 (C-14), 13.8 (C-4) ppm.

HRMS (ESI) m/z: $[M+H]^+$ calcd for $C_{17}H_{20}NO^+$ 254.1540, found 254.1536.

IR (thin film): ṽ 3291 (br), 1447 (s), 1365 (s), 1009 (s) cm^{-1}.

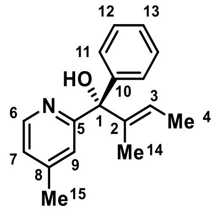

$C_{17}H_{19}NO$
M = 253.35

(*S*,*E*)-2-Methyl-1-(4-methylpyridin-2-yl)-1-phenylbut-2-en-1-ol (**3dp**, **GP 6**) was prepared from (4-methylpyridin-2-yl)(phenyl)methanol (**1d**, 19.9 mg, 0.10 mmol, 100 mol%) and but-2-yne (**2p**, 58.7 µL, 40.6 mg, 0.75 mmol, 750 mol%), using $[Ir(cod)_2]$BARF (6.36 mg, 5.0 µmol, 5.0 mol%), **L1** (3.11 mg, 5.0 µmol, 5.0 mol%) and *i*-PrOH (19.1 µL, 15.0 mg, 0.25 mmol, 250 mol%) in toluene (0.2 mL, 0.5 M) at 110 °C for 48 h. Purification by flash column chromatography on silica gel (hexane/ethyl acetate from 100/1 to 19/1) afforded **3dp** (18.6 mg, 73% yield, 98% ee) as a brown oil.

R*f* = 0.5 (Hexane/EtOAc = 15/1).

^{1}H NMR (500 MHz, $CDCl_3$, 298 K): δ 8.41 (d, *J* = 5.0 Hz, 1H, H-6), 7.44–7.41 (m, 2H, H-11), 7.34–7.29 (m, 2H, H-12), 7.27–7.22 (m, 1H, H-13), 7.01 (d, *J* = 5.0 Hz, 1H, H-7), 6.96 (s, 1H, H-9), 5.99 (s, 1H, OH), 4.98 (q, *J* = 6.7 Hz, 1H, H-3), 2.32 (s, 3H, H-15), 1.68 (s, 3H, H-14), 1.63 (d, *J* = 6.7 Hz, 3H, H-4) ppm.

^{13}C NMR (125 MHz, $CDCl_3$, 298 K): δ 162.3 (C-5), 147.6 (C-8 or C-6), 147.5 (C-8 or C-6), 144.8 (C-10), 140.8 (C-2), 128.0 (C-12), 127.9 (C-11), 127.1 (C-13), 124.2 (C-3), 123.6 (C-9), 123.0 (C-7), 82.5 (C-1), 21.4 (C-15), 14.1 (C-14), 13.8 (C-4) ppm.

HRMS (ESI) m/z: $[M+H]^+$ calcd for $C_{17}H_{20}NO^+$ 254.1540, found 254.1532.

IR (thin film): ṽ 3371 (br), 1604 (s), 1446 (s), 1371 (s), 1174 (s), 1028 (s), 817 (s) cm^{-1}.

Specific rotation: $[\alpha]_D^{25}$ = +2.1 (*c* 0.5, CH_2Cl_2).

The **enantiomeric ratio** of **3dp** was determined by SFC analysis (CHIRALPACK IC (25 cm), column temperature 25 °C, solvent CO_2/MeOH (with 0.5% Et_3N) = 97/3, flow rate = 1.0 mL/min): t_R = 16.1 min (minor), t_R = 17.4 min (major).

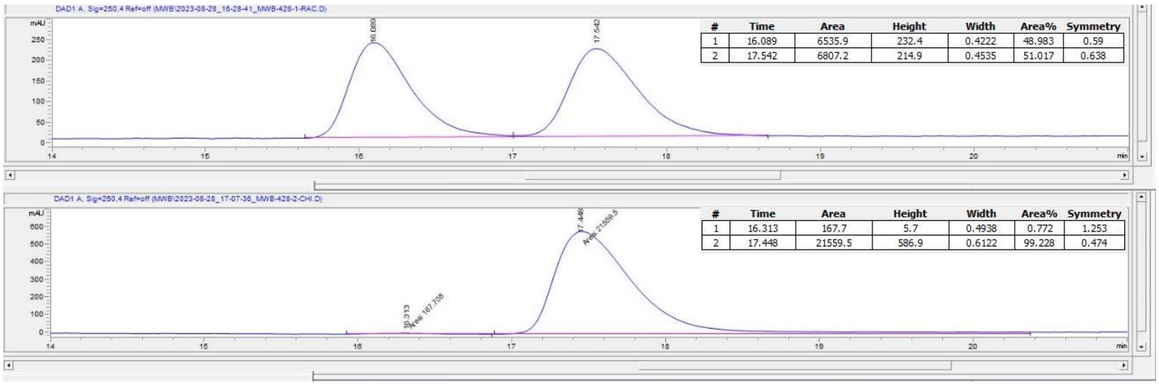

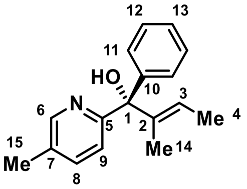

$C_{17}H_{19}NO$
M = 253.35

(*S*,*E*)-2-Methyl-1-(5-methylpyridin-2-yl)-1-phenylbut-2-en-1-ol (**3ep**, **GP 6**) was prepared from (5-methylpyridin-2-yl)(phenyl)methanol (**1e**, 19.9 mg, 0.10 mmol, 100 mol%) and but-2-yne (**2p**, 58.7 µL, 40.6 mg, 0.75 mmol, 750 mol%), using $[Ir(cod)_2]$BARF (6.36 mg, 5.0 µmol, 5.0 mol%), **L1** (3.11 mg, 5.0 µmol, 5.0 mol%) and *i*-PrOH (19.1 µL, 15.0 mg, 0.25 mmol, 250 mol%) in toluene (0.2 mL, 0.5 M) at 110 °C for 48 h. Purification by flash column chromatography on silica gel (hexane/ethyl acetate from 100/1 to 19/1) afforded **3ep** (15.4 mg, 61% yield, 97% ee) as a brown oil.

R_f = 0.5 (Hexane/EtOAc = 15/1).

^{1}H NMR (500 MHz, $CDCl_3$, 298 K): δ 8.38 (s, 1H, H-6), 7.44–7.40 (m, 3H, H-8 and H-11), 7.33–7.28 (m, 2H, H-12), 7.25–7.21 (m, 1H, H-13), 7.05 (d, *J* = 8.0 Hz, 1H, H-9), 5.92 (s, 1H, OH),

4.98 (q, J = 6.7 Hz, 1H, H-3), 2.33 (s, 3H, H-15), 1.68 (s, 3H, H-14), 1.62 (d, J = 6.7 Hz, 3H, H-4) ppm.

^{13}C NMR (125 MHz, $CDCl_3$, 298 K): δ 159.6 (C-5), 148.1 (C-6), 144.9 (C-10), 140.9 (C-2), 137.0 (C-8), 131.8 (C-7), 128.0 (C-12), 127.8 (C-11), 127.1 (C-13), 124.1 (C-3), 122.4 (C-9), 82.4 (C-1), 18.2 (C-15), 14.0 (C-14), 13.8 (C-4) ppm.

HRMS (ESI) m/z: $[M+H]^+$ calcd for $C_{17}H_{20}NO^+$ 254.1540, found 254.1531.

IR (thin film): $\tilde{\nu}$ 3376 (br), 1600 (s), 1446 (s), 1373 (s), 1174 (s), 1026 (s), 753 (s) cm^{-1}.

Specific rotation: $[\alpha]_D^{26}$ = +37.4 (c 0.5, CH_2Cl_2).

The **enantiomeric ratio** of **3ep** was determined by SFC analysis (CHIRALPACK IC (25 cm), column temperature 25 °C, solvent CO_2/MeOH (with 0.5% Et_3N) = 97/3, flow rate = 1.0 mL/min): t_R = 15.7 min (minor), t_R = 17.2 min (major).

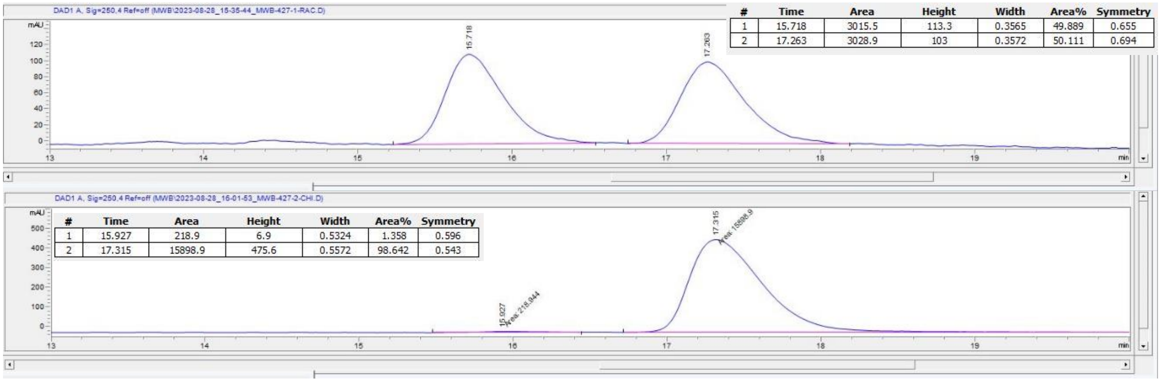

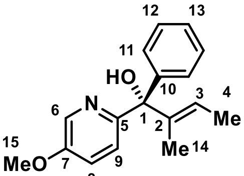

$C_{17}H_{19}NO_2$
M = 269.34

(*S*,*E*)-1-(5-Methoxypyridin-2-yl)-2-methyl-1-phenylbut-2-en-1-ol (**3gp**, **GP 6**) was prepared from (5-methoxypyridin-2-yl)(phenyl)methanol (**1g**, 21.5 mg, 0.10 mmol, 100 mol%) and but-2-yne (**2p**, 58.7 µL, 40.6 mg, 0.75 mmol, 750 mol%), using $[Ir(cod)_2]$BARF (6.36 mg, 5.0 µmol, 5.0 mol%), **L1** (3.11 mg, 5.0 µmol, 5.0 mol%) and *i*-PrOH (19.1 µL, 15.0 mg, 0.25 mmol, 250 mol%) in toluene (0.2 mL, 0.5 M) at 110 °C for 48 h. Purification by flash column chromatography on silica gel (hexane/ethyl acetate from 100/1 to 19/1) afforded **3gp** (14.7 mg, 61% yield, 95% ee) as a brown oil.

R_f = 0.2 (Hexane/EtOAc = 15/1).

^{1}H NMR (500 MHz, $CDCl_3$, 298 K): δ 8.24 (d, *J* = 2.9 Hz, 1H, H-6), 7.43–7.39 (m, 2H, H-11), 7.33–7.28 (m, 2H, H-12), 7.26–7.21 (m, 1H, H-13), 7.14 (dd, *J* = 8.7, 2.9 Hz, 1H, H-8), 7.06 (d, *J* = 8.7 Hz, 1H, H-9), 5.71 (s, 1H, OH), 4.96 (q, *J* = 6.7 Hz, 1H, H-3), 3.86 (s, 3H, H-15), 1.68 (s, 3H, H-14), 1.63 (d, *J* = 6.7 Hz, 3H, H-4) ppm.

^{13}C NMR (125 MHz, $CDCl_3$, 298 K): δ 154.7 (C-5 or C-7), 154.6 (C-7 or C-5), 145.0 (C-10), 141.0 (C-2), 134.8 (C-6), 128.0 (C-12), 127.8 (C-11), 127.1 (C-13), 124.1 (C-3), 123.1 (C-9), 121.4 (C-8), 82.3 (C-1), 55.9 (C-15), 14.0 (C-14), 13.8 (C-4) ppm.

HRMS (ESI) m/z: $[M–H_2O+H]^+$ calcd for $C_{17}H_{18}NO^+$ 252.1383, found 252.1382.

IR (thin film): ṽ 3402 (br), 2920 (w), 2859 (w), 1597 (w), 1477 (s), 1374 (s), 1270 (s), 1180 (m), 1027 (s), 700 (s) cm^{-1}.

Specific rotation: $[\alpha]_D^{26}$ = +34.8 (*c* 0.5, CH_2Cl_2).

The **enantiomeric ratio** of **3gp** was determined by SFC analysis (CHIRALPACK IC (25 cm), column temperature 25 °C, solvent CO_2/MeOH (with 0.5% Et_3N) = 97/3, flow rate = 1.0 mL/min): t_R = 19.4 min (minor), t_R = 20.9 min (major).

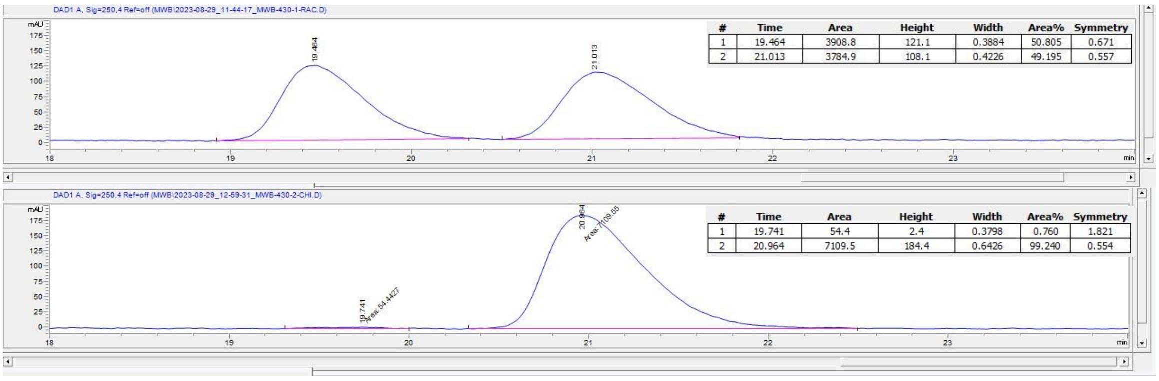

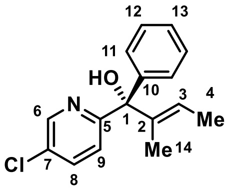

$C_{16}H_{16}ClNO$
M = 273.76

(*S*,*E*)-1-(5-Chloropyridin-2-yl)-2-methyl-1-phenylbut-2-en-1-ol (**3hp**, **GP 6**) was prepared from (5-chloropyridin-2-yl)(phenyl)methanol (**1h**, 22.0 mg, 0.10 mmol, 100 mol%) and but-2-yne (**2p**, 58.7 µL, 40.6 mg, 0.75 mmol, 750 mol%), using $[Ir(cod)_2]$BARF (6.36 mg, 5.0 µmol, 5.0 mol%), **L1** (3.11 mg, 5.0 µmol, 5.0 mol%) and *i*-PrOH (19.1 µL, 15.0 mg, 0.25 mmol, 250 mol%) in toluene (0.2 mL, 0.5 M) at 110 °C for 48 h. Purification by flash column

chromatography on silica gel (hexane/ethyl acetate from 100/1 to 19/1) afforded **3hp** (21.3 mg, 78% yield, 95% ee) as a brown oil.

R*f* = 0.5 (Hexane/EtOAc = 15/1).

^{1}H NMR (500 MHz, $CDCl_3$, 298 K): δ 8.53 (d, *J* = 2.4 Hz, 1H, H-6), 7.59 (dd, *J* = 8.5, 2.5 Hz, 1H, H-8), 7.42–7.38 (m, 2H, H-11), 7.35–7.30 (m, 2H, H-12), 7.28–7.24 (m, 1H, H-13), 7.12 (d, *J* = 8.5 Hz, 1H, H-9), 5.46 (s, 1H, OH), 4.97 (q, *J* = 6.7 Hz, 1H, H-3), 1.69 (s, 3H, H-14), 1.64 (d, *J* = 6.7 Hz, 3H, H-4) ppm.

^{13}C NMR (125 MHz, $CDCl_3$, 298 K): δ 161.0 (C-5), 146.9 (C-6), 144.1 (C-10), 140.5 (C-2), 136.1 (C-8), 130.7 (C-7), 128.2 (C-12), 127.7 (C-11), 127.4 (C-13), 124.7 (C-3), 123.7 (C-9), 82.6 (C-1), 14.0 (C-14), 13.8 (C-4) ppm.

HRMS (ESI) m/z: $[M–H_2O+H]^+$ calcd for $C_{16}H_{15}{}^{35}ClN^+$ 256.0887, found 256.0892.

IR (thin film): ṽ 3470 (br), 2920 (w), 2859 (w), 1578 (w), 1461 (s), 1366 (s), 1111 (s), 1013 (s), 765 (s) cm^{-1}.

Specific rotation: $[\alpha]_D^{25}$ = +24.4 (*c* 0.5, CH_2Cl_2).

The **enantiomeric ratio** of **3hp** was determined by SFC analysis (CHIRALPACK AD (25 cm), column temperature 25 °C, solvent CO_2/MeOH (with 0.5% Et_3N) = 97/3, flow rate = 1.0 mL/min): t_R = 19.6 min (major), t_R = 21.5 min (minor).

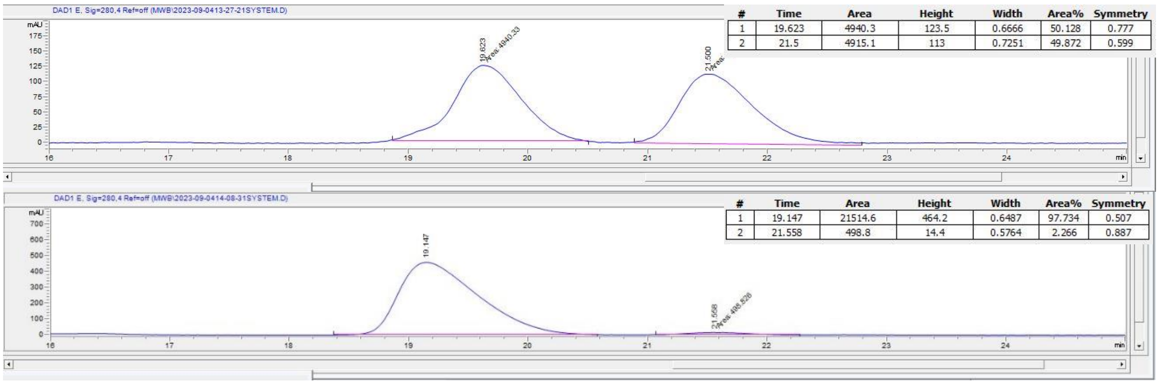

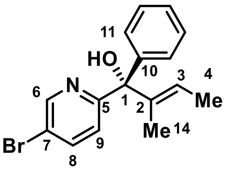

$C_{16}H_{16}BrNO$
M = 318.21

(*S*,*E*)-1-(5-Bromopyridin-2-yl)-2-methyl-1-phenylbut-2-en-1-ol (**3ip**, **GP 6**) was prepared from (5-chloropyridin-2-yl)(phenyl)methanol (**1i**, 26.4 mg, 0.10 mmol, 100 mol%) and but-2-yne (**2p**, 58.7 μL, 40.6 mg, 0.75 mmol, 750 mol%), using [Ir(cod)$_2$]BARF (6.36 mg, 5.0 μmol, 5.0 mol%), **L1** (3.11 mg, 5.0 μmol, 5.0 mol%) and *i*-PrOH (19.1 μL, 15.0 mg, 0.25 mmol, 250 mol%) in toluene (0.2 mL, 0.5 M) at 110 °C for 48 h. Purification by flash column chromatography on silica gel (hexane/ethyl acetate from 100/1 to 19/1) afforded **3ip** (23.2 mg, 73% yield, 92% ee) as a colorless oil.

R$_f$ = 0.6 (Hexane/EtOAc = 15/1).

^{1}H NMR (500 MHz, $CDCl_3$, 298 K): δ 8.61 (d, *J* = 2.3 Hz, 1H, H-6), 7.74 (dd, *J* = 8.5, 2.3 Hz, 1H, H-8), 7.40 (d, *J* = 7.2 Hz, 2H, H-11), 7.35–7.30 (m, 2H, H-12), 7.28–7.24 (m, 1H, H-13), 7.07 (d, *J* = 8.5 Hz, 1H, H-9), 5.45 (s, 1H, OH), 4.98 (q, *J* = 6.7 Hz, 1H, H-3), 1.69 (s, 3H, H-14), 1.64 (d, *J* = 6.7 Hz, 3H, H-4) ppm.

^{13}C NMR (125 MHz, $CDCl_3$, 298 K): δ 161.4 (C-5), 149.1 (C-6), 144.1 (C-10), 140.4 (C-2), 138.9 (C-8), 128.2 (C-12), 127.7 (C-11), 127.4 (C-13), 124.7 (C-3), 124.3 (C-9), 119.3 (C-7), 82.7 (C-1), 14.0 (C-14), 13.8 (C-4) ppm.

HRMS (ESI) m/z: $[M+H]^+$ calcd for $C_{16}H_{16}{}^{79}BrNONa^+$ 340.0307, found 340.0303.

IR (thin film): ṽ 3438 (br), 1447 (s), 1362 (s), 1172 (s), 1008 (s), 761 (s) cm^{-1}.

Specific rotation: $[\alpha]_D^{23}$ = +33.2 (*c* 0.5, CH_2Cl_2).

The **enantiomeric ratio** of **3ip** was determined by SFC analysis (CHIRALPACK AD (25 cm), column temperature 25 °C, solvent CO_2/MeOH (with 0.5% Et_3N) = 97/3, flow rate = 2.0 mL/min): t_R = 12.6 min (major), t_R = 14.5 min (minor).

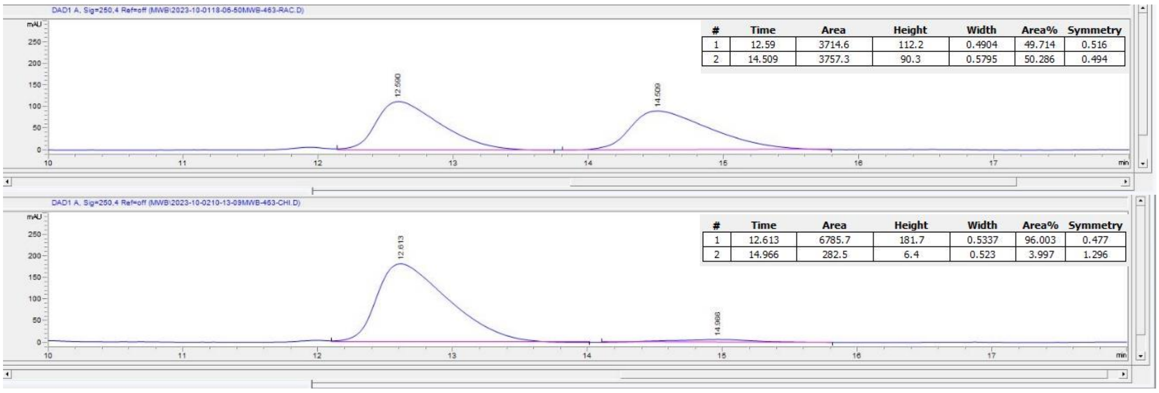

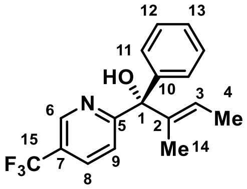

C17H16F3NO
M = 307.32

(*S*,*E*)-2-Methyl-1-phenyl-1-(5-(trifluoromethyl)pyridin-2-yl)but-2-en-1-ol (**3jp**, **GP 6**) was prepared from phenyl(5-(trifluoromethyl)pyridin-2-yl)methanol (**1j**, 25.3 mg, 0.10 mmol, 100 mol%) and but-2-yne (**2p**, 58.7 µL, 40.6 mg, 0.75 mmol, 750 mol%), using [Ir(cod)$_2$]BARF (6.36 mg, 5.0 µmol, 5.0 mol%), **L1** (3.11 mg, 5.0 µmol, 5.0 mol%) and *i*-PrOH (19.1 µL, 15.0 mg, 0.25 mmol, 250 mol%) in toluene (0.2 mL, 0.5 M) at 110 °C for 48 h. Purification by flash column chromatography on silica gel (hexane/ethyl acetate from 100/1 to 19/1) afforded **3jp** (17.7 mg, 58% yield, 98% ee) as a colorless oil.

R*f* = 0.6 (Hexane/EtOAc = 15/1).

^{1}H NMR (500 MHz, CDCl$_3$, 298 K): δ 8.85 (s, 1H, H-6), 7.86 (dd, *J* = 8.4, 2.3 Hz, 1H, H-8), 7.43–7.38 (m, 2H, H-11), 7.36–7.30 (m, 3H, H-9 and H-12), 7.28–7.26 (m, 1H, H-13), 5.53 (s, 1H, OH), 4.97 (q, *J* = 6.7 Hz, 1H, H-3), 1.70 (s, 3H, H-14), 1.65 (d, *J* = 6.7 Hz, 3H, H-4) ppm.

^{13}C NMR (125 MHz, CDCl$_3$, 298 K): δ 166.7 (C-5), 145.1 (q, $J_{C–F}$ = 4.2 Hz, C-6), 143.7 (C-10), 140.3 (C-2), 133.5 (q, $J_{C–F}$ = 3.3 Hz, C-8), 128.3 (C-12), 127.8 (C-11), 127.6 (C-13), 125.4 (q, $J_{C–F}$ = 33.8 Hz, C-7), 125.0 (C-3), 122.8 (C-9), 123.6 (q, $J_{C–F}$ = 271 Hz, C-15), 83.0 (C-1), 14.0 (C-14), 13.8 (C-4) ppm.

^{19}F NMR (471 MHz, CDCl$_3$, 298 K): δ –62.6 (s, ArCF$_3$) ppm.

HRMS (ESI) m/z: [M +H]$^+$ calcd for $C_{17}H_{17}F_3NO^+$ 308.1257, found 308.1257.

IR (thin film): ṽ 3435 (br), 1608 (s), 1324 (s), 1127 (s), 1017 (s), 754 (s) cm^{-1}.

Specific rotation: $[\alpha]_D^{27}$ = +34.6 (*c* 0.5, CH_2Cl_2).

The **enantiomeric ratio** of **3jp** was determined by SFC analysis (CHIRALPACK AD (25 cm), column temperature 25 °C, solvent CO_2/MeOH (with 0.5% Et_3N) = 97/3, flow rate = 0.3 mL/min): t_R = 24.4 min (major), t_R = 27.3 min (minor).

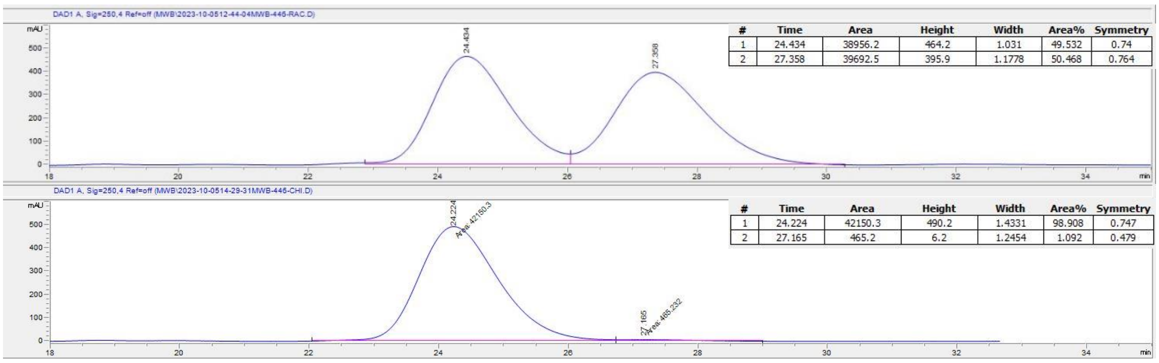

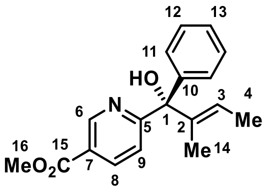

$C_{18}H_{19}NO_3$
M = 297.35

Methyl (*S*,*E*)-6-(1-hydroxy-2-methyl-1-phenylbut-2-en-1-yl)nicotinate (**3kp**, **GP 6**) was prepared from methyl 6-(hydroxy(phenyl)methyl)nicotinate (**1k**, 24.3 mg, 0.10 mmol, 100 mol%) and but-2-yne (**2p**, 58.7 µL, 40.6 mg, 0.75 mmol, 750 mol%), using [Ir(cod)$_2$]BARF (6.36 mg, 5.0 µmol, 5.0 mol%), **L1** (3.11 mg, 5.0 µmol, 5.0 mol%) and *i*-PrOH (19.1 µL, 15.0 mg, 0.25 mmol, 250 mol%) in toluene (0.2 mL, 0.5 M) at 110 °C for 48 h. Purification by flash column chromatography on silica gel (hexane/ethyl acetate from 100/1 to 19/1) afforded **3kp** (25.5 mg, 86% yield, 95% ee) as a colorless oil.

R*f* = 0.2 (Hexane/EtOAc = 15/1).

^{1}H NMR (500 MHz, $CDCl_3$, 298 K): δ 9.16 (d, *J* = 2.1 Hz, 1H, H-6), 8.22 (dd, *J* = 8.3 Hz, *J* = 2.1 Hz, 1H, H-8), 7.42–7.39 (m, 2H, H-11), 7.34–7.30 (m, 2H, H-12), 7.28–7.23 (m, 2H, H-9 and H-13), 5.71 (s, 1H, OH), 4.97 (q, *J* = 6.7 Hz, 1H, H-3), 3.95 (s, 3H, H-16), 1.69 (s, 3H, H-14), 1.63 (d, *J* = 6.7 Hz, 3H, H-4) ppm.

^{13}C NMR (125 MHz, $CDCl_3$, 298 K): δ 166.9 (C-5), 165.6 (C15), 149.3 (C-6), 143.9 (C-10), 140.3 (C-2), 137.4 (C-8), 128.2 (C-12), 127.8 (C-11), 127.5 (C-13), 124.9 (C-3), 124.9 (C-7), 122.6 (C-9), 83.0 (C-1), 52.6 (C-16), 14.0 (C-14), 13.8 (C-4) ppm.

HRMS (ESI) m/z: $[M+H]^+$ calcd for $C_{18}H_{20}NO_3^+$ 298.1438, found 298.1434.

IR (thin film): ṽ 3428 (br), 1726 (s), 1596 (s), 1435 (s), 1374 (s), 1289 (s), 1116 (s), 1023 (s), 757 (s) cm^{-1}.

Specific rotation: $[\alpha]_D^{27}$ = +28.8 (*c* 0.5, CH_2Cl_2).

The **enantiomeric ratio** of **3kp** was determined by SFC analysis (CHIRALPACK SC (25 cm), column temperature 25 °C, solvent CO_2/MeOH (with 0.5% Et_3N) = 95/5, flow rate = 2.0 mL/min): t_R = 7.7 min (minor), t_R = 8.8 min (major).

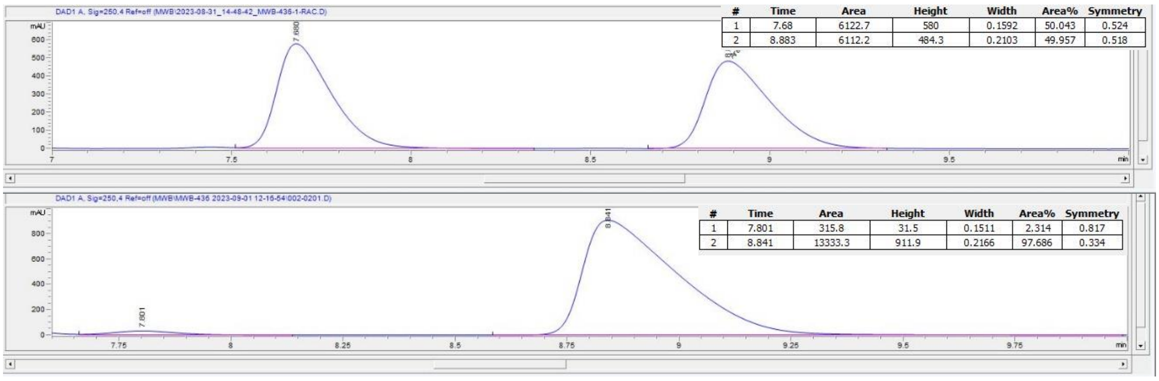

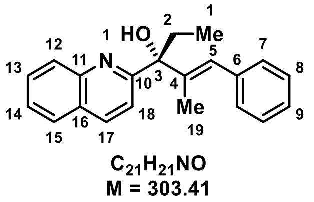

(*R*,*E*)-2-methyl-1-phenyl-3-(quinolin-2-yl)pent-1-en-3-ol (**3la**, **GP 6**) was prepared from 1-(quinolin-2-yl)propan-1-ol (**1l**, 18.7 mg, 0.10 mmol, 100 mol%) and 1-phenyl-1-propyne (**2a**, 31.6 µL, 29.0 mg, 0.25 mmol, 250 mol%), using [Ir(cod)$_2$]BARF (12.7 mg, 10 µmol, 10 mol%), **L1** (6.23 mg, 10 µmol, 10 mol%) and 3,3-dimethylbutan-2-ol (32 µL, 25.5 mg, 0.25 mmol, 250 mol%) in toluene (0.2 mL, 0.5 M) at 110 °C for 24 h. Purification by flash column chromatography on silica gel (hexane/ethyl acetate from 100/1 to 19/1) afforded **3la** (18.6 mg, 61% yield, 98% ee, >25:1 r.r.) as a colorless oil. The regioisomer ratio of the process (11:1 r.r.) was determined from the ^{1}H NMR spectrum of crude material.

R$_f$ = 0.3 (Hexane/EtOAc = 15/1).

^{1}H NMR (500 MHz, $CDCl_3$, 298 K): δ 8.15 (d, *J* = 8.6 Hz, 1H, H-17), 8.13 (d, *J* = 8.4 Hz, 1H, H-12), 7.84 (d, *J* = 8.3 Hz, 1H, H-15), 7.78–7.72 (m, 1H, H-13), 7.59–7.54 (m, 1H, H-14), 7.46 (d, *J* = 8.6 Hz, 1H, H-18), 7.35–7.28 (m, 4H, H-7 and H-8), 7.24–7.19 (m, 1H, H-9), 6.88 (s, 1H, H-5), 6.27 (s, 1H, OH), 2.39–2.30 (m, 1H, H-2), 2.26–2.17 (m, 1H, H-2), 1.67 (s, 3H, H-19), 0.79 (t, *J* = 7.3 Hz, 3H, H-1) ppm.

^{13}C NMR (125 MHz, $CDCl_3$, 298 K): δ 162.5 (C-10), 145.8 (C-11), 142.5 (C-4), 138.3 (C-6), 137.3 (C-17), 129.9 (C-13), 129.3 (C-7), 129.0 (C-12), 128.2 (C-8), 127.6 (C-15), 127.3 (C-16), 126.7 (C-14), 126.5 (C-9), 125.9 (C-5), 118.1 (C-18), 79.2 (C-3), 31.5 (C-2), 14.6 (C-19), 8.0 (C-1) ppm.

HRMS (ESI) m/z: $[M+H]^+$ calcd for $C_{21}H_{22}NO^+$ 304.1696, found 304.1697.

IR (thin film): ṽ 3364 (br), 1599 (s), 1504 (s), 1372 (s), 1126 (s) cm^{-1}.

Specific rotation: $[\alpha]_D^{24}$ = −200.4 (*c* 0.5, CH_2Cl_2).

The **enantiomeric ratio** of **3la** was determined by SFC analysis (CHIRALPACK IE (25 cm), column temperature 25 °C, solvent CO_2/MeOH (with 0.5% Et_3N) = 90/10, flow rate = 2.0 mL/min): t_R = 9.2 min (minor), t_R = 10.2 min (major).

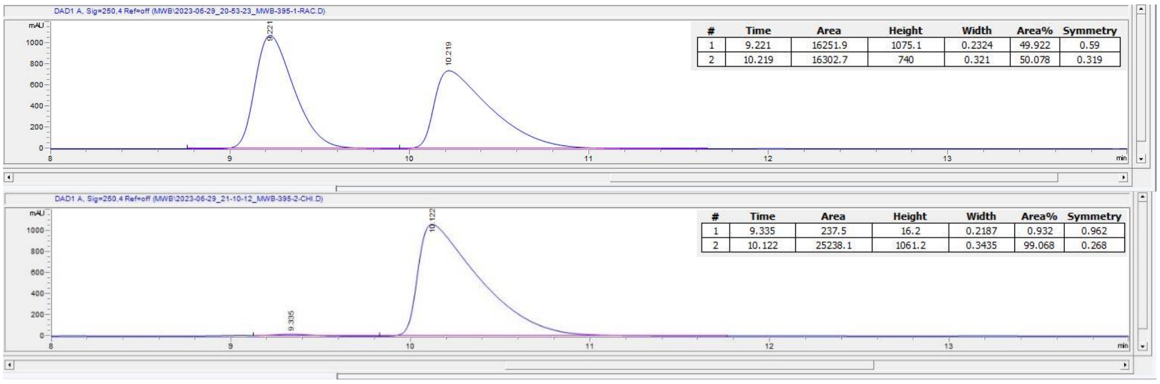

Characteristic signals for regioisomer *iso*-**3la**

¹ H NMR (500 MHz, $CDCl_3$, 298 K): 6.07 (q, *J* = 6.8 Hz, 1H), 1.43 (d, *J* = 6.9 Hz, 3H).

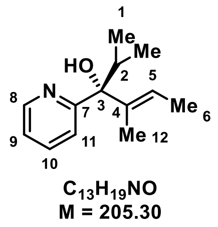

(*R*,*E*)-2,4-Dimethyl-3-(pyridin-2-yl)hex-4-en-3-ol (**3mp**, **GP 6**) was prepared from 2-methyl-1-(pyridin-2-yl)propan-1-ol (**1m**, 15.1 mg, 0.10 mmol, 100 mol%) and but-2-yne (**2p**, 58.7 μL, 40.6 mg, 0.75 mmol, 750 mol%), using $[Ir(cod)_2]$BARF (6.36 mg, 5.0 μmol, 5.0 mol%), **L1** (3.11 mg, 5.0 μmol, 5.0 mol%) and *i*-PrOH (19.1 μL, 15.0 mg, 0.25 mmol, 250 mol%) in toluene (0.2 mL, 0.5 M) at 110 °C for 48 h. Purification by flash column chromatography on silica gel (hexane/ethyl acetate from 100/1 to 19/1) afforded **3mp** (15.2 mg, 74% yield, >99% ee) as a yellow oil.

$\mathbf{R_f}$ = 0.4 (Hexane/EtOAc = 15/1).

¹H NMR (500 MHz, $CDCl_3$, 298 K): δ 8.49 (d, *J* = 5.1 Hz, 1H, H-8), 7.68–7.63 (m, 1H, H-10), 7.34 (d, *J* = 8.1 Hz, 1H, H-11), 7.18–7.14 (m, 1H, H-9), 5.89 (q, *J* = 6.7 Hz, 1H, H-5), 5.46 (s,

1H, OH), 2.57–2.48 (m, 1H, H-2), 1.63 (d, J = 6.7 Hz, 3H, H-6), 1.50 (s, 3H, H-12), 1.00 (d, J = 6.7 Hz, 3H, H-1), 0.60 (d, J = 6.7 Hz, 3H, H-1) ppm.

^{13}C NMR (125 MHz, $CDCl_3$, 298 K): δ 163.1 (C-7), 147.1 (C-8), 139.0 (C-4), 136.6 (C-10), 121.8 (C-9), 120.4 (C-11), 119.5 (C-5), 80.9 (C-3), 33.1 (C-2), 17.3 (C-1), 17.1 (C-1), 13.7 (C-6), 12.7 (C-12) ppm.

HRMS (ESI) m/z: $[M+H]^+$ calcd for $C_{13}H_{20}NO^+$ 206.1540, found 206.1537.

IR (thin film): ṽ 3374 (br), 2968 (s), 1592 (s), 1432 (s), 1384 (s), 1005 (s), 772 (s) cm^{-1}.

Specific rotation: $[\alpha]_D^{22}$ = –39.9 (c 0.5, CH_2Cl_2).

The **enantiomeric ratio** of **3mp** was determined by SFC analysis (CHIRALPACK IE (25 cm), column temperature 25 °C, solvent CO_2/MeOH (with 0.5% Et_3N) = 97/3, flow rate = 2.0 mL/min): t_R = 4.3 min (major), t_R = 5.2 min (minor).

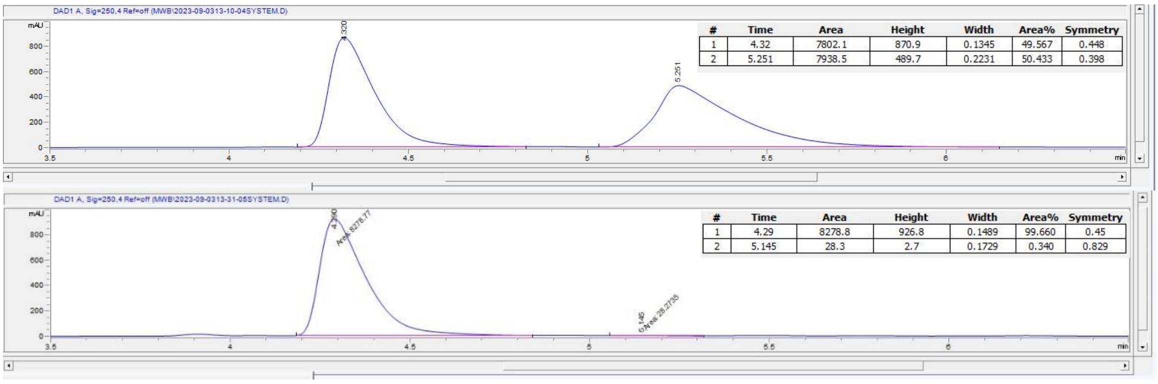

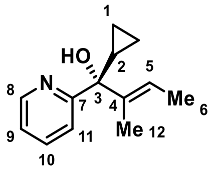

$C_{13}H_{17}NO$
M = 203.29

(*R*,*E*)-1-Cyclopropyl-2-methyl-1-(pyridin-2-yl)but-2-en-1-ol (**3np**, **GP 6**) was prepared from cyclopropyl(pyridin-2-yl)methanol (**1n**, 14.9 mg, 0.10 mmol, 100 mol%) and but-2-yne (**2p**, 58.7 µL, 40.6 mg, 0.75 mmol, 750 mol%), using $[Ir(cod)_2]$BARF (12.7 mg, 10 µmol, 10 mol%), **L1** (6.23 mg, 10 µmol, 10 mol%) and *i*-PrOH (19.1 µL, 15.0 mg, 0.25 mmol, 250 mol%) in toluene (0.2 mL, 0.5 M) at 110 °C for 48 h. Purification by flash column chromatography on silica gel (hexane/ethyl acetate from 100/1 to 19/1) afforded **3np** (5.90 mg, 29% yield, 96% ee) as a yellow oil.

R_f = 0.5 (Hexane/EtOAc = 15/1).

^{1}H NMR (500 MHz, $CDCl_3$, 298 K): δ 8.48 (d, *J* = 4.9 Hz, 1H, H-8), 7.69–7.64 (m, 1H, H-10), 7.29 (d, *J* = 8.0 Hz, 1H, H-11), 7.21–7.16 (m, 1H, H-9), 6.13 (q, *J* = 6.7 Hz, 1H, H-5), 5.33 (s, 1H, OH), 1.71 (d, *J* = 6.7 Hz, 3H, H-6), 1.40 (s, 3H, H-12), 1.31–1.24 (m, 1H, H-2), 0.61–0.55 (m, 1H, H-1), 0.55–0.49 (m, 1H, H-1), 0.34–0.28 (m, 1H, H-1), 0.18–0.11 (m, 1H, H-1) ppm.

^{13}C NMR (125 MHz, $CDCl_3$, 298 K): δ 163.9 (C-7), 147.0 (C-8), 139.7 (C-4), 136.9 (C-10), 122.0 (C-5), 121.7 (C-9), 120.5 (C-11), 76.5 (C-3), 19.4 (C-2), 13.8 (C-6), 13.0 (C-12), 2.2 (C-1), 0.1 (C-1) ppm.

HRMS (ESI) m/z: $[M+H]^+$ calcd for $C_{13}H_{18}NO^+$ 204.1383, found 204.1384.

IR (thin film): ṽ 3371 (br), 1593 (s), 1432 (s), 1377 (s), 1011 (s), 771 (s) cm^{-1}.

Specific rotation: $[\alpha]_D^{28}$ = –91.8 (*c* 0.23, CH_2Cl_2).

The **enantiomeric ratio** of **3np** was determined by SFC analysis (CHIRALPACK AD (25 cm), column temperature 25 °C, solvent CO_2/MeOH (with 0.5% Et_3N) = 97/3, flow rate = 2.0 mL/min): t_R = 5.1 min (minor), t_R = 5.6 min (major).

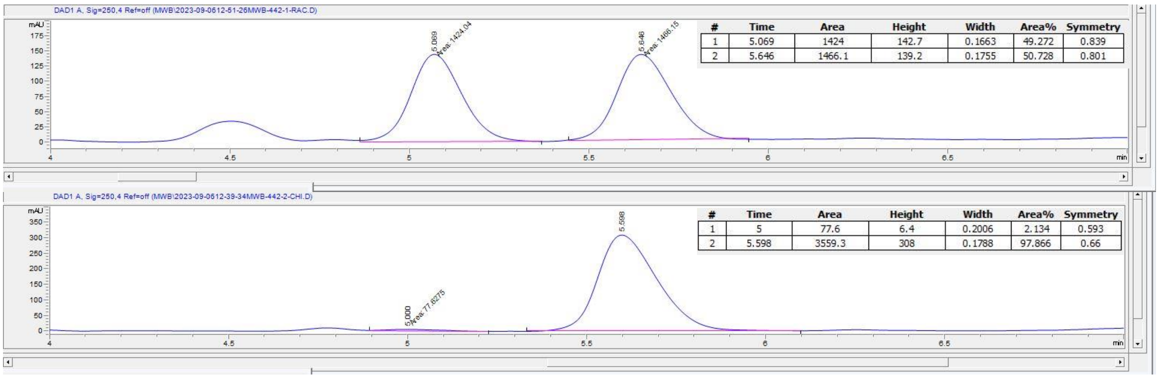

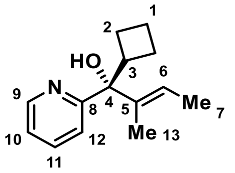

$C_{14}H_{19}NO$
M = 217.31

(*R*,*E*)-1-Cyclobutyl-2-methyl-1-(pyridin-2-yl)but-2-en-1-ol (**3op**, **GP 6**) was prepared from cyclobutyl(pyridin-2-yl)methanol (**1o**, 16.3 mg, 0.10 mmol, 100 mol%) and but-2-yne (**2p**, 58.7 µL, 40.6 mg, 0.75 mmol, 750 mol%), using $[Ir(cod)_2]$BARF (12.7 mg, 10 µmol, 10 mol%), **L1** (6.23 mg, 10 µmol, 10 mol%) and *i*-PrOH (19.1 µL, 15.0 mg, 0.25 mmol, 250 mol%) in toluene (0.2 mL, 0.5 M) at 110 °C for 48 h. Purification by flash column chromatography on silica gel (hexane/ethyl acetate from 100/1 to 19/1) afforded **3op** (14.4 mg, 66%, 99% *ee*) as a colorless solid.

R$_f$ = 0.5 (Hexane/EtOAc = 15/1).

M.P. 43–44 °C (Hexane/EtOAc).

^{1}H NMR (500 MHz, $CDCl_3$, 298 K): δ 8.48 (d, *J* = 4.8 Hz, 1H, H-9), 7.64–7.60 (m, 1H, H-11), 7.21 (d, *J* = 8.0 Hz, 1H, H-12), 7.17–7.13 (m, 1H, H-10), 5.42 (q, *J* = 6.7 Hz, 1H, H-6), 5.64 (s, 1H, OH), 3.11–3.03 (m, 1H, H-3), 2.25–2.16 (m, 1H, H-2), 2.03–1.95 (m, 1H, H-2), 1.84–1.74 (m, 2H, H-1 and H-2), 1.74–1.68 (m, 1H, H-1), 1.64 (d, *J* = 6.7 Hz, 3H, H-7), 1.35 (s, 3H, H-13), 1.32–1.26 (m, 1H, H-2) ppm.

^{13}C NMR (125 MHz, $CDCl_3$, 298 K): δ 162.4 (C-8), 146.9 (C-9), 138.4 (C-5), 136.8 (C-11), 121.9 (C-10), 120.3 (C-6 and C-12), 78.9 (C-4), 42.6 (C-3), 23.8 (C-2), 21.6 (C-2), 17.8 (C-1), 13.8 (C-7), 12.7 (C-13) ppm.

HRMS (ESI) m/z: $[M–H_2O+H]^+$ calcd for $C_{14}H_{18}N^+$ 200.1434, found 200.1429.

IR (thin film): ṽ 3378 (br), 2927 (s), 1593 (s), 1432 (s), 1376 (s), 768 (s) cm^{-1}.

Specific rotation: $[\alpha]_D^{24}$ = –108.3 (*c* 0.5, CH_2Cl_2).

The **enantiomeric ratio** of **3op** was determined by SFC analysis (CHIRALPACK AD (25 cm), column temperature 25 °C, solvent CO_2/MeOH (with 0.5% Et_3N) = 97/3, flow rate = 2.0 mL/min): t_R = 6.6 min (minor), t_R = 8.0 min (major).

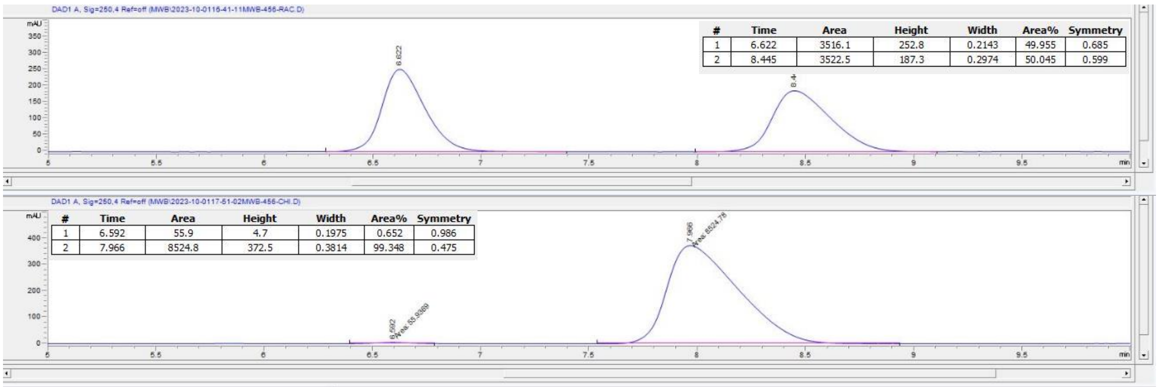

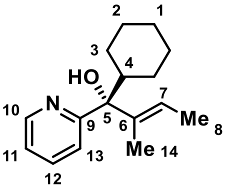

$C_{16}H_{23}NO$
M = 245.37

(*R*,*E*)-1-Cyclohexyl-2-methyl-1-(pyridin-2-yl)but-2-en-1-ol (**3pp**, **GP 6**) was prepared from cyclohexyl(pyridin-2-yl)methanol (**1p**, 19.1 mg, 0.10 mmol, 100 mol%) and but-2-yne (**2p**, 58.7

μL, 40.6 mg, 0.75 mmol, 750 mol%), using [Ir(cod)$_2$]BARF (12.7 mg, 10 μmol, 10 mol%), **L1** (6.23 mg, 10 μmol, 10 mol%) and *i*-PrOH (19.1 μL, 15.0 mg, 0.25 mmol, 250 mol%) in toluene (0.2 mL, 0.5 M) at 110 °C for 48 h. Purification by flash column chromatography on silica gel (hexane/ethyl acetate from 100/1 to 19/1) afforded **3pp** (17.4 mg, 71% yield, 97% ee) as a colorless oil.

R*f* = 0.5 (Hexane/EtOAc = 15/1).

^{1}H NMR (500 MHz, $CDCl_3$, 298 K): δ 8.48 (d, *J* = 5.0 Hz, 1H, H-10), 7.68–7.63 (m, 1H, H-12), 7.34 (d, *J* = 8.0 Hz, 1H, H-13), 7.17–7.13 (m, 1H, H-11), 5.87 (q, *J* = 6.7 Hz, 1H, H-7), 5.47 (s, 1H, OH), 2.15–2.07 (m, 1H, H-4), 1.84–1.74 (m, 2H, CyH), 1.66–1.59 (m, 2H, CyH), 1.62 (d, *J* = 6.7 Hz, 3H, H-8), 1.49 (s, 3H, H-14), 1.30–1.22 (m, 2H, H-3), 1.19–1.08 (m, 3H, CyH and H-3), 0.96–0.88 (m, 1H, CyH) ppm.

^{13}C NMR (125 MHz, $CDCl_3$, 298 K): δ 162.9 (C-9), 147.2 (C-10), 138.5 (C-6), 136.5 (C-12), 121.7 (C-11), 120.4 (C-13), 119.4 (C-7), 80.8 (C-5), 43.5 (C-4), 27.2 (Cy), 27.0 (Cy), 27.0 (Cy), 26.9 (Cy), 26.7 (Cy), 13.7 (C-8), 12.7 (C-14) ppm.

HRMS (ESI) m/z: $[M+H]^+$ calcd for $C_{16}H_{24}NO^+$ 246.1853, found 246.1844.

IR (thin film): ṽ 3370 (br), 2929 (s), 2851 (s), 1591 (s), 1432 (s), 1382 (s), 773 (s) cm^{-1}.

Specific rotation: $[\alpha]_D^{25}$ = –21.2 (*c* 0.5, CH_2Cl_2).

The **enantiomeric ratio** of **3pp** was determined by SFC analysis (CHIRALPACK AD (25 cm), column temperature 25 °C, solvent CO_2/MeOH (with 0.5% Et_3N) = 97/3, flow rate = 2.0 mL/min): t_R = 6.3 min (major), t_R = 7.0 min (minor).

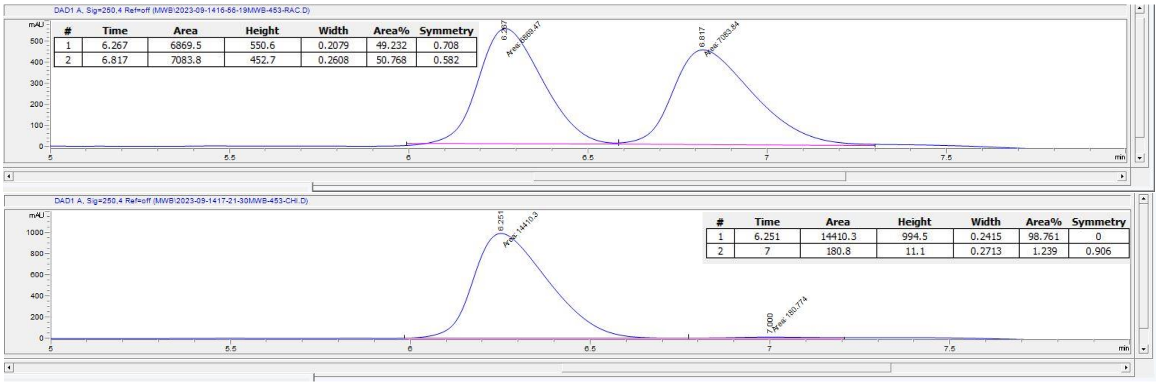

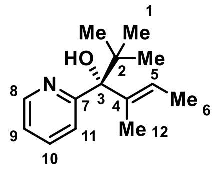

$C_{14}H_{21}NO$
M = 219.33

(*R*,*E*)-2,2,4-trimethyl-3-(pyridin-2-yl)hex-4-en-3-ol (**2qp**, **GP 6**) was prepared from 2,2-dimethyl-1-(pyridin-2-yl)propan-1-ol (**1q**, 16.5 mg, 0.10 mmol, 100 mol%) and but-2-yne (**2p**, 58.7 μL, 40.6 mg, 0.75 mmol, 750 mol%), using [Ir(cod)$_2$]BARF (12.7 mg, 10 μmol, 10 mol%), **L1** (6.23 mg, 10 μmol, 10 mol%) and *i*-PrOH (19.1 μL, 15.0 mg, 0.25 mmol, 250 mol%) in toluene (0.2 mL, 0.5 M) at 110 °C for 48 h. Purification by flash column chromatography on silica gel (hexane/ethyl acetate from 100/1 to 19/1) afforded **3qp** (4.9 mg, 22% yield, 95% ee) as a colorless oil.

R*f* = 0.5 (Hexane/EtOAc = 15/1).

^{1}H NMR (500 MHz, $CDCl_3$, 298 K): δ 8.52 (d, *J* = 4.9 Hz, 1H, H-8), 7.66–7.61 (m, 1H, H-10), 7.44 (d, *J* = 8.0 Hz, 1H, H-11), 7.20–7.16 (m, 1H, H-9), 6.04 (q, *J* = 6.7 Hz, 1H, H-5), 5.94 (s, 1H, OH), 1.66 (d, *J* = 6.7 Hz, 3H, H-6), 1.41 (s, 3H, H-12), 1.05 (s, 9H, H-1) ppm.

^{13}C NMR (125 MHz, $CDCl_3$, 298 K): δ 162.0 (C-7), 146.6 (C-8), 137.9 (C-4), 135.6 (C-10), 123.0 (C-11), 122.4 (C-5), 121.9 (C-9), 82.6 (C-3), 39.4 (C-2), 27.8 (C-1), 15.2 (C-12), 14.0 (C-6) ppm.

HRMS (ESI) m/z: $[M+H]^+$ calcd for $C_{14}H_{22}NO^+$ 220.1696, found 220.1691.

IR (thin film): ṽ 3372 (br), 2958 (s), 1592 (s), 1431 (s), 1360 (s), 1000 (s), 770 (s) cm^{-1}.

Specific rotation: $[\alpha]_D^{25}$ = –60.0 (*c* 0.25, CH_2Cl_2).

The **enantiomeric ratio** of **3qp** was determined by SFC analysis (CHIRALPACK AD (25 cm), column temperature 25 °C, solvent CO_2/MeOH (with 0.5% Et_3N) = 97/3, flow rate = 1.0 mL/min): t_R = 9.9 min (major), t_R = 10.6 min (minor).

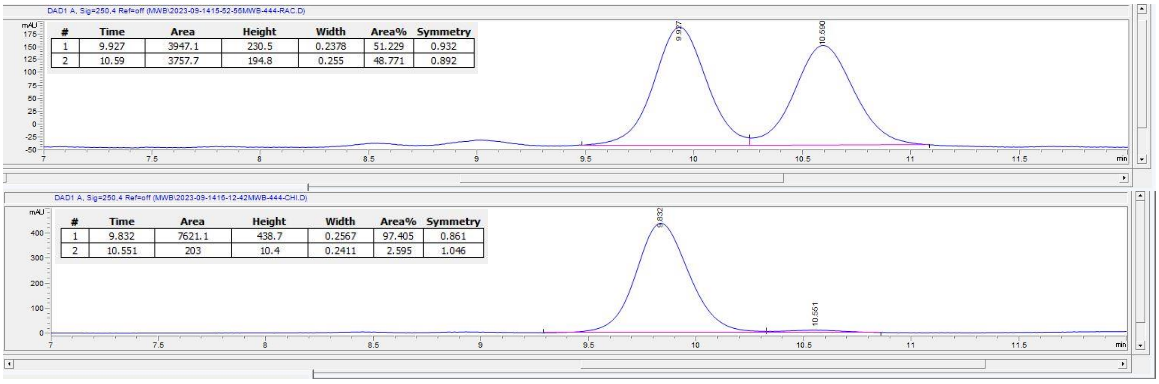

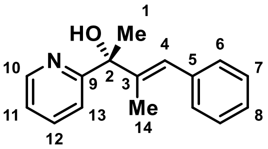

$C_{16}H_{17}NO$
M = 239.32

(*R*,*E*)-3-Methyl-4-phenyl-2-(pyridin-2-yl)but-3-en-2-ol (**3ra**, **GP 6**) was prepared from 1-(pyridin-2-yl)ethan-1-ol (**1r**, 12.3 mg, 0.10 mmol, 100 mol%) and 1-phenyl-1-propyne (**2a**, 31.6 µL, 29.0 mg, 0.25 mmol, 250 mol%), using [Ir(cod)$_2$]BARF (12.7 mg, 10 µmol, 10 mol%), **L6** (3.34 mg, 10 µmol, 10 mol%) and *i*-PrOH (19.1 µL, 15.0 mg, 0.25 mmol, 250 mol%) in toluene (0.2 mL, 0.5 M) at 110 °C for 24 h. Purification by flash column chromatography on silica gel (hexane/ethyl acetate from 100/1 to 9/1) afforded **3ra** (16.6 mg, 69% yield, 90% ee, >25:1 r.r.) as a brown oil. The regioisomer ratio of the process (6:1 r.r.) was determined from the ^{1}H NMR spectrum of crude material.

R*f* = 0.2 (Hexane/EtOAc = 9/1).

^{1}H NMR (500 MHz, CDCl$_3$, 298 K): δ 8.55 (d, *J* = 4.6 Hz, 1H, H-10), 7.72–7.67 (m, 1H, H-12), 7.38 (d, *J* = 8.0 Hz, 1H, H-13), 7.35–7.31 (m, 2H, H-7), 7.31–7.27 (m, 2H, H-6), 7.24–7.18 (m, 2H, H-8 and H-11), 6.79 (s, 1H, H-4), 5.72 (s, 1H, OH), 1.76 (s, 3H, H-1), 1.67 (s, 3H, H-14) ppm.

^{13}C NMR (125 MHz, CDCl$_3$, 298 K): δ 163.8 (C-9), 147.4 (C-10), 142.9 (C-3), 138.2 (C-5), 137.1 (C-12), 129.2 (C-6), 128.2 (C-7), 126.5 (C-8), 125.5 (C-4), 122.2 (C-11), 120.1 (C-13), 76.9 (C-2), 27.9 (C-1), 14.5 (C-14) ppm.

HRMS (ESI) m/z: $[M+H]^+$ calcd for $C_{16}H_{18}NO^+$ 240.1382, found 240.1375.

IR (thin film): ṽ 3378 (br), 2978 (s), 2935 (s), 1591 (s), 1431 (s), 1366 (s), 1122 (s), 1071 (s) cm^{-1}.

Specific rotation: $[\alpha]_D^{25}$ = −62.3 (*c* 0.5, CH_2Cl_2).

The **enantiomeric ratio** of **3ra** was determined by SFC analysis (CHIRALPACK SC (25 cm), column temperature 25 °C, solvent CO_2/MeOH (with 0.5% Et_3N) = 97/3, flow rate = 2.0 mL/min): t_R = 8.9 min (minor), t_R = 9.6 min (major).

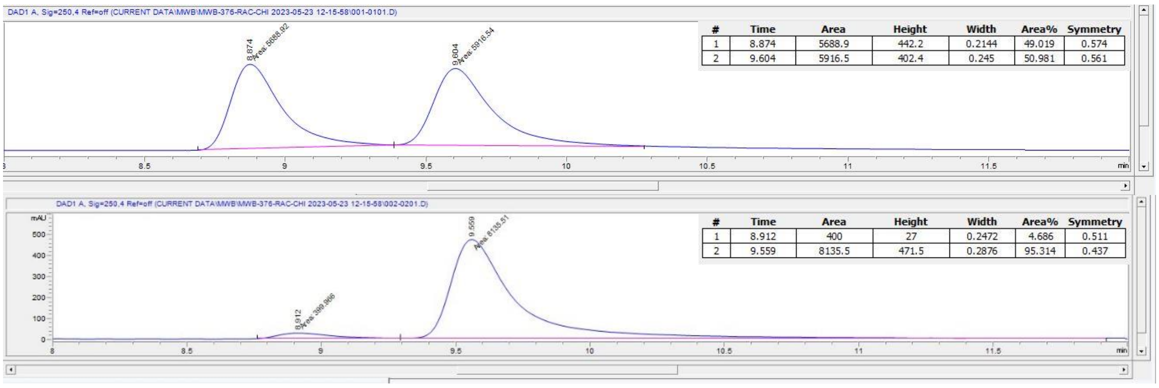

Characteristic signals for regioisomer *iso*-**3ra**

^{1}H NMR (500 MHz, $CDCl_3$, 298 K): 6.02 (q, *J* = 6.8 Hz, 1H), 1.42 (d, *J* = 6.8 Hz, 3H).

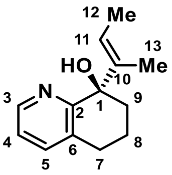

$C_{13}H_{17}NO$
M = 203.29

(*R*,*E*)-8-(but-2-en-2-yl)-5,6,7,8-tetrahydroquinolin-8-ol (**3sp**, **GP 6**) was prepared from 1,2,3,4-tetrahydronaphthalen-1-ol (**1s**, 14.8 mg, 0.10 mmol, 100 mol%) and but-2-yne (**2p**, 58.7 µL, 40.6 mg, 0.75 mmol, 750 mol%), using [Ir(cod)$_2$]BARF (12.7 mg, 10 µmol, 10 mol%), **L1** (6.23 mg, 10 µmol, 10 mol%) and *i*-PrOH (19.1 µL, 15.0 mg, 0.25 mmol, 250 mol%) in toluene (0.2 mL, 0.5 M) at 110 °C for 48 h. Purification by flash column chromatography on silica gel (hexane/acetone from 100/1 to 9/1) afforded **3sp** (12.2 mg, 60% yield, 97% ee) as a colorless oil.

R_f = 0.4 (Hexane/Acetone = 10/1).

^{1}H NMR (500 MHz, $CDCl_3$, 298 K): δ 8.43 (d, *J* = 4.7 Hz, 1H, H-3), 7.40 (d, *J* = 7.7 Hz, 1H, H-5), 7.10 (dd, *J* = 7.7, 4.7 Hz, 1H, H-4), 4.71 (q, *J* = 6.7 Hz, 1H, H-11), 3.82 (s, 1H, OH), 2.81–2.76 (m, 2H, H-7), 2.23–2.16 (m, 1H, H-9), 1.87–1.81 (m, 1H, H-8), 1.81–1.76 (m, 1H, H-9), 1.76–1.69 (m, 1H, H-8), 1.69 (s, 3H, H-13), 1.56 (d, *J* = 6.7 Hz, 3H, H-12) ppm.

^{13}C NMR (125 MHz, $CDCl_3$, 298 K): δ 160.0 (C-2), 147.1 (C-3), 141.8 (C-10), 136.7 (C-5), 132.6 (C-6), 122.3 (C-11), 122.2 (C-4), 76.9 (C-1), 33.6 (C-9), 28.5 (C-7), 18.9 (C-8), 13.7 (C-12), 12.4 (C-13) ppm.

HRMS (ESI) m/z: $[M+H]^+$ calcd for $C_{13}H_{18}NO^+$ 204.1383, found 204.1376.

IR (thin film): ṽ 3422 (br), 2937 (s), 1575 (s), 1443 (s), 1363 (s), 1088 (s), 1039 (s), 785 (s) cm^{-1}.

SPecific rotation: $[\alpha]_D^{24}$ = +60.2 (*c* 0.5, CH_2Cl_2).

The **enantiomeric ratio** of **3sp** was determined by SFC analysis (CHIRALPACK SC (25 cm), column temperature 25 °C, solvent CO_2/MeOH (with 0.5% Et_3N) = 95/5, flow rate = 2.0 mL/min): t_R = 5.6 min (minor), t_R = 6.5 min (major).

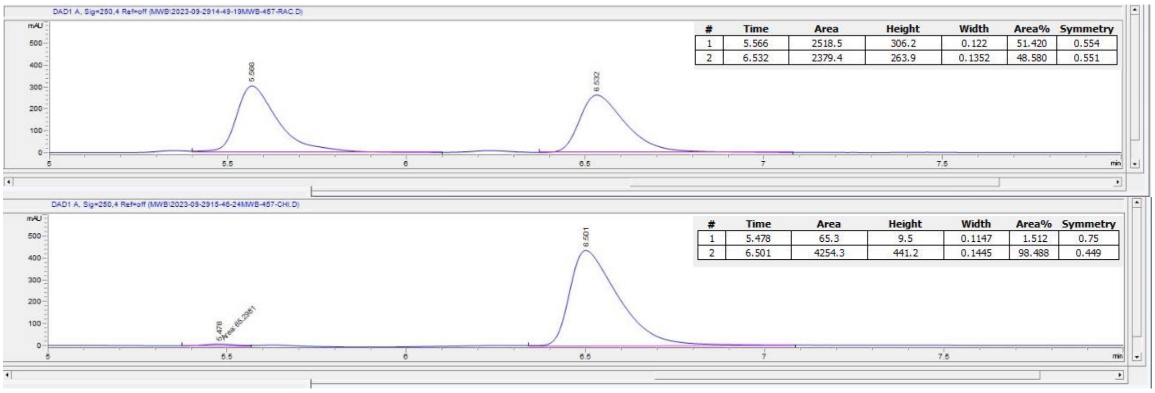

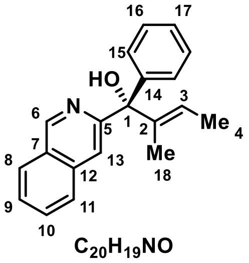

$C_{20}H_{19}NO$
M = 289.38

(*S*,*E*)-1-(isoquinolin-3-yl)-2-methyl-1-phenylbut-2-en-1-ol (**3tp**, **GP 6**) was prepared from isoquinolin-3-yl(phenyl)methanol (**1t**, 23.5 mg, 0.10 mmol, 100 mol%) and but-2-yne (**2p**, 58.7 µL, 40.6 mg, 0.75 mmol, 750 mol%), using $[Ir(cod)_2]$BARF (12.7 mg, 10 µmol, 10 mol%), **L1** (6.23 mg, 10 µmol, 10 mol%) and *i*-PrOH (19.1 µL, 15.0 mg, 0.25 mmol, 250 mol%) in toluene (0.2 mL, 0.5 M) at 110 °C for 48 h. Purification by flash column chromatography on silica gel (hexane/ethyl acetate from 100/1 to 19/1) afforded **3tp** (19.0 mg, 66% yield, 96% ee) as a colorless solid.

$\mathbf{R_f}$ = 0.5 (Hexane/EtOAc = 15/1).

M.P. 107–108 °C (Hexane/EtOAc).

^{1}H NMR (500 MHz, $CDCl_3$, 298 K): δ 9.24 (s, 1H, H-6), 7.98 (d, *J* = 8.1 Hz, 1H, H-8), 7.76 (d, *J* = 8.3 Hz, 1H, H-11), 7.71–7.66 (m, 1H, H-10), 7.62–7.57 (m, 1H, H-9), 7.48 (d, *J* = 7.3 Hz,

2H, H-15), 7.41 (s, 1H, H-13), 7.37–7.31 (m, 2H, H-16), 7.30–7.25 (m, 1H, H-17), 5.65 (s, 1H, OH), 4.99 (q, J = 6.7 Hz, 1H, H-3), 1.76 (s, 3H, H-18), 1.65 (d, J = 6.7 Hz, 3H, H-4) ppm.

^{13}C NMR (125 MHz, $CDCl_3$, 298 K): δ 156.2 (C-5), 151.3 (C-6), 144.8 (C-14), 140.9 (C-2), 136.1 (C-12), 130.9 (C-10), 128.0 (C-16), 128.0 (C-15), 127.7 (C-8), 127.7 (C-7), 127.5 (C-9), 127.2 (C-17), 127.2 (C-11), 124.6 (C-3), 118.9 (C-13), 83.1 (C-1), 14.1 (C-18), 13.9 (C-4) ppm.

HRMS (ESI) m/z: $[M–H_2O+H]^+$ calcd for $C_{20}H_{18}N^+$ 272.1434, found 272.1430.

IR (thin film): ṽ 3407 (br), 1588 (s), 1443 (s), 1368 (s), 956 (s), 752 (s) cm^{-1}.

Specific rotation: $[\alpha]_D^{22}$ = –90.2 (c 0.5, CH_2Cl_2).

The **enantiomeric ratio** of **3tp** was determined by SFC analysis (CHIRALPACK IC (25 cm), column temperature 25 °C, solvent CO_2/MeOH (with 0.5% Et_3N) = 95/5, flow rate = 2.0 mL/min): t_R = 10.5 min (minor), t_R = 11.4 min (major).

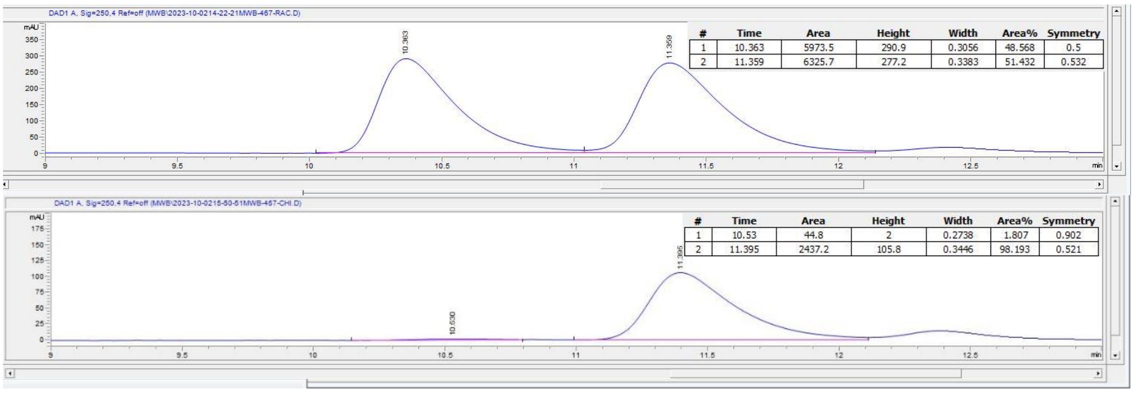

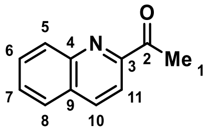

$C_{11}H_9NO$
M = 171.20

1-(Quinolin-2-yl)ethan-1-one

R$_f$ = 0.45 (Hexane/ethyl acetate = 15/1).

^{1}H NMR (500 MHz, $CDCl_3$, 298 K): δ 8.24 (d, J = 8.5 Hz, 1H, H-10), 8.19 (d, J = 8.5 Hz, 1H, H-5), 8.11 (d, J = 8.5 Hz, 1H, H-11), 7.85 (d, J = 8.2 Hz, 1H, H-8), 7.80–7.75 (m, 1H, H-6), 7.66–7.61 (m, 1H, H-7), 2.86 (s, 3H, H-1) ppm.

^{13}C NMR (125 MHz, $CDCl_3$, 298 K): δ 200.8 (C-2), 153.3 (C-3), 147.3 (C-4), 137.0 (C-10), 130.7 (C-5), 130.1 (C-6), 129.7 (C-9), 128.7 (C-7), 127.8 (C-8), 118.1 (C-11), 25.7 (C-1) ppm.

4 Iridium-Catalyzed Cross-Coupling of a Primary Alcohol with Styrene

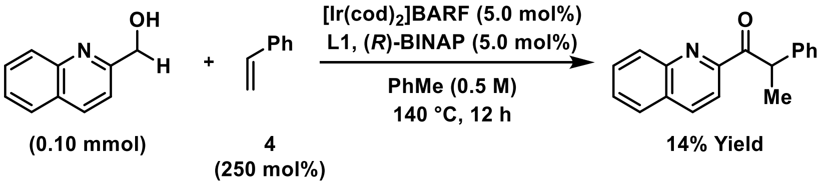

Scheme S12. Iridium-catalyzed cross coupling of primary alcohol and styrene.

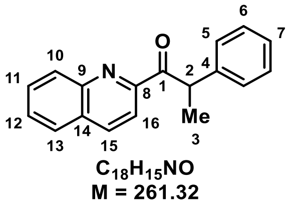

2-Phenyl-1-(quinolin-2-yl)propan-1-one (**GP 6**) was prepared from quinolin-2-ylmethanol (15.9 mg, 0.10 mmol, 100 mol%) and styrene (**4**, 28.7 µL, 26.0 mg, 0.25 mmol, 250 mol%), using [Ir(cod)$_2$]BARF (6.36 mg, 5.0 µmol, 5.0 mol%) and **L1** (3.11 mg, 5.0 µmol, 5.0 mol%) in toluene (0.2 mL, 0.5 M) at 140 °C for 12 h. Purification by flash column chromatography on silica gel (hexane/ethyl acetate from 100/1 to 19/1) afforded the product (3.7 mg, 14% yield) as a yellow solid. Data were in accordance with those reported.[11]

R*f* = 0.3 (Hexane/EtOAc = 15/1).

^{1}H NMR (500 MHz, $CDCl_3$, 298 K): δ 8.24–8.19 (m, 2H, H-10 and H-15), 8.09 (d, *J* = 8.6 Hz, 1H, H-16), 7.83 (d, *J* = 8.1 Hz, 1H, H-13), 7.80–7.75 (m, 1H, H-11), 7.65–7.60 (m, 1H, H-12), 7.49–7.46 (m, 2H, H-5), 7.28–7.23 (m, 2H, H-6), 7.17–7.12 (m, 1H, H-7), 5.77 (q, *J* = 7.1 Hz, 1H, H-2), 1.62 (d, *J* = 7.1 Hz, 3H, H-3) ppm.

^{13}C NMR (125 MHz, $CDCl_3$, 298 K): δ 202.2 (C-1), 152.5 (C-8), 147.2 (C-9), 141.2 (C-4), 137.0 (C-15), 130.8 (C-11), 130.0 (C-10), 129.6 (C-14), 128.7 (C-12 or C-5 or C-6), 128.7 (C-12 or C-5 or C-6), 128.6 (C-12 or C-5 or C-6), 127.7 (C-13), 126.8 (C-7), 119.1 (C-16), 44.9 (C-2), 18.2 (C-3) ppm.

5 Gram Scale Reaction

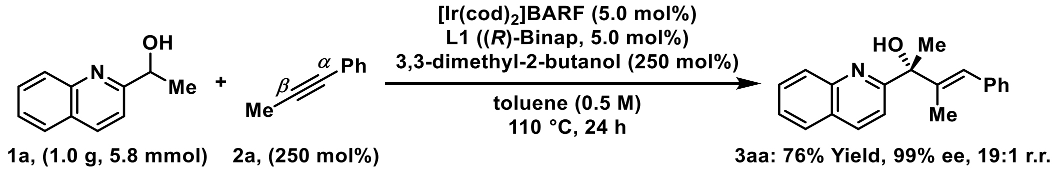

Scheme 13. Gram Scale reaction.

(*R*,*E*)-3-Methyl-4-phenyl-2-(quinolin-2-yl)but-3-en-2-ol (**3aa**, **GP 6**) was prepared from 1-(quinolin-2-yl)ethan-1-ol (**1a**, 1.00 g, 5.8 mmol, 100 mol%), 1-phenyl-1-propyne (**2a**, 1.8 mL, 1.68 g, 14.5 mmol, 250 mol%), using [Ir(cod)$_2$]BARF (369 mg, 0.29 mmol, 5.0 mol%), **L1** (181 mg, 0.29 mmol, 5.0 mol%) and 3,3-dimethylbutan-2-ol (1.8 mL, 1.48 g, 14.5 mmol, 250 mol%) in toluene (12 mL, 0.5 M) at 110 °C for 24 h. After cooling to room temperature, solvent was removed under reduced pressur. Purification by flash column chromatography on silica gel (hexane/ethyl acetate from 100/1 to 19/1) afforded **3aa** (1.28 g, 76% yield, 99% ee, >25:1 r.r.) as a colorless solid. Regiomer ratios (19:1 r.r.) were determined from the ^{1}H NMR spectrums of crude material.

6 Derivatizations of 3aa

6.1 Epoxidation

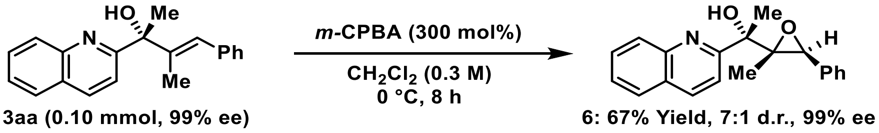

Scheme S13. Epoxidation of **3aa**.

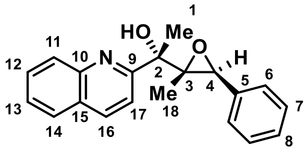

$C_{20}H_{19}NO_2$
M = 305.38

(*S*)-1-((2*R*,3*S*)-2-methyl-3-phenyloxiran-2-yl)-1-(quinolin-2-yl)ethan-1-ol (**6**) was prepared according to a reported procedure.[12] To a round bottom flask equipped with magnetic stirrer were added (*R*,*E*)-3-methyl-4-phenyl-2-(quinolin-2-yl)but-3-en-2-ol (**3aa**, 28.9 mg, 0.10 mmol, 100 mol%) and DCM (3 mL). Then *m*-chloroperbenzoic acid (*m*-CPBA, 70.0 mg, 0.15 mmol, 150 mol%) was added in portions over a period of 7 hour (10.0 mg per hour) at 0 °C in an ice bath. The reaction was stirred at this temperature for another 1 hour. Saturated aqueous $Na_2S_2O_3$ was added to quench the reaction. The reaction mixture was extracted with DCM (3 × 5 mL) and washed by sat. $NaHCO_3$ solution (10 mL). The combined residue was dried through anhydrous Na_2SO_4, filtered and concentrated under reduced pressure. Purification by flash column chromatography on silica gel (hexane/ethyl acetate from 100/1 to 19/1) afforded **6** (major, 20.3 mg, 67% yield, 99% ee, >25:1 d.r.) as a colorless soild. The diastereomer ratio of the process (7:1 d.r.) was determined from the ^{1}H NMR spectrum of crude material.

R*f* = 0.5 (Hexane/EtOAc = 15/1).

M.**P**. 100–101 °C (Hexane/EtOAc).

^{1}H NMR (500 MHz, $CDCl_3$, 298 K): δ 8.24 (d, *J* = 8.6 Hz, 1H, H-16), 8.11 (d, *J* = 8.4 Hz, 1H, H-11), 7.87 (d, *J* = 8.1 Hz, 1H, H-14), 7.78–7.72 (m, 2H, H-12 and H-17), 7.61–7.55 (m, 1H, H-13), 7.37–7.31 (m, 4H, H-6 and H-7), 7.30–7.25 (m, 1H, H-8), 5.95 (s, 1H, OH), 4.52 (s, 1H, H-4), 1.64 (s, 3H, H-1), 0.82 (s, 3H, H-18) ppm.

^{13}C NMR (125 MHz, $CDCl_3$, 298 K): δ 162.7 (C-9), 145.9 (C-10), 137.6 (C-16), 136.2 (C-5), 130.0 (C-12), 129.0 (C-11), 128.2 (C-6), 127.7 (C-7), 127.7 (C-15), 127.6 (C-14), 126.8 (C-8), 126.7 (C-13), 118.7 (C-17), 75.6 (C-2), 67.0 (C-3), 59.7 (C-4), 23.4 (C-1), 13.0 (C-18) ppm.

HRMS (ESI) m/z: $[M+H]^+$ calcd for $C_{20}H_{20}NO_2^+$ 306.1488, found 306.1495.

IR (thin film): ṽ 3364 (br), 1600 (s), 1504 (s), 1133 (s), 1065 (s), 755 (s) cm^{-1}.

Specific rotation: $[\alpha]_D^{19}$ = +17.0 (*c* 0.35, CH_2Cl_2).

The **enantiomeric ratio** of **diastereomers 1** was determined by SFC analysis (CHIRALPACK AD (25 cm), column temperature 25 °C, solvent CO_2/MeOH (with 0.5% Et_3N) = 70/30, flow rate = 2.0 mL/min): t_R = 4.6 min (minor), t_R = 10.0 min (major).

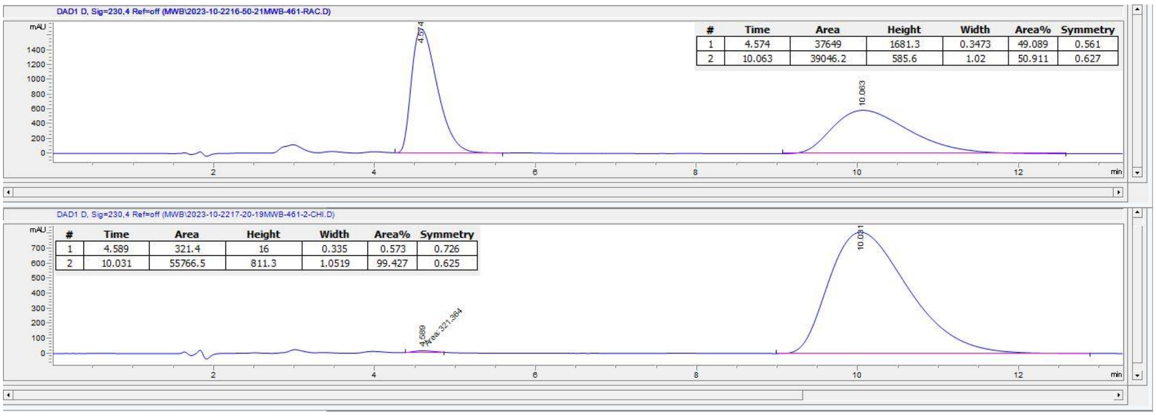

Crystal *racemic* **6** was obtained through recrystallization in the solution of CH_2Cl_2 and methanol at room temperature. The relative configuration of *racemic* **6** was confirmed unambiguously by **X-ray diffraction analysis**. CCDC 2398066 contains the supplementary crystallographic data for this compound.

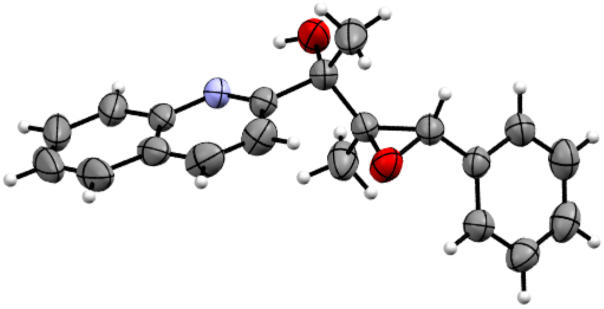

Figure S2. Molecular structure of *racemic* **6**. Thermal ellipsoids represent 50% probability level.

Racemic **(1-(2-methyl-3-phenyloxiran-2-yl)-1-(quinolin-2-yl)ethan-1-ol** was prepared according to the procedure above, using racemic 3-methyl-4-phenyl-2-(quinolin-2-yl)but-3-en-2-ol ((±)-**3aa**, 28.9 mg, 0.10 mmol, 100 mol%) as the starting material.

Characteristic signals for diastereomer

^{1} **H NMR** (500 MHz, $CDCl_3$, 298 K): 3.93 (s, 1H), 1.75 (s, 3H), 1.08 (s, 3H).

6.2 Stereodivergent Synthesis by Stereoselective Quinoline Reduction

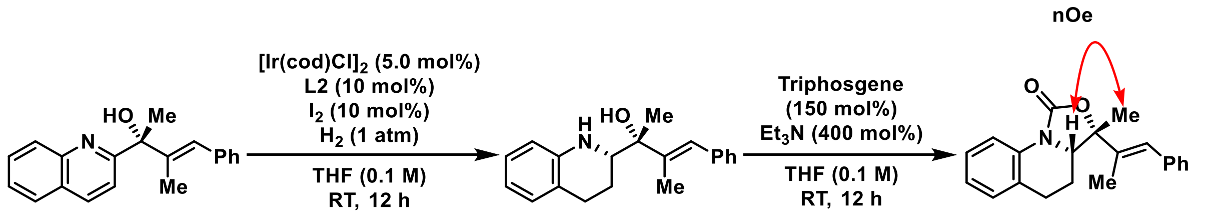

3aa (0.10 mmol, 99% ee) — 7a: 77%, >99% ee >25:1 d.r. — 74%, >99% ee >25:1 d.r.

Scheme S14. Selective reduction of the quinoline unit of **3aa**.

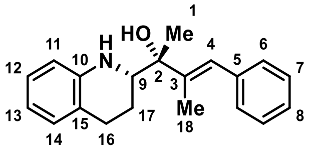

$C_{20}H_{23}NO$
M = 293.41

(*R*,*E*)-3-Methyl-4-phenyl-2-((*S*)-1,2,3,4-tetrahydroquinolin-2-yl)but-3-en-2-ol (**7a**) was prepared according to a reported procedure.[13a] A Schlenk tube was charged with (*R*,*E*)-3-methyl-4-phenyl-2-(quinolin-2-yl)but-3-en-2-ol (**3aa**, 28.9 mg, 0.10 mmol, 100 mol%), $[Ir(cod)Cl]_2$ (3.36 mg, 5.0 µmol, 5.0 mol%), **L2** ((*R*)-SEGPHOS, 6.11 mg, 10 µmol, 10 mol%) and I_2 (2.54 mg, 10 µmol, 10 mol%). The *Schlenk* tube was evacuated and backfilled with H_2 for three times. Then THF (1 mL, 0.1 M) was added. The reaction mixture was stirred at room temperature for 12 hours. Purification by flash column chromatography on silica gel (hexane/ethyl acetate from 100/1 to 19/1) afforded **7a** (22.6 mg, 77% yield, >99% ee, >25:1 d.r.) as a brown oil. The diastereomer ratio of the process (>25:1 d.r.) was determined from the ^{1}H NMR spectrum of crude material.

R_f = 0.2 (Hexane/EtOAc = 9/1).

^{1}H NMR (500 MHz, $CDCl_3$, 298 K): δ 7.38–7.31 (m, 2H, H-7), 7.29 (d, *J* = 8.3 Hz, 2H, H-6), 7.25–7.21 (m, 1H, H-8), 7.03–6.97 (m, 2H, H-12 and H-14), 6.79 (s, 1H, H-4), 6.70–6.66 (m, 1H, H-13), 6.60 (d, *J* = 8.0 Hz, 1H, H-11), 3.92 (s, 1H, NH), 3.47 (dd, *J* = 10.4, 3.2 Hz, 1H, H-9), 2.87–2.74 (m, 2H, H-16), 2.44 (s, 1H, OH), 1.99–1.93 (m, 1H, H-17), 1.89 (s, 3H, H-18), 1.84–1.74 (m, 1H, H-17), 1.51 (s, 3H, H-1) ppm.

^{13}C NMR (125 MHz, $CDCl_3$, 298 K): δ 144.6 (C-10), 140.3 (C-3), 138.2 (C-5), 129.3 (C-6), 129.3 (C-14), 128.3 (C-7), 126.9 (C-12), 126.5 (C-8), 125.6 (C-4), 122.6 (C-15), 118.3 (C-13), 115.5 (C-11), 76.5 (C-2), 57.3 (C-9), 26.9 (C-16), 25.0 (C-1), 22.9 (C-17), 14.6 (C-18) ppm.

HRMS (ESI) m/z: $[M+H]^+$ calcd for $C_{20}H_{24}NO^+$ 294.1853, found 294.1847.

IR (thin film): ṽ 3304 (br), 1606 (s), 1481 (s), 1387 (s), 1309 (s), 1101 (s), 745 (s) cm^{-1}.

Specific rotation: $[\alpha]_D^{23}$ = –12.5 (*c* 0.5, CH_2Cl_2).

The **enantiomeric ratio** of **7a** was determined by SFC analysis (CHIRALPACK SB (25 cm), column temperature 25 °C, solvent CO_2/*i*-PrOH = 70/30, flow rate = 2.0 mL/min): t_R = 5.2 min (minor), t_R = 5.9 min (major).

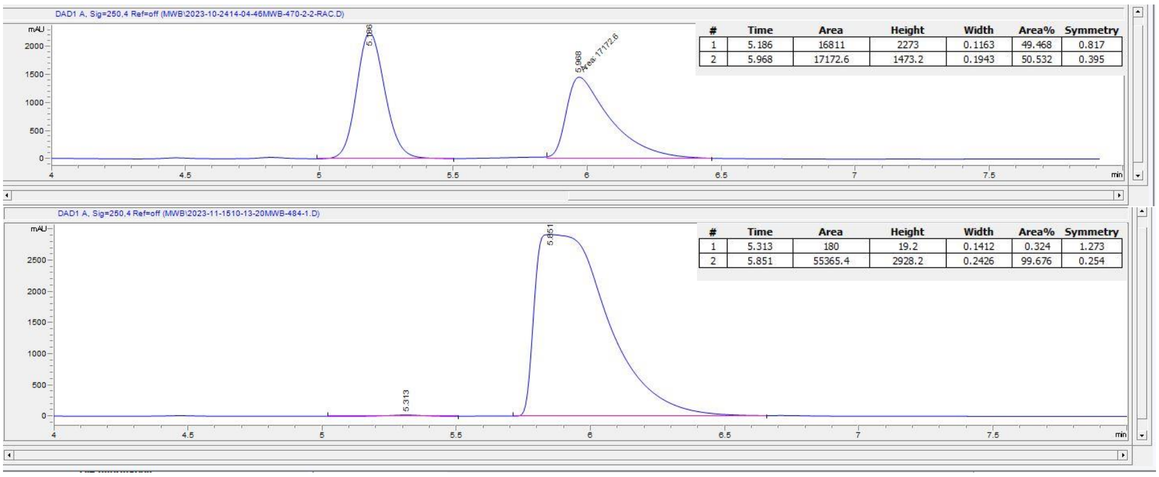

Racemic **3-methyl-4-phenyl-2-(1,2,3,4-tetrahydroquinolin-2-yl)but-3-en-2-ol** (70%, 1.3:1 d.r.) was prepared according to the procedure above, using (±)-BINAP as the ligand.

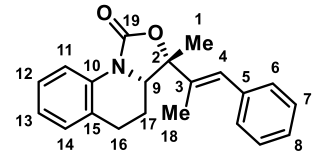

$C_{21}H_{21}NO_2$
M = 319.40

(3*R*,3a*S*)-3-Methyl-3-((*E*)-1-phenylprop-1-en-2-yl)-3,3a,4,5-tetrahydro-1*H*-oxazolo[3,4-*a*]-quinolin-1-one was prepared according to a reported procedure.[13b] A Schlenk tube was charged with (*R*,*E*)-3-methyl-4-phenyl-2-((*S*)-1,2,3,4-tetrahydroquinolin-2-yl)but-3-en-2-ol (**7a**, 29.3 mg, 0.10 mmol, 100 mol%) and triphosgene (44.5 mg, 0.15 mmol, 150 mol%). The *Schlenk* tube was evacuated and backfilled with N_2 for three times. Then THF (1 mL, 0.1 M) and Et_3N (55.7 µL, 40.4 mg, 0.40 mmol, 400 mol%) were added. The reaction mixture was stirred at room temperature for 12 hours. The reaction was quenched by water (10 mL) and extracted by ethyl acetate (3 × 10 mL). The combined organic phase was dried by anhydrous $MgSO_4$, filtered and concentrated under reduced pressure. Purification by flash column chromatography on silica gel (hexane/ethyl acetate from 100/1 to 9/1) afforded the product (23.5 mg, 74% yield, >99% ee, >25:1 d.r.) as a colorless solid. The diastereomer ratio of the process (>25:1 d.r.) was determined from the ^1H NMR spectrum of crude material.

R*f* = 0.2 (Hexane/EtOAc = 9/1).

M.P. 121–122 °C (Hexane/EtOAc).

^{1}H NMR (500 MHz, $CDCl_3$, 298 K): δ 8.13 (d, *J* = 8.3 Hz, 1H, H-11), 7.38–7.33 (m, 2H, H-7), 7.28–7.22 (m, 4H, H-6, H-8 and H-12), 7.12 (d, *J* = 7.7 Hz, 1H, H-14), 7.07–7.02 (m, 1H, H-13), 6.73 (s, 1H, H-4), 3.89 (dd, *J* = 12.2, 2.5 Hz, 1H, H-9), 2.95–2.90 (m, 2H, H-16), 2.02–1.96 (m, 1H, H-17), 1.93 (s, 3H, H-18), 1.79 (s, 3H, H-1), 1.76–1.67 (m, 1H, H-17) ppm.

^{13}C NMR (125 MHz, $CDCl_3$, 298 K): δ 154.3 (C-19), 137.1 (C-5), 135.5 (C-10), 135.1 (C-3), 129.2 (C-14), 129.1 (C-6), 128.4 (C-7), 127.3 (C-12), 127.2 (C-8), 127.0 (C-4), 125.2 (C-15), 123.8 (C-13), 119.8 (C-11), 83.6 (C-2), 64.3 (C-9), 27.2 (C-16), 26.2 (C-1), 23.7 (C-17), 16.1 (C-18) ppm.

HRMS (ESI) m/z: $[M+H]^+$ calcd for $C_{21}H_{22}NO_2^+$ 320.1645, found 320.1646.

IR (thin film): ṽ 1739 (s), 1493 (s), 1390 (s), 1226 (s), 1088 (s), 750 (s) cm^{-1}.

Specific rotation: $[\alpha]_D^{21}$ = +1.9 (*c* 0.5, CH_2Cl_2).

The **enantiomeric ratio** of **diastereomers 1** was determined by SFC analysis (CHIRALPACK SB (25 cm), column temperature 25 °C, solvent CO_2/*i*-PrOH = 70/30, flow rate = 2.0 mL/min): t_R = 4.7 min (minor), t_R = 5.5 min (major).

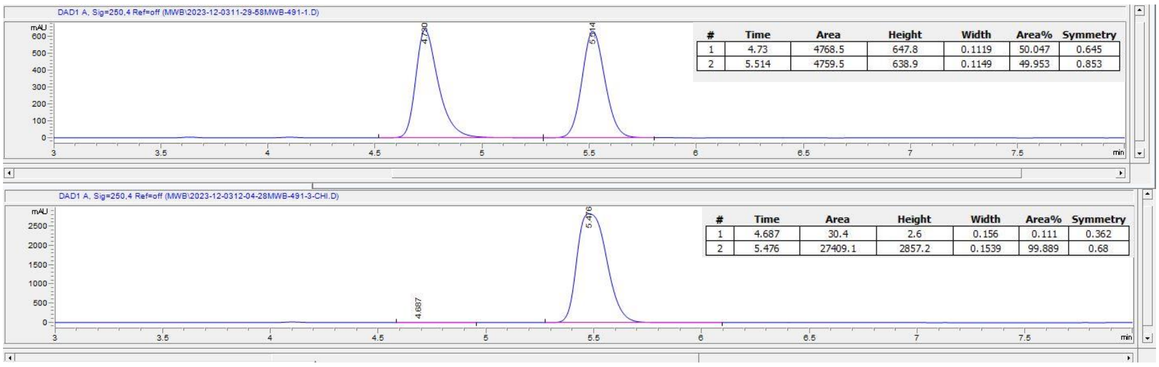

H,H-Noesy Spectra of (3*R*,3a*S*)-3-Methyl-3-((*E*)-1-phenylprop-1-en-2-yl)-3,3a,4,5-tetrahydro-1*H*-oxazolo[3,4-*a*]-quinolin-1-one

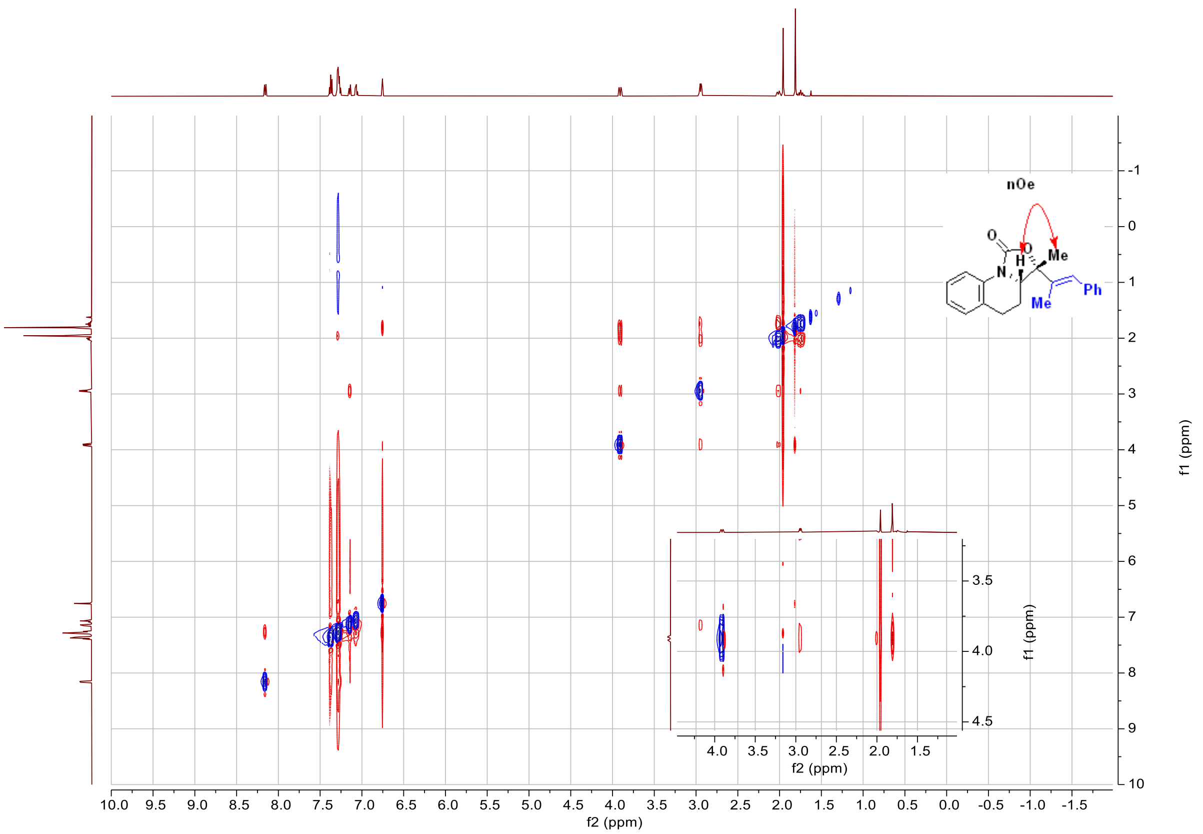

From the H,H-Noesy spectrum above, the correlation between H-9 and H-1 (3.89 ppm and 1.79 ppm) confirms the relative stereochemistry.

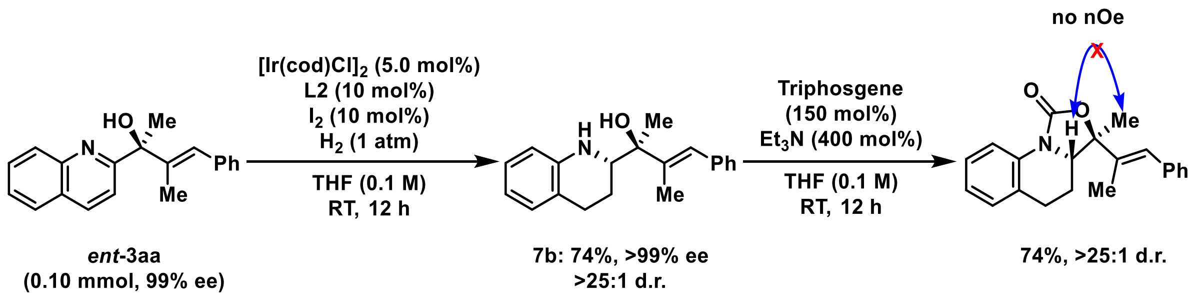

Scheme S15. Selective reduction of the quinoline unit of *ent*-**3aa**.

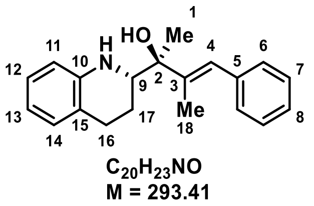

(*S*,*E*)-3-Methyl-4-phenyl-2-((*S*)-1,2,3,4-tetrahydroquinolin-2-yl)but-3-en-2-ol (**7b**) was prepared according to the procedure above, using (*S*,*E*)-3-methyl-4-phenyl-2-(quinolin-2-yl)but-3-en-2-ol (*ent*-**3aa**, 28.9 mg, 0.10 mmol, 100 mol%) as the reactant and **L2** ((*R*)-SEGPHOS, 6.11 mg, 10 µmol, 10 mol%) as the ligand. Purification by flash column chromatography on silica gel (hexane/ethyl acetate from 100/1 to 19/1) afforded **7b** (21.8 mg,

74% yield, >99% ee, >25:1 d.r.) as a brown oil. The diastereomer ratio of the process (>25:1 d.r.) was determined from the ^{1}H NMR spectrum of crude material.

R*f* = 0.3 (Hexane/EtOAc = 9/1).

^{1}H NMR (500 MHz, $CDCl_3$, 298 K): δ 7.40–7.36 (m, 2H, H-7), 7.31 (d, *J* = 8.2 Hz, 2H, H-6), 7.28–7.24 (m, 1H, H-8), 7.02–6.96 (m, 2H, H-12 and H-14), 6.89 (s, 1H, H-4), 6.70–6.66 (m, 1H, H-13), 6.52 (d, *J* = 7.9 Hz, 1H, H-11), 3.80 (s, 1H, NH), 3.51 (dd, *J* = 11, 2.9 Hz, 1H, H-9), 2.96–2.87 (m, 1H, H-16), 2.86–2.80 (m, 1H, H-16), 2.73 (s, 1H, OH), 2.15–2.09 (m, 1H, H-17), 1.91 (s, 3H, H-18), 1.95–1.85 (m, 1H, H-17), 1.43 (s, 3H, H-1) ppm.

^{13}C NMR (125 MHz, $CDCl_3$, 298 K): δ 144.5 (C-10), 141.8 (C-3), 138.0 (C-5), 129.2 (C-6 and C-14), 128.4 (C-7), 126.9 (C-12), 126.7 (C-8), 125.7 (C-4), 122.1 (C-15), 118.3 (C-13), 115.3 (C-11), 76.3 (C-2), 55.6 (C-9), 26.8 (C-16), 22.5 (C-1), 22.2 (C-17), 14.9 (C-18) ppm.

HRMS (ESI) m/z: $[M+H]^+$ calcd for $C_{20}H_{24}NO^+$ 294.1853, found 294.1849.

IR (thin film): ṽ 3405 (br), 1606 (s), 1482 (s), 1308 (s), 1095 (s), 742 (s) cm^{-1}.

Specific rotation: $[\alpha]_D^{20}$ = +26.5 (*c* 0.5, CH_2Cl_2).

The **enantiomeric ratio** of **7b** was determined by SFC analysis (CHIRALPACK SB (25 cm), column temperature 25 °C, solvent CO_2/*i*-PrOH = 70/30, flow rate = 2.0 mL/min): t_R = 4.4 min (minor), t_R = 5.1 min (major).

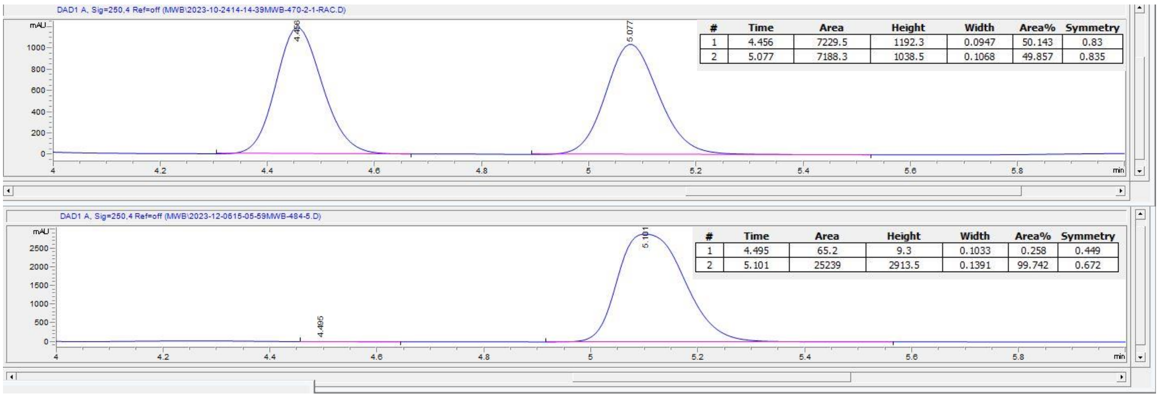

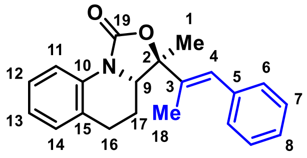

$C_{21}H_{21}NO_2$
M = 319.40

***trans*-3-Methyl-3-((*E*)-1-phenylprop-1-en-2-yl)-3,3a,4,5-tetrahydro-1*H*-oxazolo[3,4-*a*]quinolin-1-one** was prepared according to the procedure above, using racemic methyl-4-

phenyl-2-(1,2,3,4-tetrahydroquinolin-2-yl)but-3-en-2-ol ((±)-**7b**, 29.3 mg, 0.10 mmol, 100 mol%) as the starting material. Purification by flash column chromatography on silica gel (hexane/ethyl acetate from 100/1 to 9/1) afforded the product (23.6 mg, 74% yield, >25:1 d.r.) as a colorless oil. The diastereomer ratio of the process (>25:1 d.r.) was determined from the ^{1}H NMR spectrum of crude material.

R*f* = 0.3 (Hexane/EtOAc = 9/1).

^{1}H NMR (500 MHz, $CDCl_3$, 298 K): δ 8.41 (d, *J* = 8.4 Hz, 1H, H-11), 7.38–7.33 (m, 2H, H-7), 7.29–7.21 (m, 4H, H-6, H-8 and H-12), 7.14 (d, *J* = 7.5 Hz, 1H, H-14), 7.05–6.99 (m, 1H, H-13), 6.75 (s, 1H, H-4), 4.04 (dd, *J* = 12.1, 2.6 Hz, 1H, H-9), 3.00–2.96 (m, 2H, H-16), 2.18–2.11 (m, 1H, H-17), 2.01–1.90 (m, 1H, H-17), 1.96 (s, 3H, H-18), 1.60 (s, 3H, H-1) ppm.

^{13}C NMR (125 MHz, $CDCl_3$, 298 K): δ 153.4 (C-19), 138.1 (C-3), 137.0 (C-5), 135.6 (C-10), 129.2 (C-14), 129.1 (C-6), 128.3 (C-7), 127.4 (C-12), 127.1 (C-8), 125.6 (C-4), 124.3 (C-15), 123.3 (C-13), 118.0 (C-11), 83.9 (C-2), 62.1 (C-9), 27.5 (C-16), 23.2 (C-17), 20.8 (C-1), 14.2 (C-18) ppm.

HRMS (ESI) m/z: $[M+H]^+$ calcd for $C_{21}H_{22}NO_2^+$ 320.1645, found 320.1645.

IR (thin film): ṽ 1740 (s), 1493 (s), 1363 (s), 1226 (s), 1088 (s), 750 (s) cm^{-1}.

H,H-Noesy Spectra of *trans*-3-Methyl-3-((*E*)-1-phenylprop-1-en-2-yl)-3,3a,4,5-tetrahydro-1*H*-oxazolo[3,4-*a*]quinolin-1-one

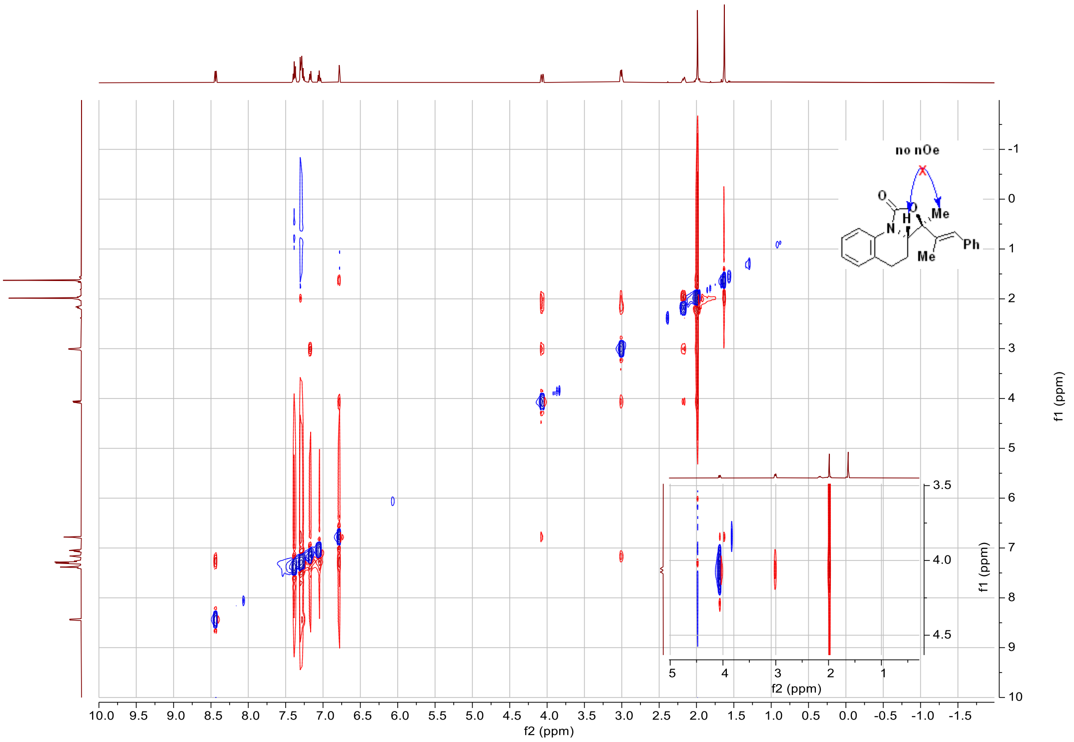

From the H,H-Noesy spectrum above, the lack of correlation between H-9 and H-1 (4.04 ppm and 1.60 ppm) confirms the relative stereochemistry.

7 Mechanistic Studies

7.1 Reaction of an Alkyne and Alkene under Standard Conditions in the Absence of a Heteroaryl Alcohol

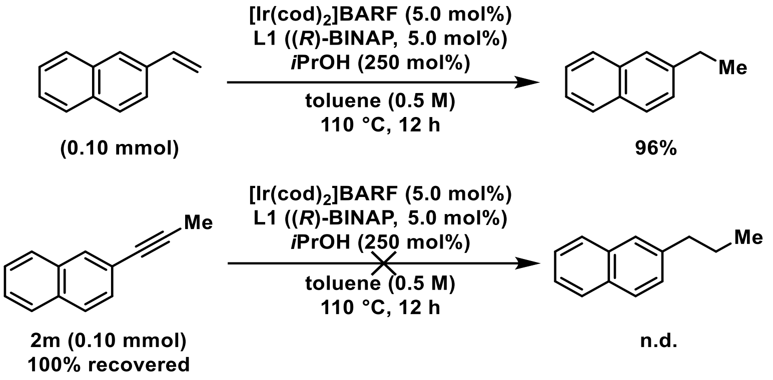

Scheme S16. Reaction of an alkyne/alkene under standard conditions.

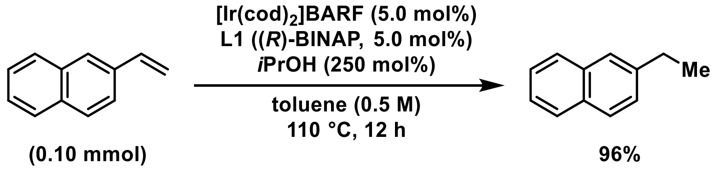

GP6: A flame-dried *Schlenk* tube was charged with 2-vinylnaphthalene (15.4 mg, 0.1 mmol, 100 mol%), [Ir(cod)$_2$]BARF (6.36 mg, 5.0 µmol, 5.0 mol%) and **L1** (3.11 mg, 5.0 µmol, 5.0 mol%). The *Schlenk* tube was evacuated and backfilled with N_2 for three times. Then toluene (0.2 mL, 0.5 M) was added, followed by the addition of *i*-PrOH (19.1 µL, 15.0 mg, 0.25 mmol, 250 mol%). The tube was sealed and heated at 110 °C for 12 hours. After cooling to room temperature, the solvent was removed under reduced pressure. Purification by flash column chromatography on silica gel (hexane) afforded the alkane (15.0 mg, 96% yield) as a colorless oil.

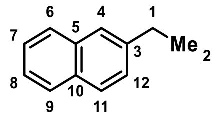

$C_{12}H_{12}$
M = 156.23

2-Ethylnaphthalene

R*f* = 0.6 (Hexane).

^{1}H NMR (500 MHz, $CDCl_3$, 298 K): δ 7.87–7.79 (m, 3H, H-11 and ArH), 7.67 (s, 1H, H-4), 7.52–7.43 (m, 2H, ArH), 7.42–7.37 (m, 1H, H-12), 2.86 (q, *J* = 7.7 Hz, 2H, H-1), 1.38 (t, *J* = 7.7 Hz, 3H, H-2) ppm.

^{13}C NMR (125 MHz, $CDCl_3$, 298 K): δ 141.9 (C-3), 133.9 (C-5), 132.1 (C-10), 127.9 (Ar), 127.7 (Ar), 127.6 (Ar), 127.2 (Ar), 126.0 (Ar), 125.7 (Ar), 125.2 (Ar), 29.2 (C-1), 15.7 (C-2) ppm.

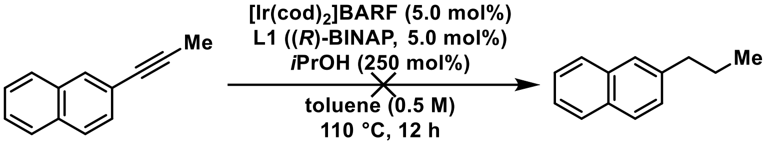

2m (0.10 mmol)
100% recovered

n.d.

GP6: A flame-dried *Schlenk* tube was charged with 2-(prop-1-yn-1-yl)naphthalene (**2m**, 16.6 mg, 0.1 mmol, 100 mol%), $[Ir(cod)_2]$BARF (6.36 mg, 5.0 μmol, 5.0 mol%) and **L1** (3.11 mg, 5.0 μmol, 5.0 mol%). The *Schlenk* tube was evacuated and backfilled with N_2 for three times. Then toluene (0.2 mL, 0.5 M) was added, followed by the addition of *i*-PrOH (19.1 μL, 15.0 mg, 0.25 mmol, 250 mol%). The tube was sealed and heated at 110 °C for 12 hours. After cooling to room temperature, the solvent was removed under reduced pressure. ^{1}H NMR of the crude mixture indicated the results above.

7.2 Reaction of Ketone 8 under Standard Conditions

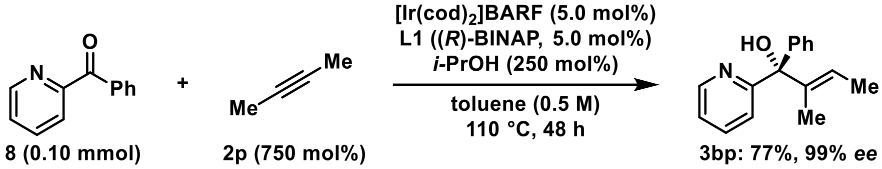

Scheme S17. Reaction of ketone 8 under standard conditions.

(*S*,*E*)-2-Methyl-1-phenyl-1-(pyridin-2-yl)but-2-en-1-ol (**3bp**, **GP 6**) was prepared from phenyl(pyridin-2-yl)methanone (**8**, 18.3 mg, 0.10 mmol, 100 mol%) and but-2-yne (**2p**, 58.7 μL, 40.6 mg, 0.75 mmol, 750 mol%), using [Ir(cod)$_2$]BARF (6.36 mg, 5.0 μmol, 5.0 mol%), **L1** (3.11 mg, 5.0 μmol, 5.0 mol%) and *i*-PrOH (19.1 μL, 15.0 mg, 0.25 mmol, 250 mol%) in toluene (0.2 mL) at 110 °C for 48 h. Purification by flash column chromatography on silica gel (hexane/ethyl acetate from 100/1 to 19/1) afforded **3bp** (18.4 mg, 77% yield, 99% ee) as a colorless oil.

7.3 Deuterium Labelling and Exchange Studies.

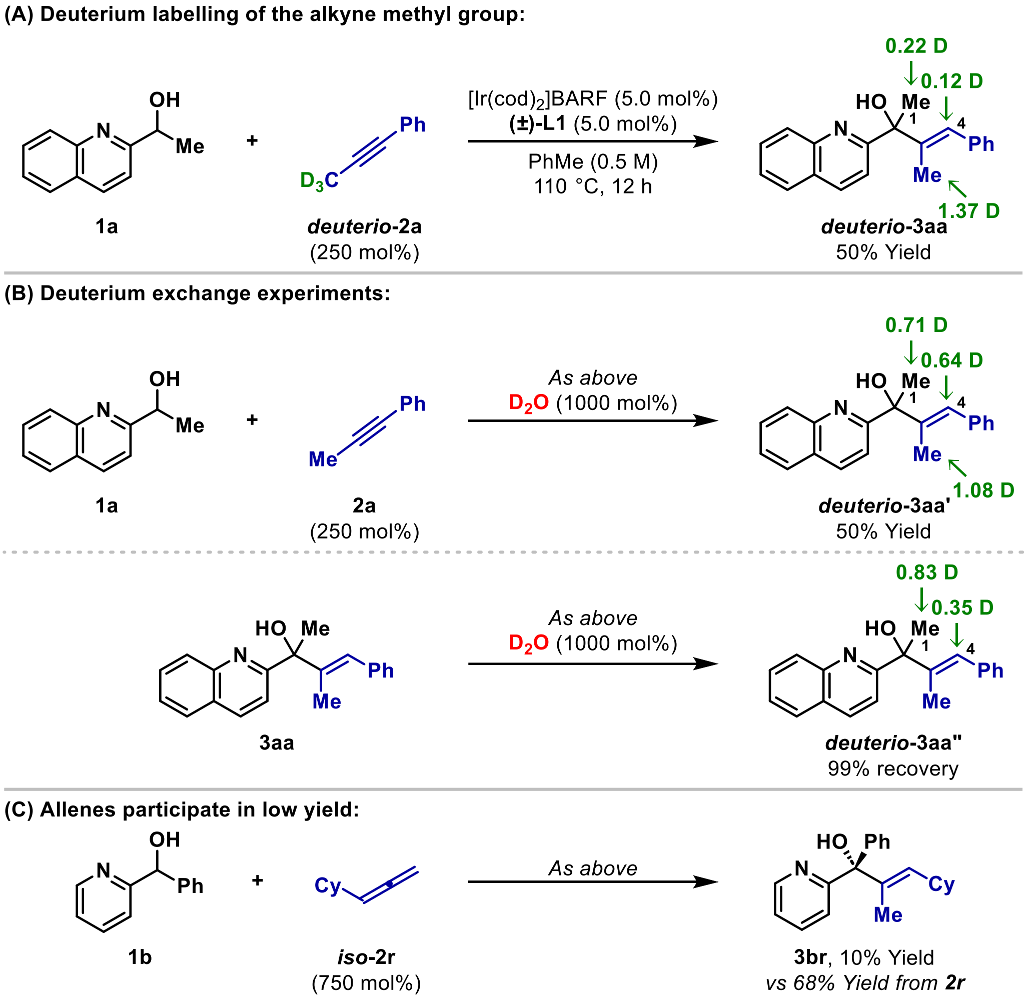

Scheme S18. Deuterium labelling and exchange studies.

Using ***deuterio*-2a**, transfer of the deuterium label from the alkyne methyl group to C1 and C4 of ***deuterio*-3aa** was observed (Scheme S18A). A deuterium exchange experiment showed that this deuterium transfer event does not occur at the stage of the product (Scheme S18B); interestingly, exchange at C1-H and C4-H of ***deuterio*-3aa’** was observed, and this is likely facilitated by heteroaryl-directed C-H activation. Collectively, these results suggest that the deuterium exchange observed in Scheme S18A occurs at the stage of the alkyne, most likely via iridium-mediated reversible alkyne-allene isomerization. To probe the viability of an allene intermediate, we reacted alcohol **1b** with cyclohexylallene under optimized conditions (Scheme S18C). This experiment provided **3br**, but in only 10% yield (versus 68% yield using alkyne **2r**).

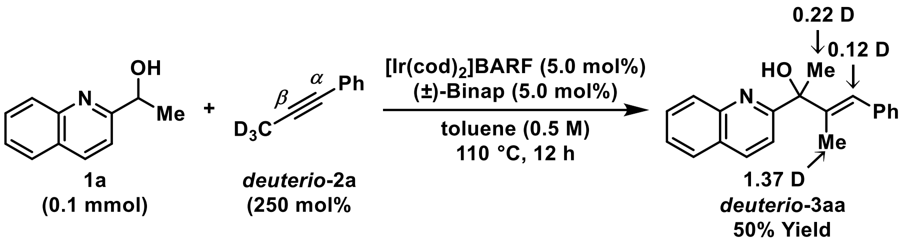

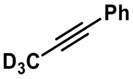

deuterio-**2a**

(Prop-1-yn-1-yl-*d*$_3$)benzene (*deuterio*-**2a**, **GP4**) were prepared from phenylacetylene (510 mg, 5.0 mmol, 100 mol%) and iodomethane-*d*$_3$ (0.467 mL, 1.09 g, 7.5 mmol, 150 mol%), using NaHMDS (3.8 mL, 2 M in THF, 7.5 mmol, 150 mol%) as the base in THF (10 mL, 0.5 M) at room temperature for 24 h. Purification by flash column chromatography on silica gel (hexane) afforded **2a** (422 mg, 71% yield) as a colorless oil. Data agree with those reported.[14]

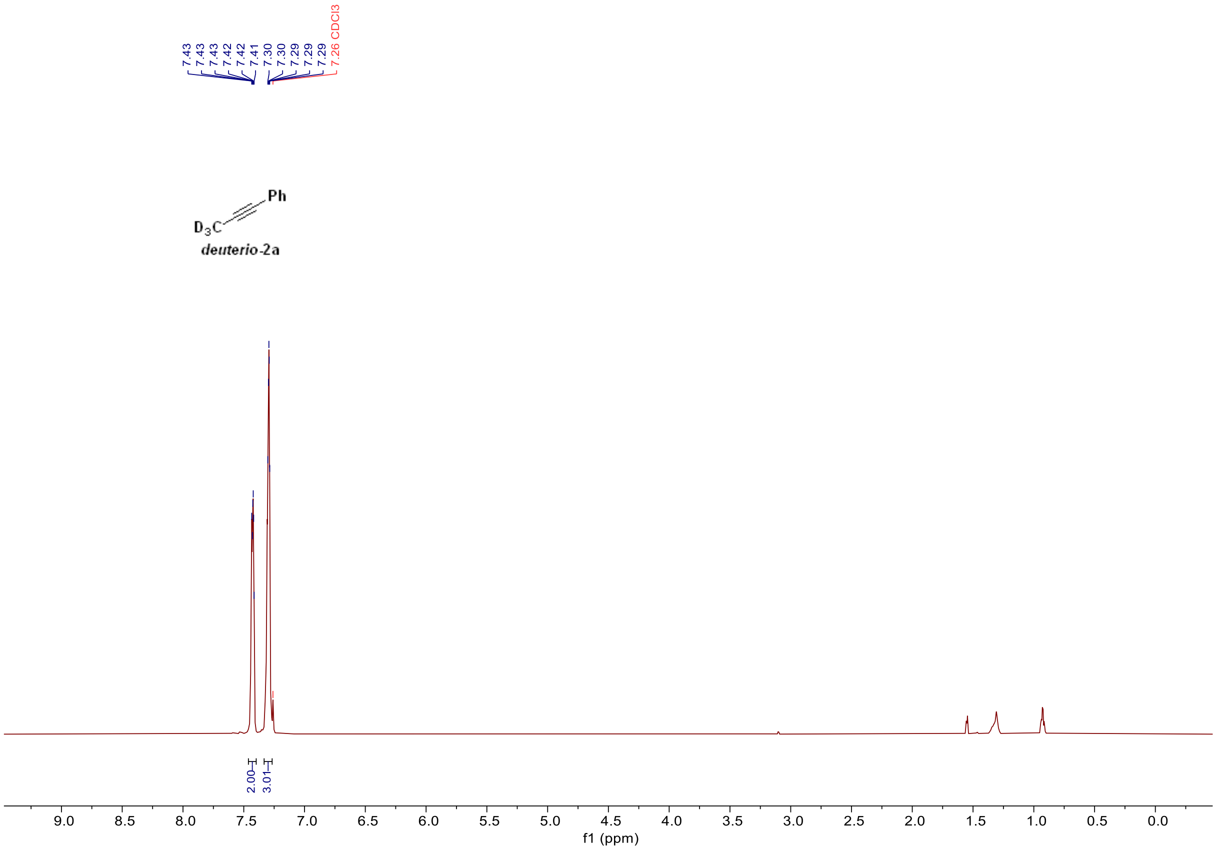

GP6: A flame-dried *Schlenk* tube was charged with 1-(quinolin-2-yl)ethan-1-ol (**1a**, 17.3 mg, 0.10 mmol, 100 mol%), [Ir(cod)$_2$]BARF (6.36 mg, 5.0 μmol, 5.0 mol%) and (±)-BINAP (3.11 mg, 5.0 μmol, 5.0 mol%). The *Schlenk* tube was evacuated and backfilled with N_2 for three times. Then toluene (0.2 mL, 0.5 M) was added, followed by the addition of (prop-1-yn-1-yl-*d*$_3$)benzene (*deuterio*-**2a**, 31.6 μL, 29.7 mg, 0.25 mmol, 250 mol%). The tube was sealed and

heated at 110 °C for 12 hours. After cooling to room temperature, the solvent was removed under reduced pressure. Purification by flash column chromatography on silica gel (hexane/ethyl acetate from 100/1 to 19/1) afforded ***deuterio*-3aa** (14.5 mg, 50% yield) as a colorless solid. The obtained products were analyzed by ^{1}H NMR and ^{2}H NMR spectroscopy using $CDCl_3$ or $CHCl_3$ as solvent.

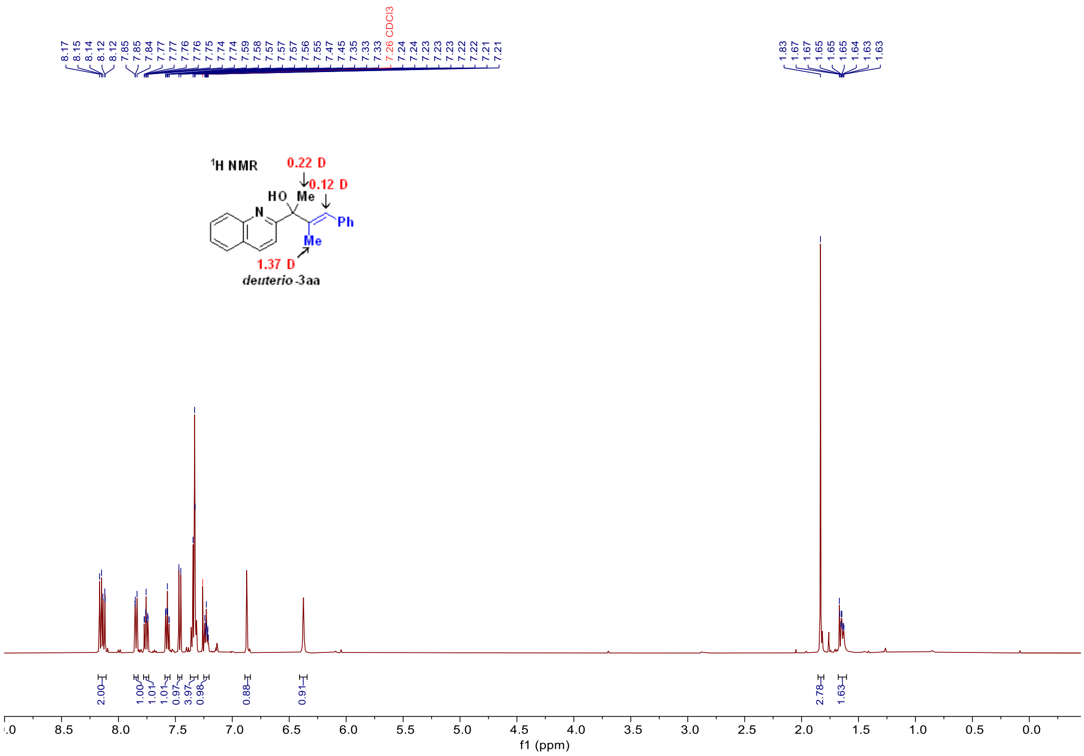

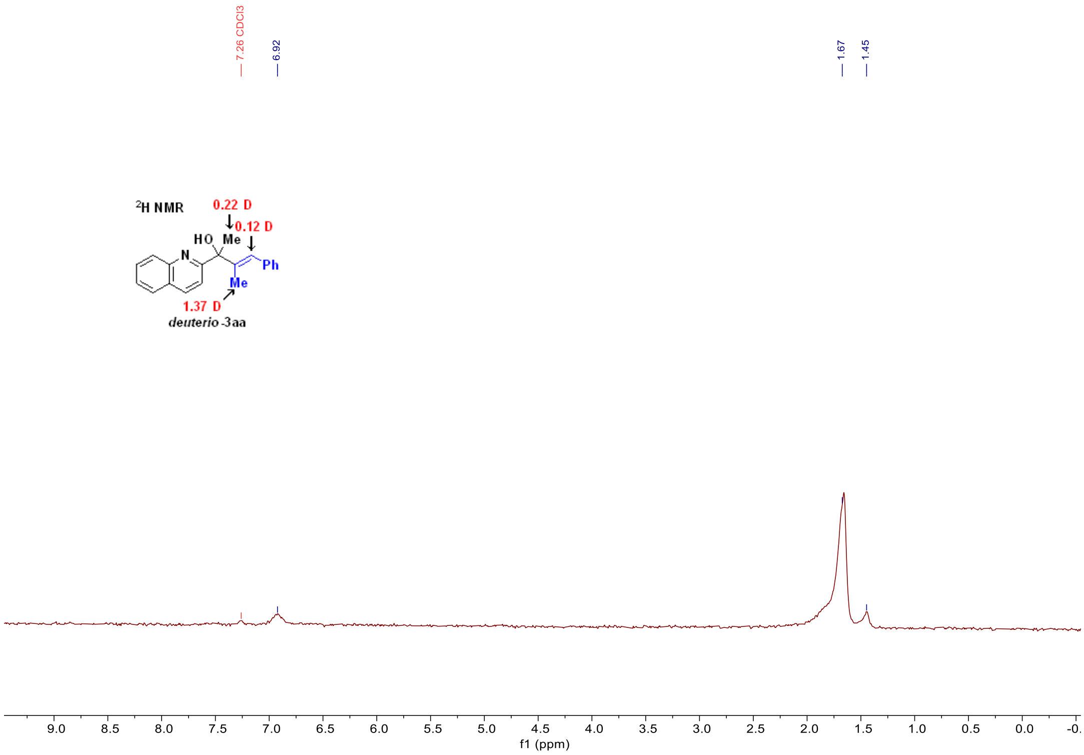

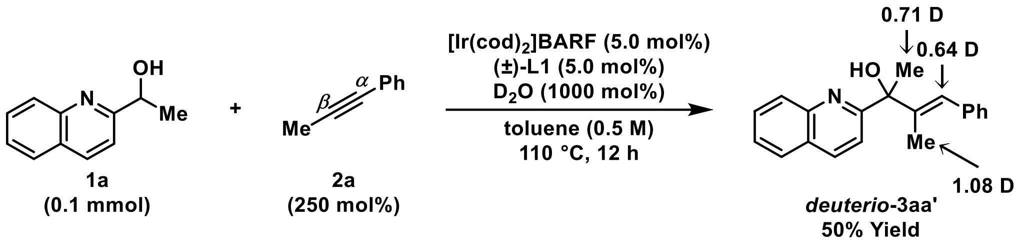

GP6: A flame-dried *Schlenk* tube was charged with 1-(quinolin-2-yl)ethan-1-ol (**1a**, 17.3 mg, 0.10 mmol, 100 mol%), [Ir(cod)$_2$]BARF (6.36 mg, 5.0 μmol, 5.0 mol%) and (±)-**L1** (3.11 mg, 5.0 μmol, 5.0 mol%). The *Schlenk* tube was evacuated and backfilled with N_2 for three times. Then toluene (0.2 mL, 0.5 M) was added, followed by the addition of 1-phenyl-1-propyne (**2a**, 31.6 μL, 29.0 mg, 0.25 mmol, 250 mol%) and D_2O (18 μL, 20.0 mg, 1.0 mmol, 1000 mol%). The tube was sealed and heated at 110 °C for 12 hours. After cooling to room temperature, the solvent was removed under reduced pressure. Purification by flash column chromatography on silica gel (hexane/ethyl acetate from 100/1 to 19/1) afforded ***deuterio*-3aa'** (14.5 mg, 50% yield) as a colorless solid. The obtained products were analyzed by ^{1}H NMR and ^{2}H NMR spectroscopy using $CDCl_3$ or $CHCl_3$ as solvent.

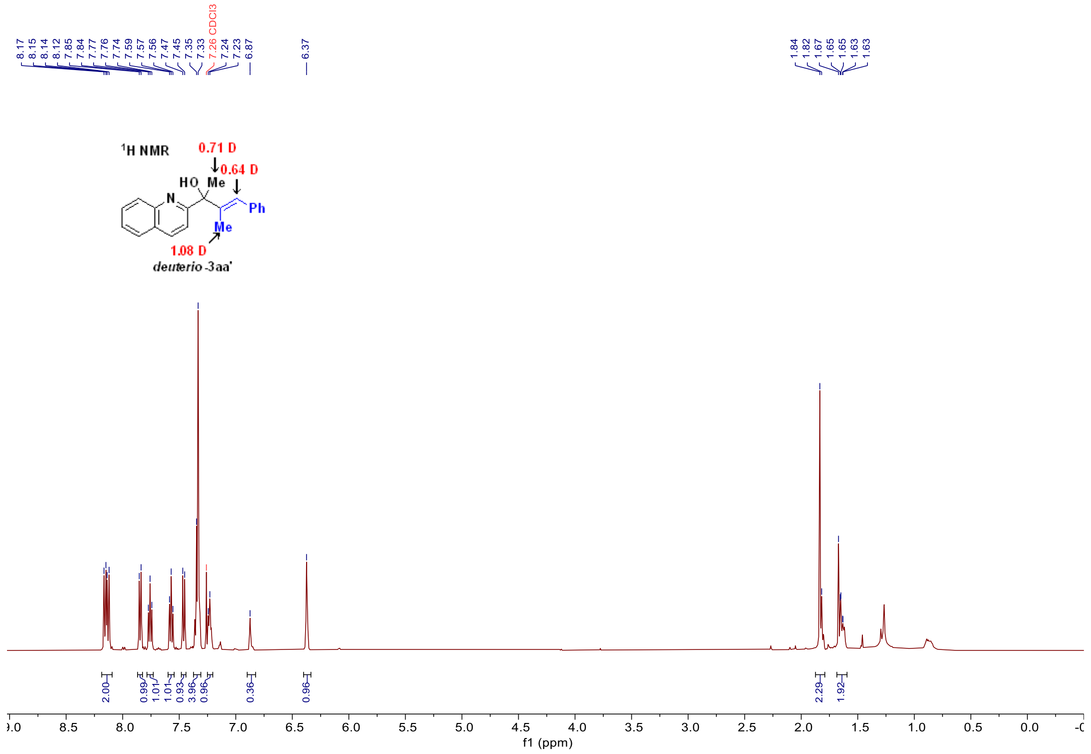

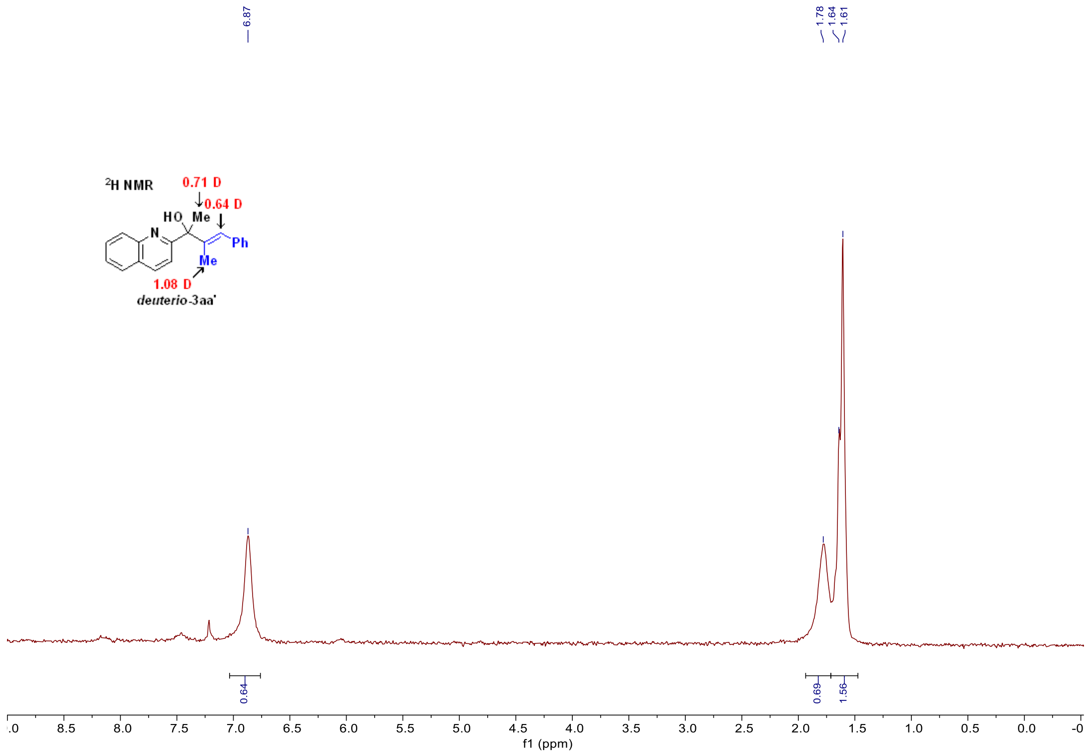

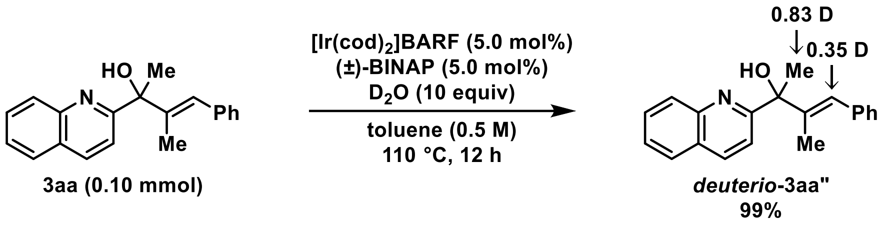

GP6: A flame-dried *Schlenk* tube was charged with 3-methyl-4-phenyl-2-(quinolin-2-yl)but-3-en-2-ol (**3aa**, 28.9 mg, 0.10 mmol, 100 mol%), [Ir(cod)$_2$]BARF (6.36 mg, 5.0 µmol, 5.0 mol%) and (±)-BINAP (3.11 mg, 5.0 µmol, 5.0 mol%). The *Schlenk* tube was evacuated and backfilled with N_2 for three times, then toluene (0.2 mL, 0.5 M) was added, followed by the addition of D_2O (18 µL, 20.0 mg, 1.0 mmol, 1000 mol%). The tube was sealed and heated at 110 °C for 12 hours. After cooling to room temperature, the solvent was removed under reduced pressure. Purification by flash column chromatography on silica gel (hexane/ethyl acetate from 100/1 to 19/1) afforded ***deuterio*-3aa"** (28.5 mg, 99% yield) as a colorless solid. The obtained products were analyzed by ^{1}H NMR and ^{2}H NMR spectroscopy using $CDCl_3$ or $CHCl_3$ as solvent.

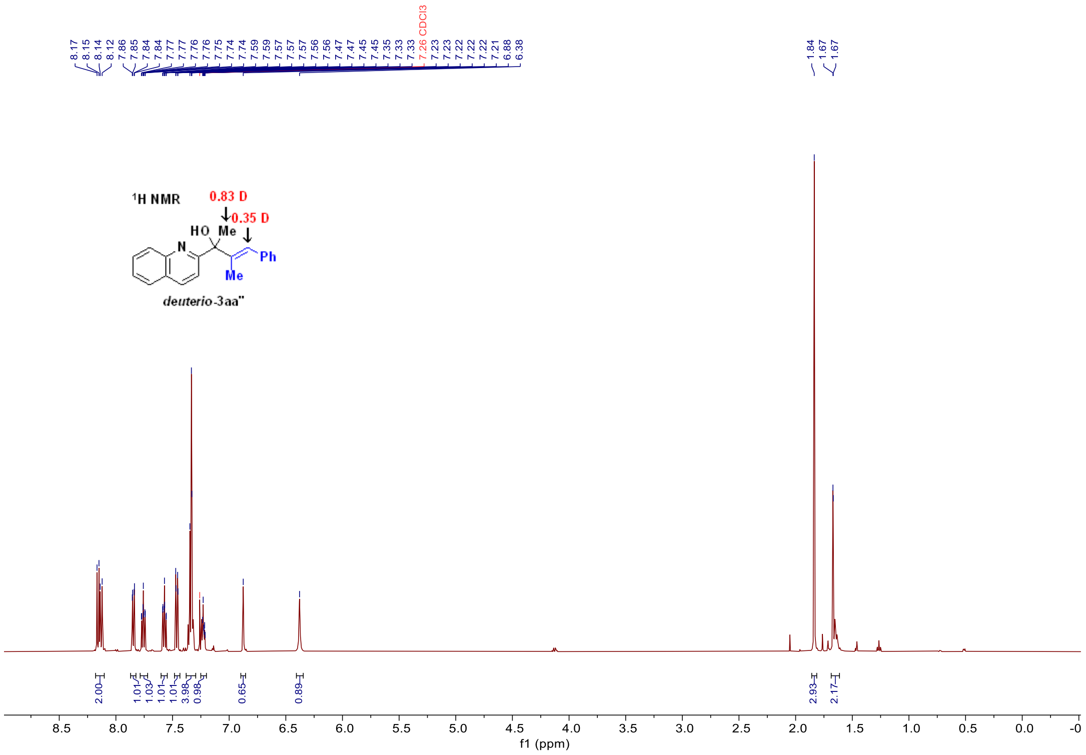

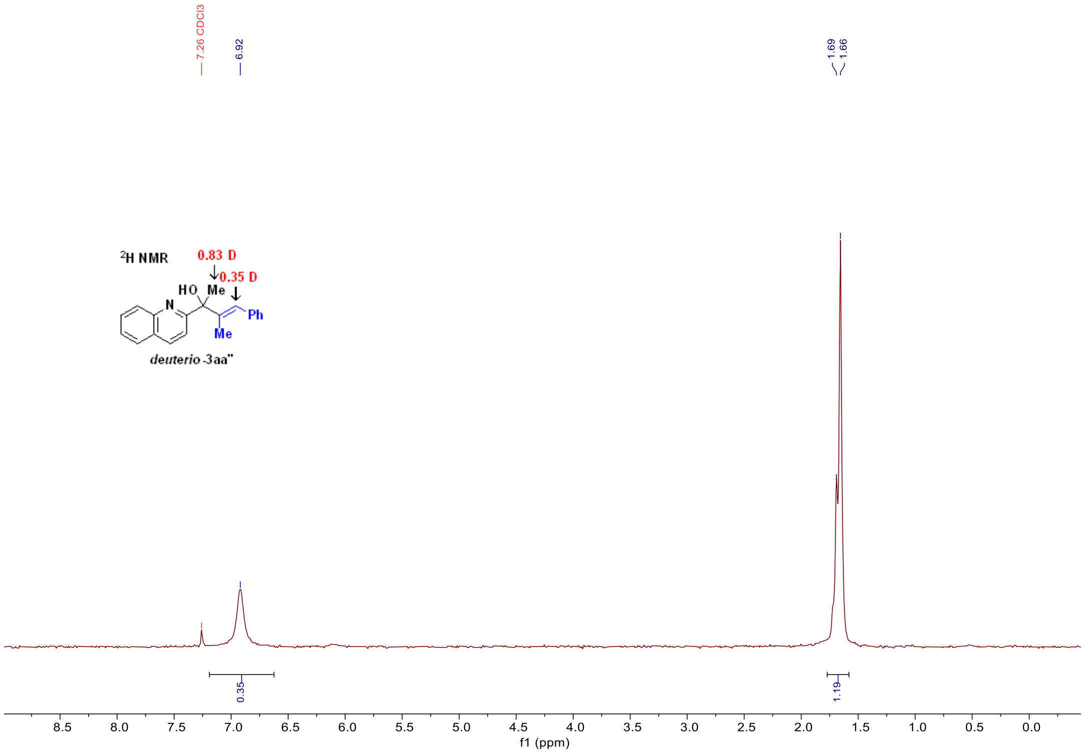

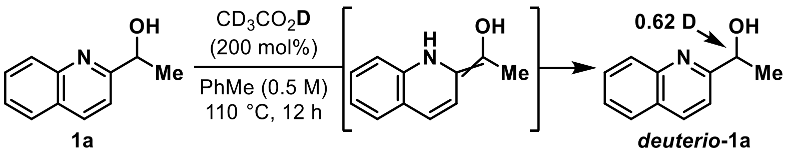

Scheme S19. Aza-enolization is viable under acidic conditions.

The reaction was conducted according to a modified porcedure.[15] A flame-dried *Schlenk* tube was charged with 1-(quinolin-2-yl)ethan-1-ol (**1a**, 17.3 mg, 0.10 mmol, 100 mol%). The *Schlenk* tube was evacuated and backfilled with N_2 for three times. Then toluene (0.2 mL, 0.5 M) was added, followed by the addition of CD_3CO_2D (11.5 µL, 12.2 mg, 0.20 mmol, 200 mol%). The tube was sealed and heated at 110 °C for 12 hours. After cooling to room temperature, the solvent was removed under reduced pressure. Purification by flash column chromatography on silica gel (hexane/ethyl acetate from 100/1 to 19/1) afforded ***deuterio*-1a** (14.5 mg, 84% yield) as a colorless solid. The obtained products were analyzed by ^1H NMR and ^2H NMR spectroscopy using $CDCl_3$ or $CHCl_3$ as solvent.

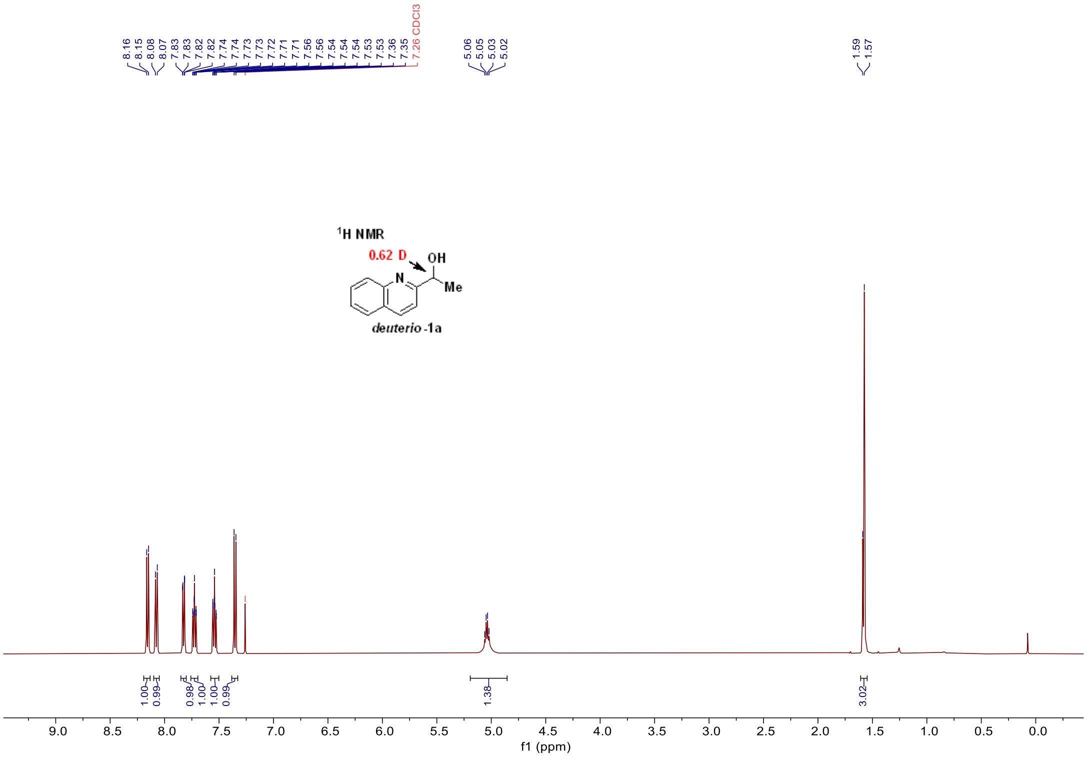

^1H NMR spectrum of *deuterio*-**1a** in $CDCl_3$

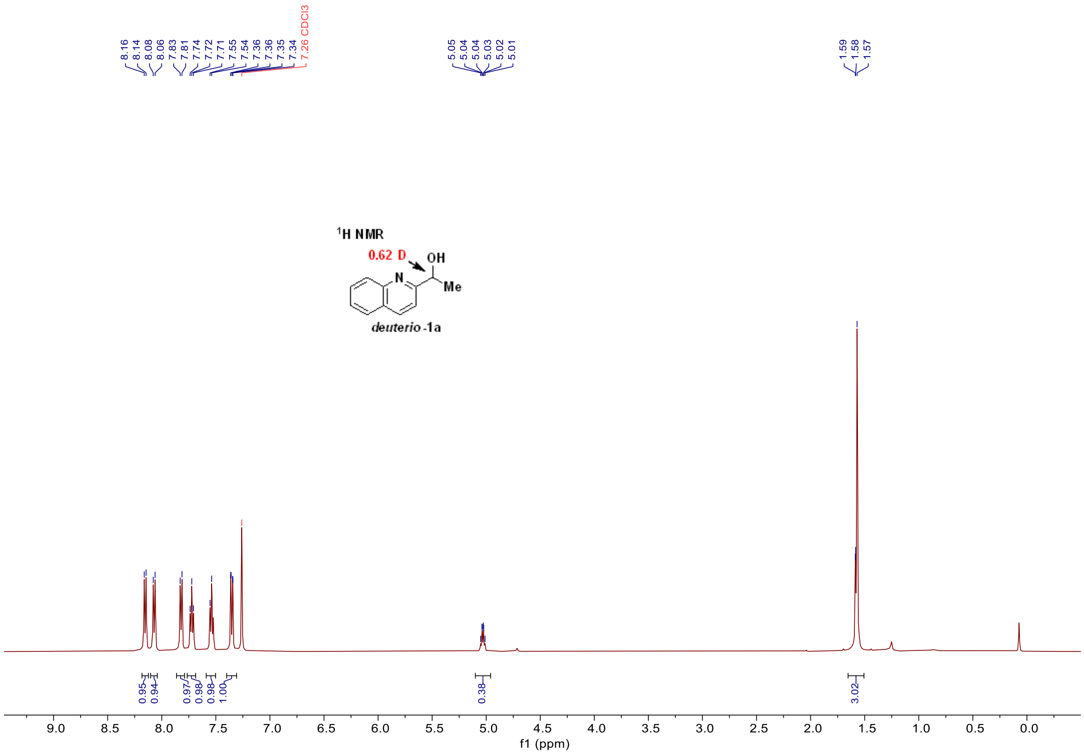

^{1}H NMR spectrum of *deuterio*-**1a** with D_2O in $CDCl_3$

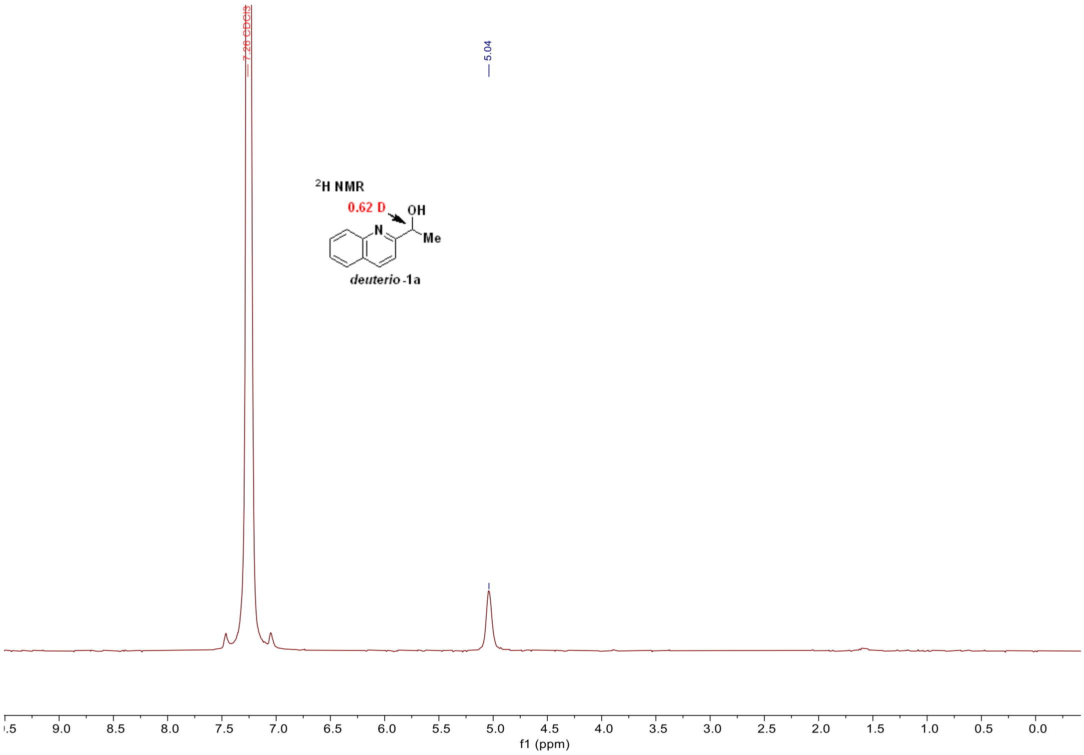

7.4 Visual Kinetic Analysis

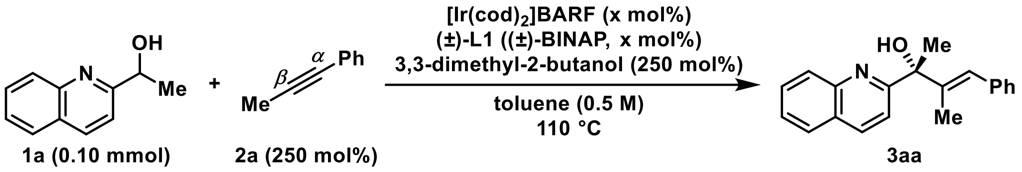

Scheme S20. Visual Kinetic Analysis of the reaction with **1a** and **2a**.

The reaction kinetic was determined using Burés' normalized time scale method.[16] Five parallel experiments of 1-(quinolin-2-yl)ethan-1-ol (**1a**, 17.3 mg, 0.10 mmol, 100 mol%) and 1-phenyl-1-propyne (**2a**, 31.6 μL, 29.0 mg, 0.25 mmol, 250 mol%) were conducted, using [Ir(cod)$_2$]BARF (6.36 mg, 5.0 μmol, 5.0 mol%), (±)-**L1** (3.11 mg, 5.0 μmol, 5.0 mol%) and 3,3-dimethylbutan-2-ol (32 μL, 25.5 mg, 0.25 mmol, 250 mol%) in toluene (0.2 mL). The concentrations of **3aa** at 0 h, 1.5 h, 3 h, 4.5 h and 7.5 h were determined by ^{1}H NMR analysis using 1,3,5-trimethoxybenzene as the internal standard. Another five parallel experiments used [Ir(cod)$_2$]BARF (8.90 mg, 7.0 μmol, 7.0 mol%) and (±)-**L1** (4.36 mg, 7.0 μmol, 7.0 mol%). The concentrations of **3aa** at 0 h, 1 h, 3 h, 4 h and 7 h were determined by ^{1}H NMR analysis using 1,3,5-trimethoxybenzene as the internal standard. The results are listed as below:

5 mol% [Ir(cod)$_2$]BARF 0.0198 mol/L		7 mol% [Ir(cod)$_2$]BARF 0.0227 mol/L	
time (h)	concentration (**3aa**, mol/L)	time (h)	concentration (**3aa**, mol/L)
0	0	0	0
1.5	0.04536	1	0.04153
3	0.07564	3	0.08401
4.5	0.1123	4	0.1188
7.5	0.1735	7	0.1908

Graphical kinetic analysis: the order in catalyst ([Ir(cod)$_2$]BARF/(±)-BINAP) is approximately **1**.

Order in cat = **0.5** change this value to check other orders

**Experiment 1 [A] 0.5010; [Cat] =**	**0.0198**	5 mol%	[Ir] (0.020 M)
time (h)	[A]	[cat]^I2	t[cat]^I2
0	0	0.140712	0
1.5	0.04536	0.140712	0.211069
3	0.07564	0.140712	0.422137
4.5	0.1123	0.140712	0.633206
7.5	0.1735	0.140712	1.055344

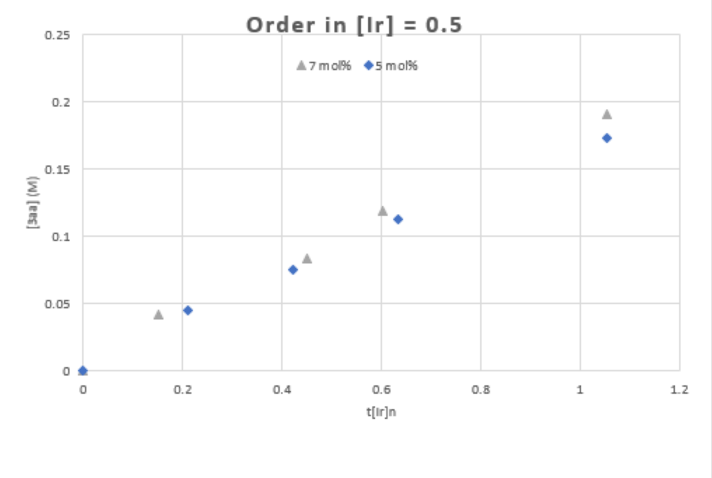

**Experiment 3 [A] 0.5009; [Cat] =**	**0.02267**	7 mol%	[Ir] (0.023 M)
time (h)	[A]	[cat]^I2	t[cat]^I2
0	0	0.150566	0
1	0.04153	0.150566	0.150566
3	0.08401	0.150566	0.451697
4	0.1188	0.150566	0.602262
7	0.1908	0.150566	1.053959

Order in cat = **1** change this value to check other orders

**Experiment 1 [A] 0.5010; [Cat] =**	**0.0198**	5 mol%	[Ir] (0.020 M)
time (h)	[A]	[cat]^I2	t[cat]^I2
0	0	0.0198	0
1.5	0.04536	0.0198	0.0297
3	0.07564	0.0198	0.0594
4.5	0.1123	0.0198	0.0891
7.5	0.1735	0.0198	0.1485

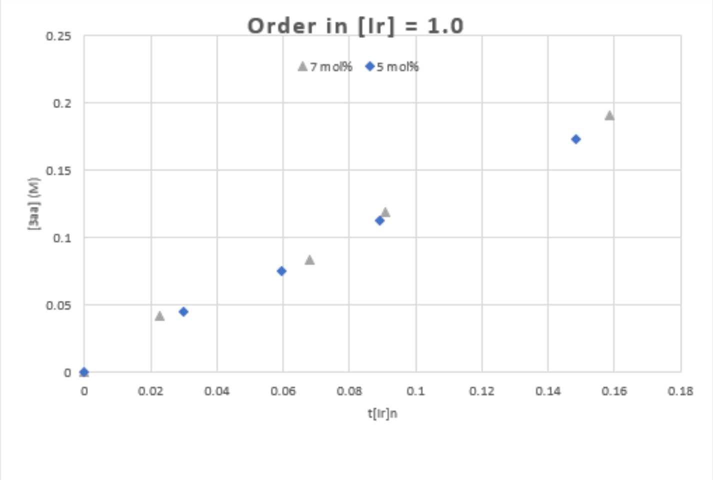

**Experiment 3 [A] 0.5009; [Cat] =**	**0.02267**	7 mol%	[Ir] (0.023 M)
time (h)	[A]	[cat]^I2	t[cat]^I2
0	0	0.02267	0
1	0.04153	0.02267	0.02267
3	0.08401	0.02267	0.06801
4	0.1188	0.02267	0.09068
7	0.1908	0.02267	0.15869

Experiment 1 [A] 0.5010; [Cat] =	0.0198	5 mol%	[Ir] (0.020 M)	Order in cat =	2	change this value to check other orders
time (h)	[A]	[cat]^I2	t[cat]^I2			
0	0	0.000392	0			
1.5	0.04536	0.000392	0.000588			
3	0.07564	0.000392	0.001176			
4.5	0.1123	0.000392	0.001764			
7.5	0.1735	0.000392	0.00294			

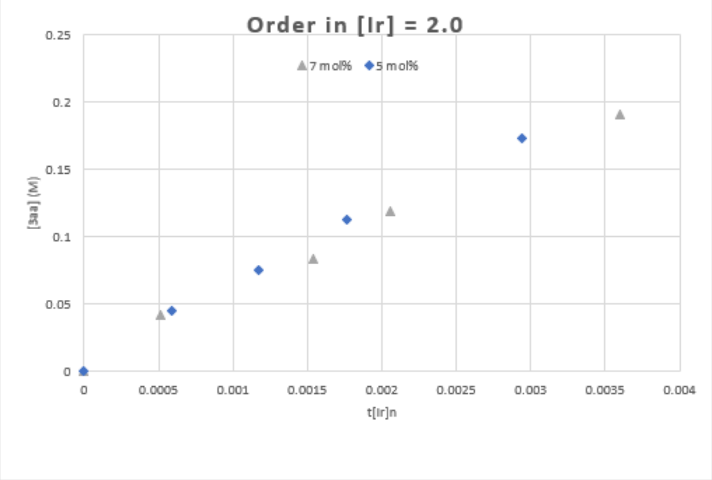

Experiment 3 [A] 0.5009; [Cat] =	0.02267	7 mol%	[Ir] (0.023 M)
time (h)	[A]	[cat]^I2	t[cat]^I2
0	0	0.000514	0
1	0.04153	0.000514	0.000514
3	0.08401	0.000514	0.001542
4	0.1188	0.000514	0.002056
7	0.1908	0.000514	0.003598

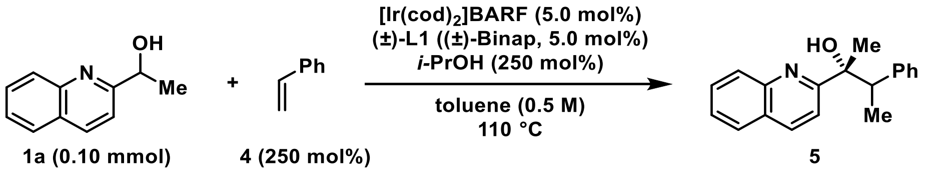

Scheme S21. Visual Kinetic Analysis of the reaction with **1a** and **4**.

The reaction kinetic was determined using Burés' normalized time scale method.[16] Five parallel experiments of 1-(quinolin-2-yl)ethan-1-ol (**1a**, 17.3 mg, 0.10 mmol, 100 mol%) and styrene (**4**, 28.7 μL, 26.0 mg, 0.25 mmol, 250 mol%) were conducted, using [Ir(cod)$_2$]BARF (6.36 mg, 5.0 μmol, 5.0 mol%), (±)-BINAP (3.11 mg, 5.0 μmol, 5.0 mol%) and *i*-PrOH (19.1 μL, 15.0 mg, 0.25 mmol, 250 mol%) in toluene (0.2 mL, 0.5 M). The concentrations of **5** at 0 h, 2 h, 4 h, 7 h and 9 h were determined by ^{1}H NMR analysis using 1,3,5-trimethoxybenzene as the internal standard. Another five parallel experiments used [Ir(cod)$_2$]BARF (8.90 mg, 7.0 μmol, 7.0 mol%) and (±)-BINAP (4.36 mg, 7.0 μmol, 7.0 mol%). The concentrations of **5** at 0 h, 1 h, 3 h, 4 h and 7 h were determined by ^{1}H NMR analysis using 1,3,5-trimethoxybenzene as the internal standard. The results are listed as below:

5 mol% [Ir(cod)$_2$]BARF 0.01923 mol/L		7 mol% [Ir(cod)$_2$]BARF 0.02593 mol/L	
time (h)	concentration (**5**, mol/L)	time (h)	concentration (**5**, mol/L)
0	0	0	0
2	0.08354	1	0.05337
4	0.1477	3	0.148
7	0.2269	4	0.1915
9	0.274	7	0.3071

Graphical kinetic analysis: the order in catalyst ([Ir(cod)$_2$]BARF/(±)-BINAP) is approximately **1**.

Experiment 1;	[Cat] =	0.01923	5 mol%	[Ir] (0.01923 M)	Order in cat =	0.5	change this value to check other orders
time (h)		[5]	[cat]^I2	t[cat]^I2			
	0	0	0.138672	0			
	2	0.08354	0.138672	0.277345			
	4	0.1477	0.138672	0.554689			
	7	0.2269	0.138672	0.970706			
	9	0.274	0.138672	1.24805			

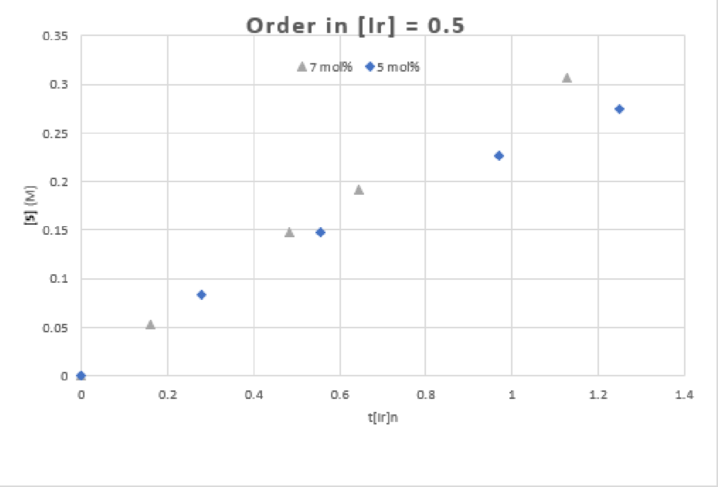

Experiment 2;	[Cat] =	0.02593	7 mol%	[Ir] (0.02593 M)
time (h)		[5]	[cat]^I2	t[cat]^I2
	0	0	0.161028	0
	1	0.05337	0.161028	0.161028
	3	0.148	0.161028	0.483084
	4	0.1915	0.161028	0.644112
	7	0.3071	0.161028	1.127196

Experiment 1;	[Cat] =	0.01923	5 mol%	[Ir] (0.01923 M)	Order in cat =	1	change this value to check other orders
time (h)		[5]	[cat]^I2	t[cat]^I2			
	0	0	0.01923	0			
	2	0.08354	0.01923	0.03846			
	4	0.1477	0.01923	0.07692			
	7	0.2269	0.01923	0.13461			
	9	0.274	0.01923	0.17307			

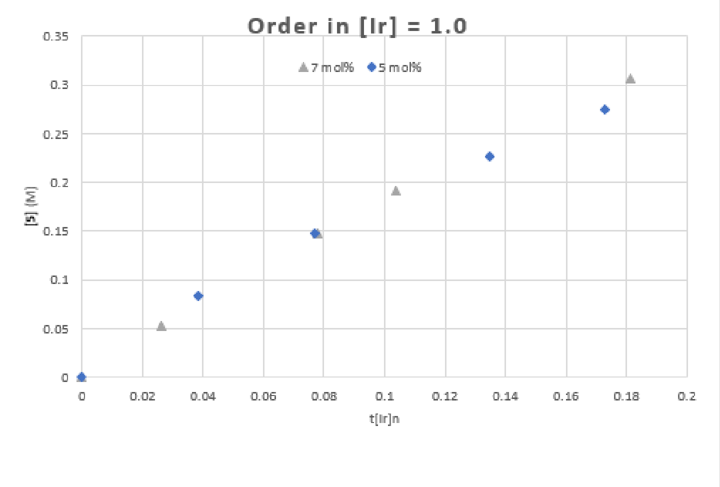

Experiment 2;	[Cat] =	0.02593	7 mol%	[Ir] (0.02593 M)
time (h)		[5]	[cat]^I2	t[cat]^I2
	0	0	0.02593	0
	1	0.05337	0.02593	0.02593
	3	0.148	0.02593	0.07779
	4	0.1915	0.02593	0.10372
	7	0.3071	0.02593	0.18151

**Experiment 1;**	**[Cat] =**	**0.01923**	5 mol%	[Ir] (0.01923 M)	**Order in cat =**	**2**	change this value to check other orders
time (h)		[5]	[cat]^I2	t[cat]^I2			
	0	0	0.00037	0			
	2	0.08354	0.00037	0.00074			
	4	0.1477	0.00037	0.001479			
	7	0.2269	0.00037	0.002589			
	9	0.274	0.00037	0.003328			

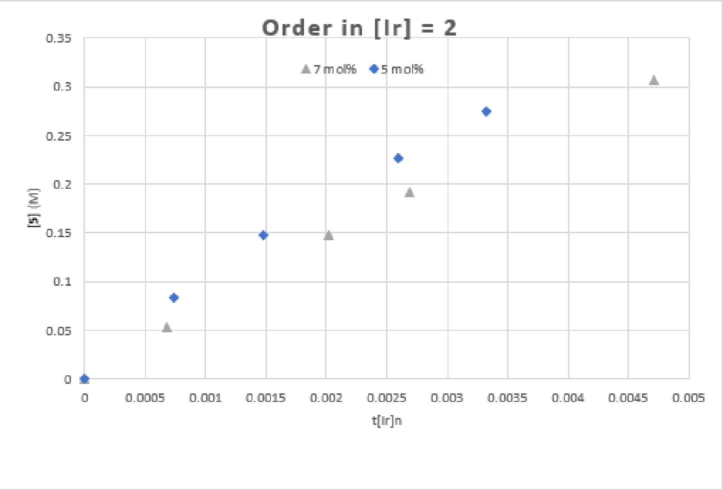

**Experiment 2;**	**[Cat] =**	**0.02593**	7 mol%	[Ir] (0.02593 M)
time (h)		[5]	[cat]^I2	t[cat]^I2
	0	0	0.000672	0
	1	0.05337	0.000672	0.000672
	3	0.148	0.000672	0.002017
	4	0.1915	0.000672	0.002689
	7	0.3071	0.000672	0.004707

7.5 Attempted C-C Bond Formation from 8 in the Absence of Reductant

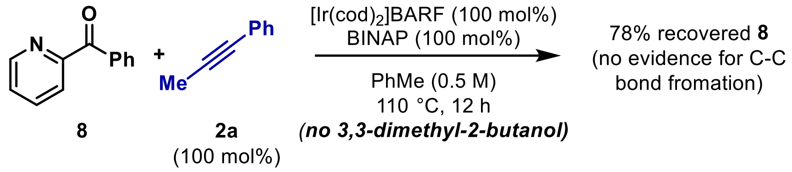

Scheme S22. Attempted C-C bond formation from **8** in the absence of reductant.

A flame-dried *Schlenk* tube was charged with ketone **8** (18.3 mg, 0.10 mmol, 100 mol%), $[Ir(cod)_2]BARF$ (127 mg, 0.10 mmol, 100 mol%) and (±)-**L1** ((±)-BINAP, 62.3 mg, 0.10 mmol, 100 mol%). The *Schlenk* tube was evacuated and backfilled with N_2 for three times. Then toluene (0.2 mL, 0.5 M) was added, followed by the addition of 1-phenyl-1-propyne (**2a**, 12.6 μL, 11.6 mg, 0.10 mmol, 100 mol%). The tube was sealed and stirred at 110 °C in a heating plate for 12 hours. After cooling to room temperature, the solvent was removed under reduced pressure. Purification by flash column chromatography on silica gel (hexane/ethyl acetate from 100/1 to 19/1) afforded **8** (14.2 mg, 78% yield) as a colorless solid.

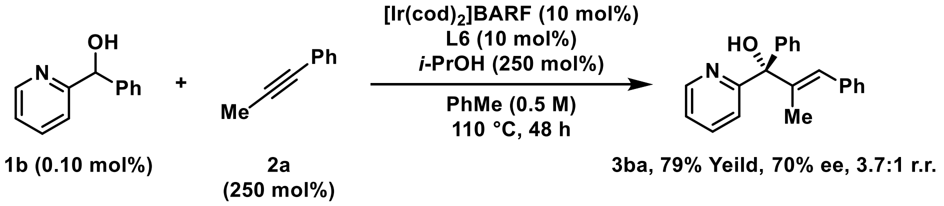

Scheme S23. Iridium-catalyzed enantioselective C-H alkenylations of **1b** with **2a**.

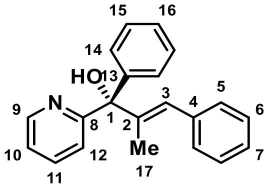

$C_{21}H_{19}NO$
M = 301.39

(*S*,*E*)-2-Methyl-1,3-diphenyl-1-(pyridin-2-yl)prop-2-en-1-ol (**3ba**, **GP 6**) was prepared from phenyl(pyridin-2-yl)methanol (**1b**, 18.5 mg, 0.10 mmol, 100 mol%) and 1-phenyl-1-propyne (**2a**, 31.6 μL, 29.0 mg, 0.25 mmol, 250 mol%), using $[Ir(cod)_2]BARF$ (12.7 mg, 10 μmol, 10 mol%), **L6** (3.34 mg, 10 μmol, 10 mol%) and *i*-PrOH (19.1 μL, 15.0 mg, 0.25 mmol, 250 mol%) in toluene (0.2 mL, 0.5 M) at 110 °C for 48 h. Purification by flash column chromatography on silica gel (hexane/ethyl acetate from 100/1 to 19/1) afforded **3ba** (23.8 mg, 79% yield, 70% ee, >25:1 r.r.) as a colorless oil. The regiomer ratio (3.7:1 r.r.) of the process was determined from the ^1H NMR spectrum of crude material.

R_f = 0.5 (Hexane/EtOAc = 9/1).

^{1}H NMR (500 MHz, $CDCl_3$, 298 K): δ 8.62 (d, *J* = 4.8 Hz, 1H, H-9), 7.70–7.65 (m, 1H, H-11), 7.54–7.50 (m, 2H, H-14), 7.39–7.34 (m, 2H, H-15), 7.34–7.27 (m, 4H, H-6, H-12 and H-16), 7.27–7.23 (m, 3H, H-5 and H-10), 7.23–7.19 (m, 1H, H-7), 6.17 (s, 1H, OH), 6.08 (s, 1H, H-3), 1.95 (s, 3H, H-17) ppm.

^{13}C NMR (125 MHz, $CDCl_3$, 298 K): δ 162.0 (C-8), 148.0 (C-9), 144.4 (C-13), 143.1 (C-2), 137.9 (C-4), 136.5 (C-11), 129.6 (C-3), 129.2 (Ar), 128.2 (Ar), 128.1 (Ar), 127.9 (Ar), 127.4 (Ar), 126.7 (Ar), 123.0 (Ar), 122.5 (Ar), 82.9 (C-1), 16.1 (C-7) ppm.

HRMS (ESI) m/z: $[M+H]^+$ calcd for $C_{21}H_{20}NO^+$ 302.1540, found 302.1541.

IR (thin film): ṽ 3373 (br), 2922 (w), 2857 (w), 1590 (s), 1431 (s), 1374 (s), 1152 (s), 1038 (s) cm^{-1}.

Specific rotation: $[\alpha]_D^{23}$ = +22.7 (*c* 0.5, CH_2Cl_2).

The **enantiomeric ratio** of **3ba** was determined by SFC analysis (CHIRALPACK OD-H (25 cm), column temperature 25 °C, solvent CO_2/MeOH (with 0.5% Et_3N) = 99/1, flow rate = 2.0 mL/min): t_R = 26.7 min (major), t_R = 31.0 min (minor).

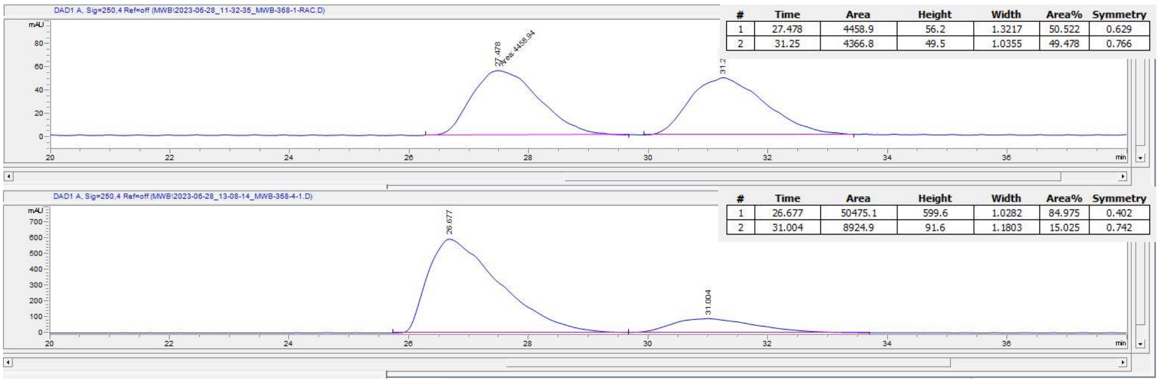

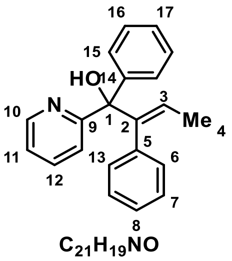

$C_{21}H_{19}NO$
M = 301.39

(*E*)-1,2-diphenyl-1-(pyridin-2-yl)but-2-en-1-ol (*iso*-**3ba**, **GP 6**)

R*f* = 0.5 (Hexane/EtOAc = 9/1).

^{1}H NMR (500 MHz, $CDCl_3$, 298 K): δ 8.51 (d, *J* = 4.8 Hz, 1H, H-10), 7.64–7.59 (m, 1H, H-12), 7.58–7.54 (m, 2H, H-15), 7.37 (d, *J* = 7.9 Hz, 1H, H-13), 7.34–7.30 (m, 2H, H-16), 7.27–7.22 (m, 1H, H-17), 7.20–7.11 (m, 4H, H-7, H-8 and H-11), 7.08–7.04 (m, 2H, H-6), 6.04 (s, 1H, OH), 5.50 (q, *J* = 6.8 Hz, 1H, H-3), 1.52 (d, *J* = 6.8 Hz, 3H, H-4) ppm.

^{13}C NMR (125 MHz, $CDCl_3$, 298 K): δ 162.0 (C-9), 147.7 (C-10), 146.6 (C-2), 144.9 (C-14), 138.7 (C-5), 136.1 (C-12), 130.5 (C-6), 128.1 (Ar), 127.9 (Ar), 127.9 (Ar), 127.6 (Ar), 127.2 (Ar), 126.6 (Ar), 123.4 (C-13), 122.3 (C-11), 82.3 (C-1), 15.3 (C-4) ppm.

8 NMR Spectra

^{1}H NMR (500 MHz, CD_3Cl, 298 K) and ^{13}C NMR (125 MHz, CD_3Cl, 298 K) of **1h**.

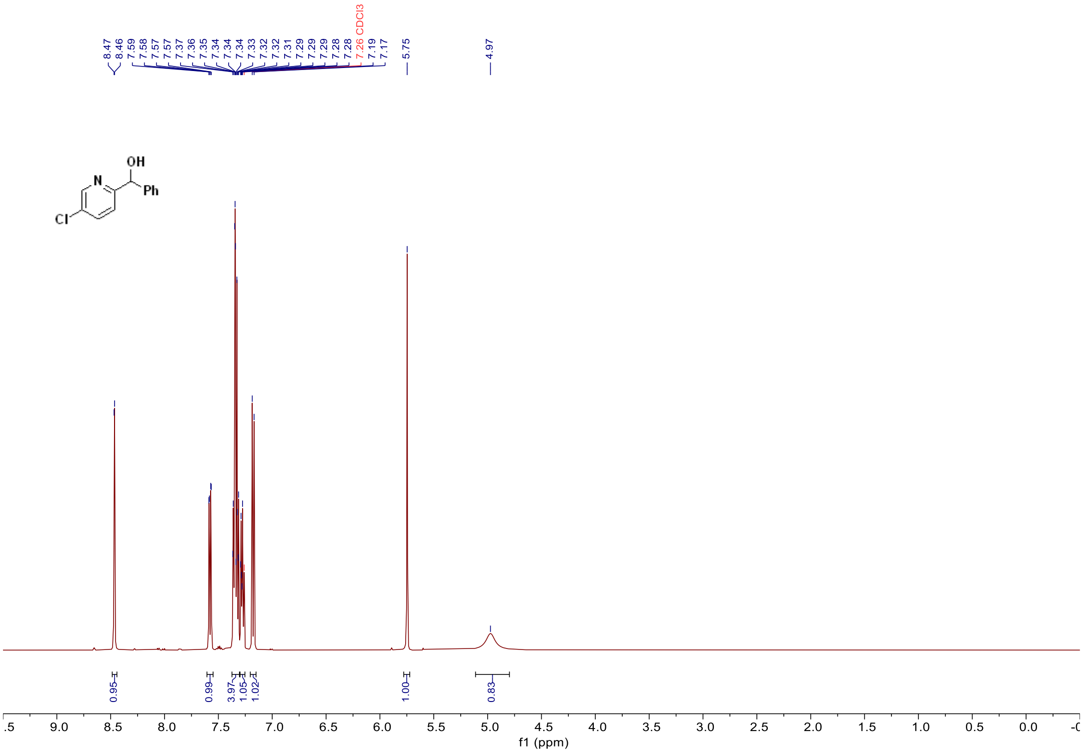

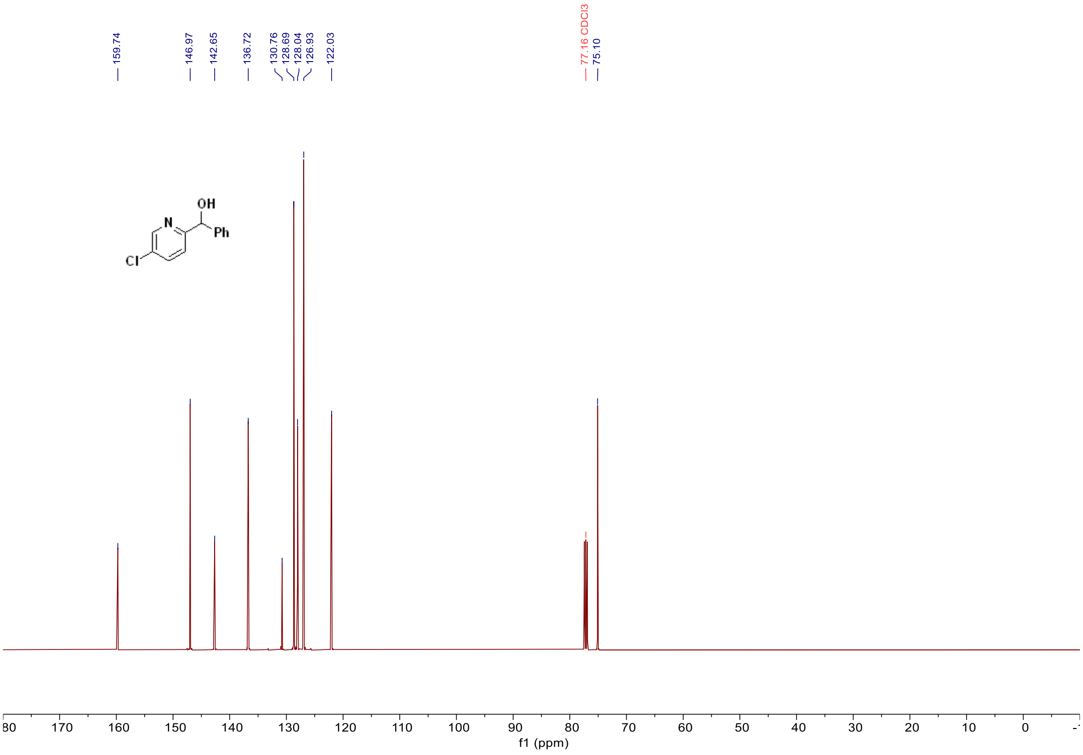

^{1}H NMR (500 MHz, CD_3Cl, 298 K), ^{13}C NMR (125 MHz, CD_3Cl, 298 K) and ^{19}F NMR (471 MHz, CD_3Cl, 298 K) of **1j**.

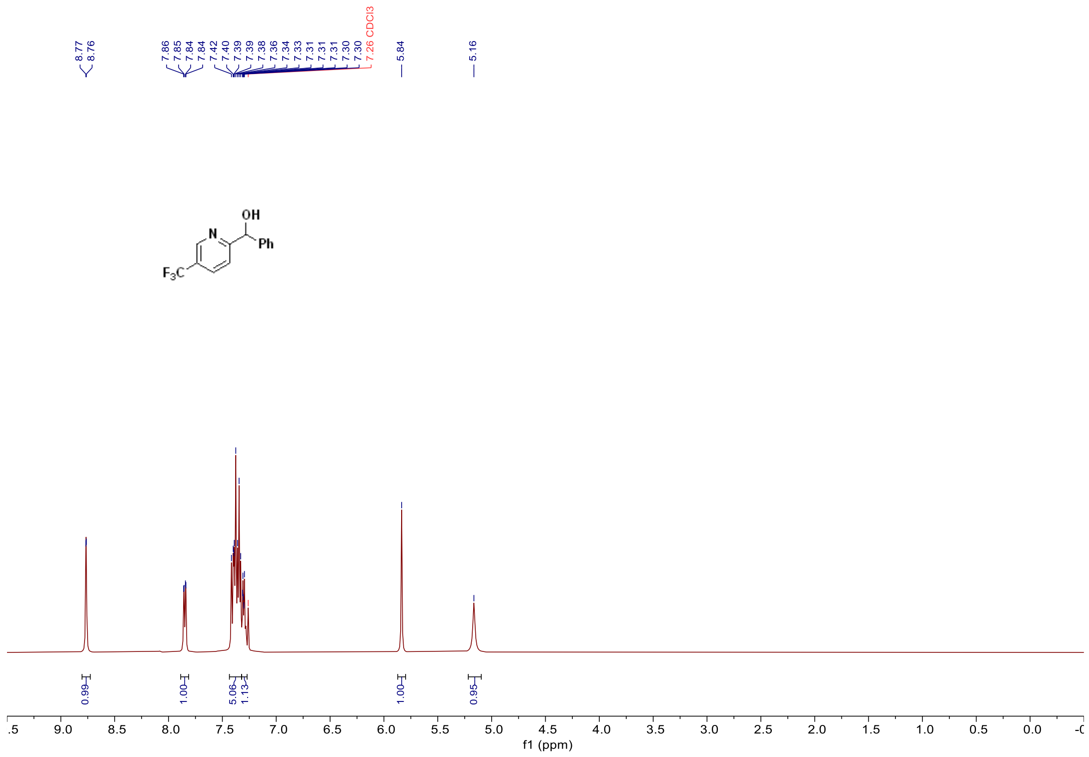

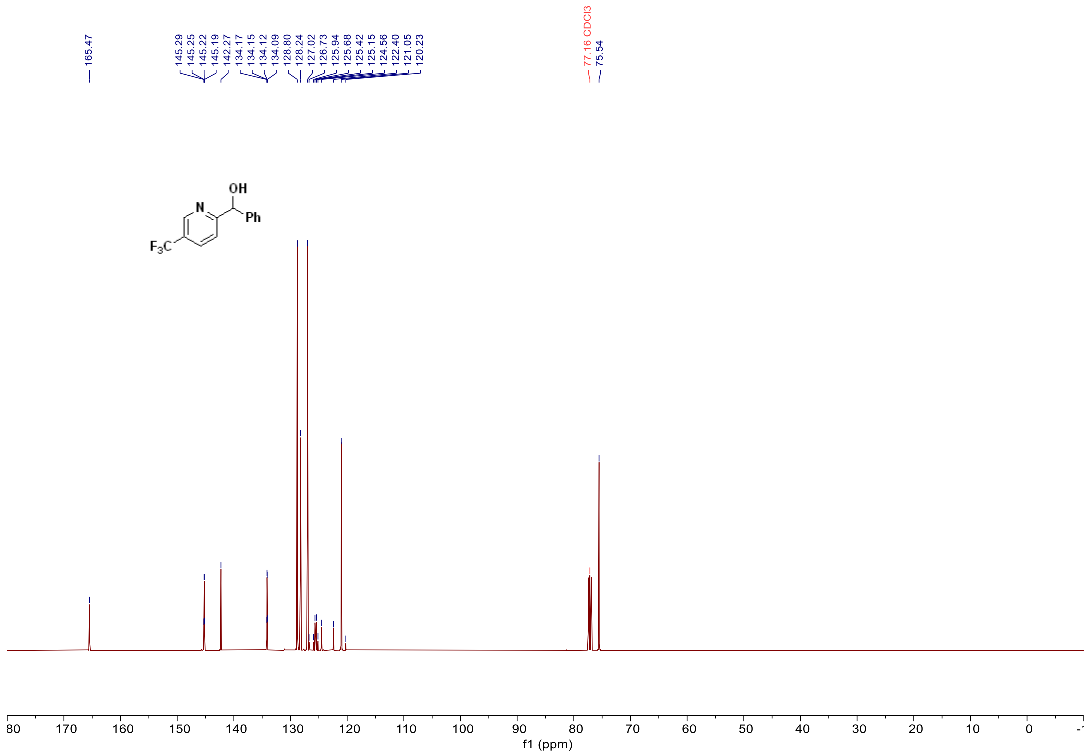

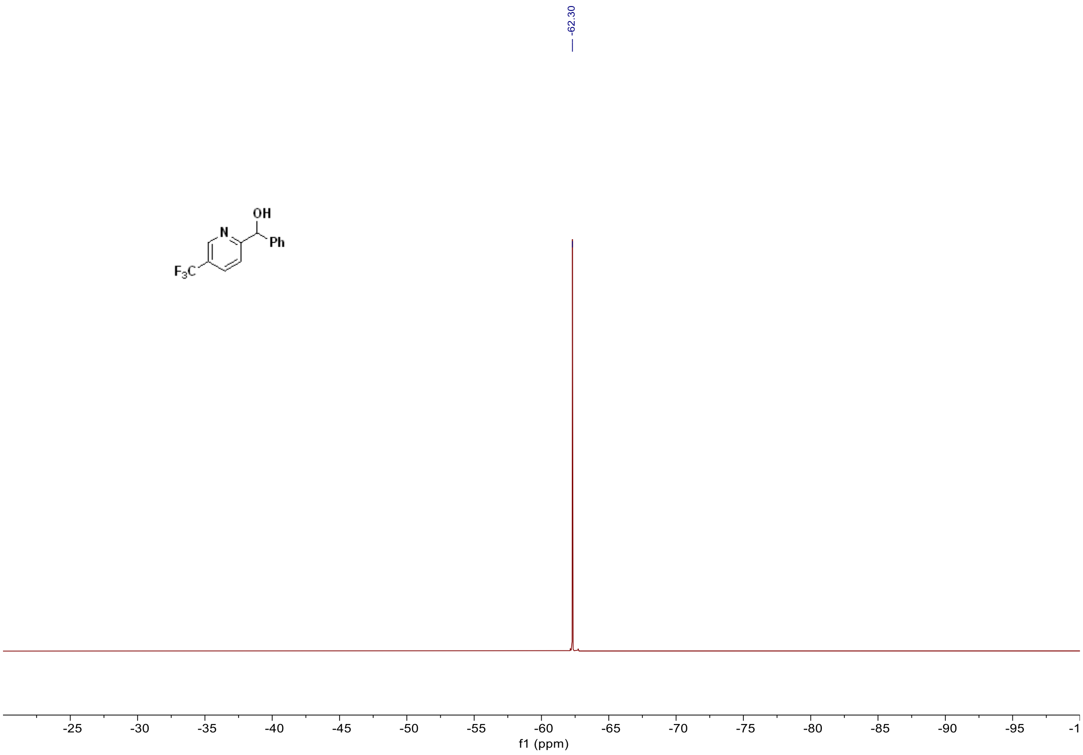
-62.30
OH
N
Ph
F3C
-25
-30
-35
-40
-45
-50
-55
-60
-65
-70
-75
-80
-85
-90
-95
f1 (ppm)

^{1}H NMR (500 MHz, CD_3Cl, 298 K) and ^{13}C NMR (125 MHz, CD_3Cl, 298 K) of **1k**.

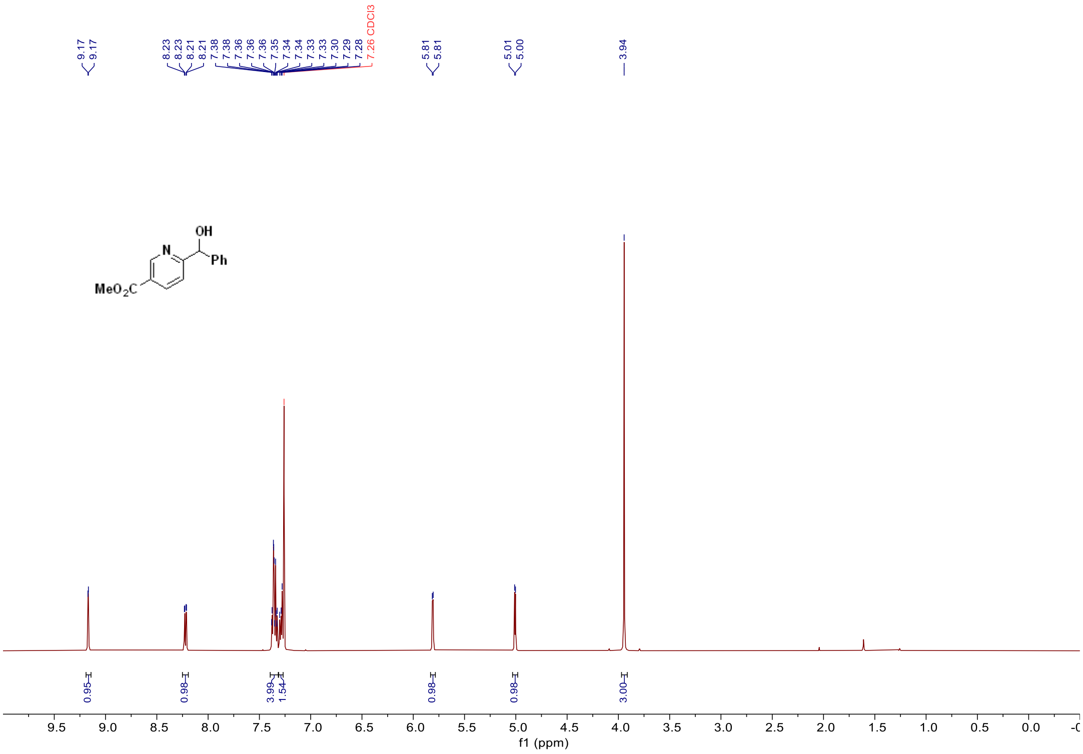

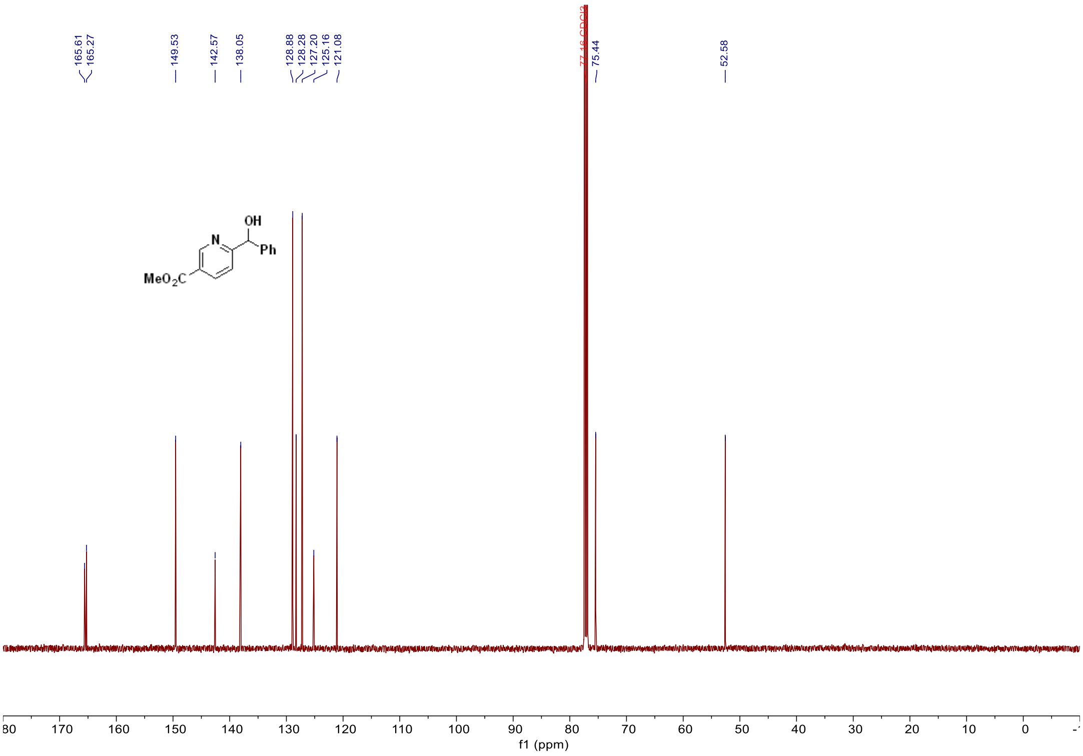

^{1}H NMR (500 MHz, CD_3Cl, 298 K) and ^{13}C NMR (125 MHz, CD_3Cl, 298 K) of **1o**.

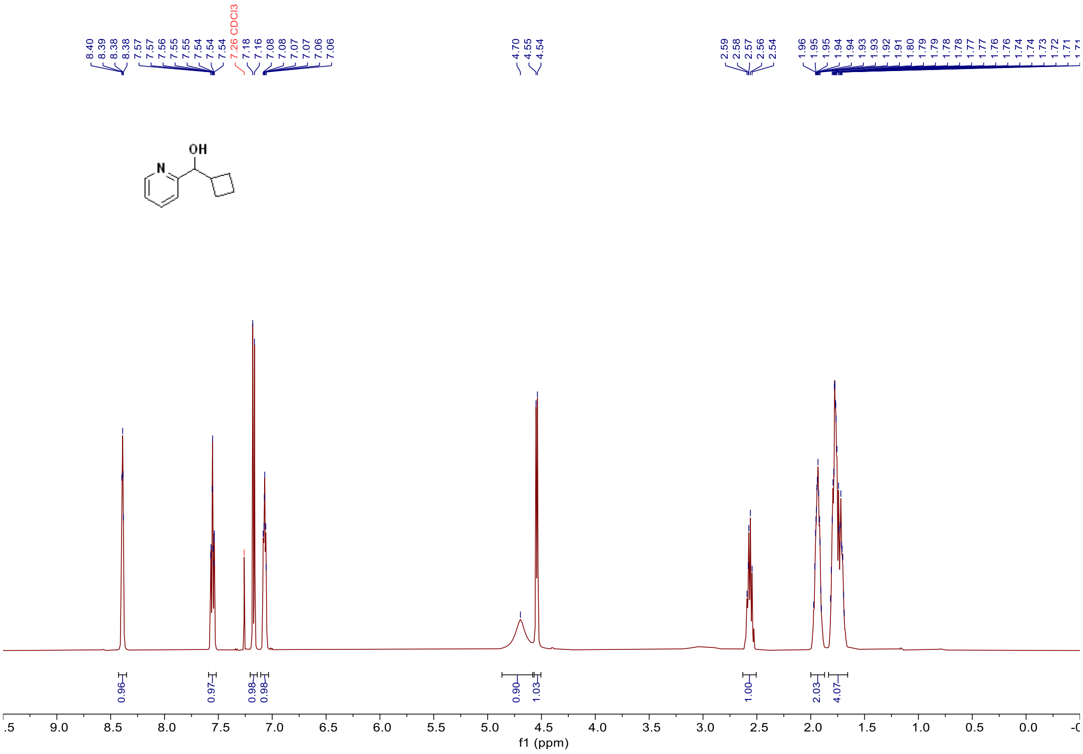

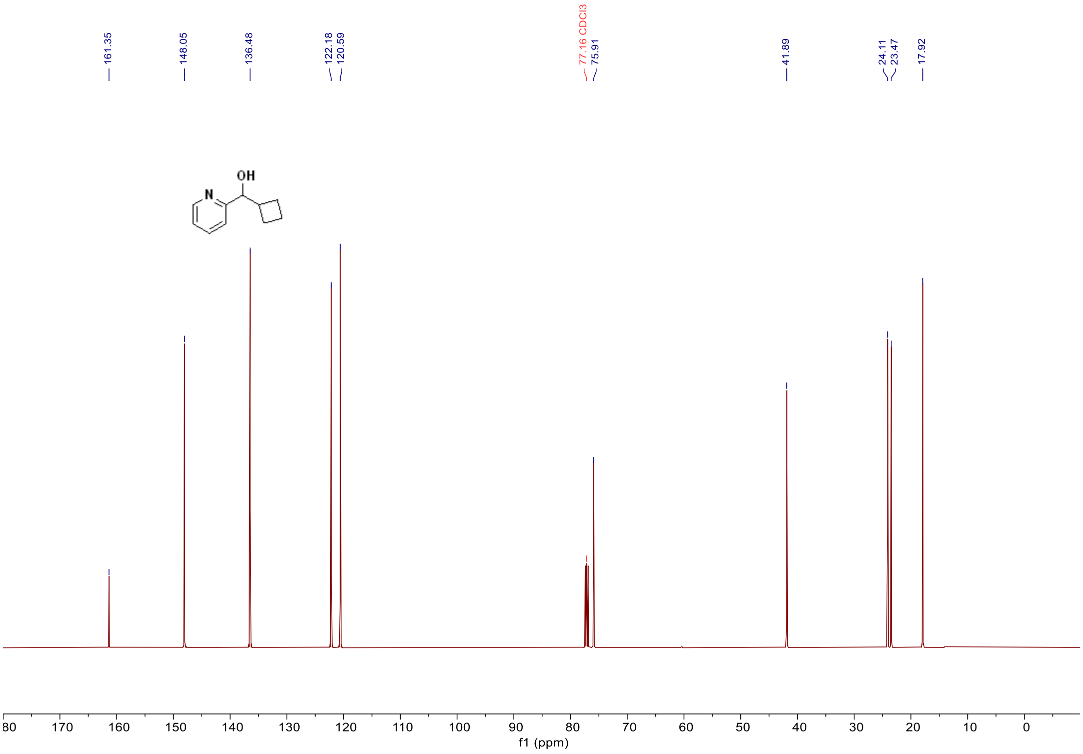

^{1}H NMR (500 MHz, CD_3Cl, 298 K) and ^{13}C NMR (125 MHz, CD_3Cl, 298 K) of **2l**.

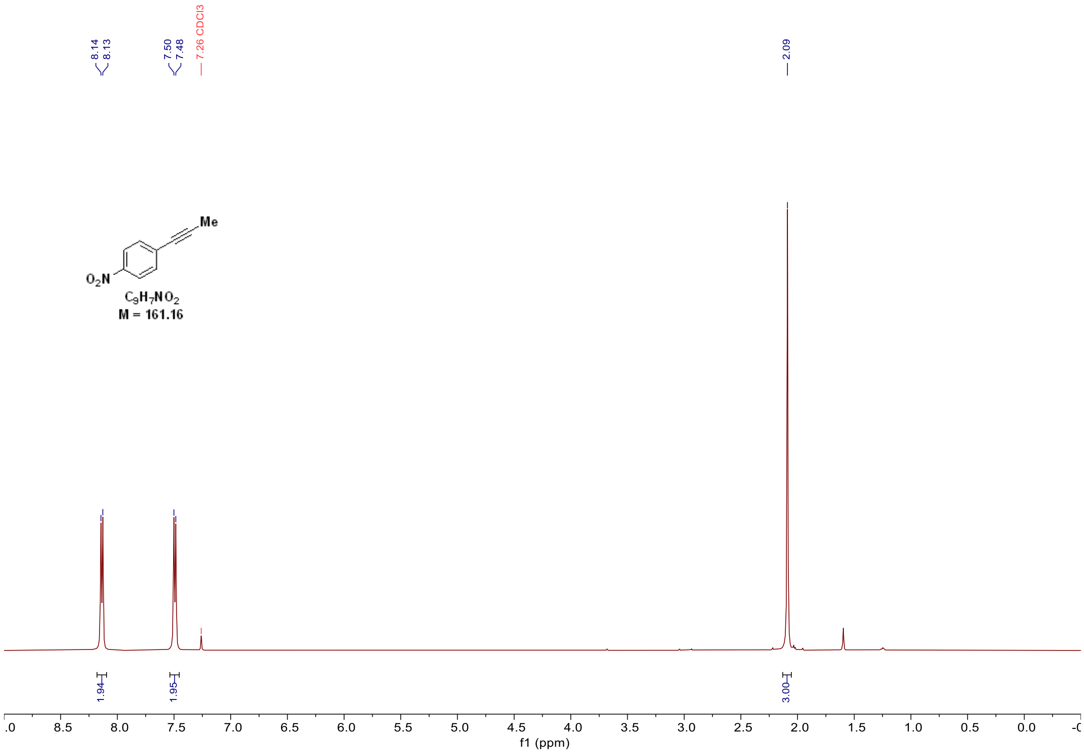

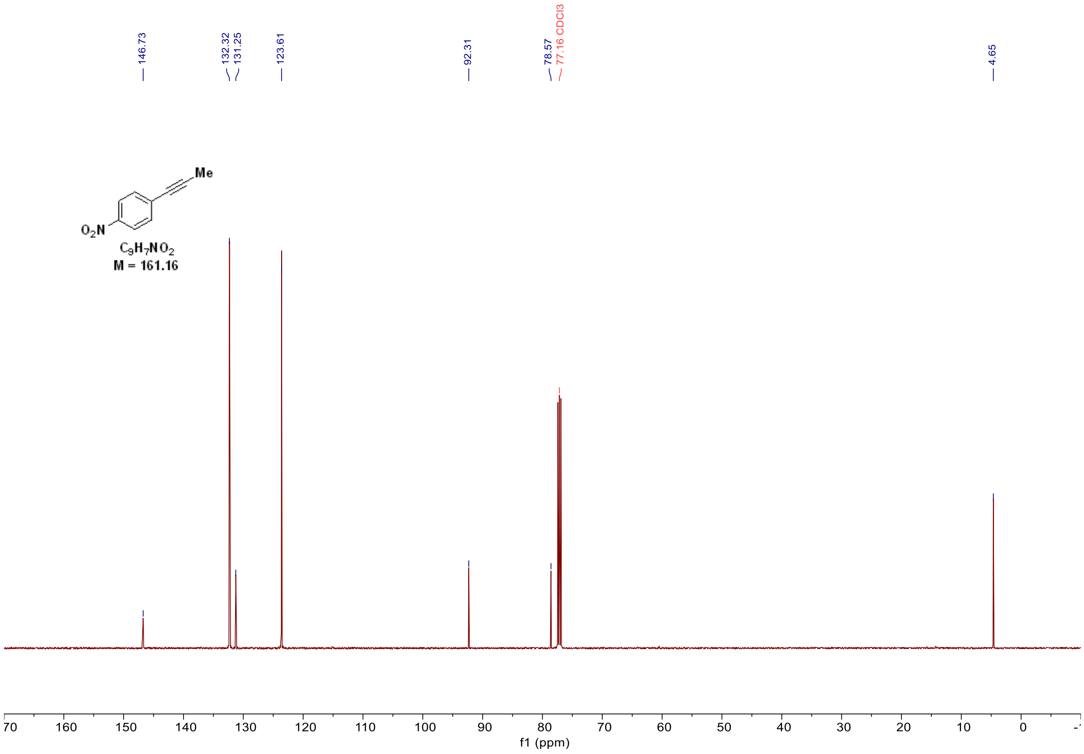

^{1}H NMR (500 MHz, CD_3Cl, 298 K), ^{13}C NMR (125 MHz, CD_3Cl, 298 K) and ^{19}F NMR (471 MHz, CD_3Cl, 298 K) of $[Ir(cod)_2]BARF$

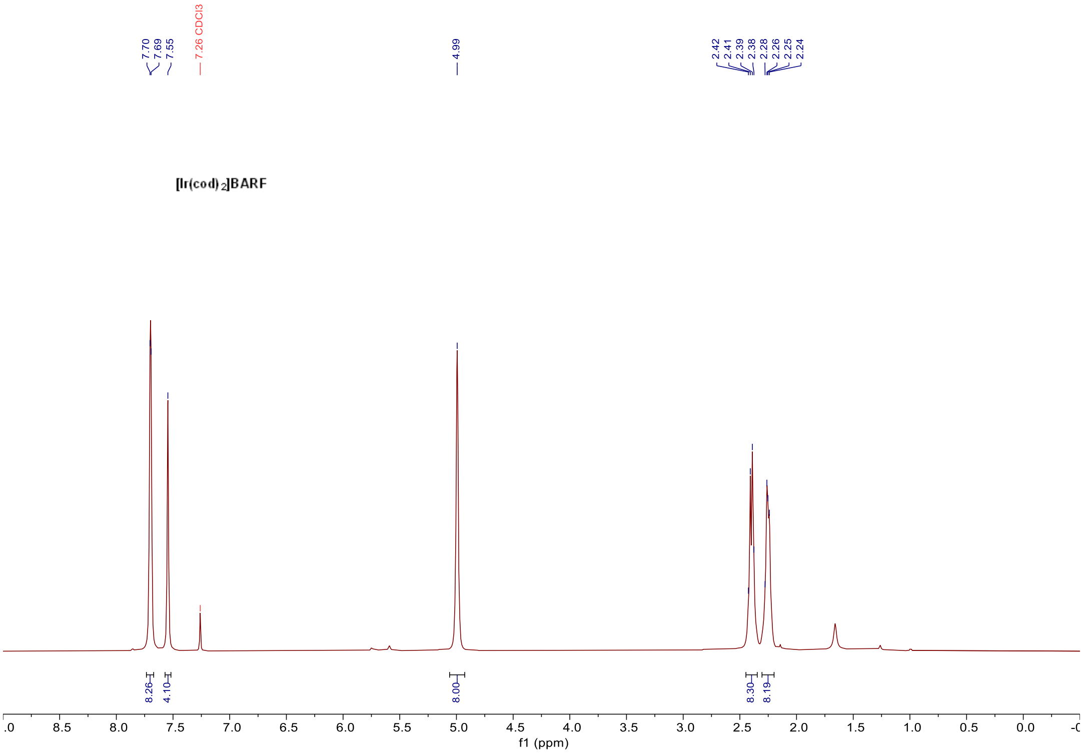

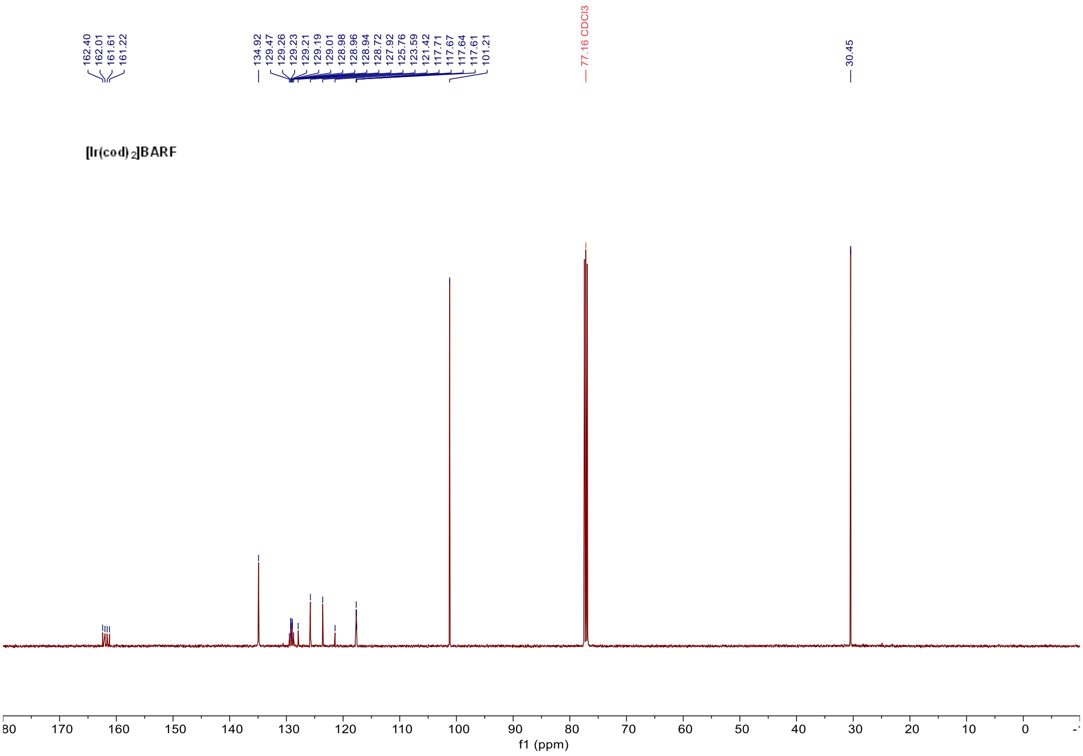

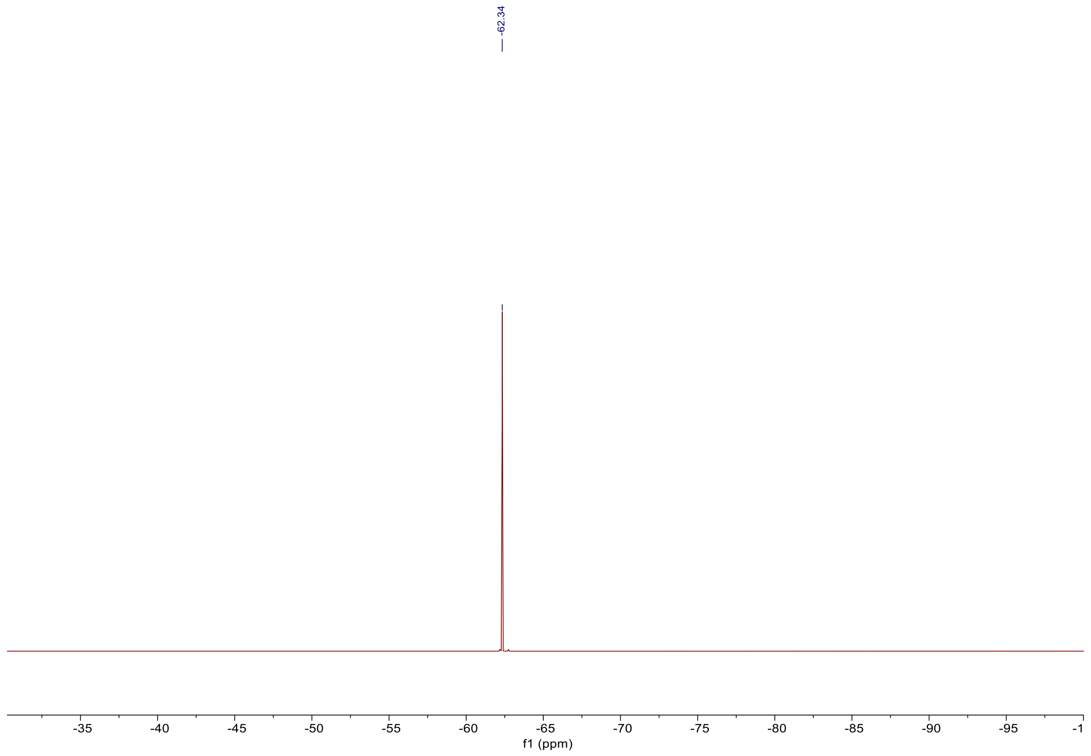
-62.34
-35
-40
-45
-50
-55
-60
-65
-70
-75
-80
-85
-90
-95
-1
f1 (ppm)

^{1}H NMR (500 MHz, CD_3Cl, 298 K) and ^{13}C NMR (125 MHz, CD_3Cl, 298 K) of **5** (diastereomer 1).

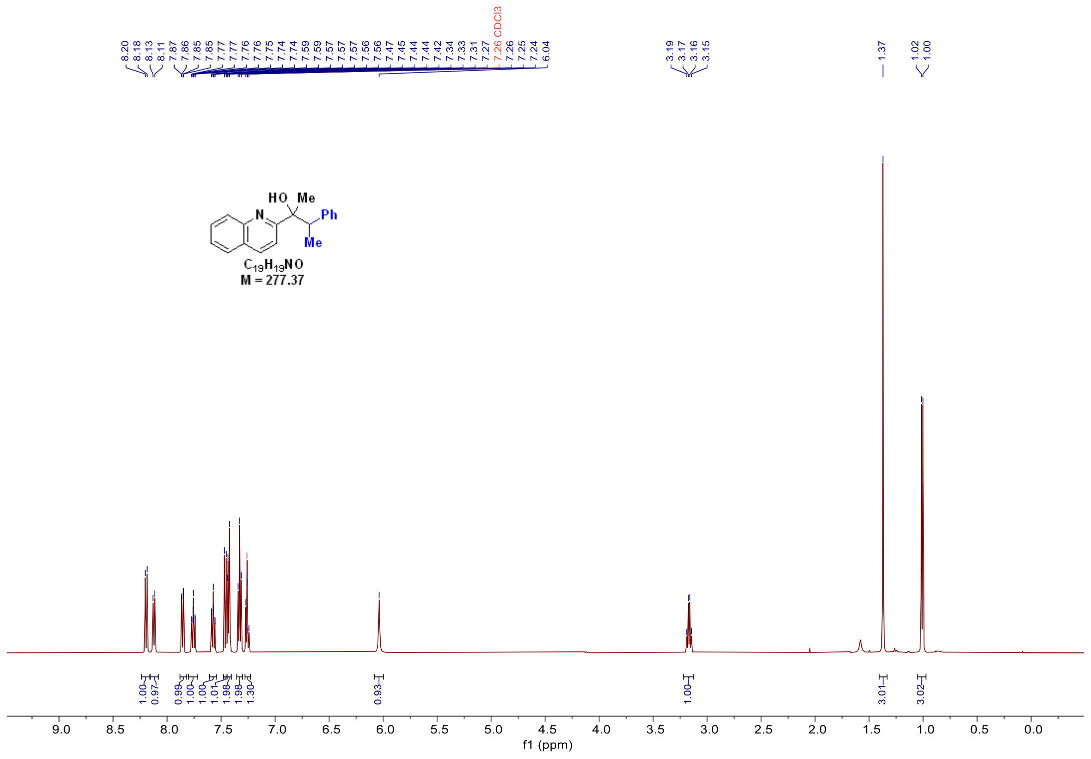

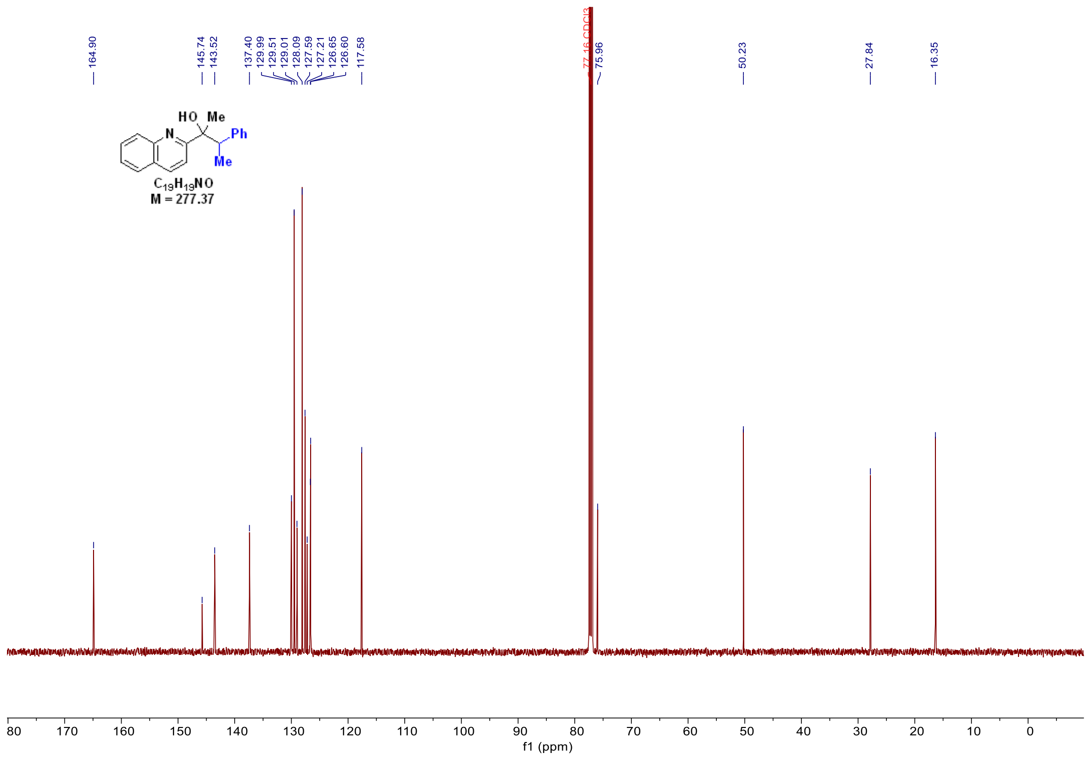

^{1}H NMR (500 MHz, CD_3Cl, 298 K) and ^{13}C NMR (125 MHz, CD_3Cl, 298 K) of **5** (diastereomer 2).

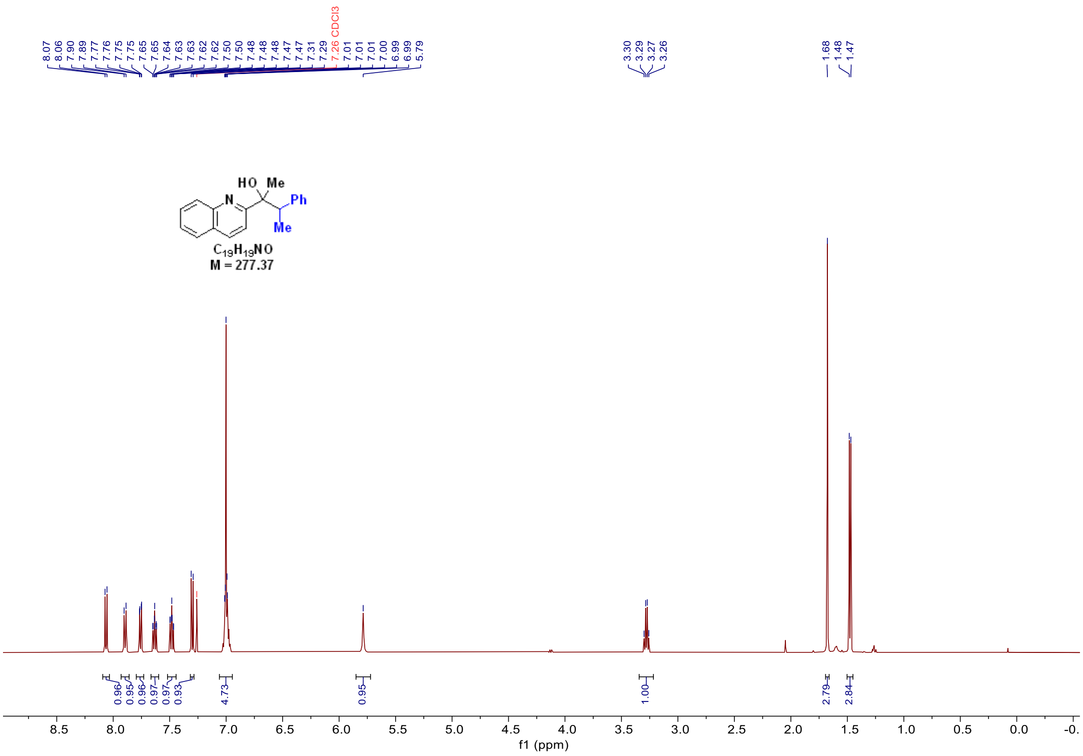

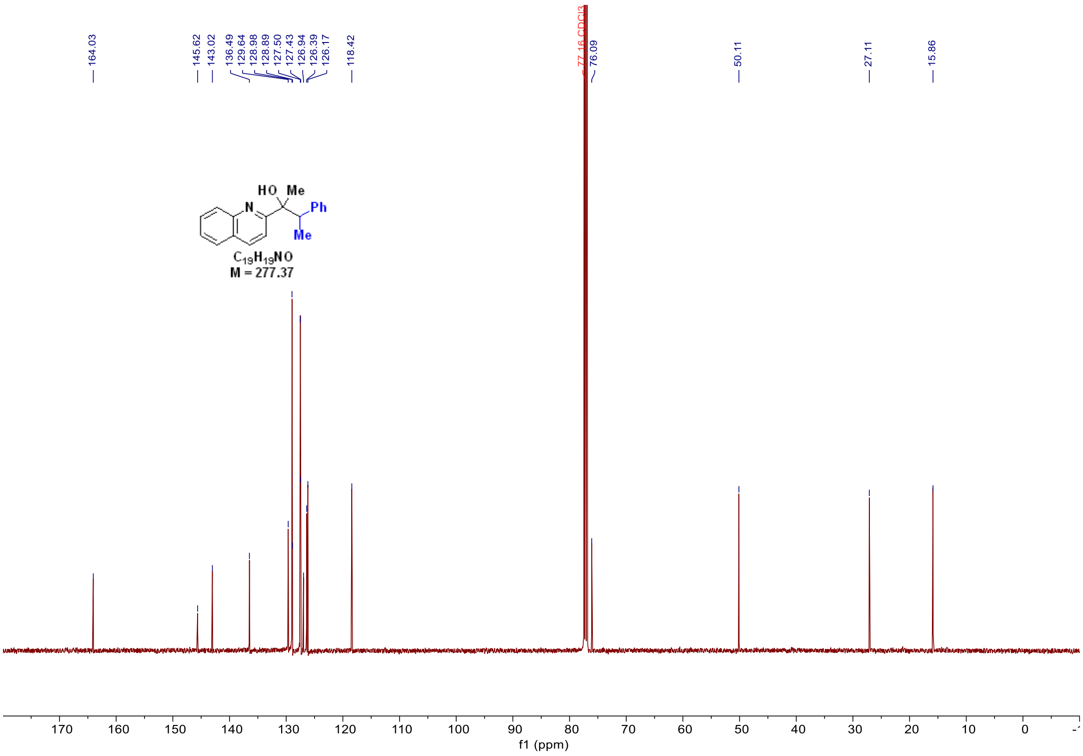

^{1}H NMR (500 MHz, CD_3Cl, 298 K) and ^{13}C NMR (125 MHz, CD_3Cl, 298 K) of **3aa**.

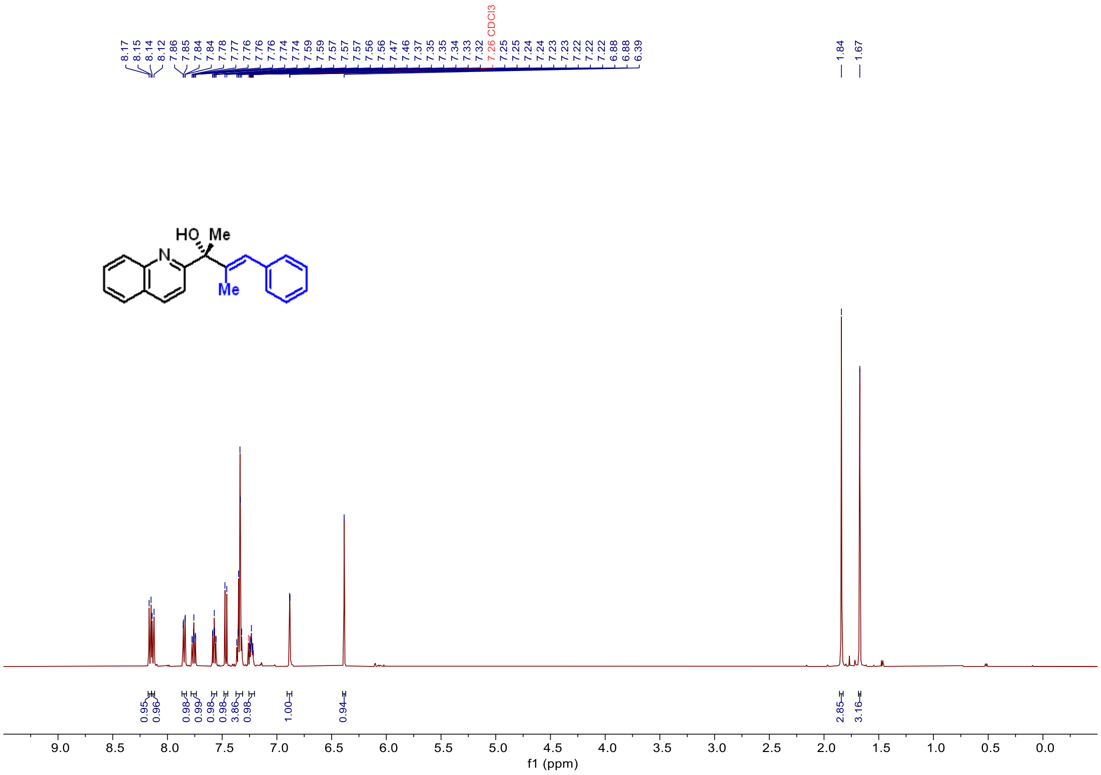

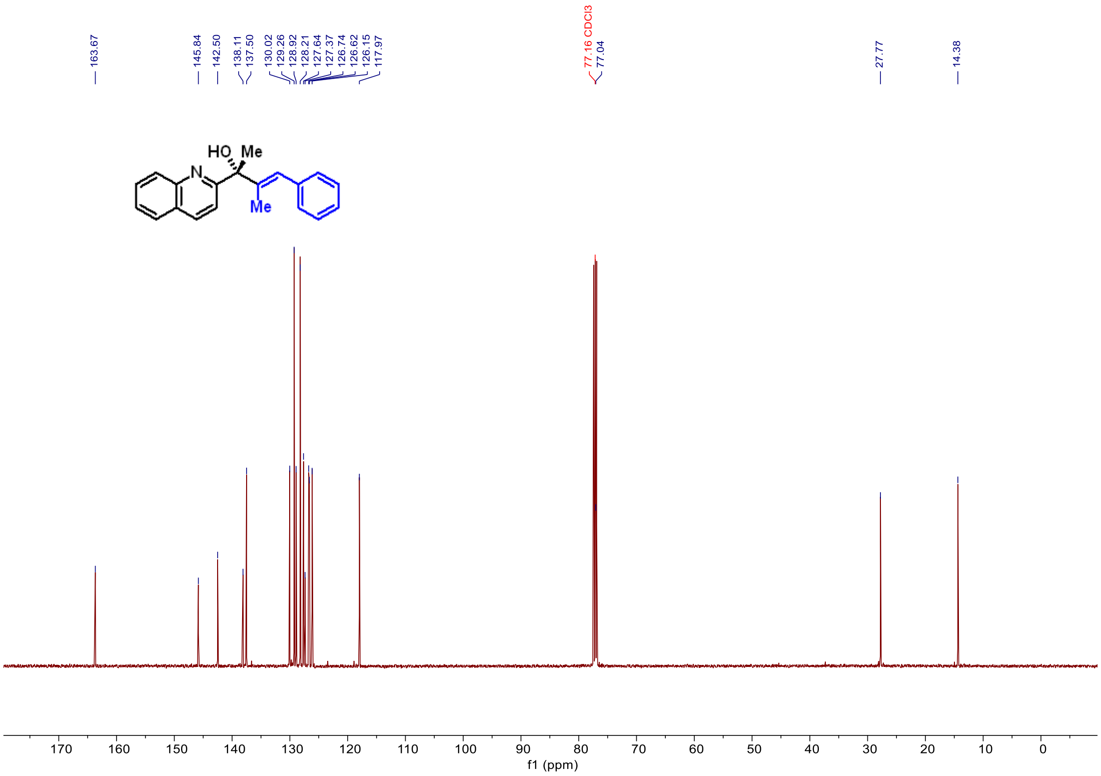

^{1}H NMR (500 MHz, CD_3Cl, 298 K) and ^{13}C NMR (125 MHz, CD_3Cl, 298 K) of **3ab**.

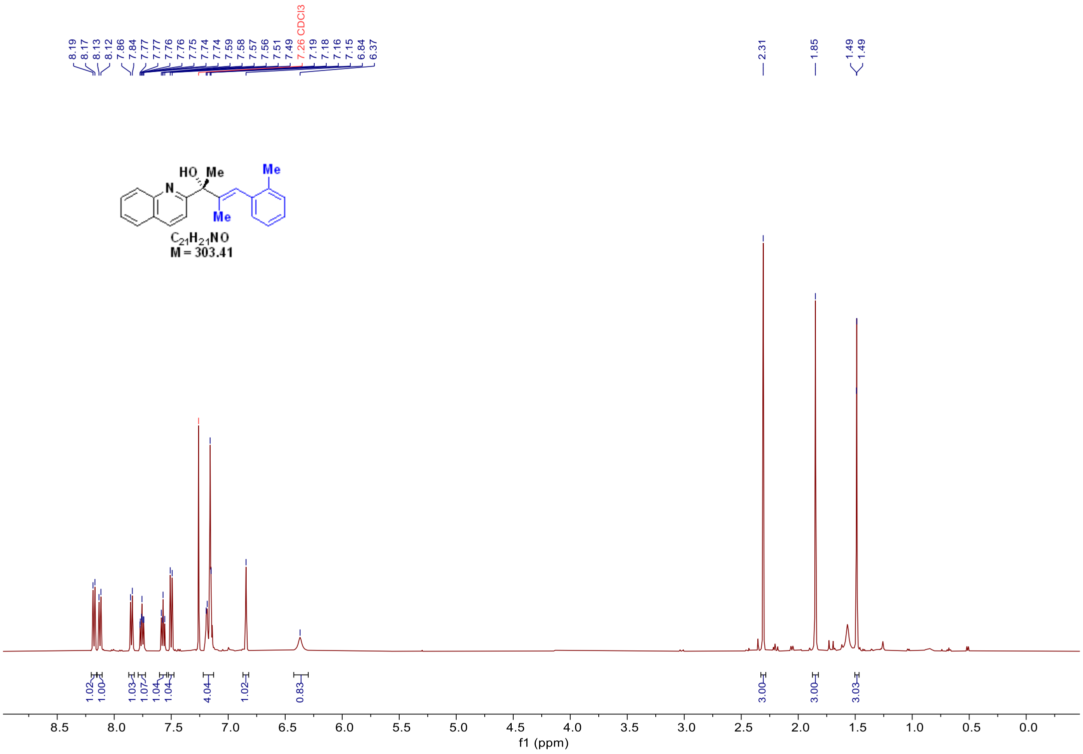

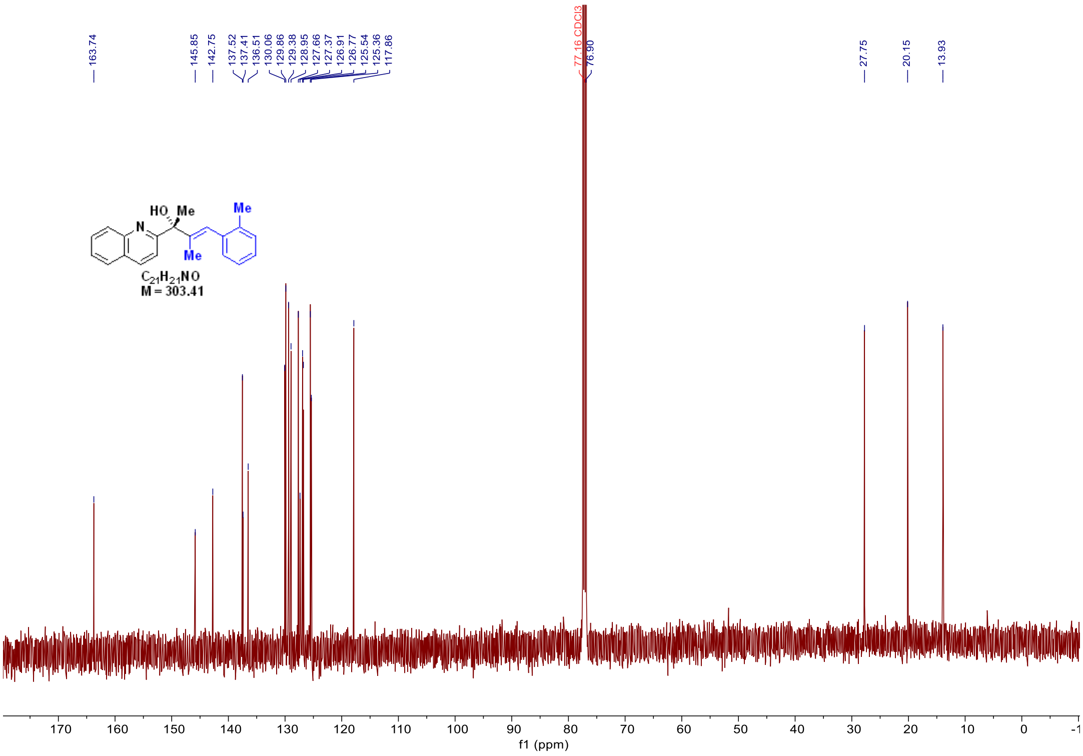

^{1}H NMR (500 MHz, CD_3Cl, 298 K) and ^{13}C NMR (125 MHz, CD_3Cl, 298 K) of **3ac**.

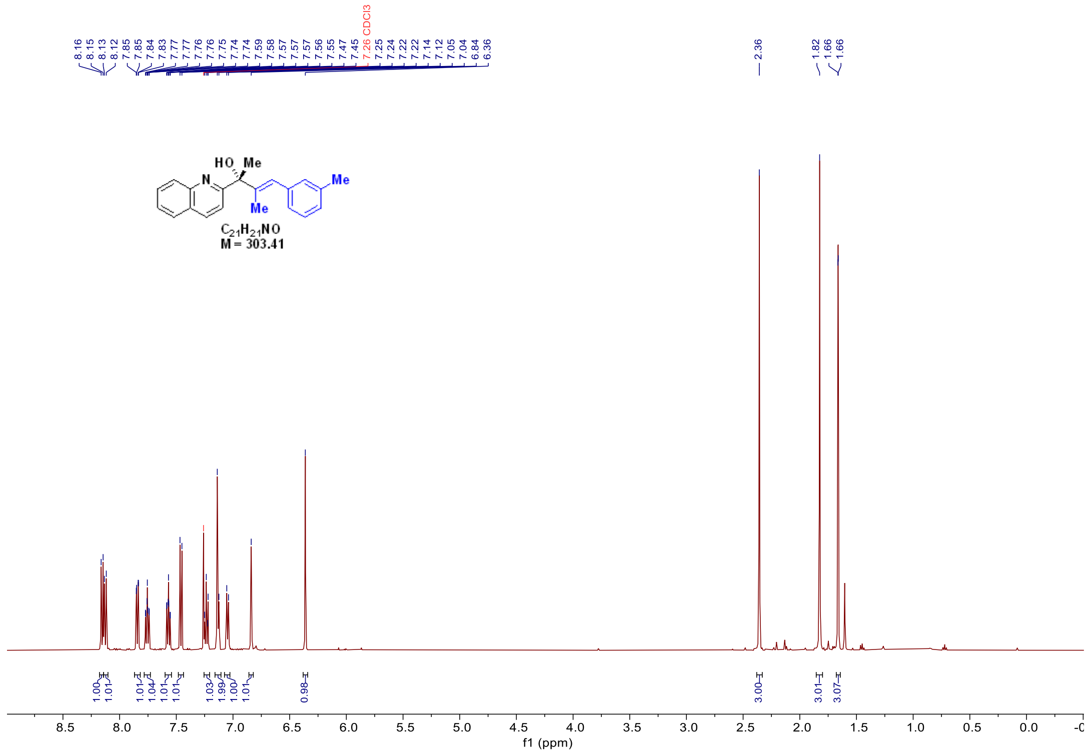

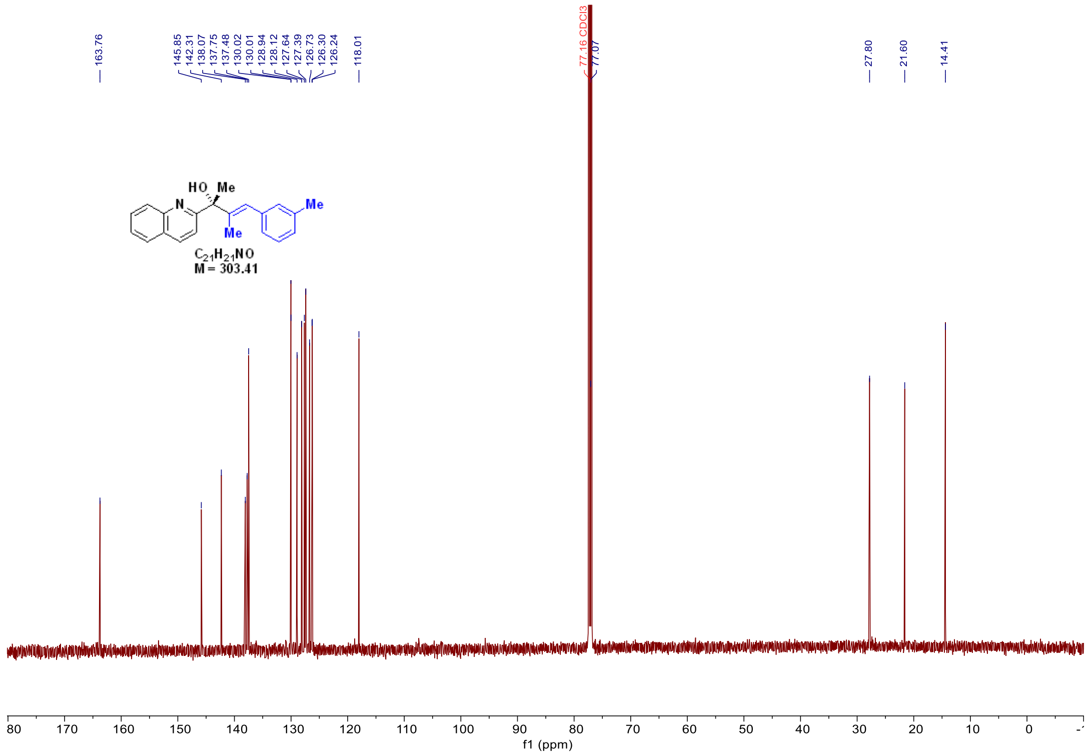

^{1}H NMR (500 MHz, CD_3Cl, 298 K) and ^{13}C NMR (125 MHz, CD_3Cl, 298 K) of **3ad**.

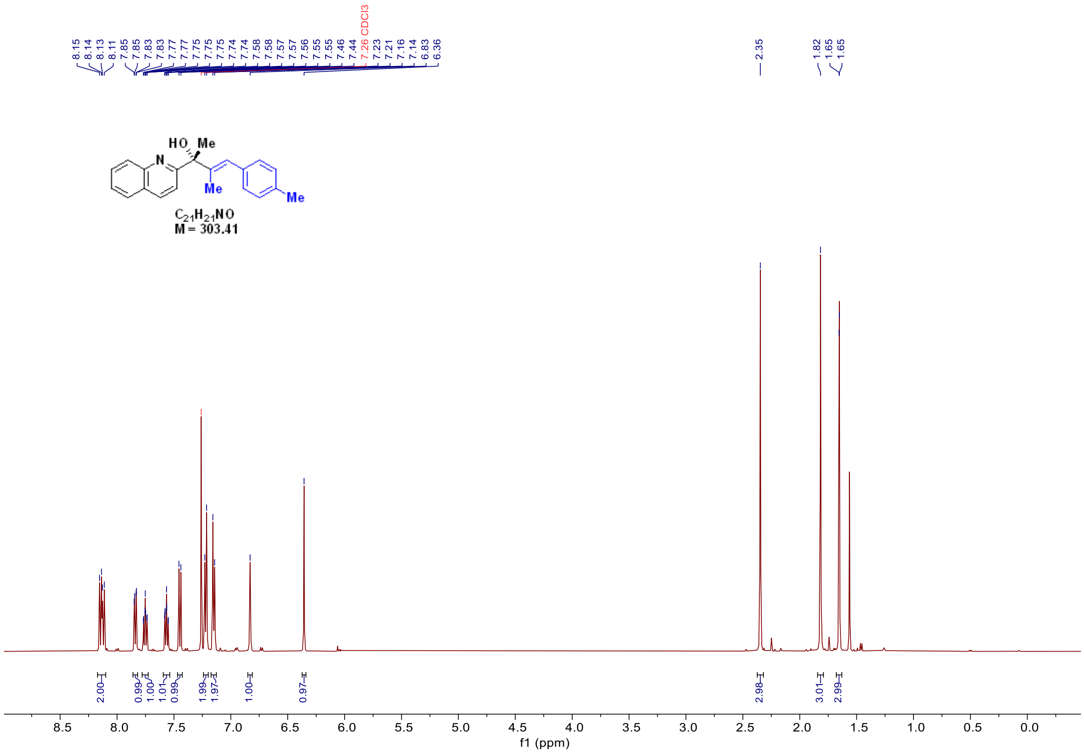

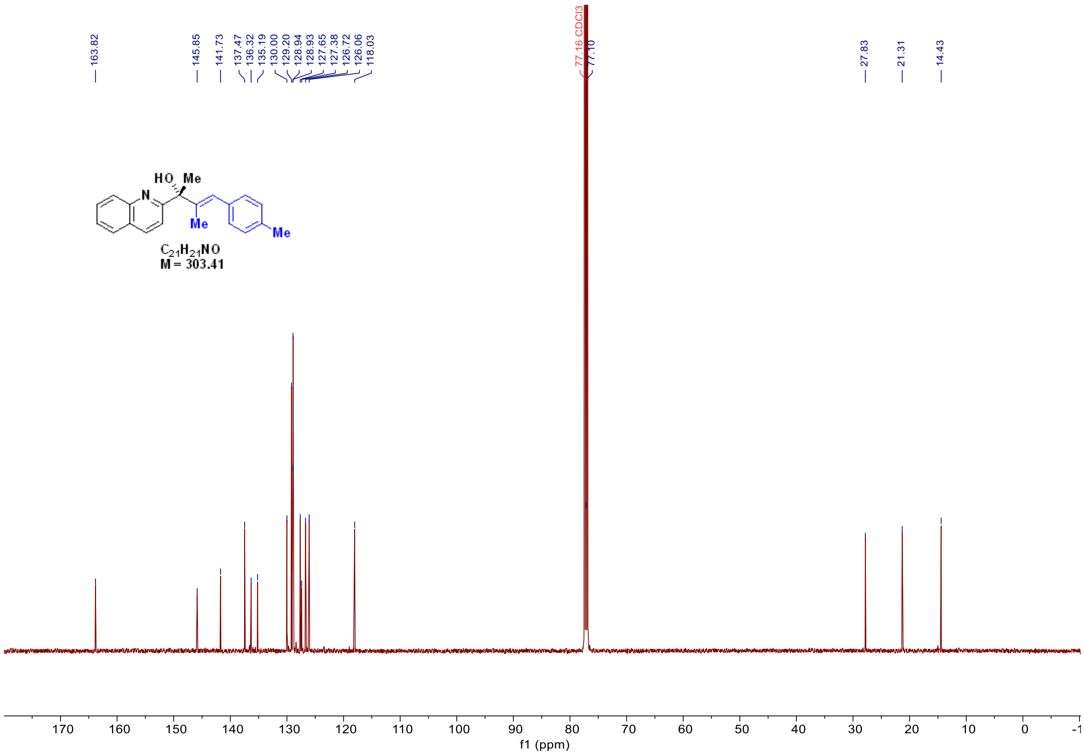

^{1}H NMR (500 MHz, CD_3Cl, 298 K) and ^{13}C NMR (125 MHz, CD_3Cl, 298 K) of **3ae**.

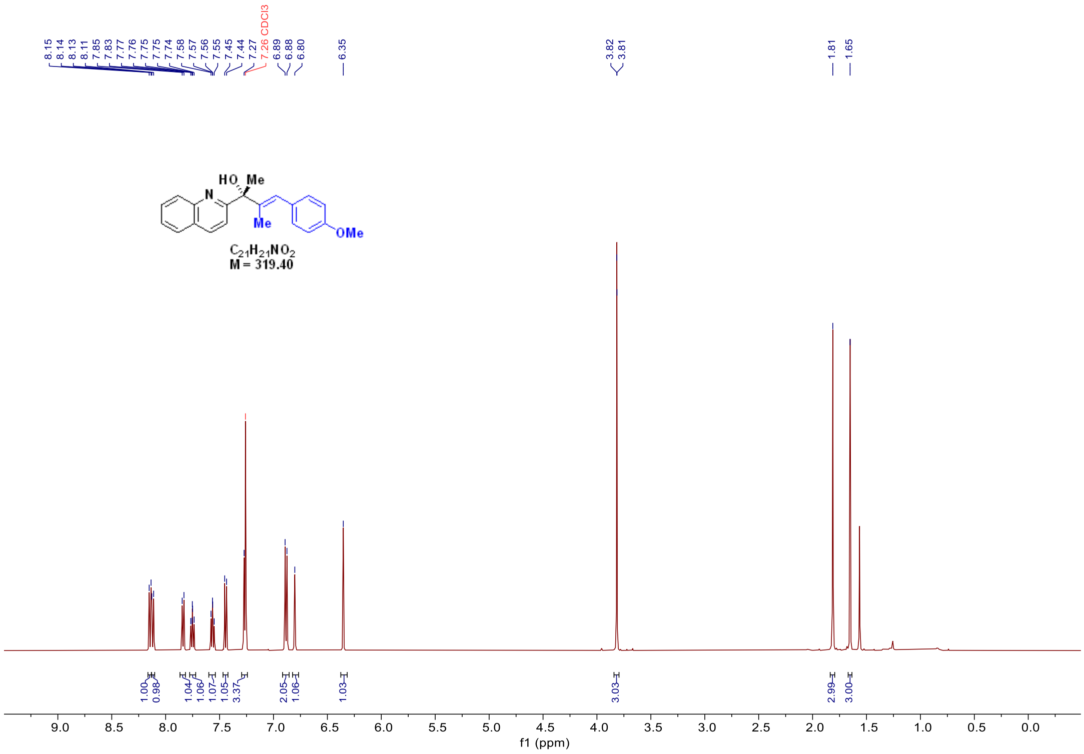

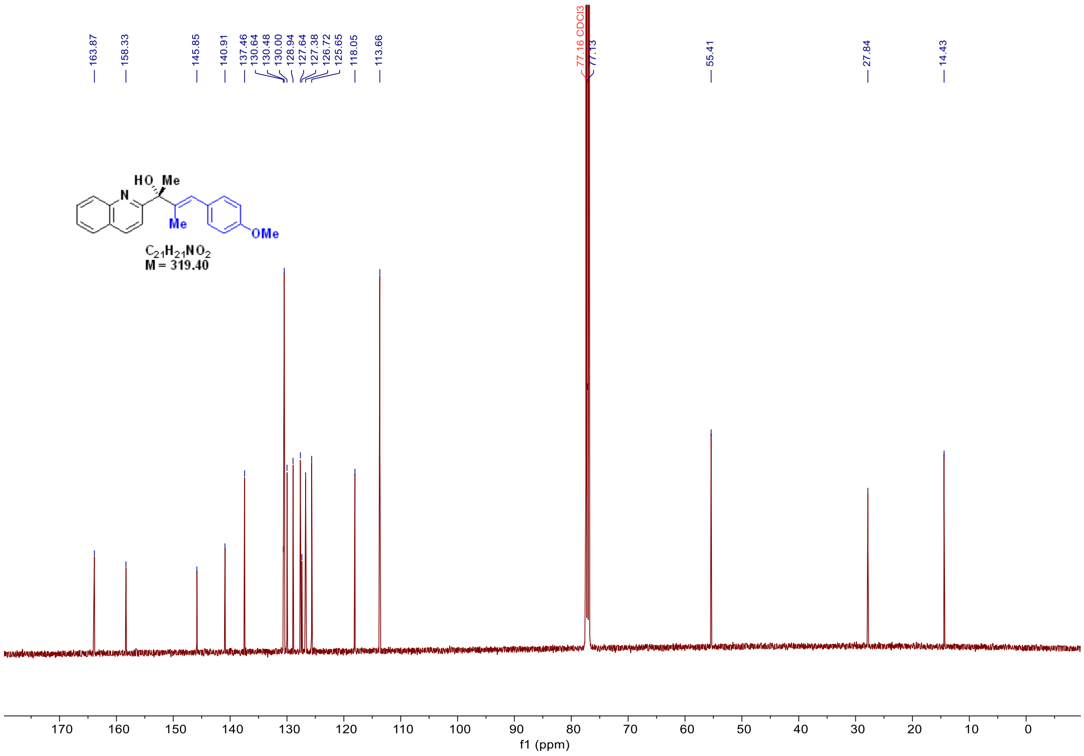

^{1}H NMR (500 MHz, CD_3Cl, 298 K), ^{13}C NMR (125 MHz, CD_3Cl, 298 K) and ^{19}F NMR (471 MHz, CD_3Cl, 298 K) of **3af**.

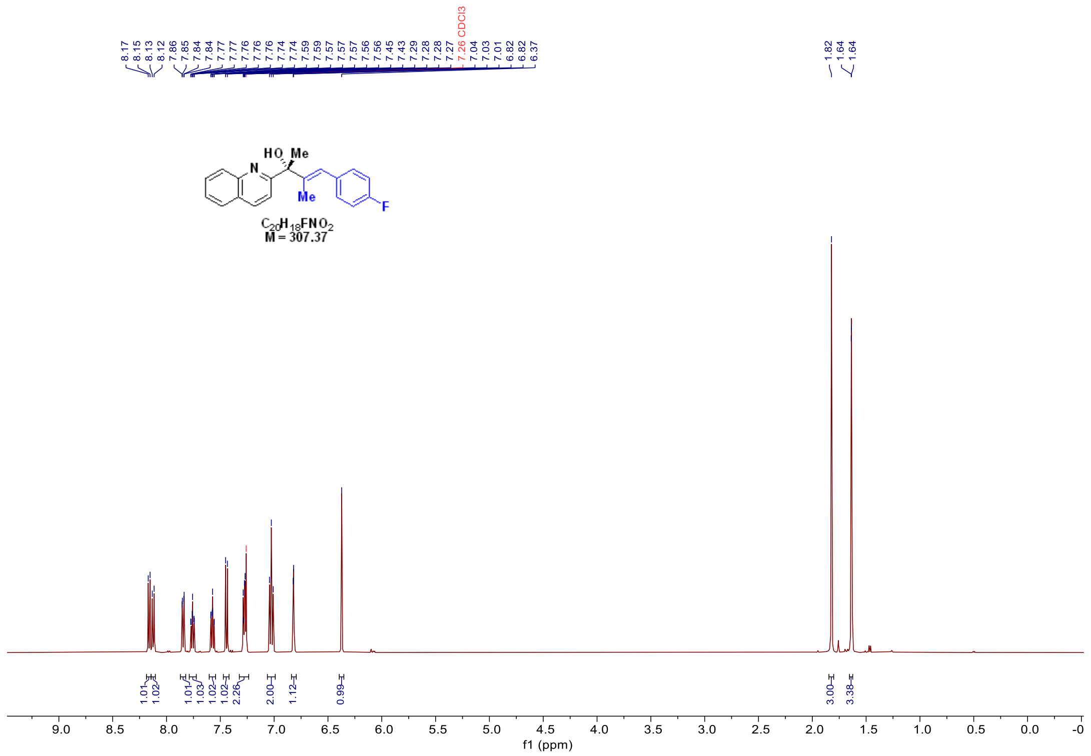

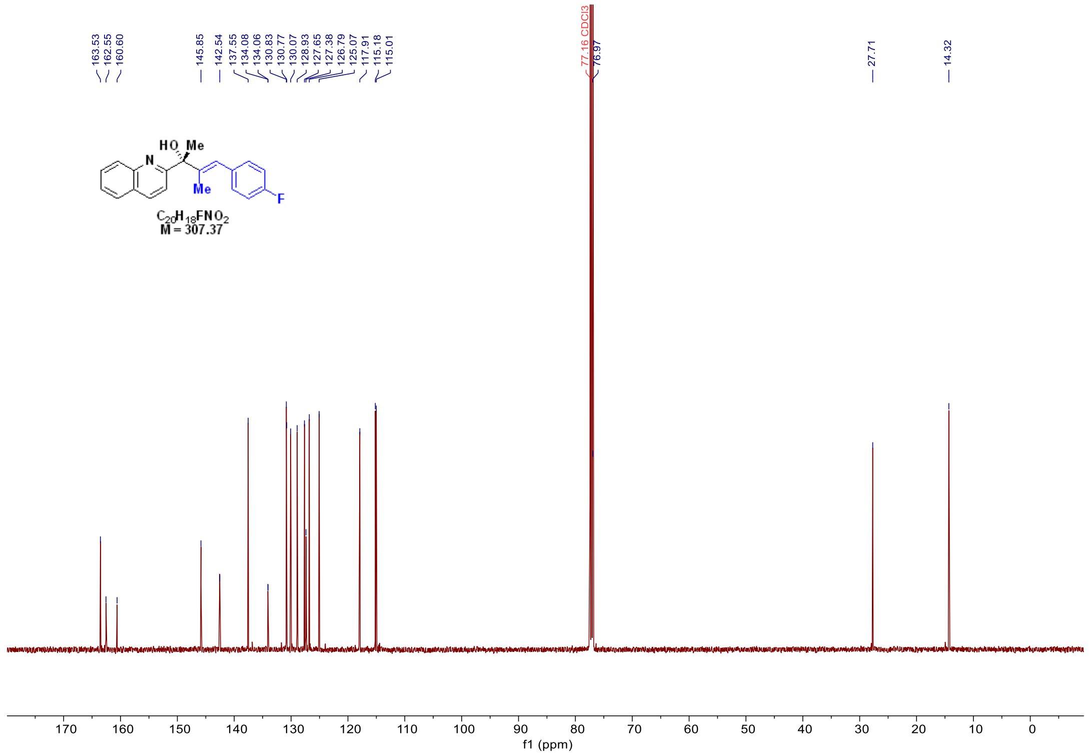

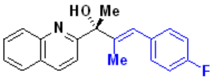

$C_{20}H_{18}FNO_2$
M = 307.37

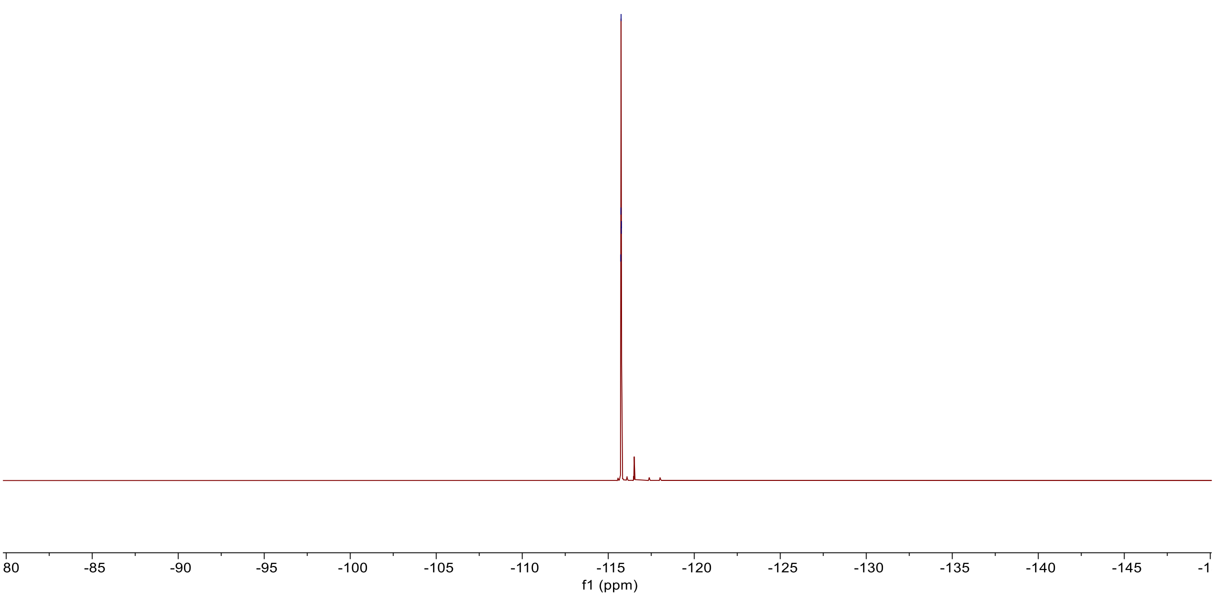

^{1}H NMR (500 MHz, CD_3Cl, 298 K) and ^{13}C NMR (125 MHz, CD_3Cl, 298 K) of **3ag**.

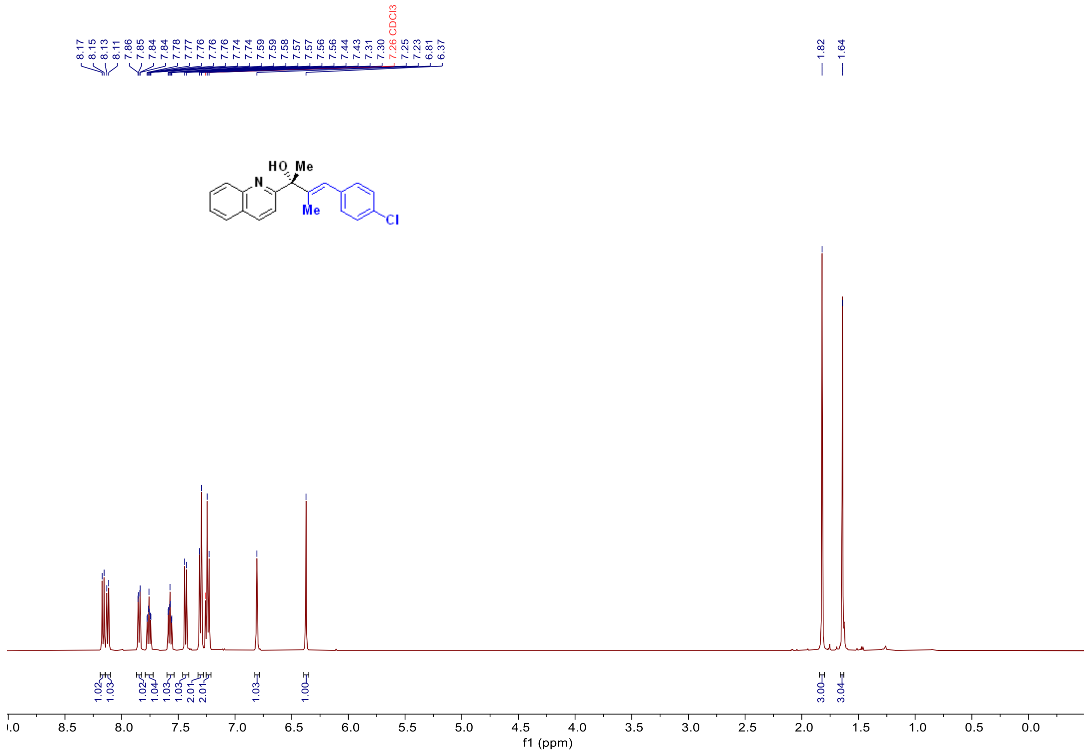

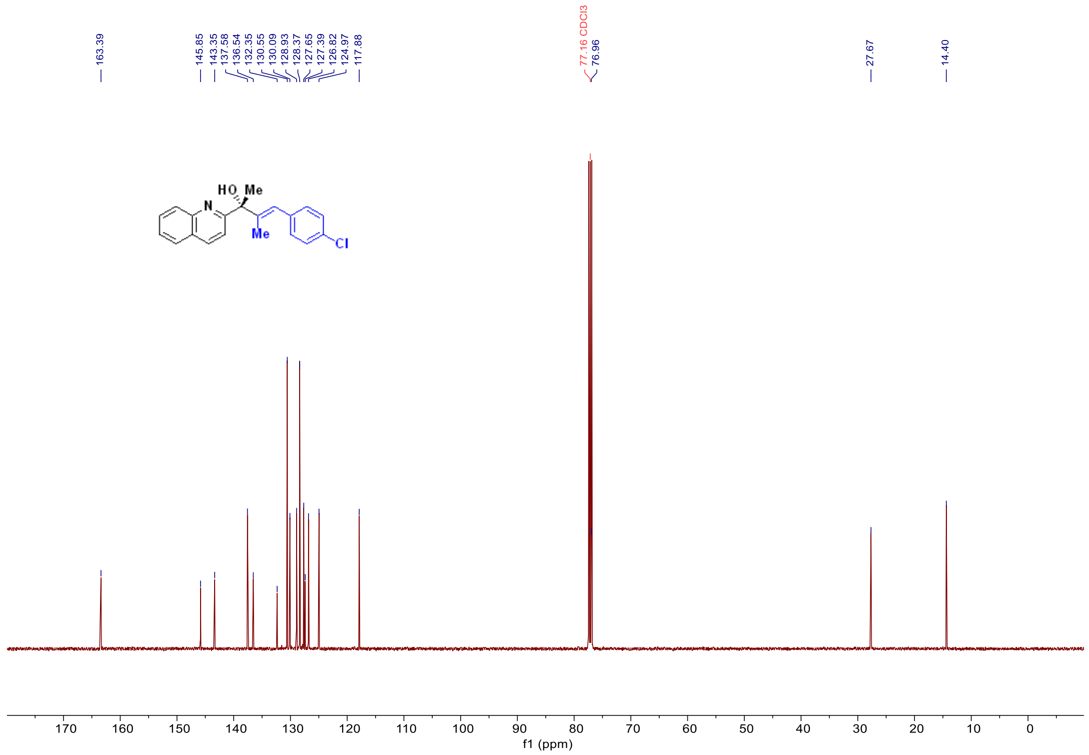

^{1}H NMR (500 MHz, CD_3Cl, 298 K) and ^{13}C NMR (125 MHz, CD_3Cl, 298 K) of **3ah**.

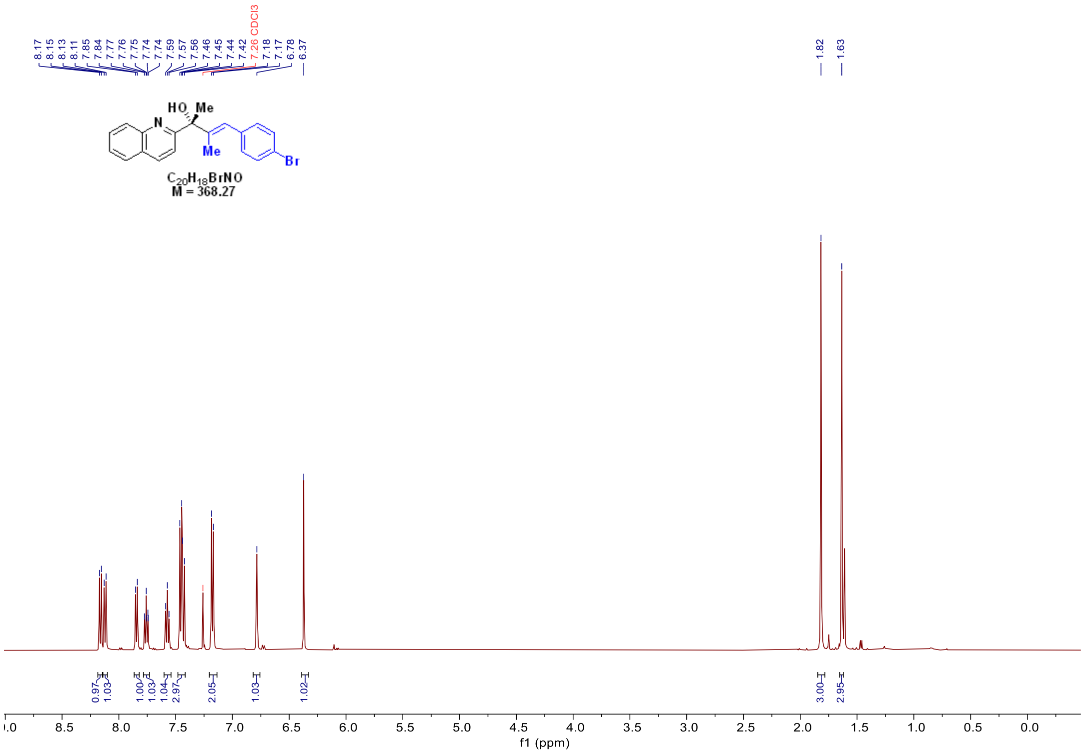

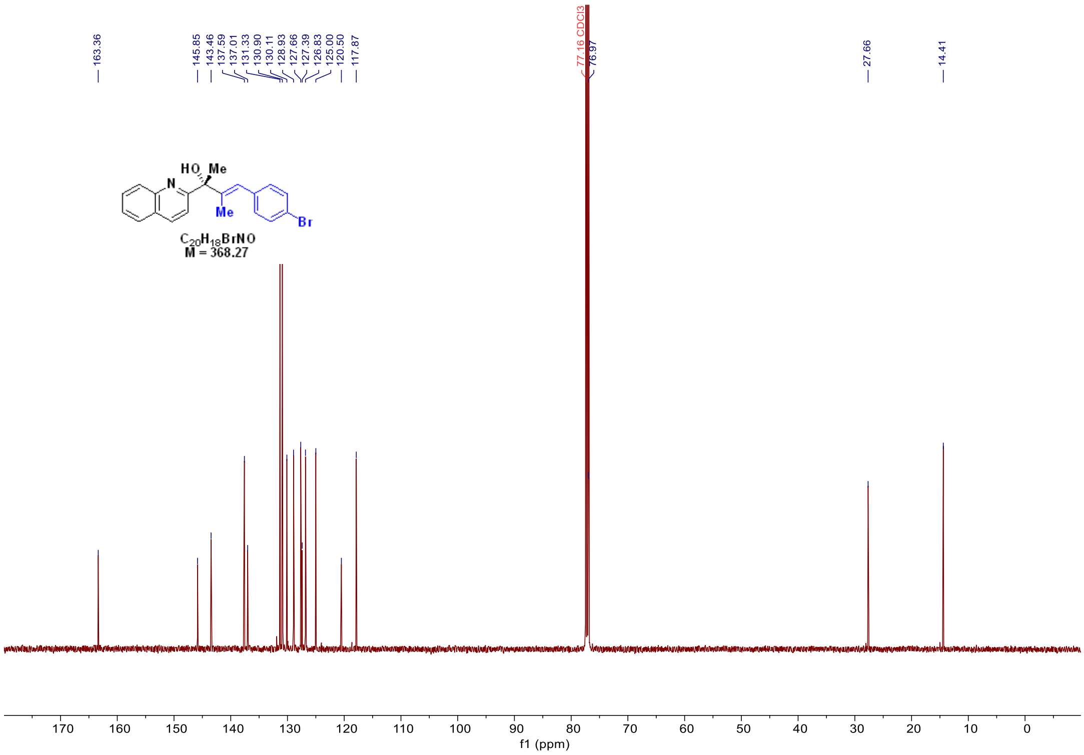

^{1}H NMR (500 MHz, CD_3Cl, 298 K), ^{13}C NMR (125 MHz, CD_3Cl, 298 K) and ^{19}F NMR (471 MHz, CD_3Cl, 298 K) of **3ai**.

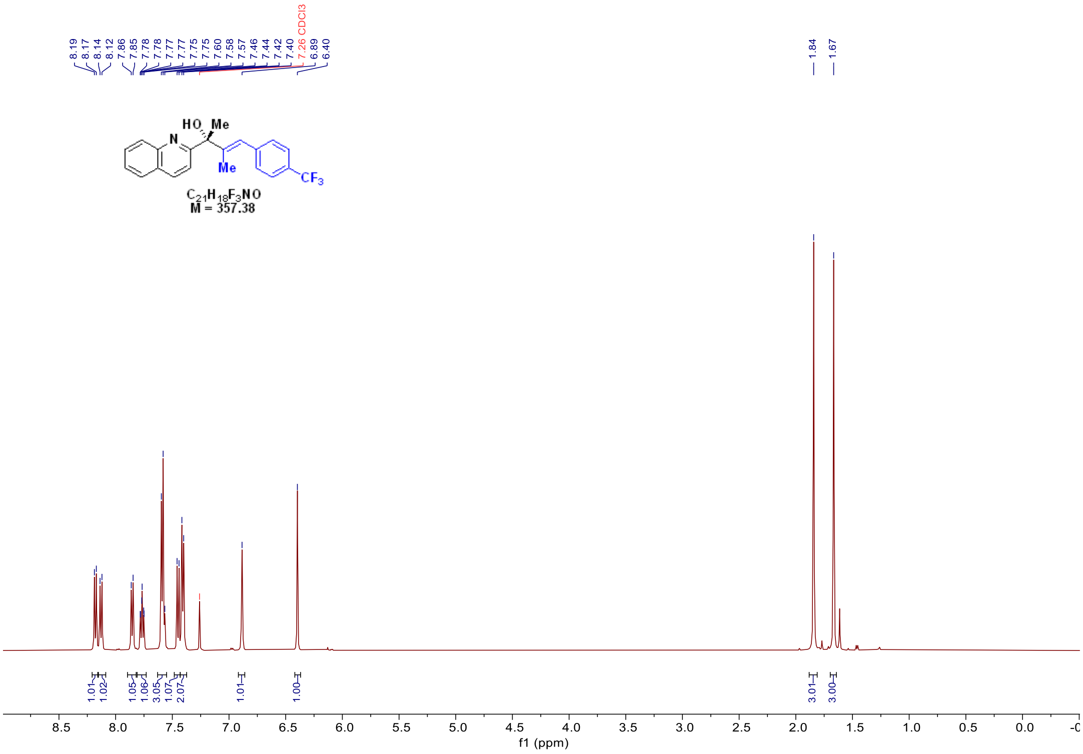

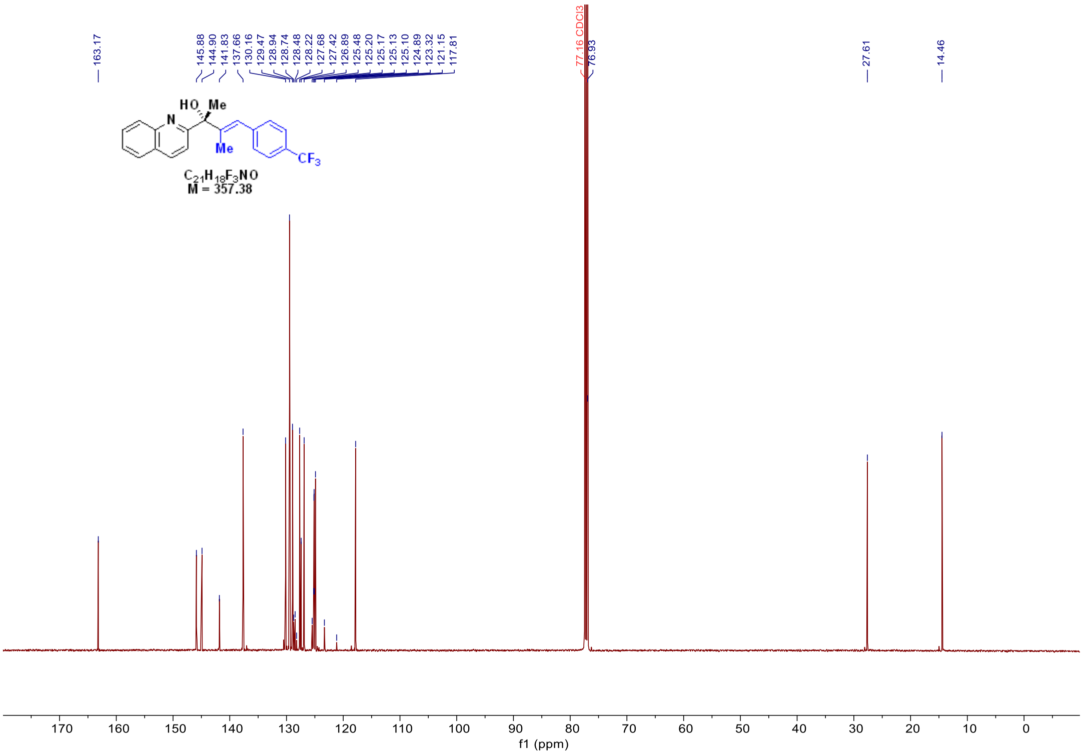

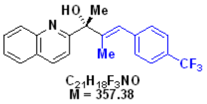
HO Me
N
Me
CF3
C21H18F3NO
M = 357.38

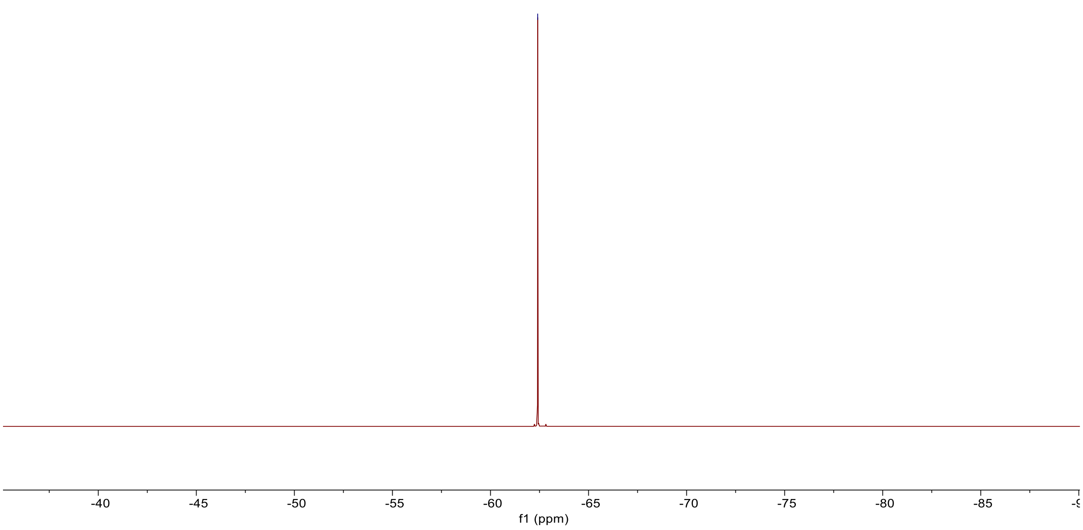
-62.41
-40
-45
-50
-55
-60
-65
-70
-75
-80
-85
f1 (ppm)

^{1}H NMR (400 MHz, CD_3Cl, 298 K) and ^{13}C NMR (100 MHz, CD_3Cl, 298 K) of **3aj**.

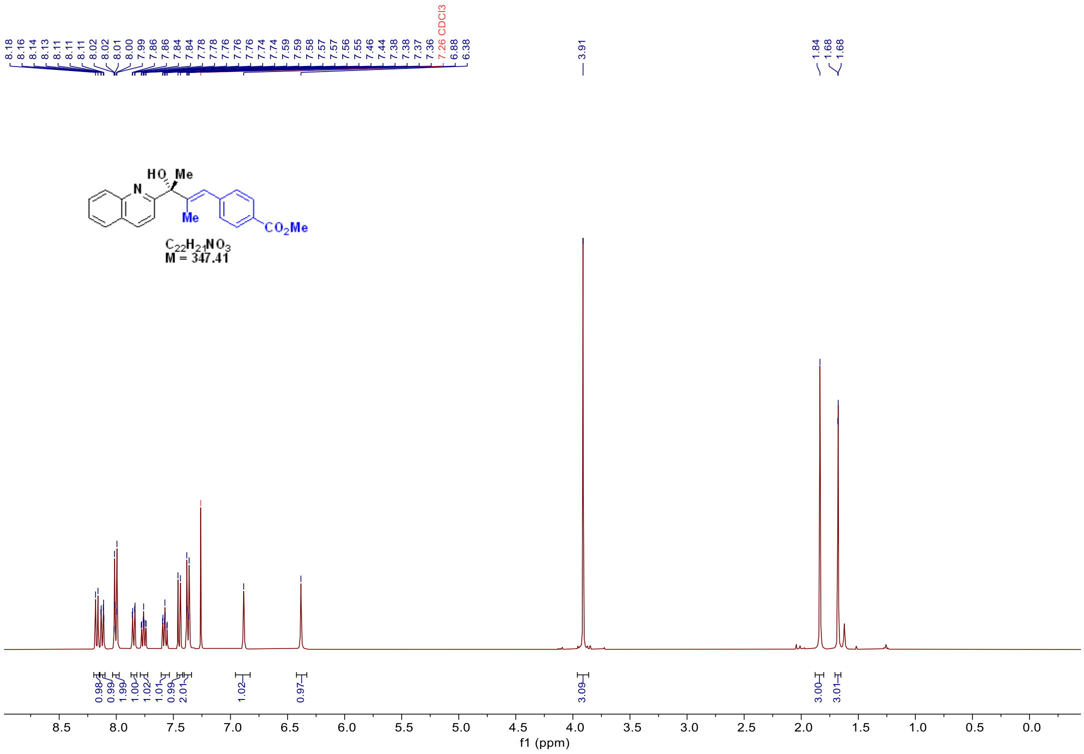

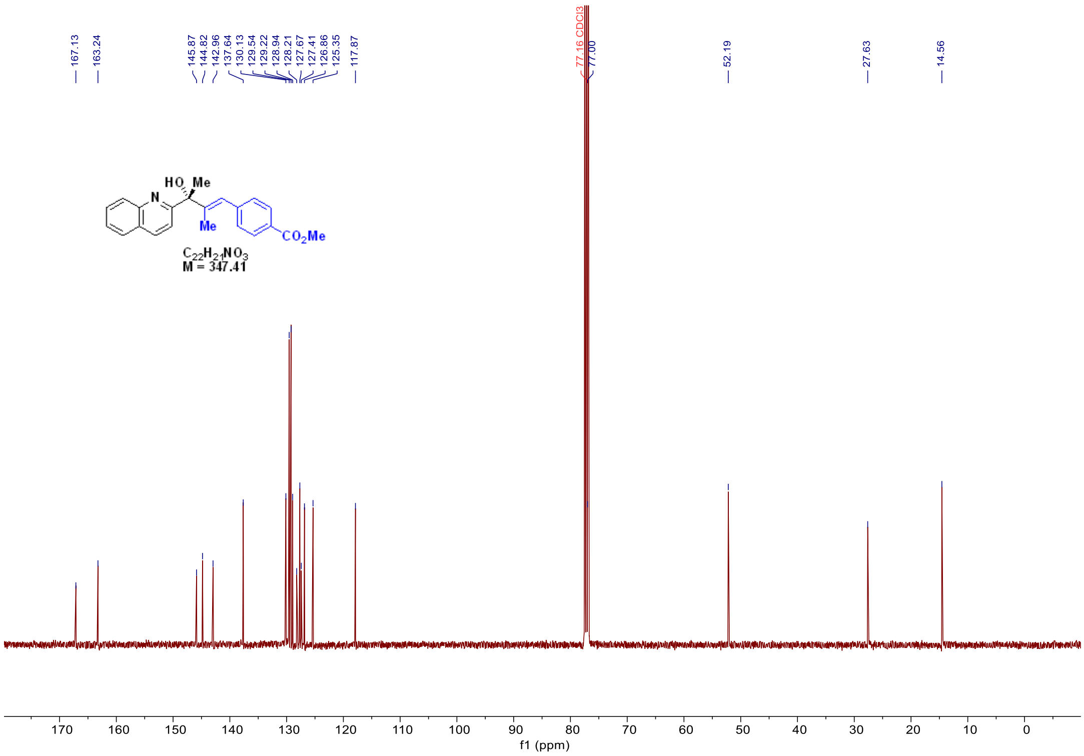

^{1}H NMR (400 MHz, CD_3Cl, 298 K) and ^{13}C NMR (100 MHz, CD_3Cl, 298 K) of **3ak**.

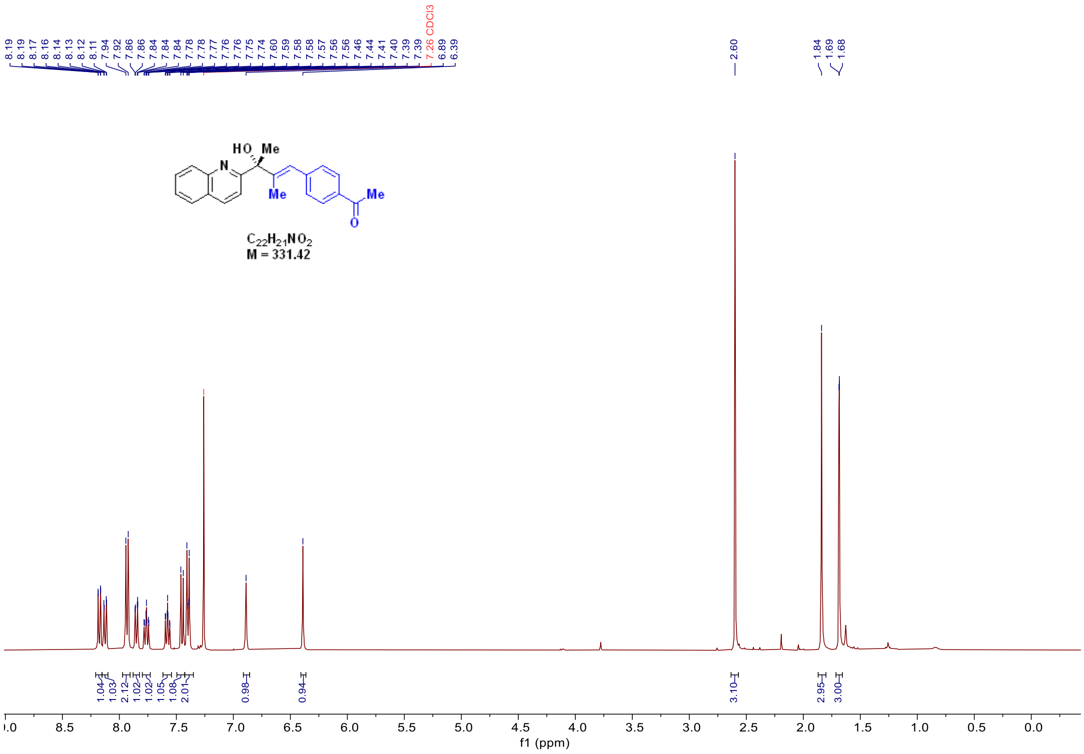

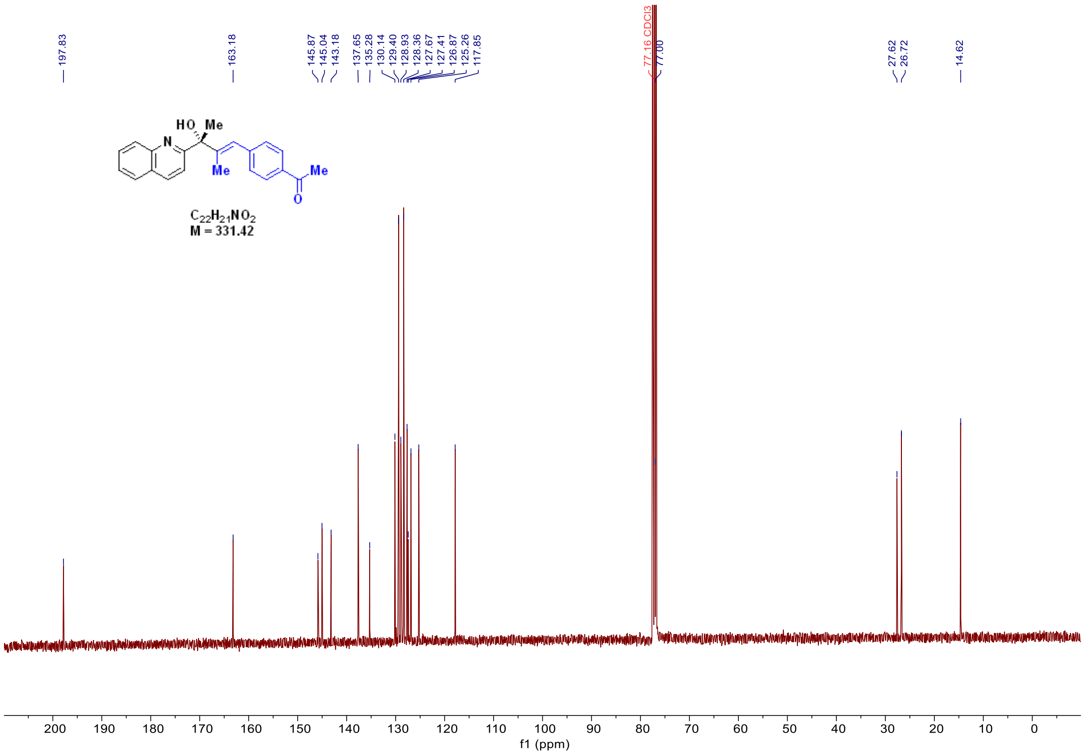

^{1}H NMR (400 MHz, CD_3Cl, 298 K) and ^{13}C NMR (100 MHz, CD_3Cl, 298 K) of **3al**.

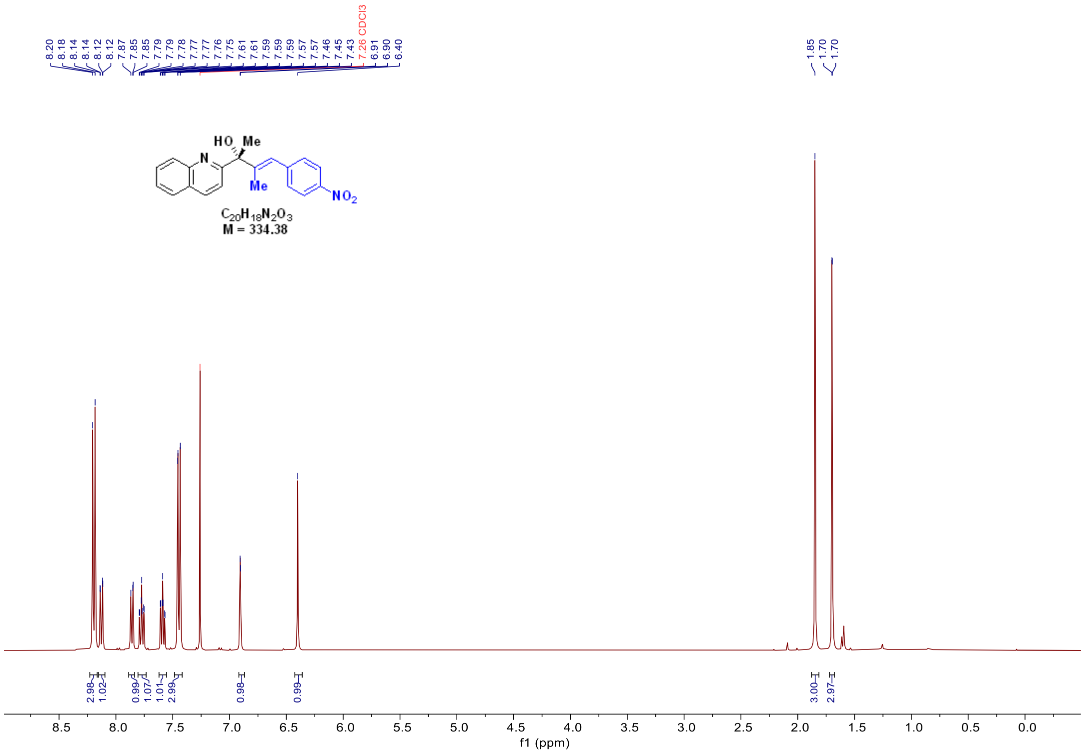

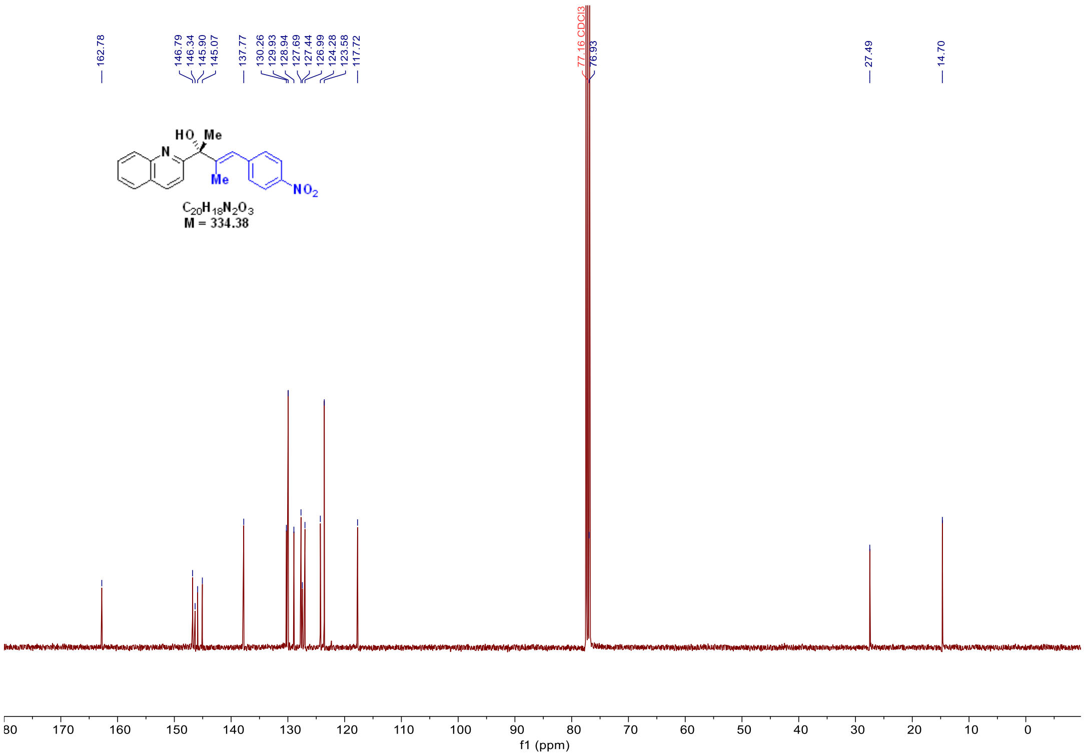

^{1}H NMR (500 MHz, CD_3Cl, 298 K) and ^{13}C NMR (125 MHz, CD_3Cl, 298 K) of **3am**.

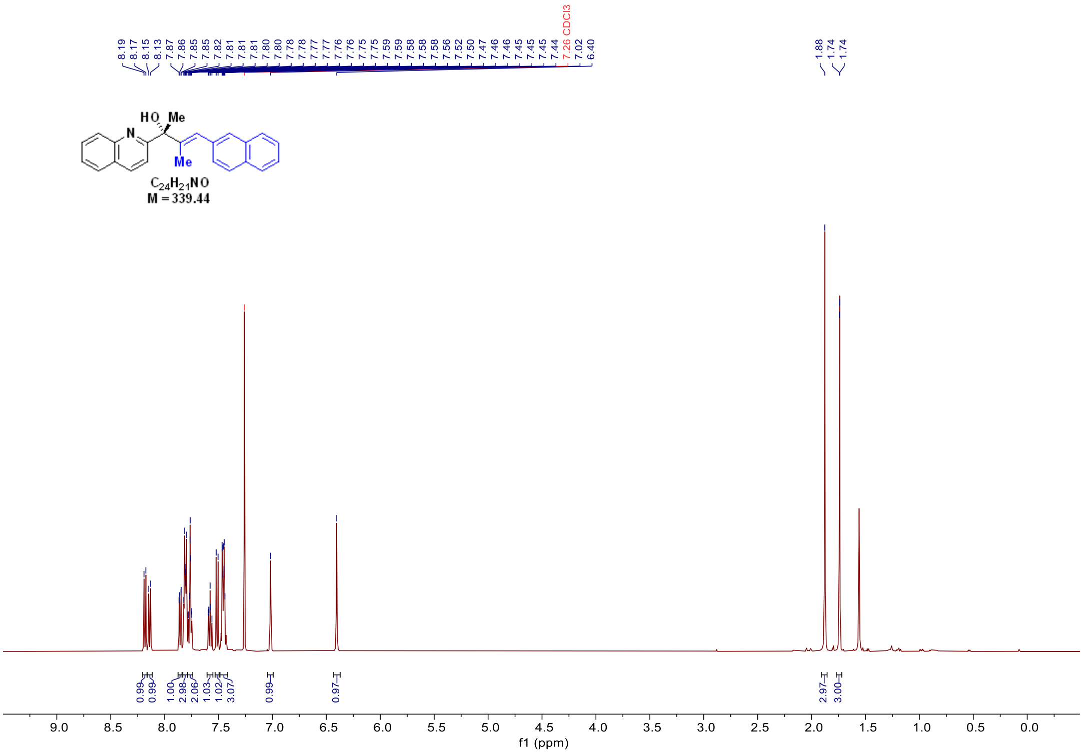

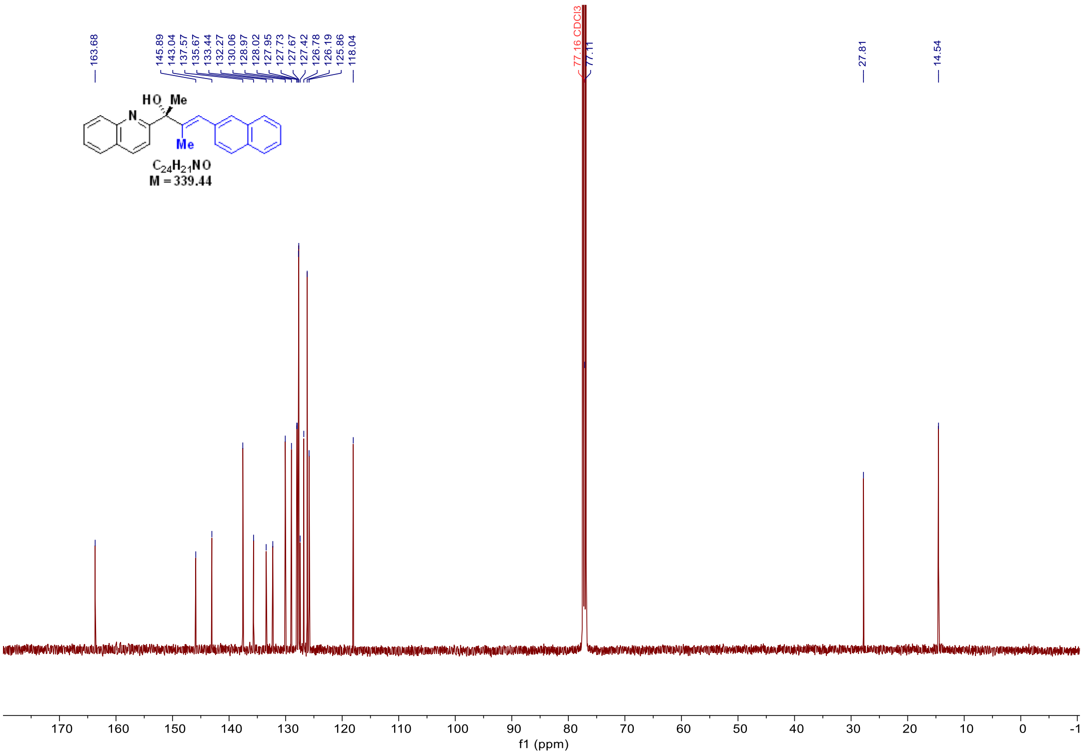

^{1}H NMR (500 MHz, CD_3Cl, 298 K), ^{13}C NMR (125 MHz, CD_3Cl, 298 K) and HMBC of **3an**.

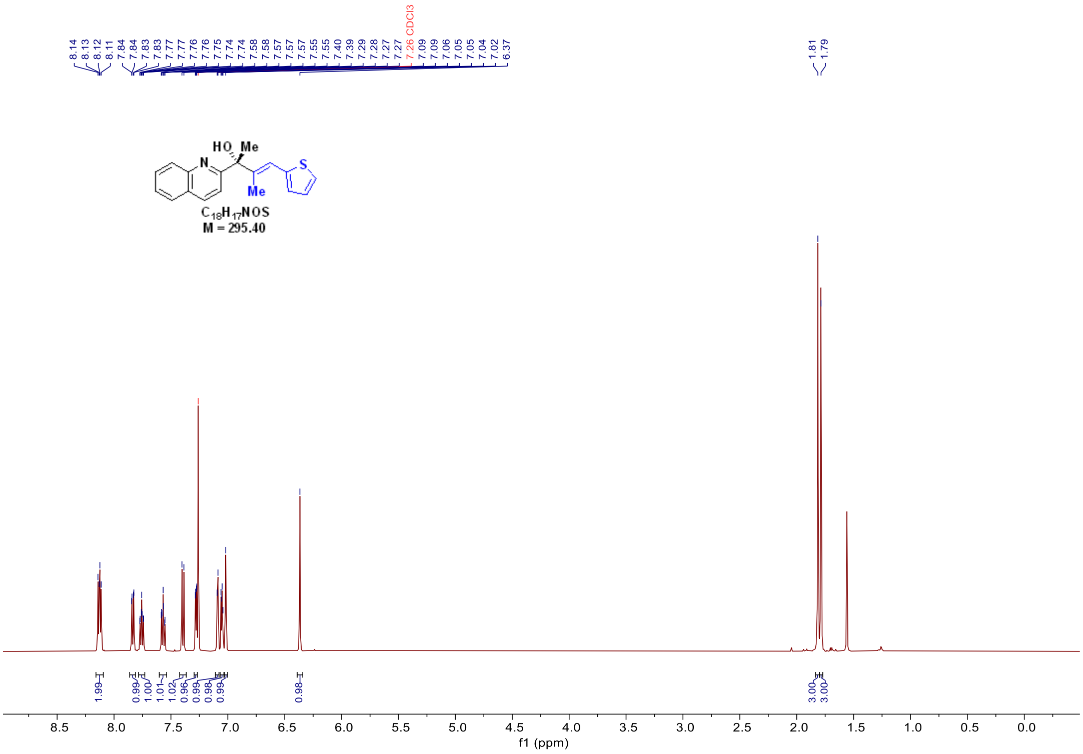

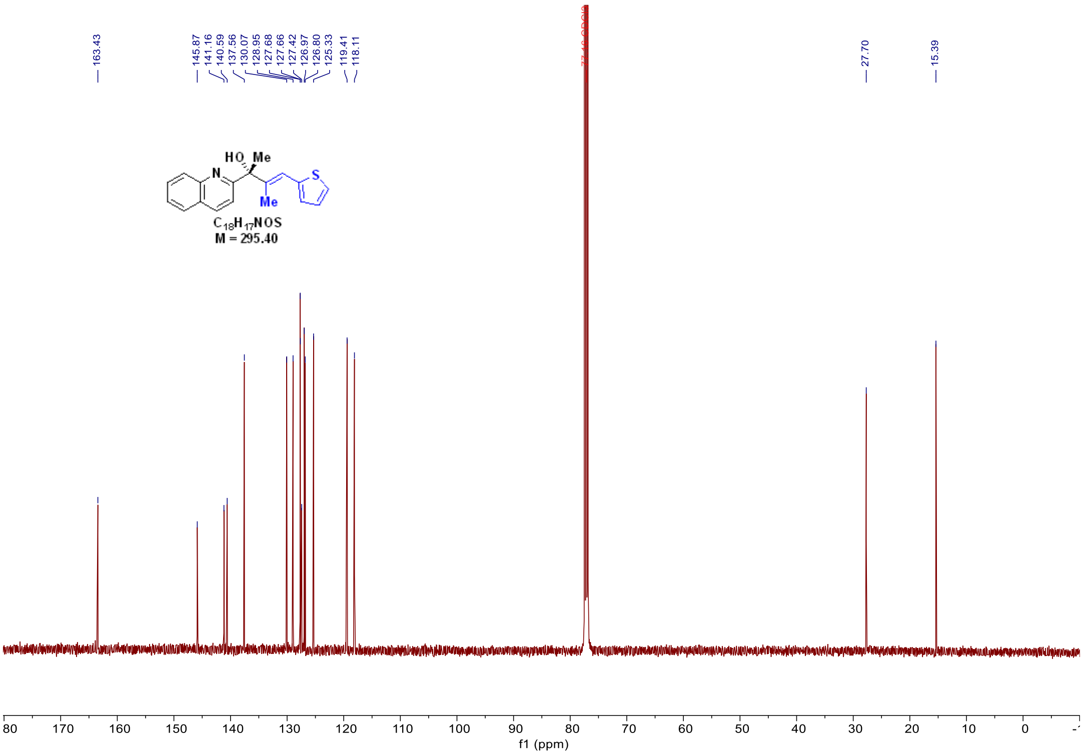

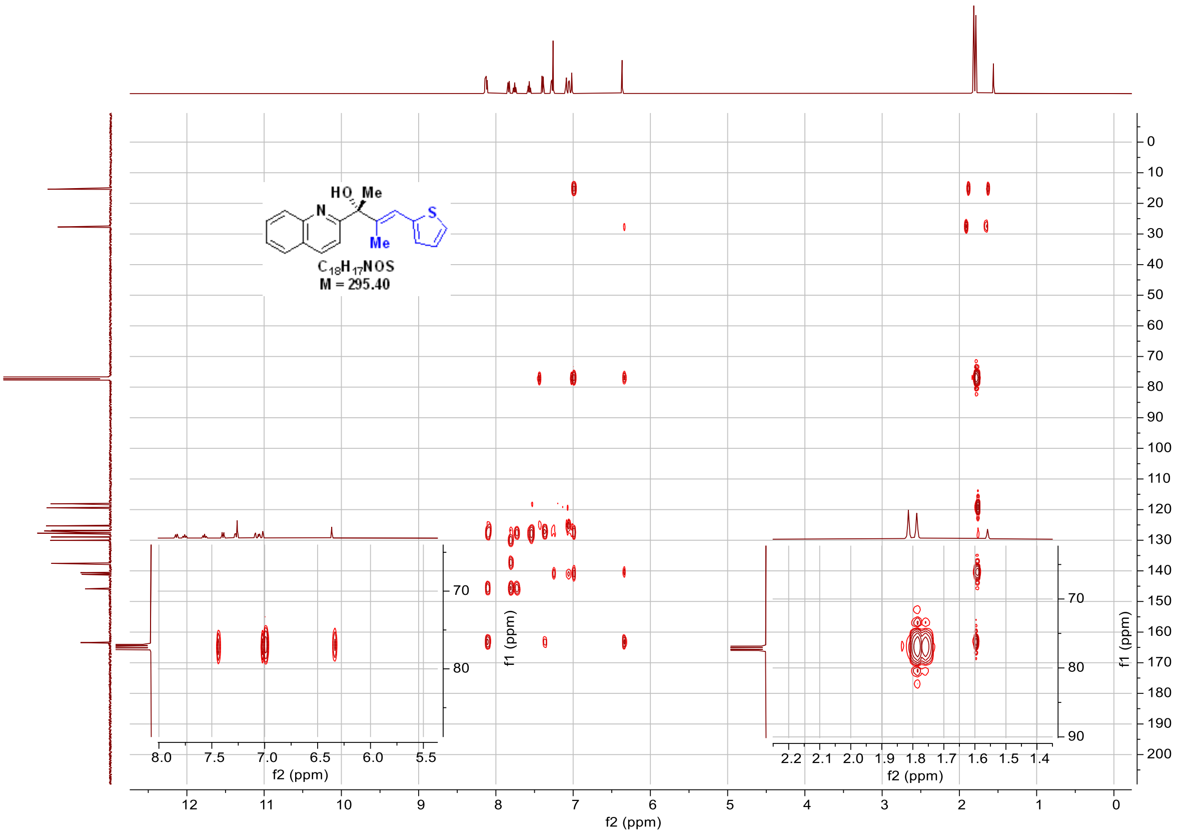

HO Me
N
S
Me
C18H17NOS
M = 295.40
f1 (ppm)
f2 (ppm)

^{1}H NMR (500 MHz, CD_3Cl, 298 K) and ^{13}C NMR (125 MHz, CD_3Cl, 298 K) of **3ao**.

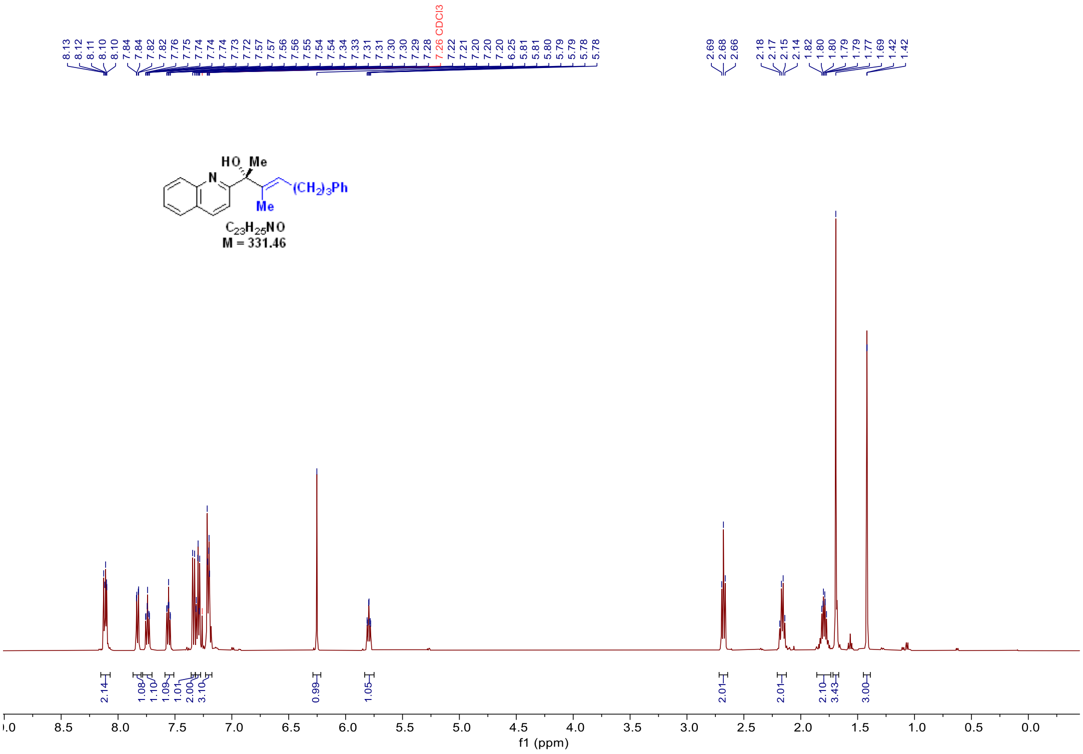

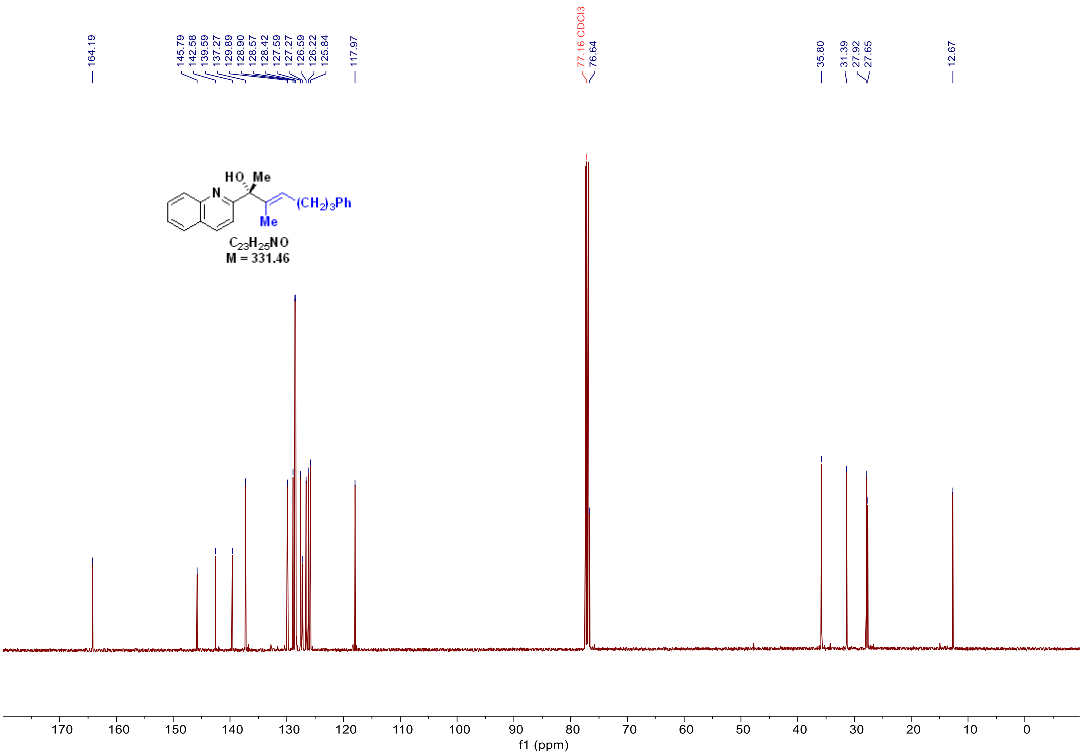

^{1}H NMR (500 MHz, CD_3Cl, 298 K) and ^{13}C NMR (125 MHz, CD_3Cl, 298 K) of **3bo**.

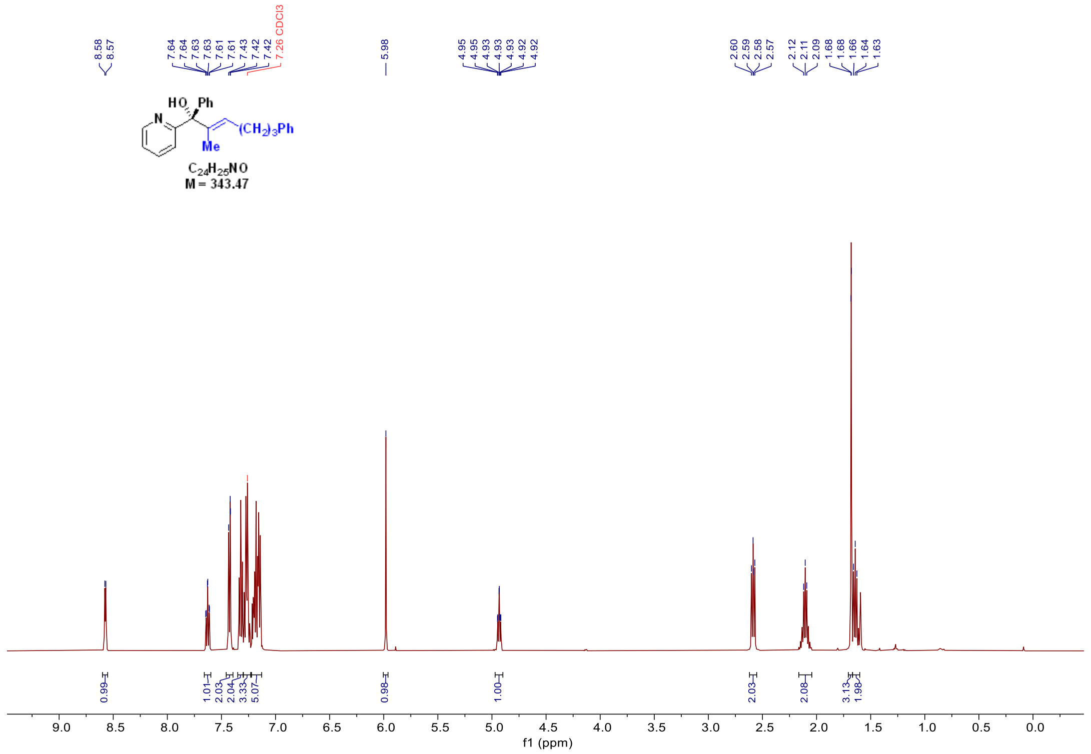

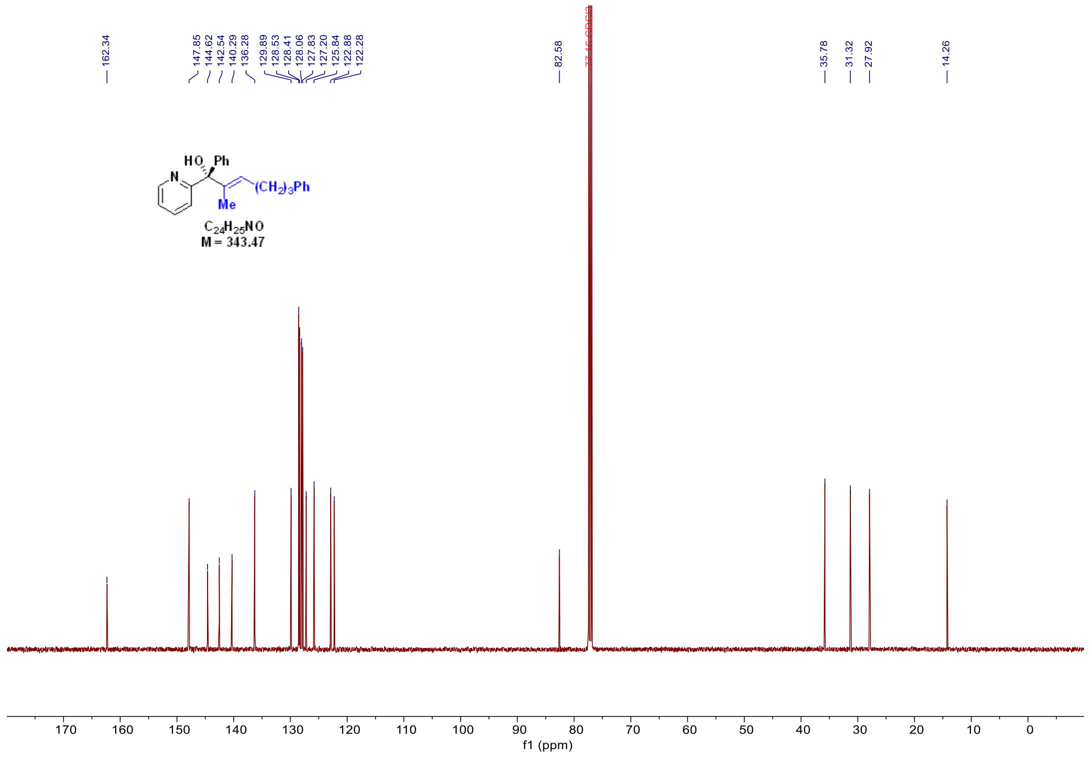

^{1}H NMR (500 MHz, CD_3Cl, 298 K) and ^{13}C NMR (125 MHz, CD_3Cl, 298 K) of **3bp**.

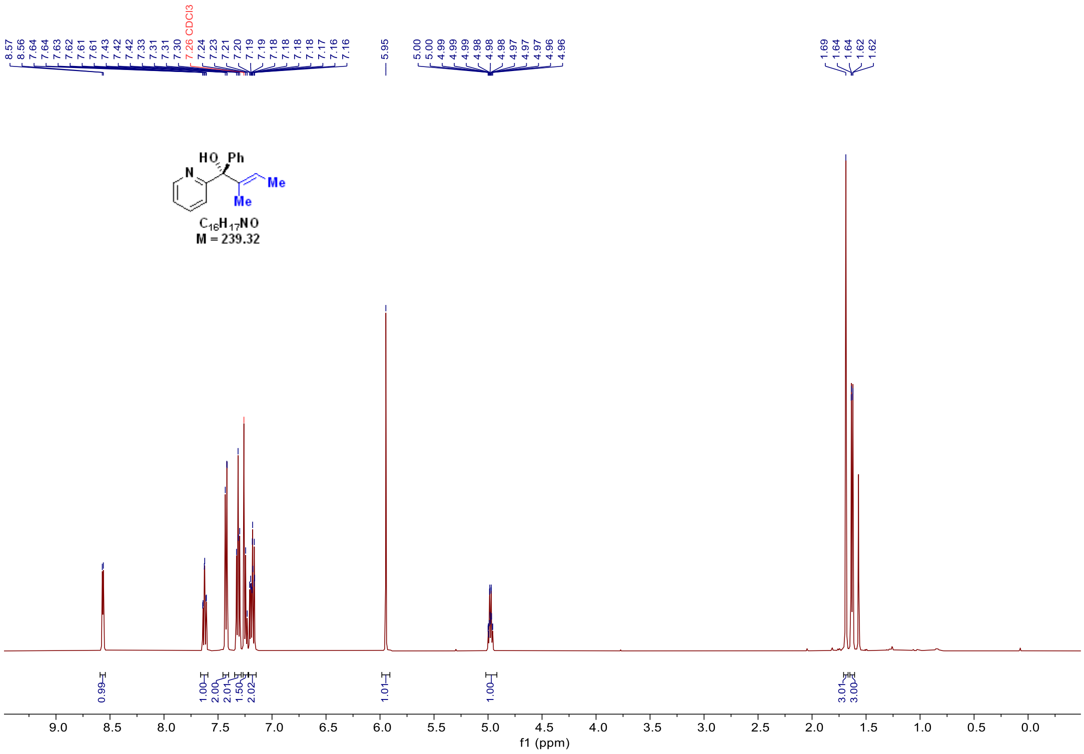

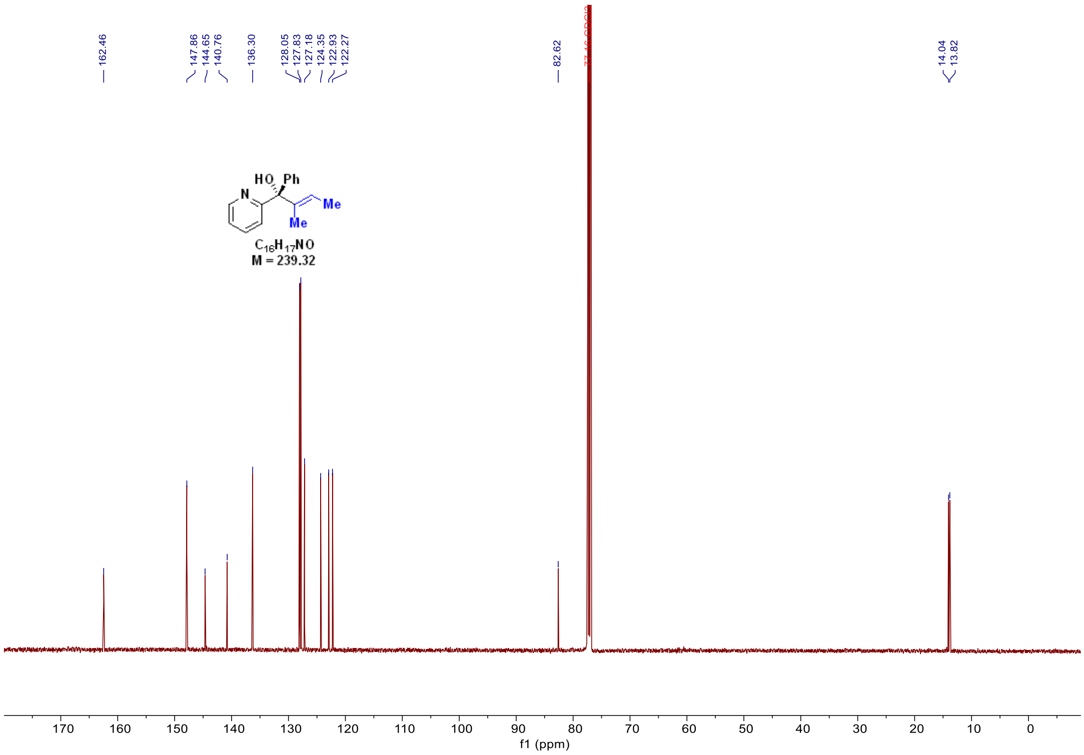

^{1}H NMR (400 MHz, CD_3Cl, 298 K) and ^{13}C NMR (100 MHz, CD_3Cl, 298 K) of **3bq**.

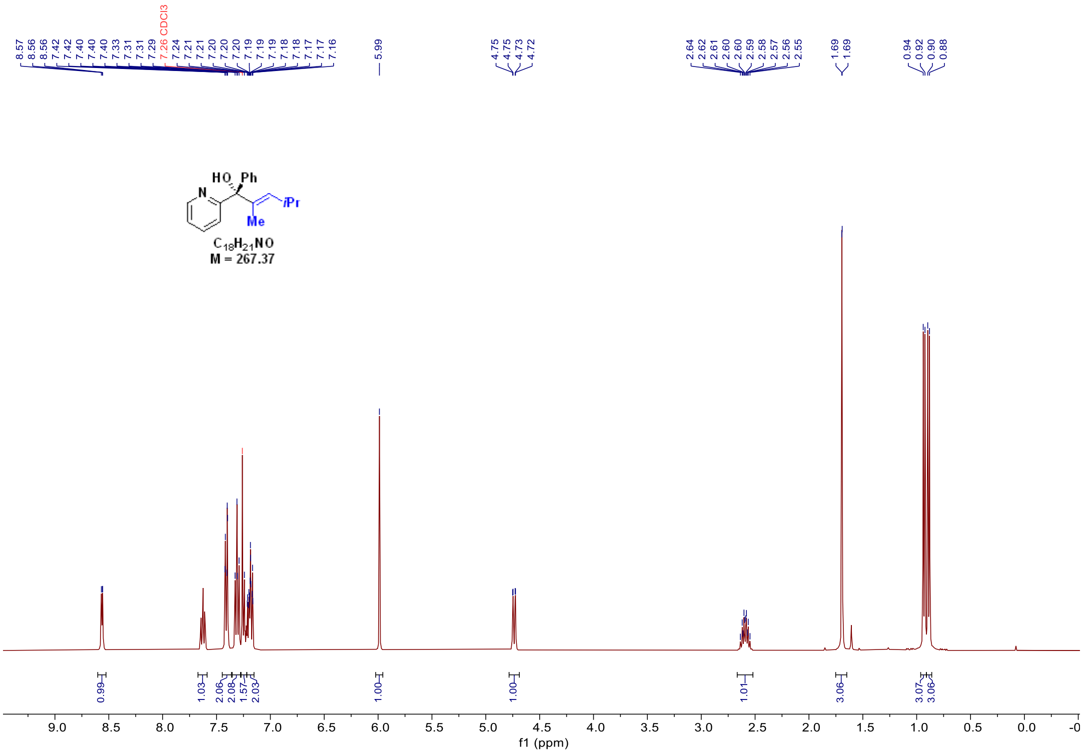

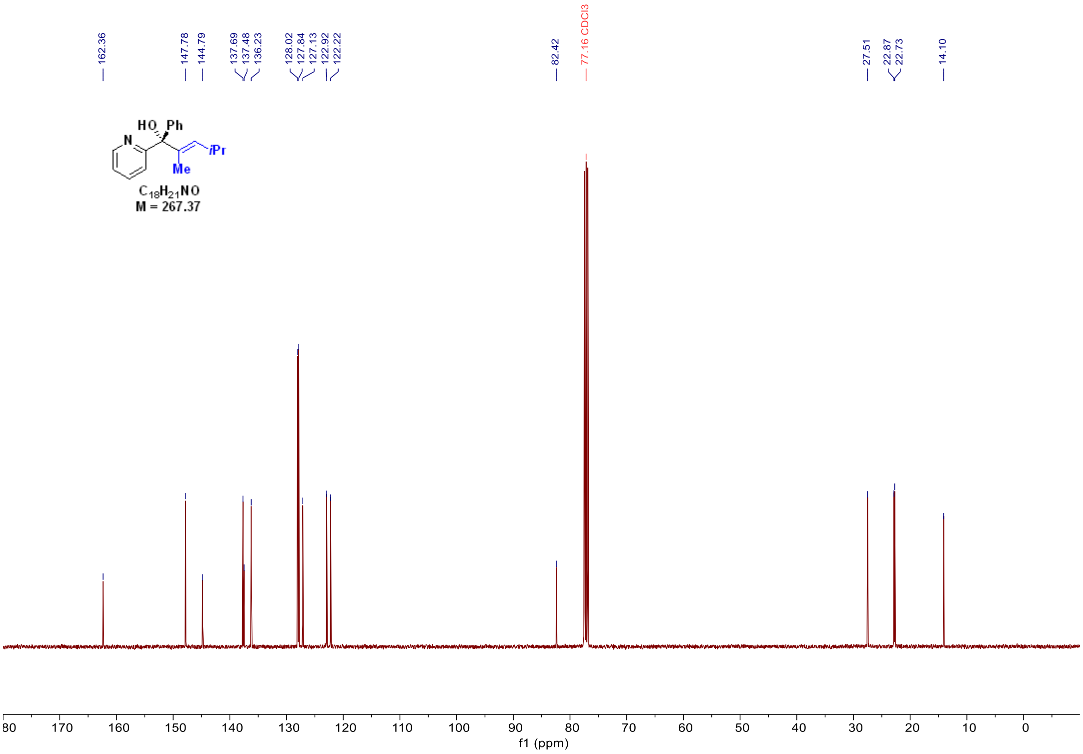

^{1}H NMR (400 MHz, CD_3Cl, 298 K) and ^{13}C NMR (100 MHz, CD_3Cl, 298 K) of **3br**.

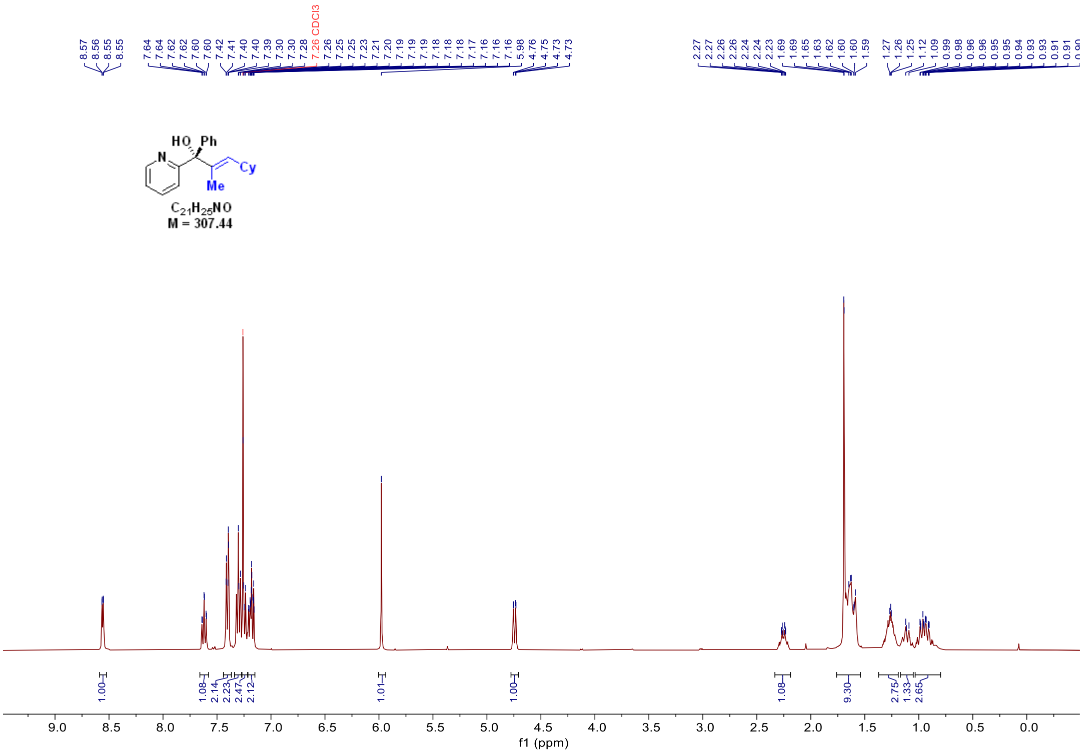

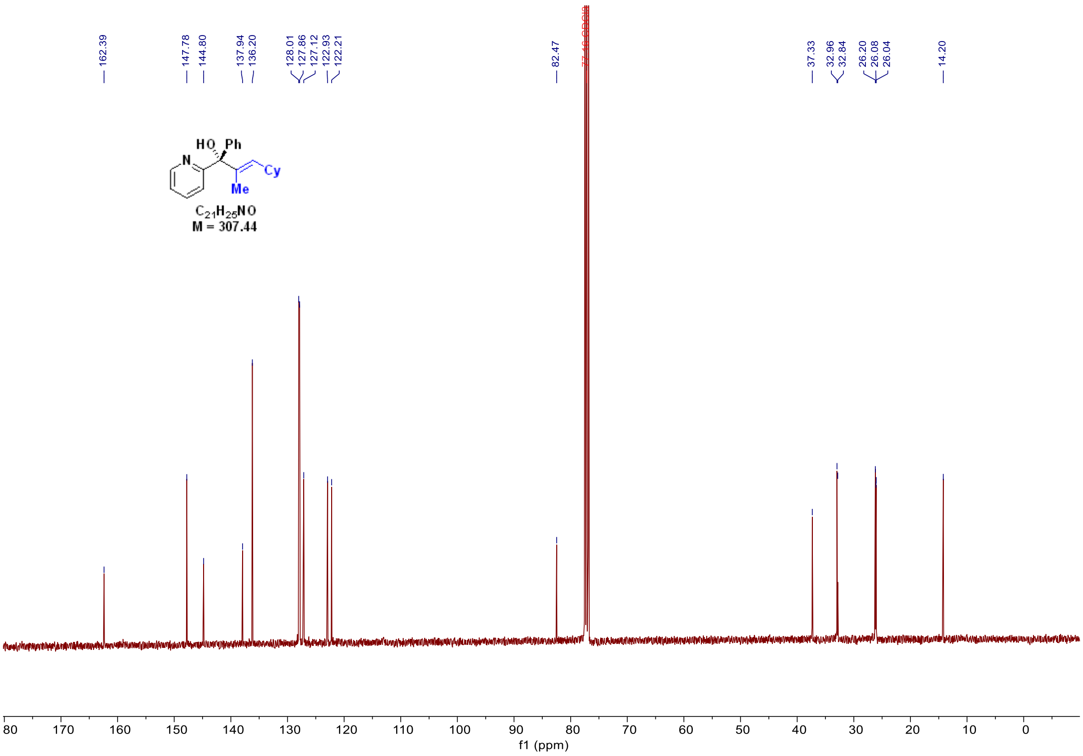

^{1}H NMR (500 MHz, CD_3Cl, 298 K) and ^{13}C NMR (125 MHz, CD_3Cl, 298 K) of **3bs**.

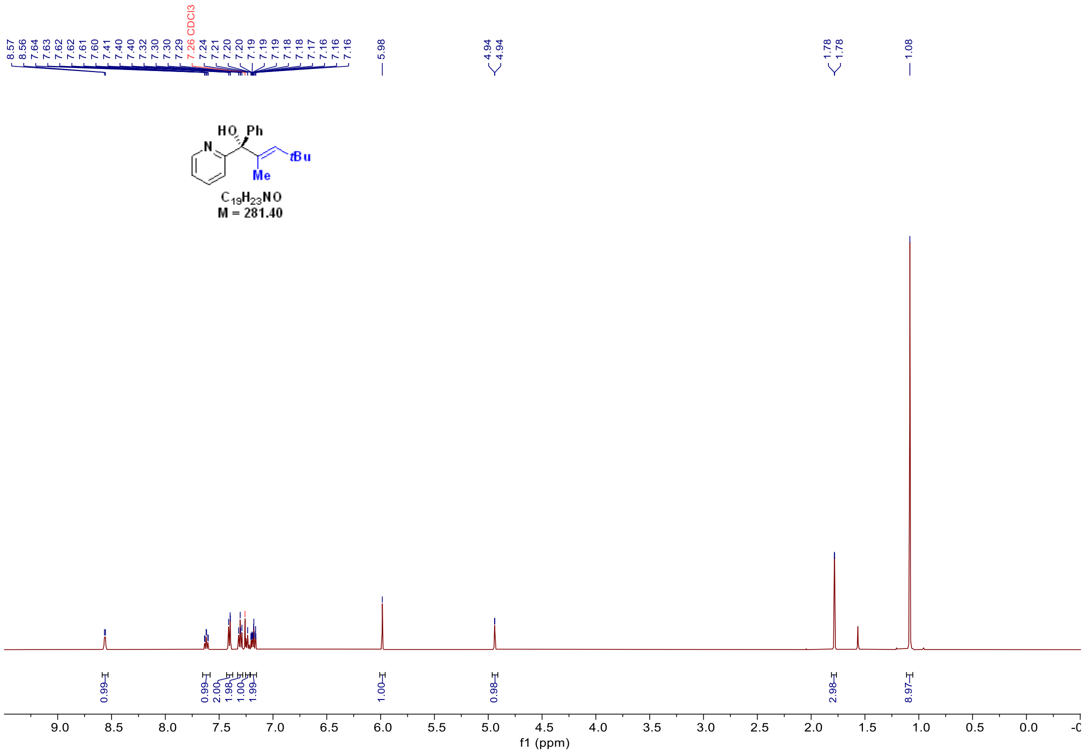

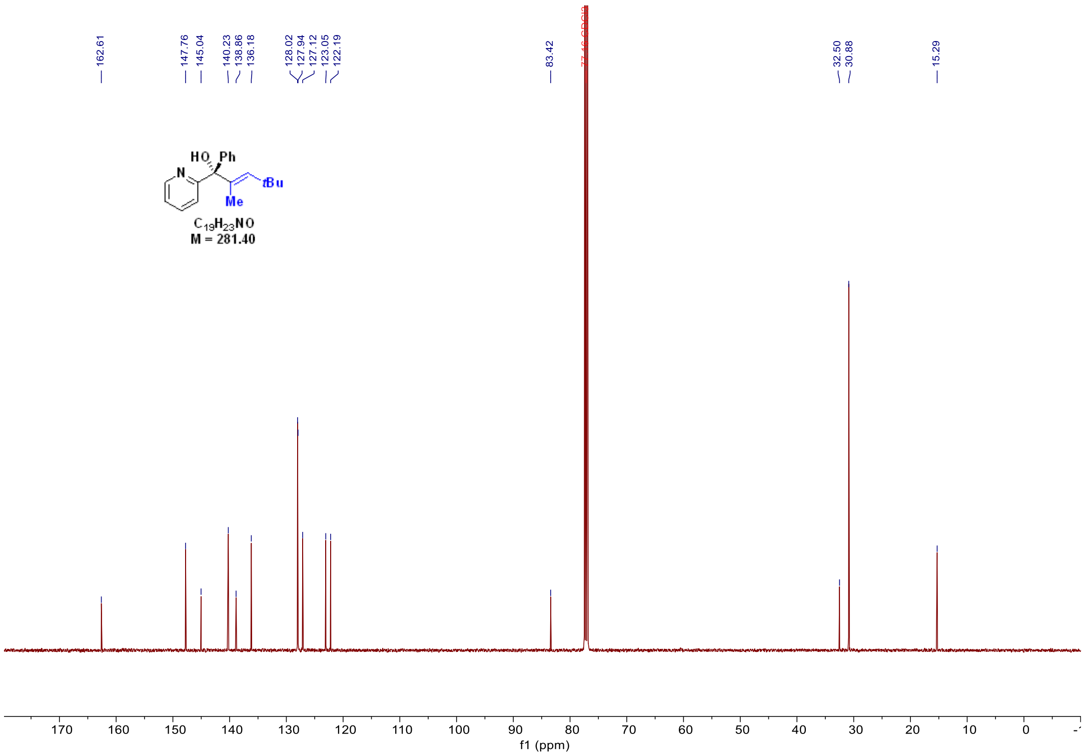

^{1}H NMR (400 MHz, CD_3Cl, 298 K) and ^{13}C NMR (100 MHz, CD_3Cl, 298 K) of **3bt**.

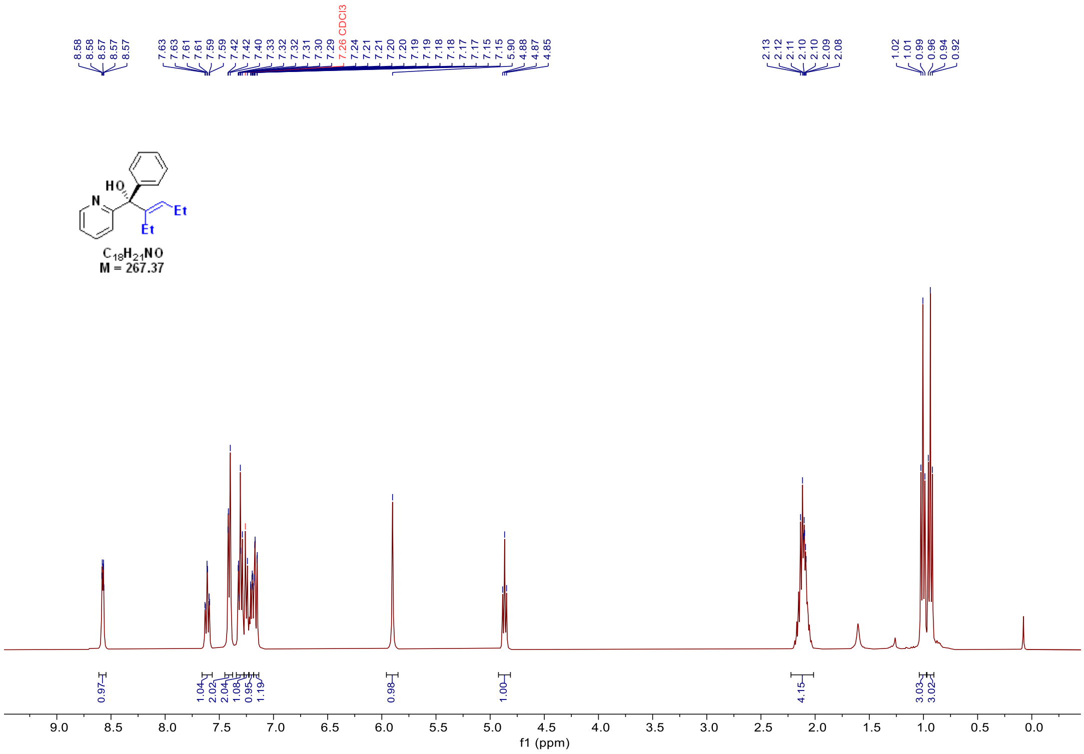

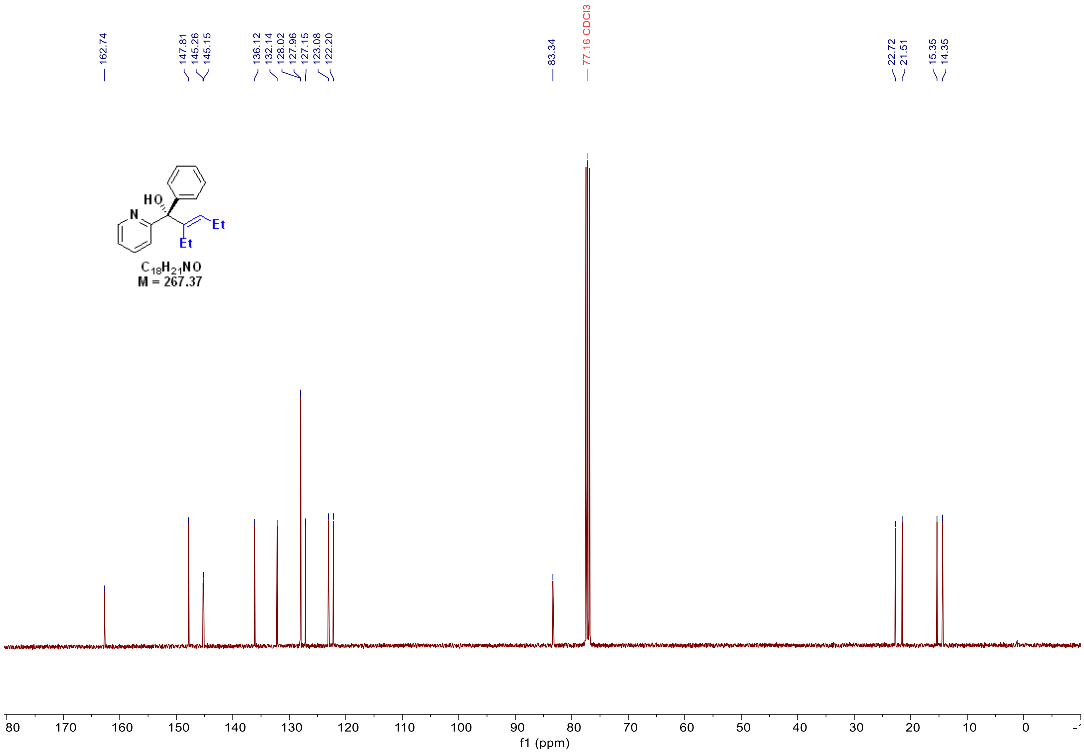

^{1}H NMR (500 MHz, CD_3Cl, 298 K) and ^{13}C NMR (125 MHz, CD_3Cl, 298 K) of **3bu**.

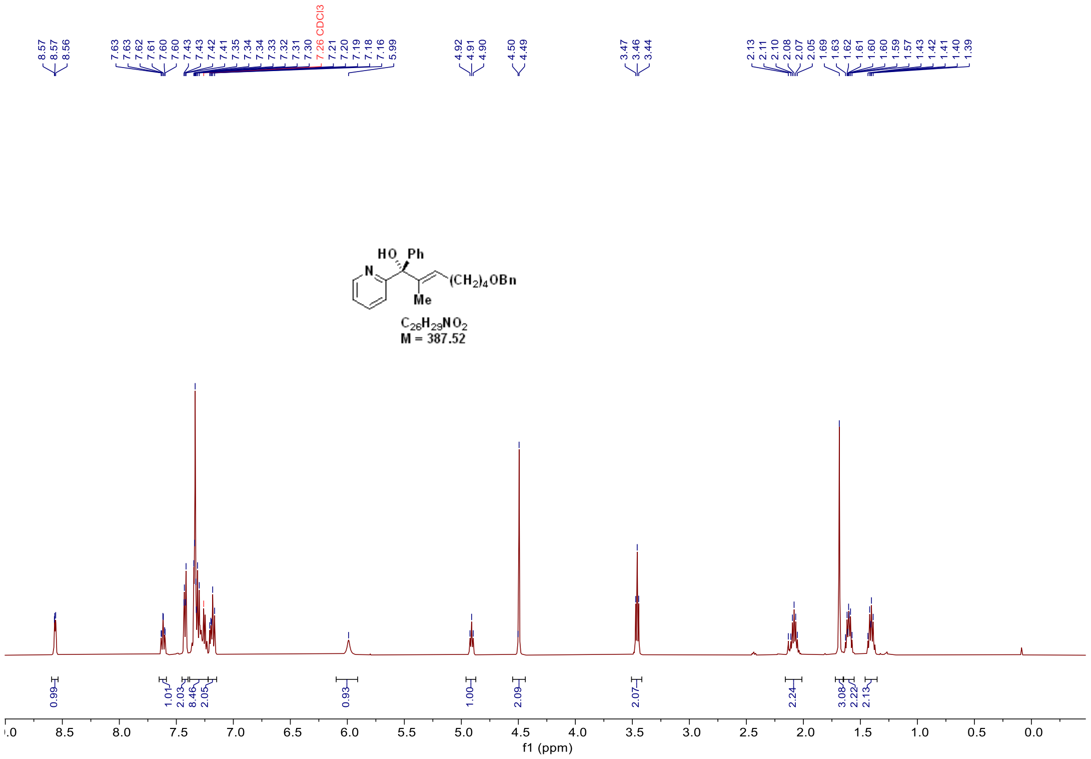

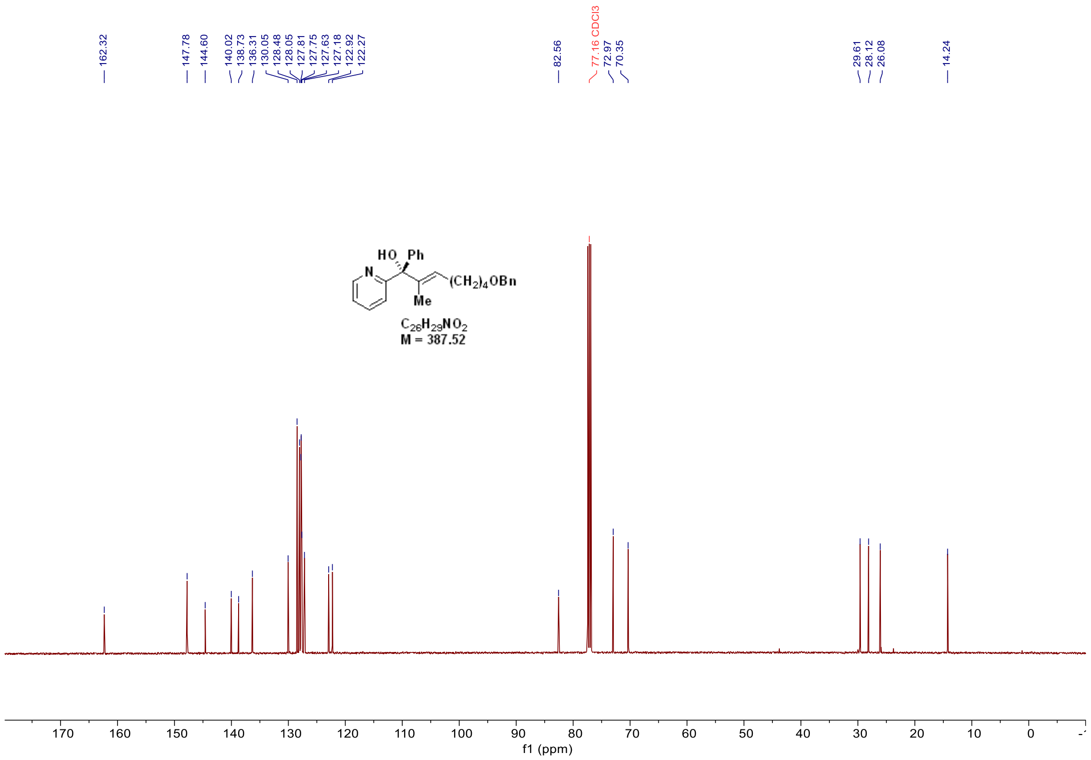

^{1}H NMR (400 MHz, CD_3Cl, 298 K) and ^{13}C NMR (100 MHz, CD_3Cl, 298 K) of **3bv**.

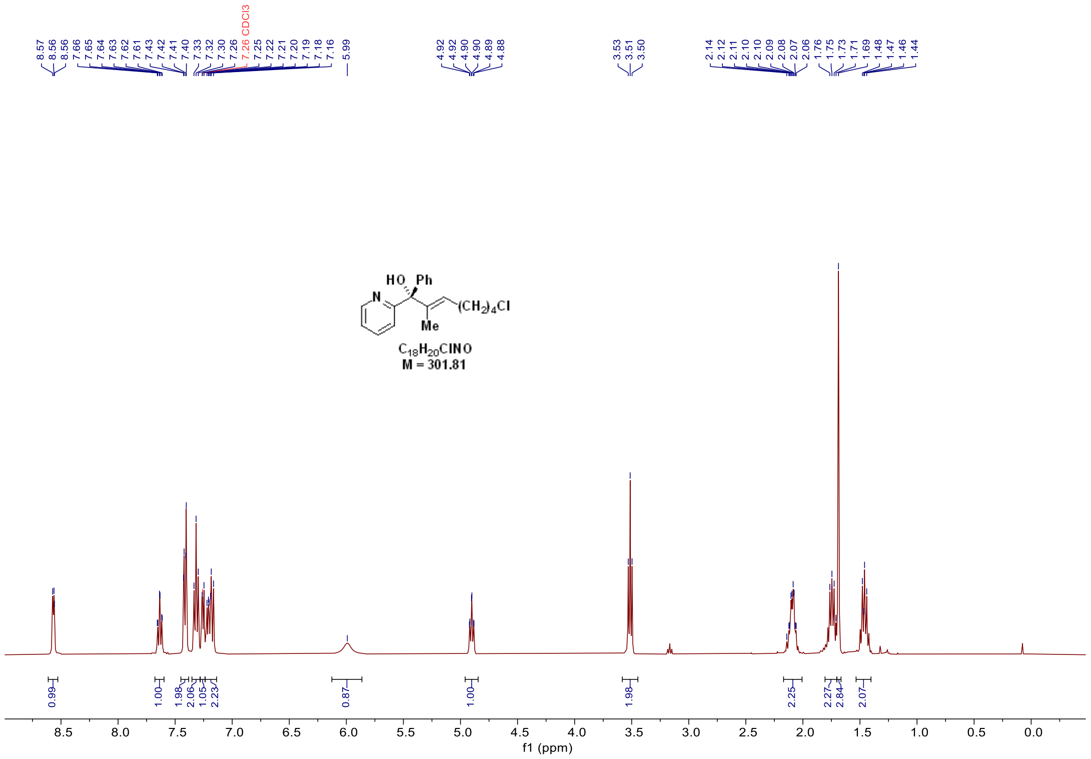

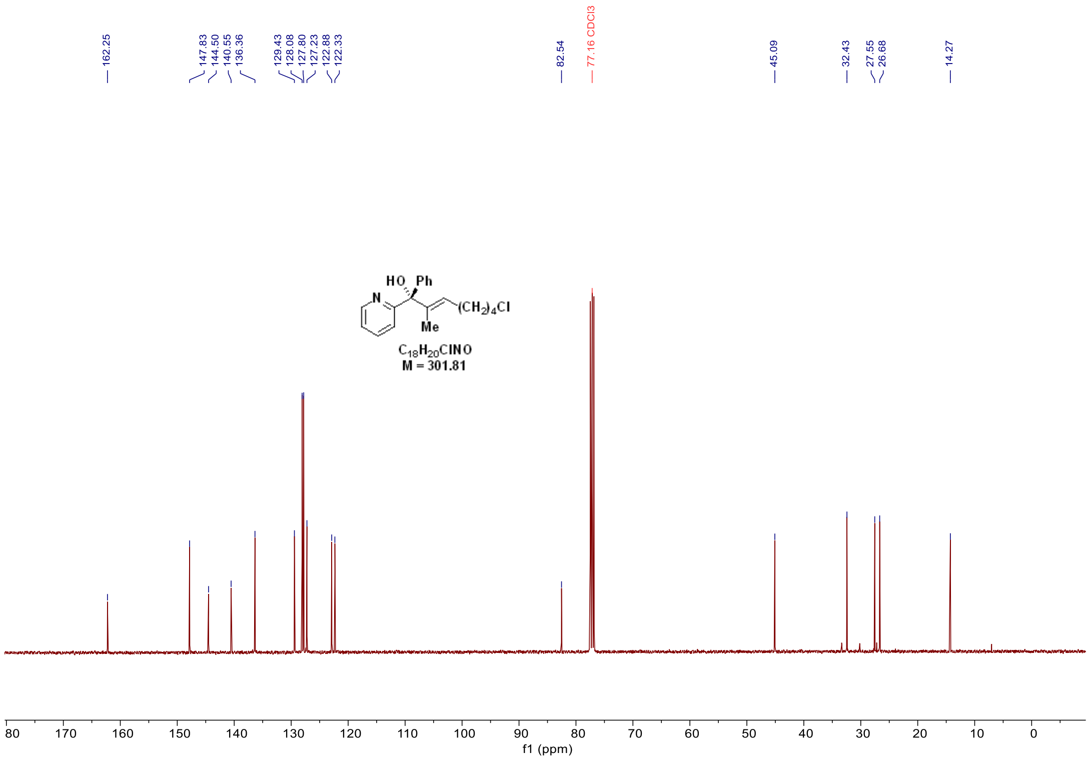

^{1}H NMR (500 MHz, CD_3Cl, 298 K) and ^{13}C NMR (125 MHz, CD_3Cl, 298 K) of **3cp**.

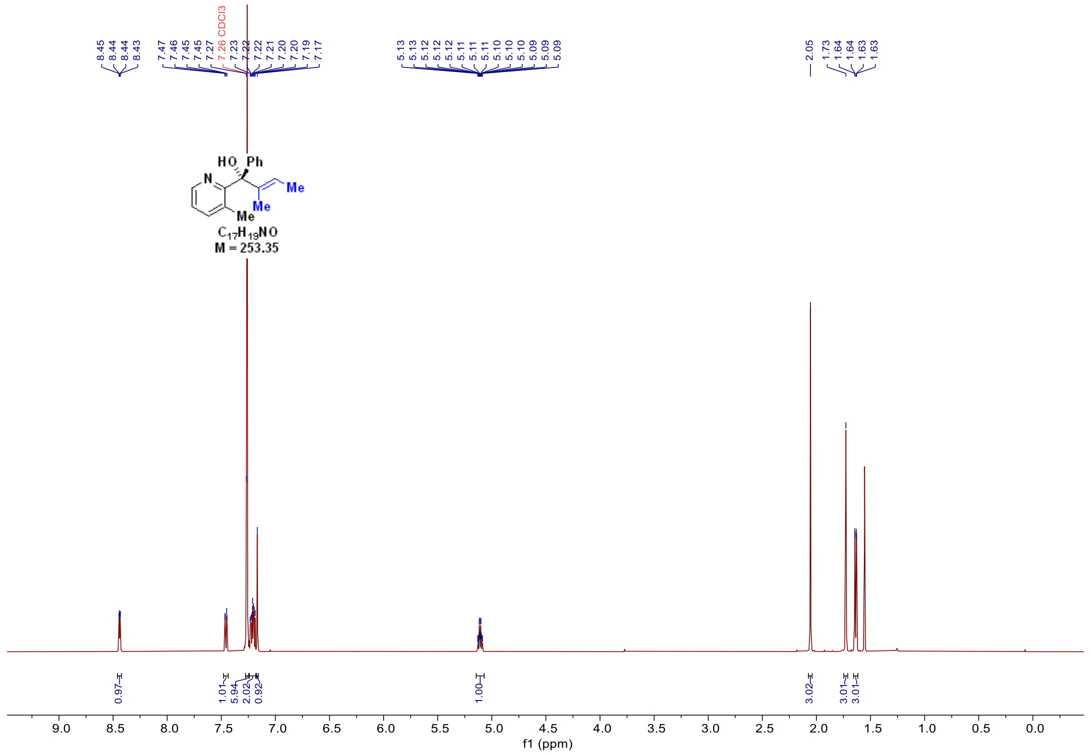

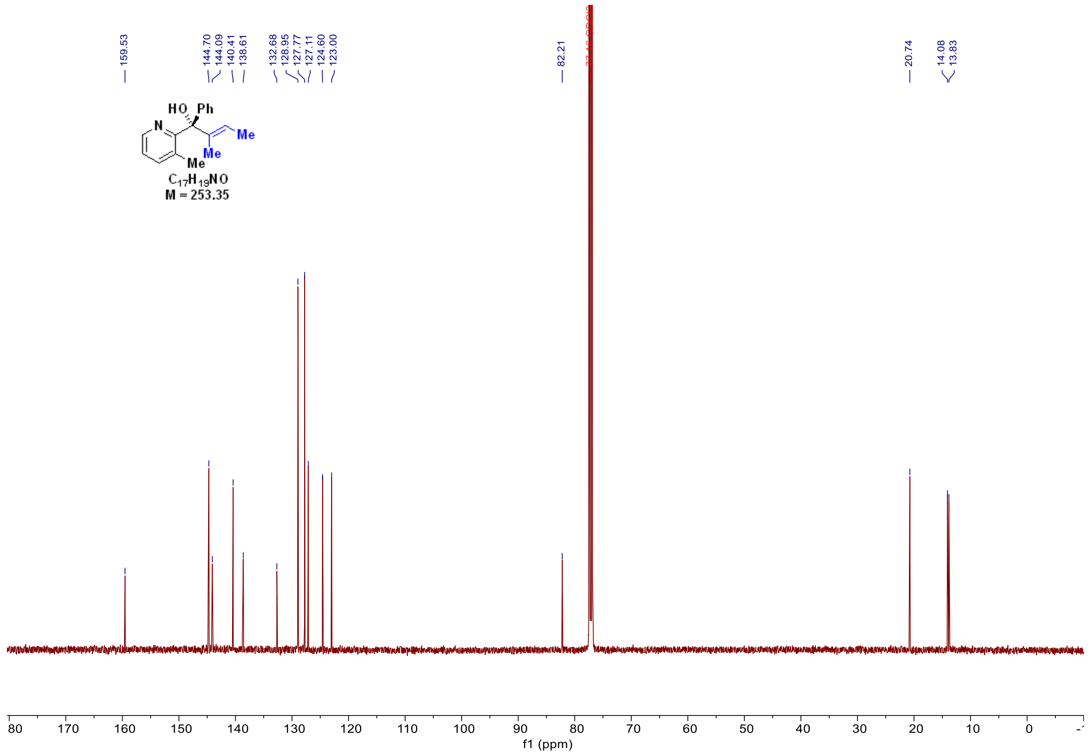

^{1}H NMR (500 MHz, CD_3Cl, 298 K) and ^{13}C NMR (125 MHz, CD_3Cl, 298 K) of **3dp**.

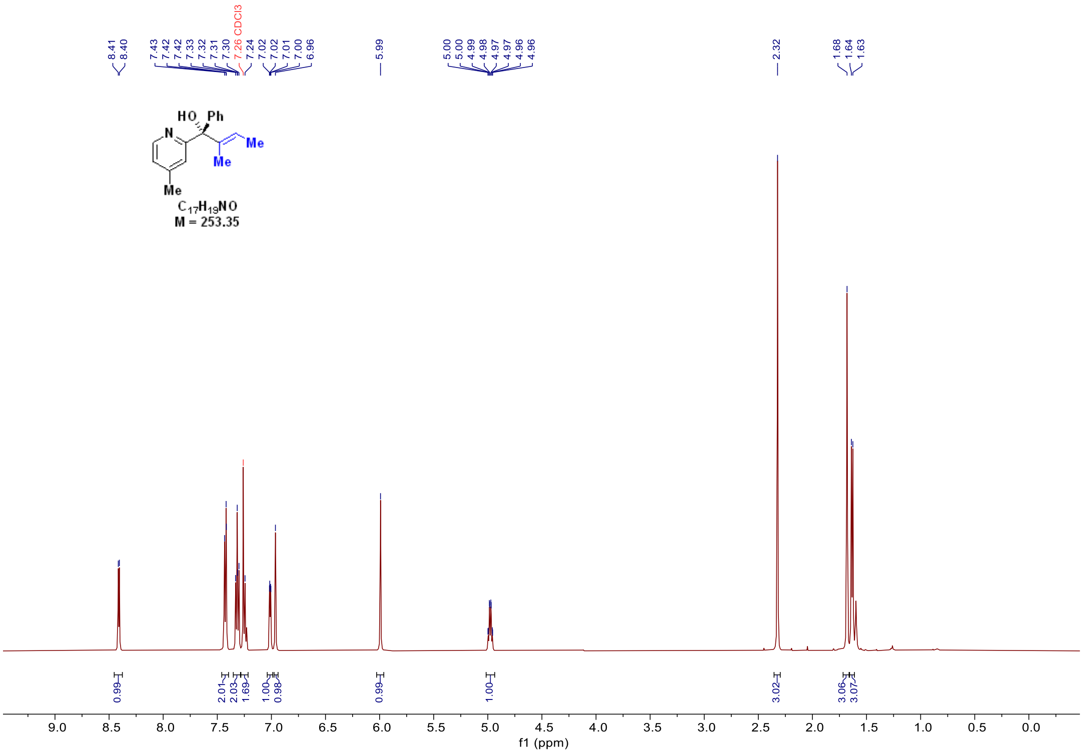

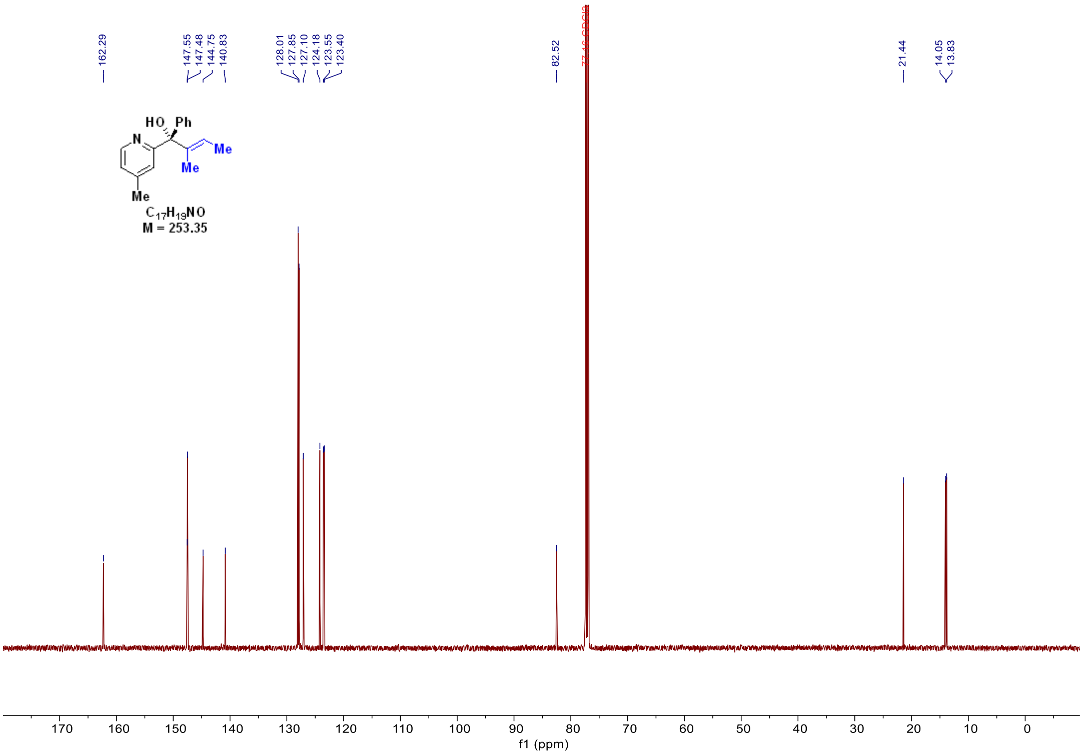

^{1}H NMR (500 MHz, CD_3Cl, 298 K) and ^{13}C NMR (125 MHz, CD_3Cl, 298 K) of **3ep**.

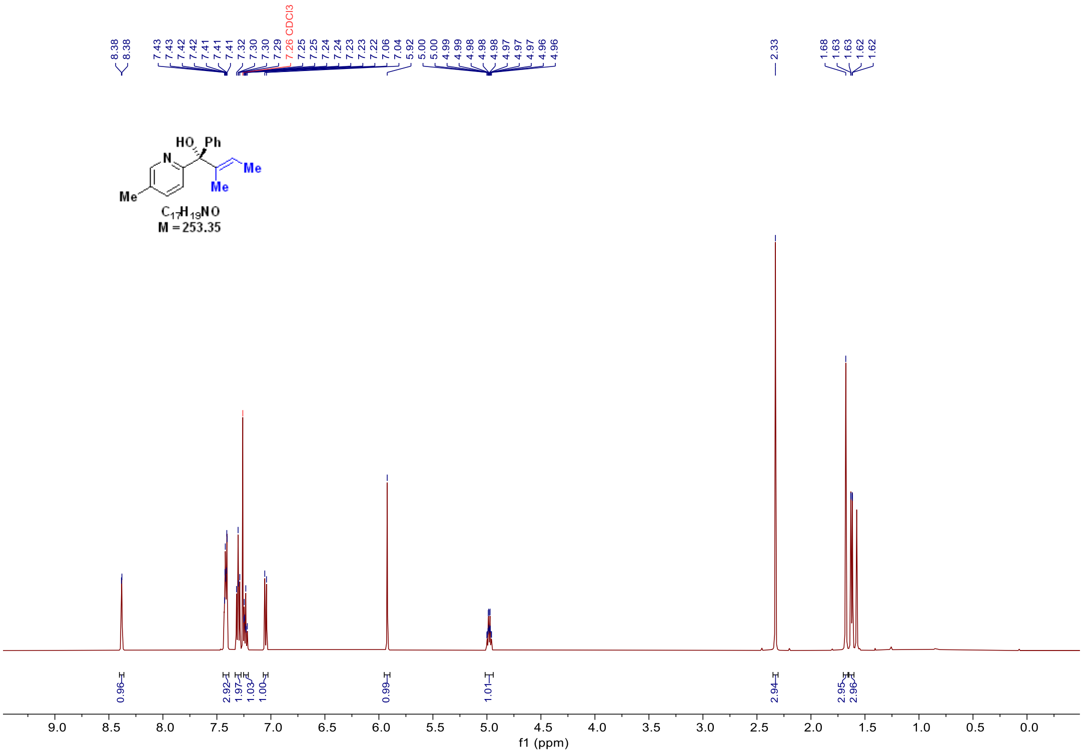

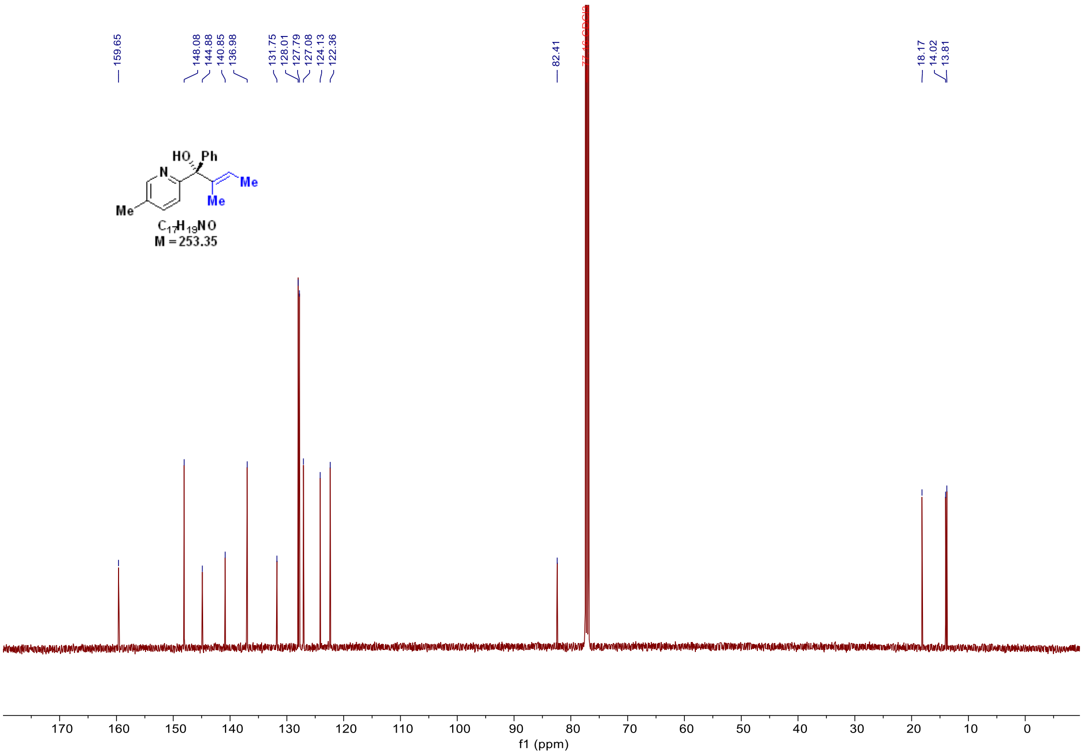

^{1}H NMR (500 MHz, CD_3Cl, 298 K) and ^{13}C NMR (125 MHz, CD_3Cl, 298 K) of **3gp**.

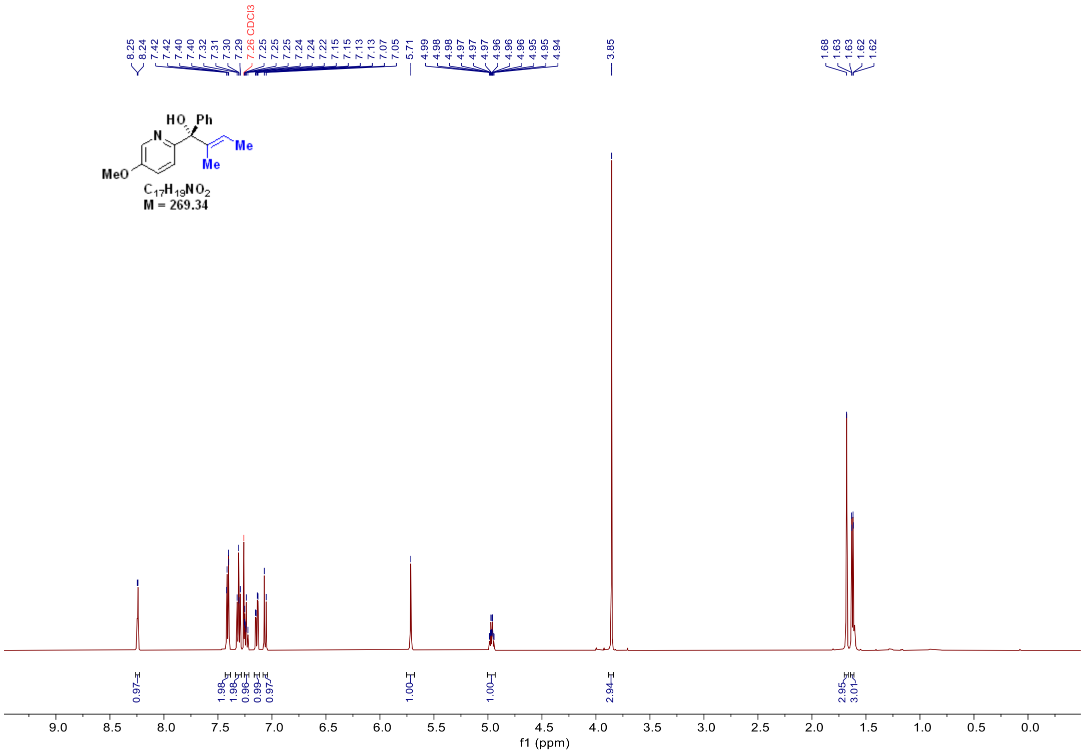

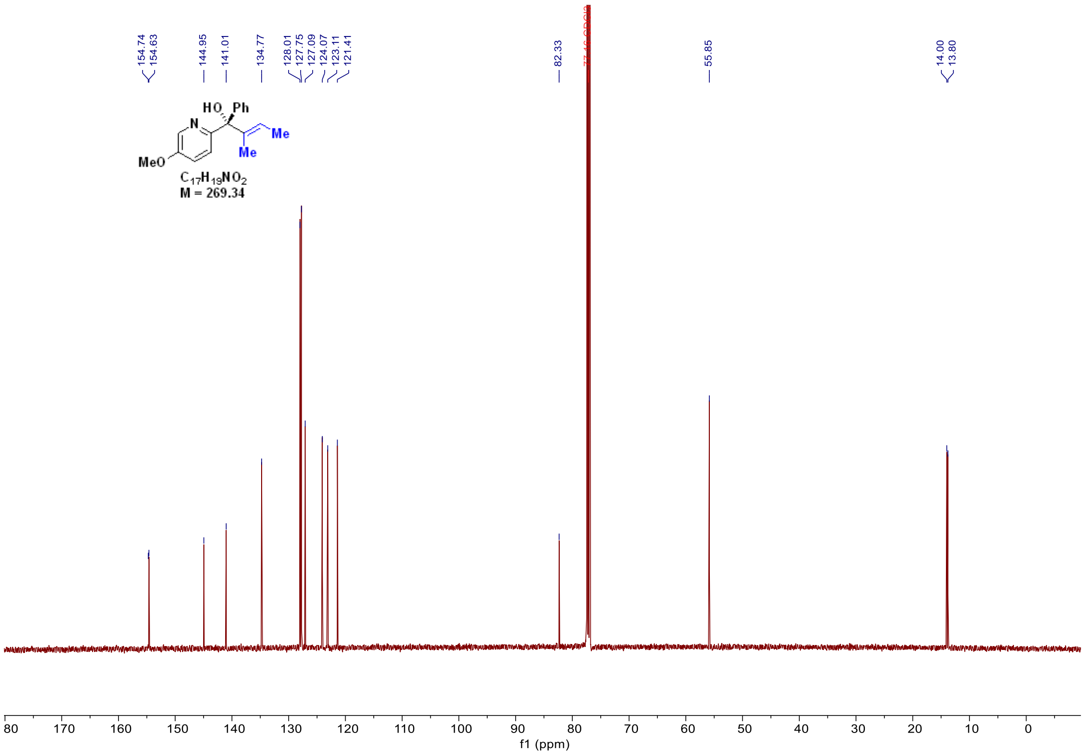

^{1}H NMR (500 MHz, CD_3Cl, 298 K) and ^{13}C NMR (125 MHz, CD_3Cl, 298 K) of **3hp**.

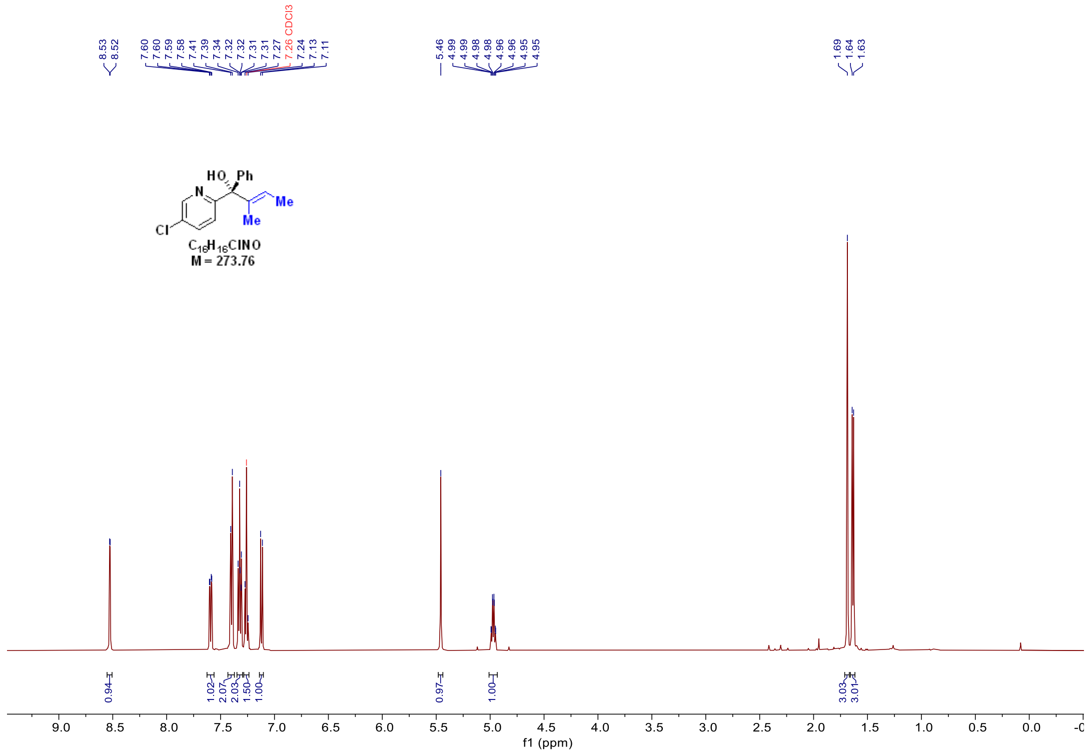

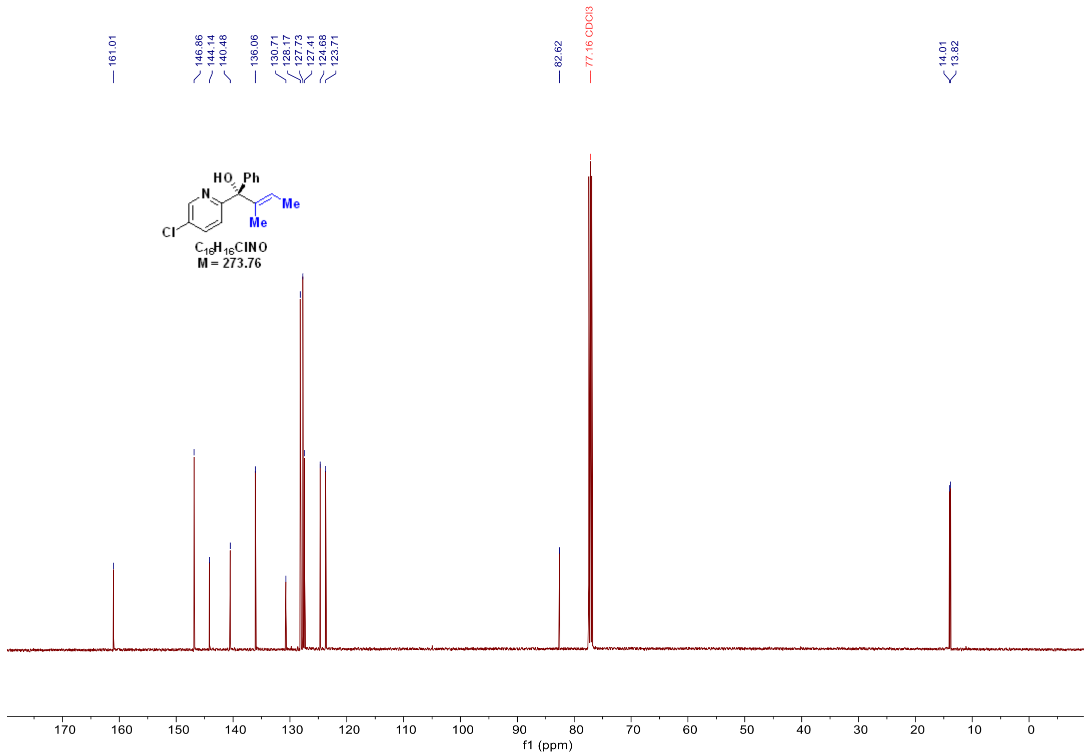

^{1}H NMR (500 MHz, CD_3Cl, 298 K) and ^{13}C NMR (125 MHz, CD_3Cl, 298 K) of **3ip**.

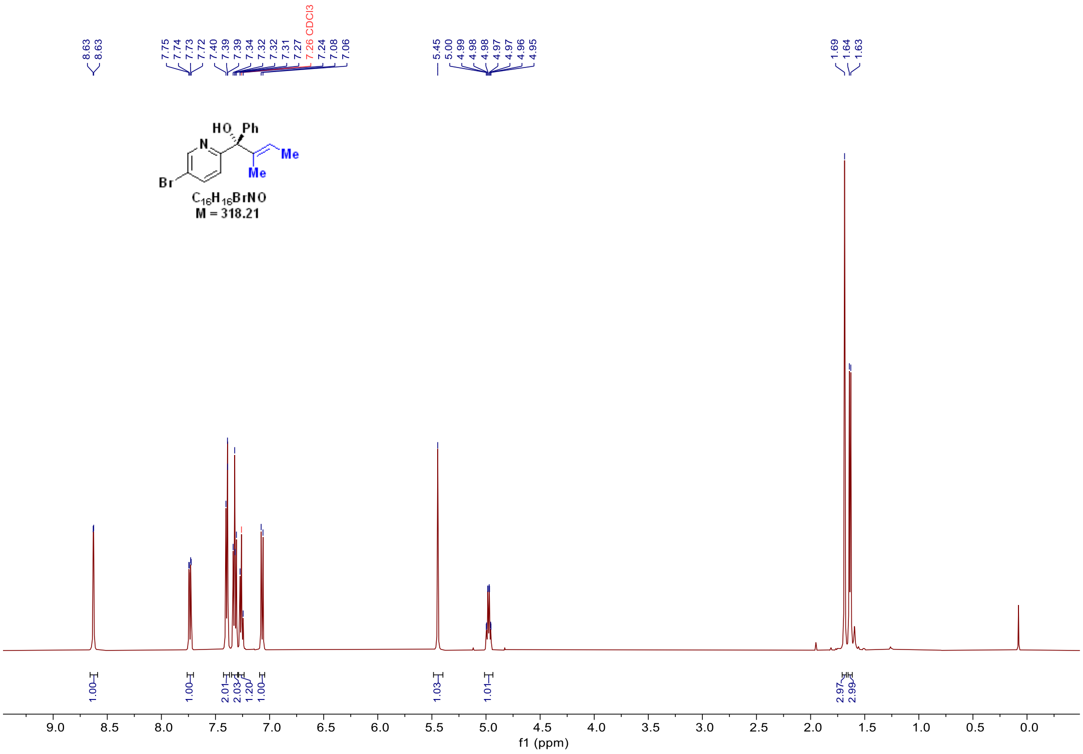

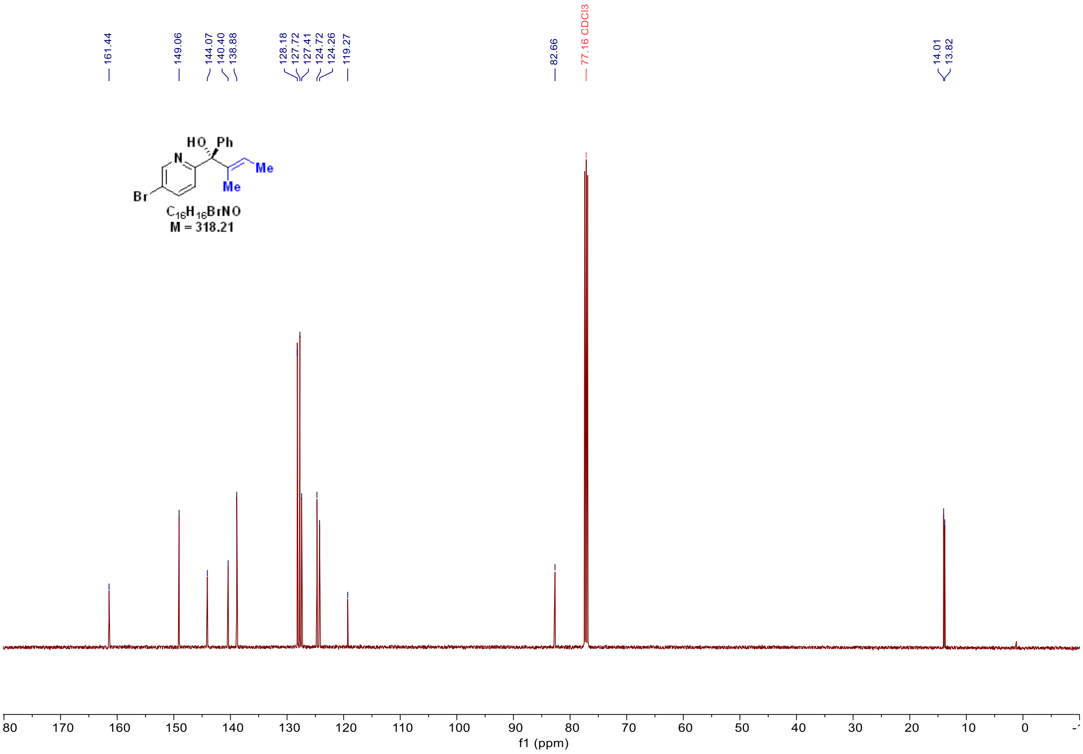

^{1}H NMR (500 MHz, CD_3Cl, 298 K), ^{13}C NMR (125 MHz, CD_3Cl, 298 K) and ^{19}F NMR (471 MHz, CD_3Cl, 298 K) of **3jp**.

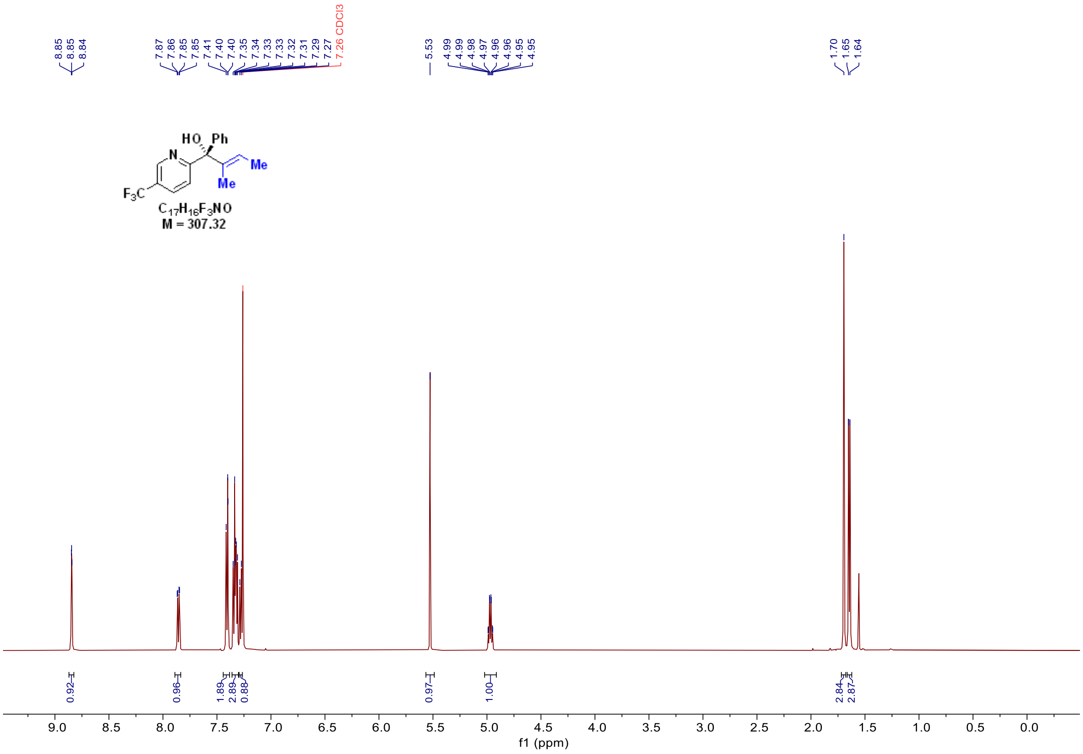

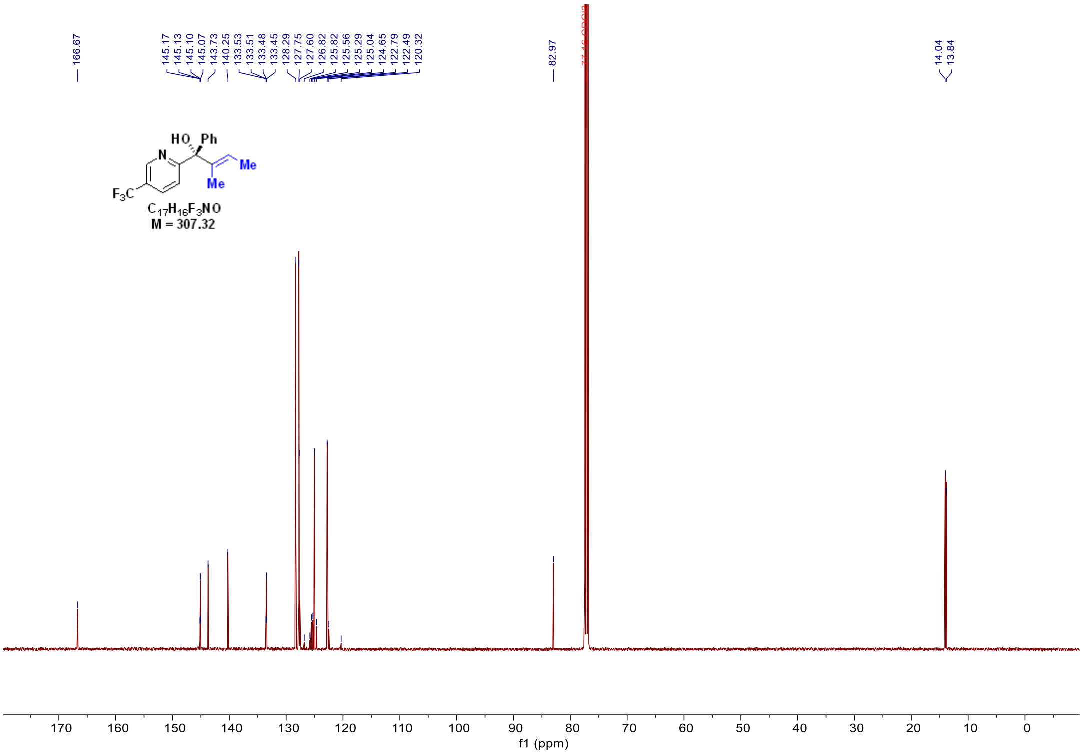

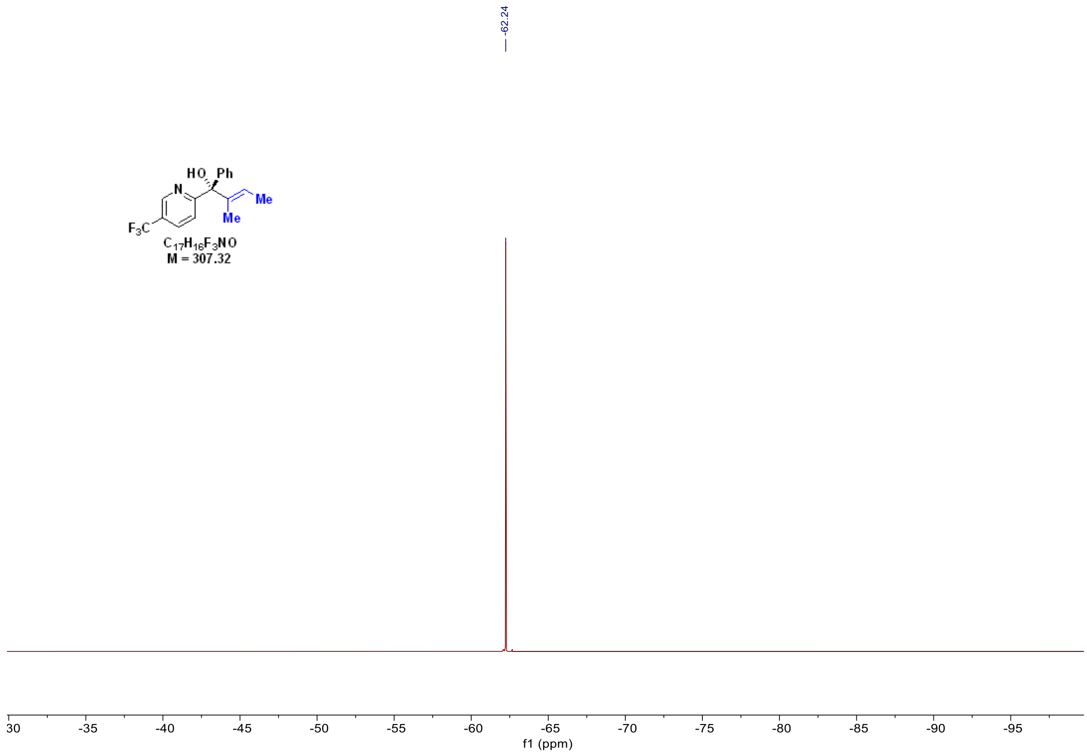
-62.24
HO Ph
N
Me
Me
F_3C
$C_{17}H_{16}F_3NO$
M = 307.32
30 -35 -40 -45 -50 -55 -60 -65 -70 -75 -80 -85 -90 -95
f1 (ppm)

^{1}H NMR (500 MHz, CD_3Cl, 298 K) and ^{13}C NMR (125 MHz, CD_3Cl, 298 K) of **3kp**.

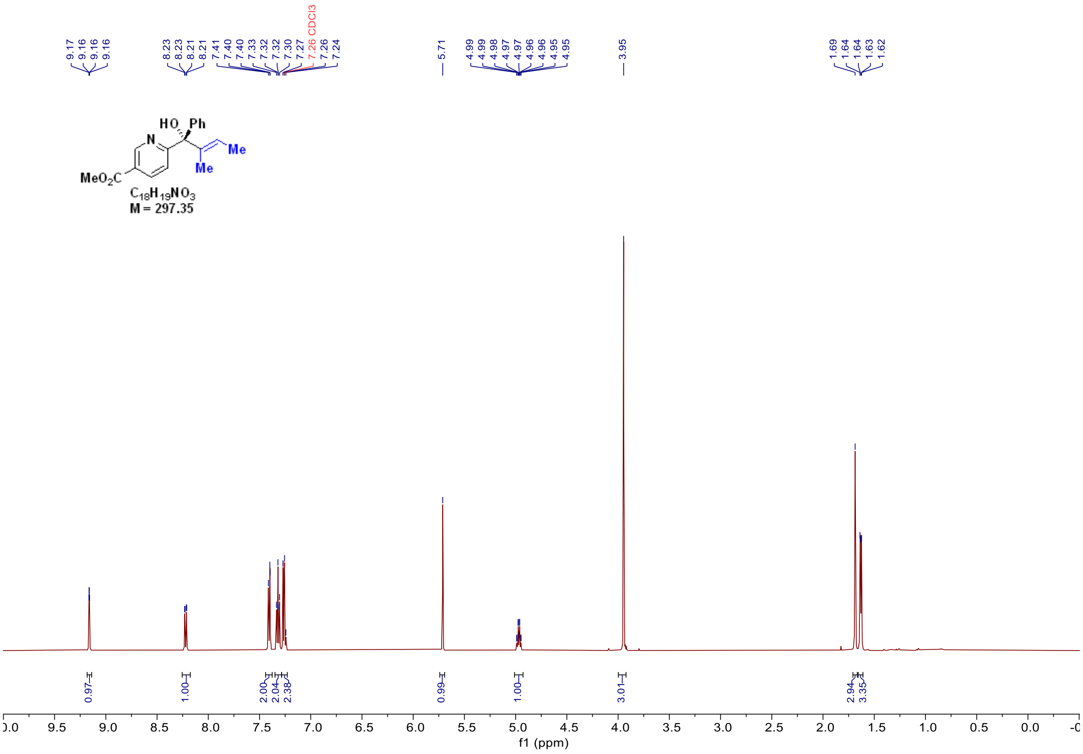

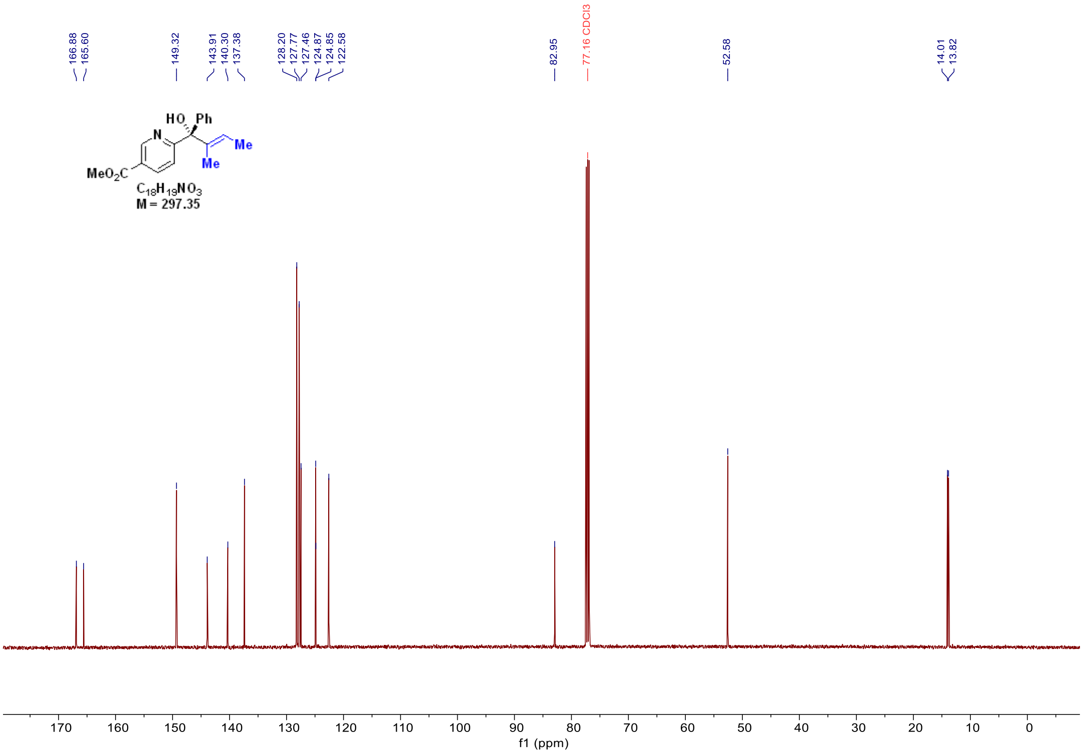

^{1}H NMR (500 MHz, CD_3Cl, 298 K) and ^{13}C NMR (125 MHz, CD_3Cl, 298 K) of **3la**.

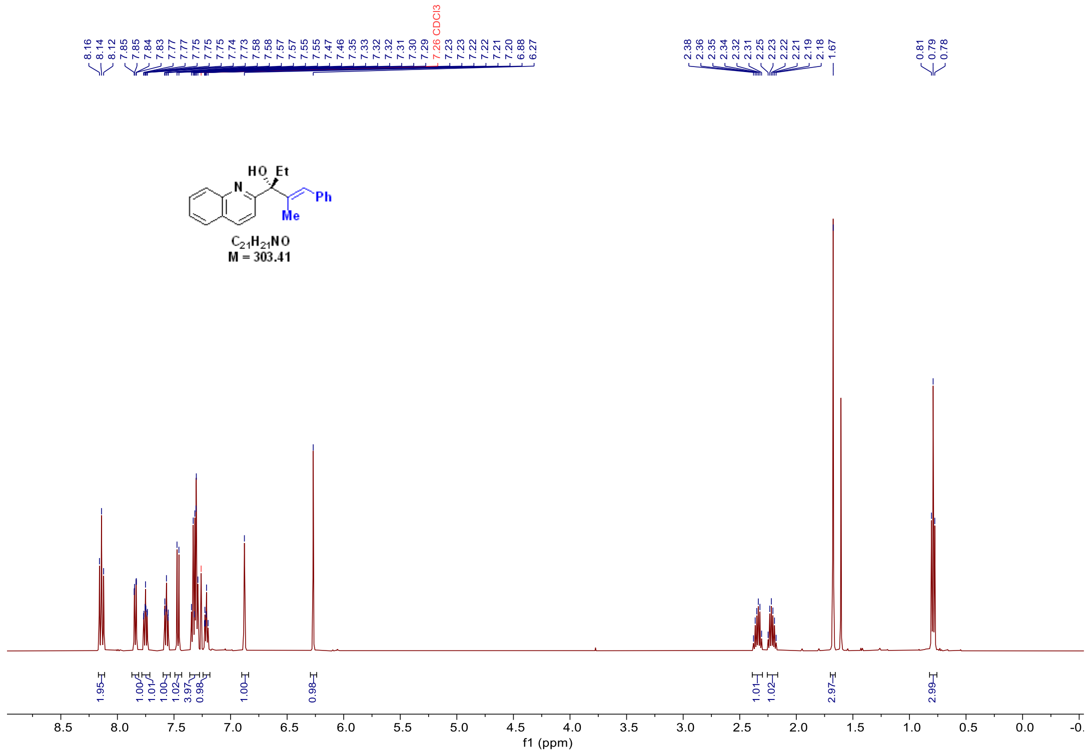

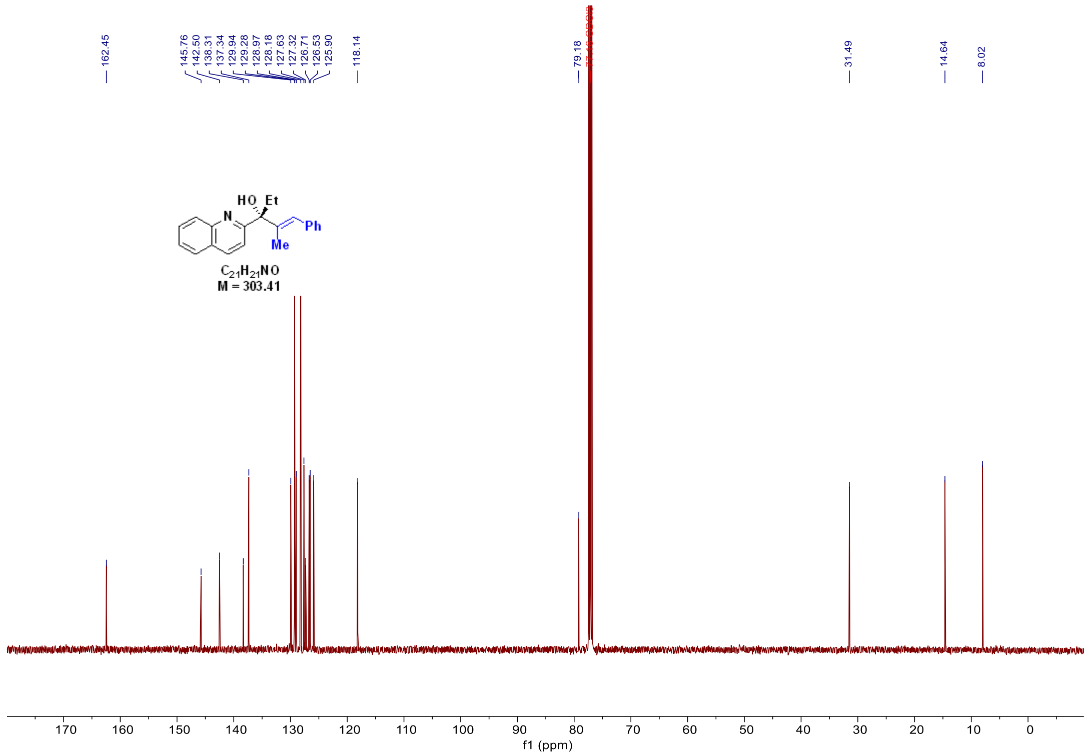

^{1}H NMR (500 MHz, CD_3Cl, 298 K) and ^{13}C NMR (125 MHz, CD_3Cl, 298 K) of **3mp**.

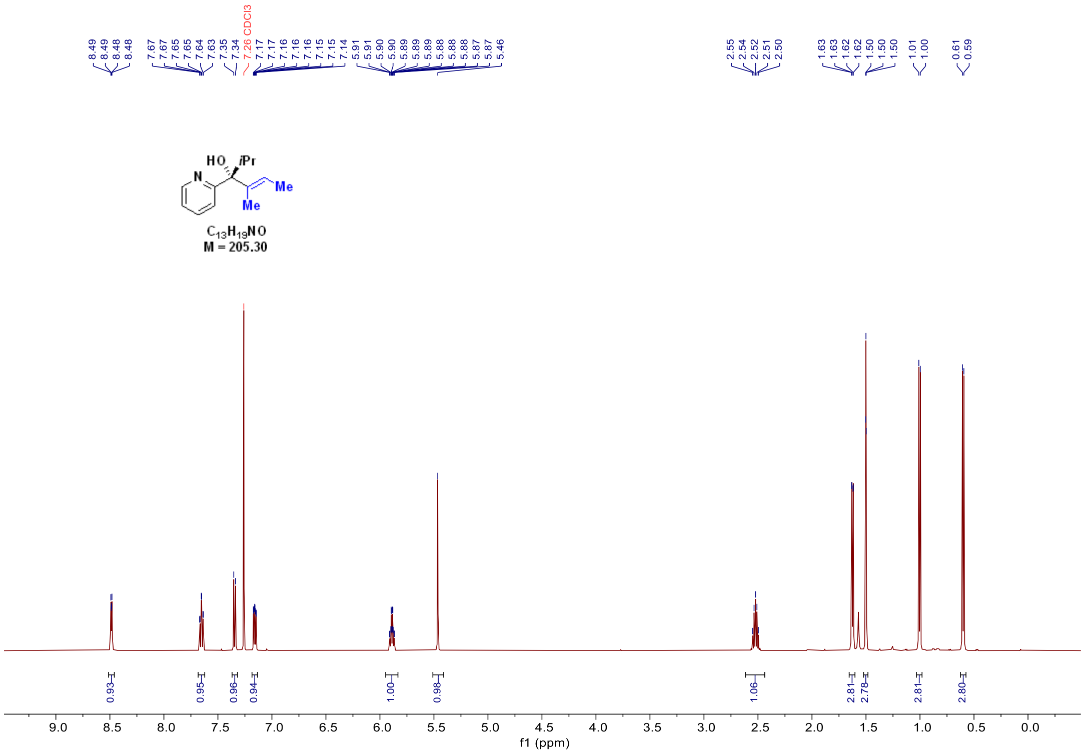

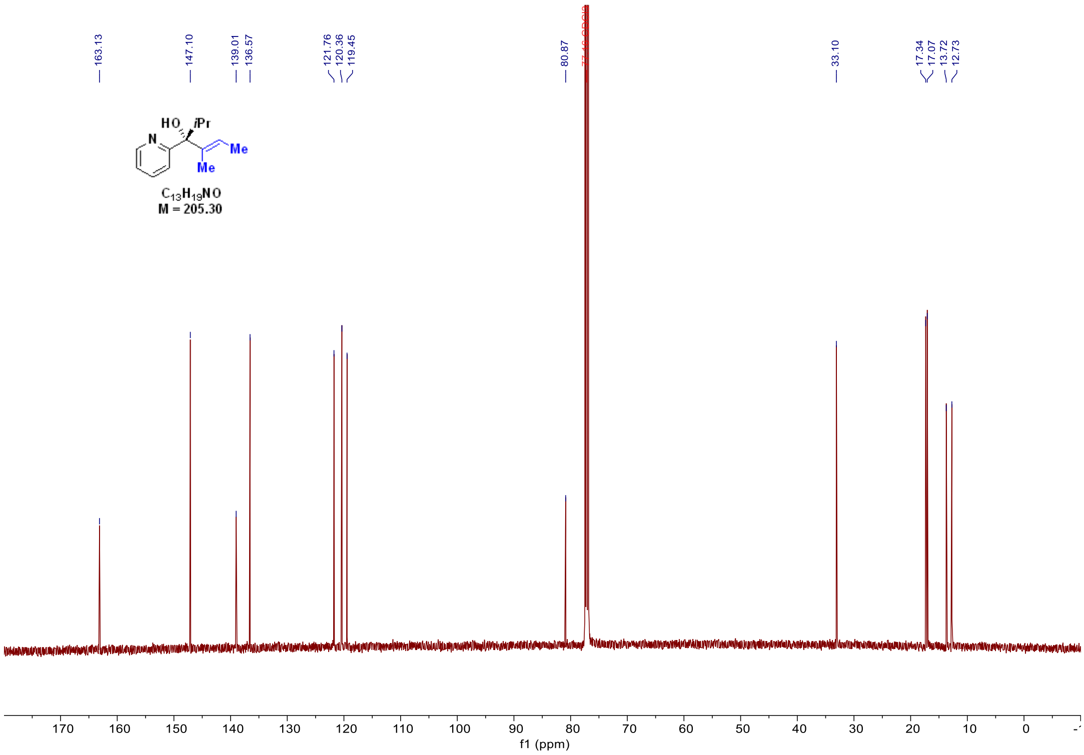

^{1}H NMR (500 MHz, CD_3Cl, 298 K) and ^{13}C NMR (125 MHz, CD_3Cl, 298 K) of **3np**.

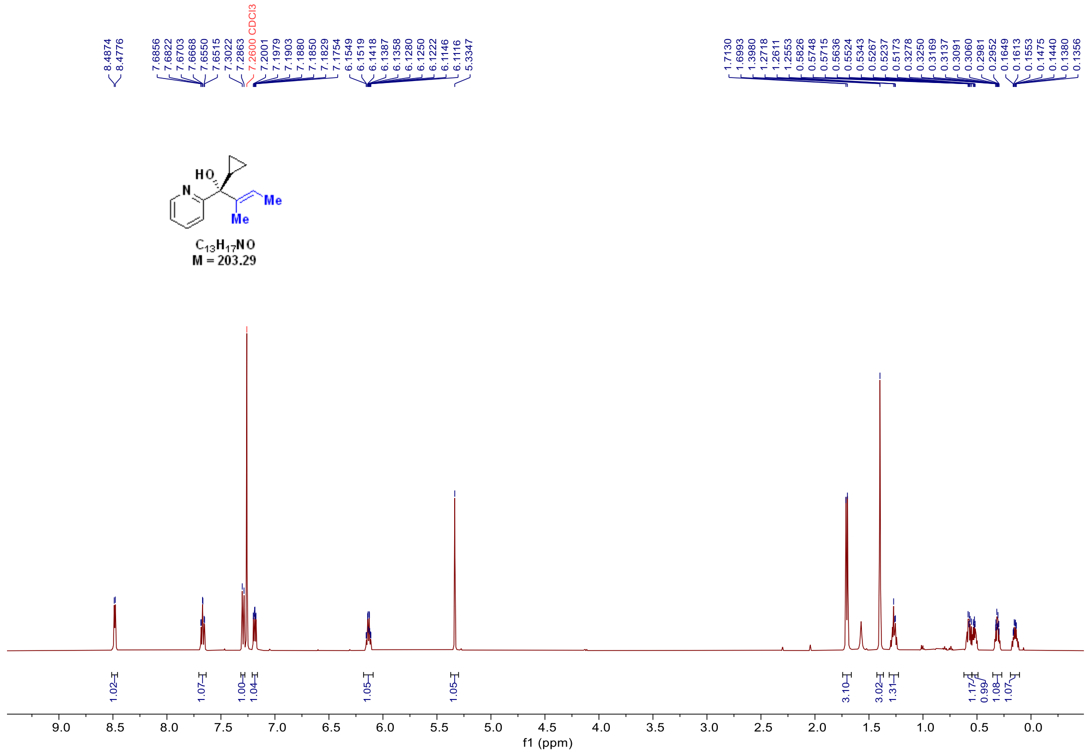

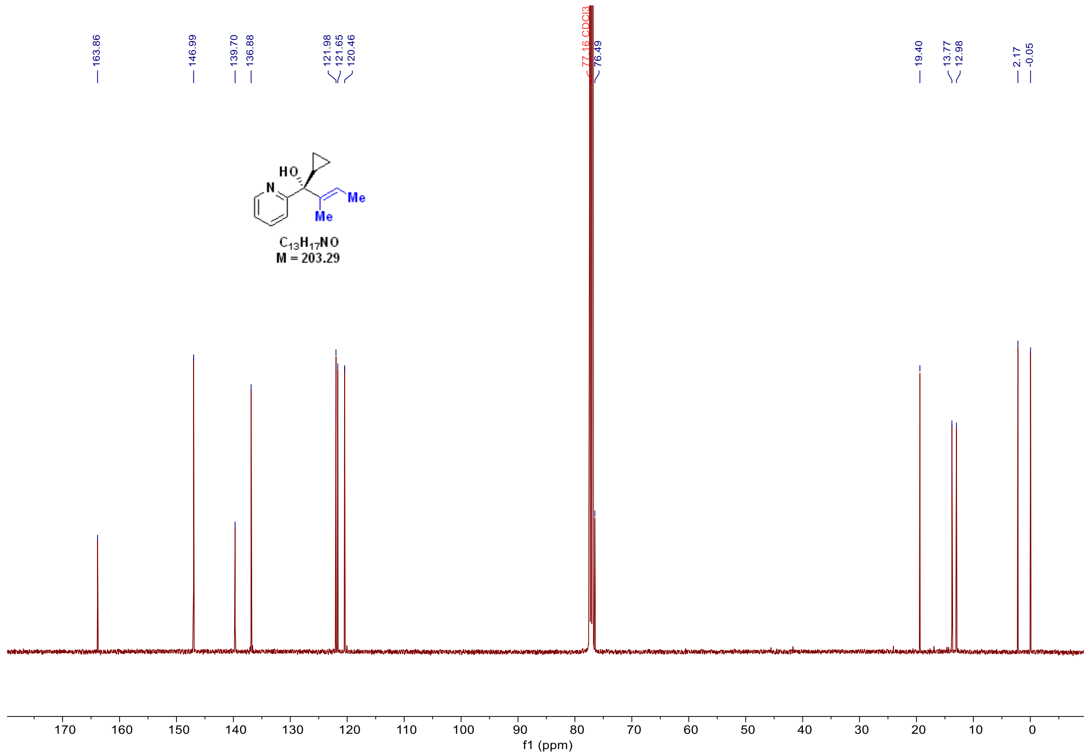

^{1}H NMR (500 MHz, CD_3Cl, 298 K) and ^{13}C NMR (125 MHz, CD_3Cl, 298 K) of **3op**.

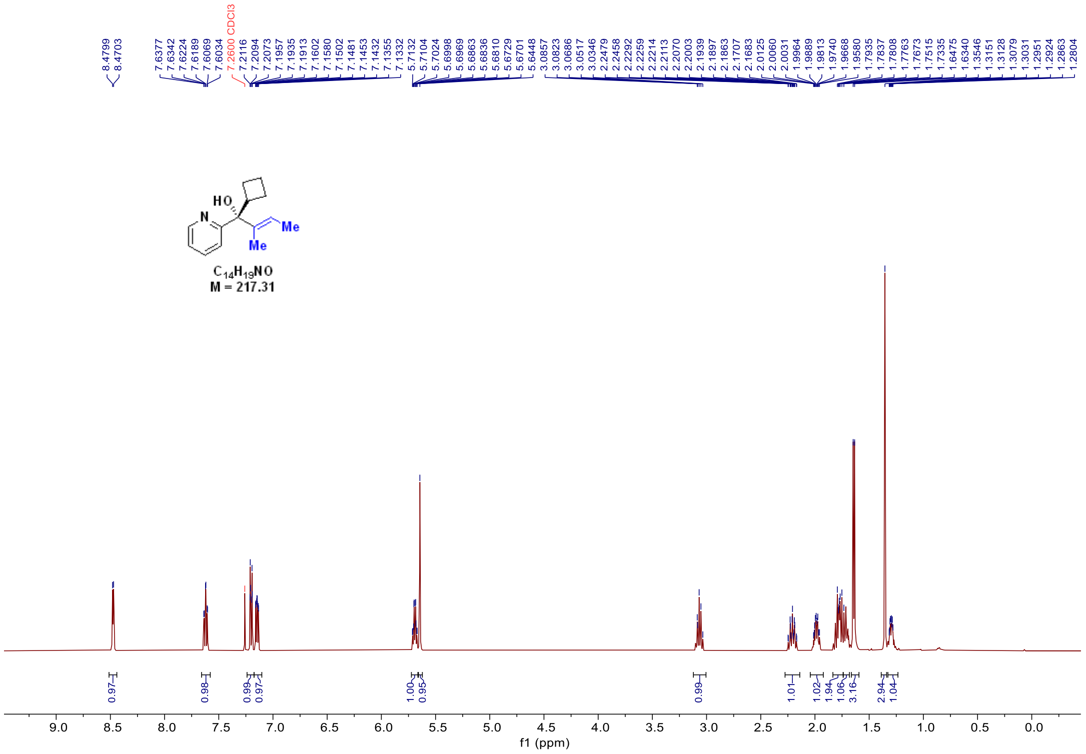

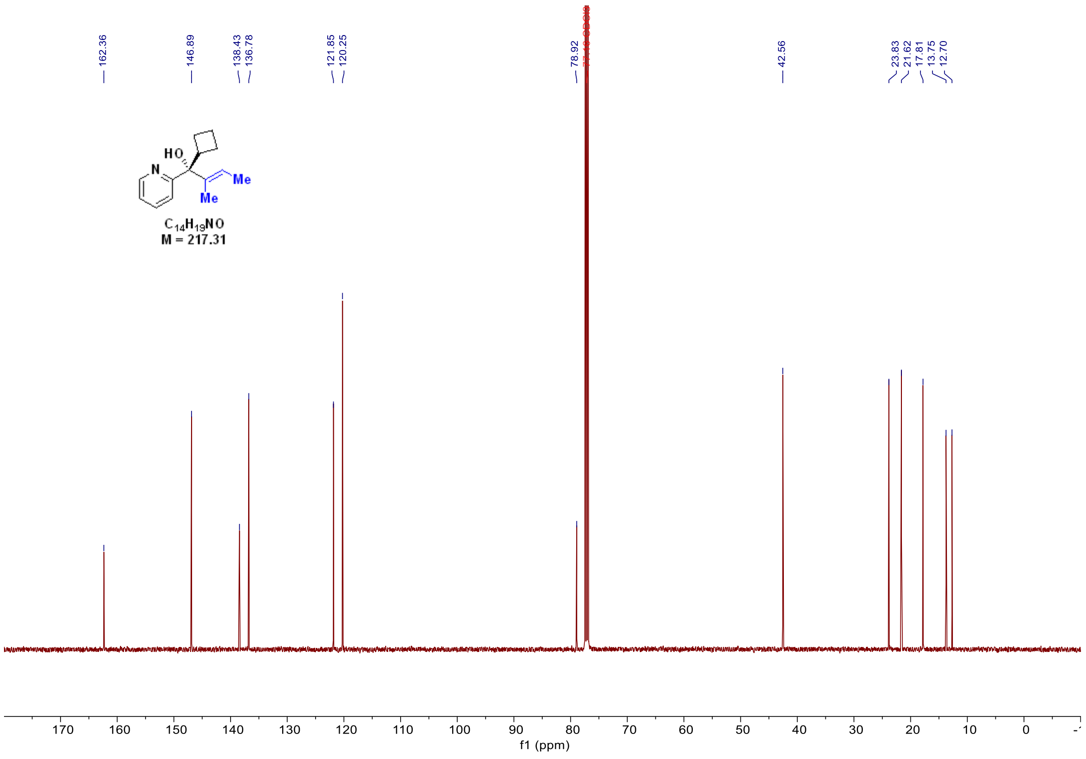

^{1}H NMR (500 MHz, CD_3Cl, 298 K) and ^{13}C NMR (125 MHz, CD_3Cl, 298 K) of **3pp**.

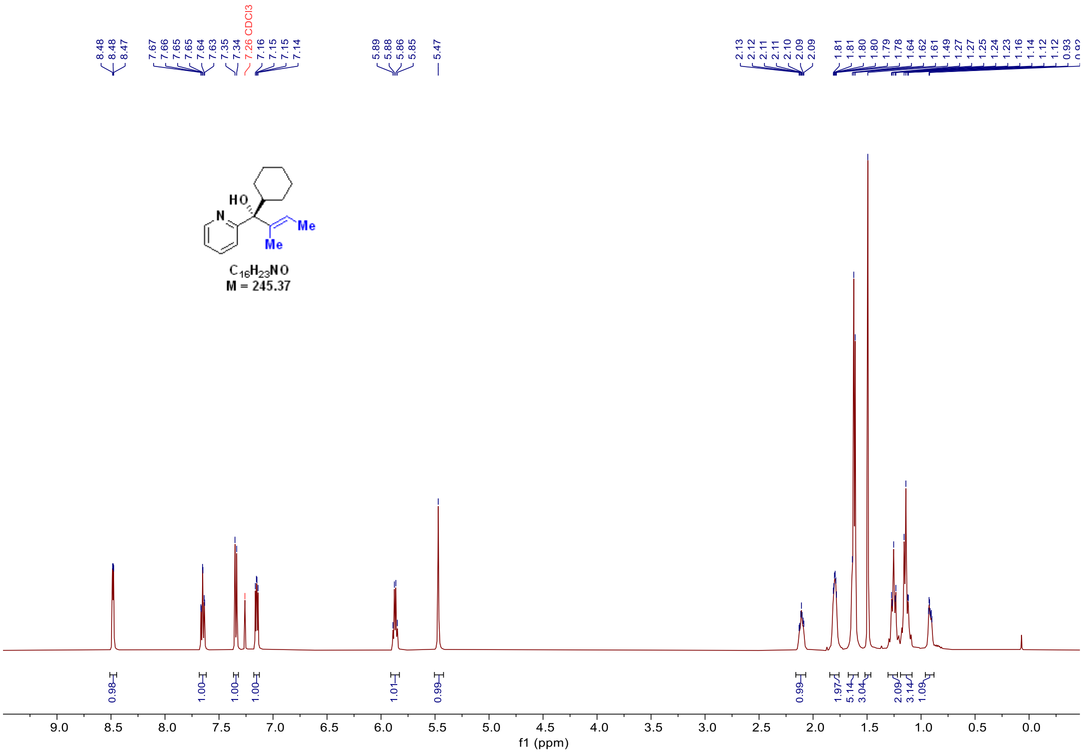

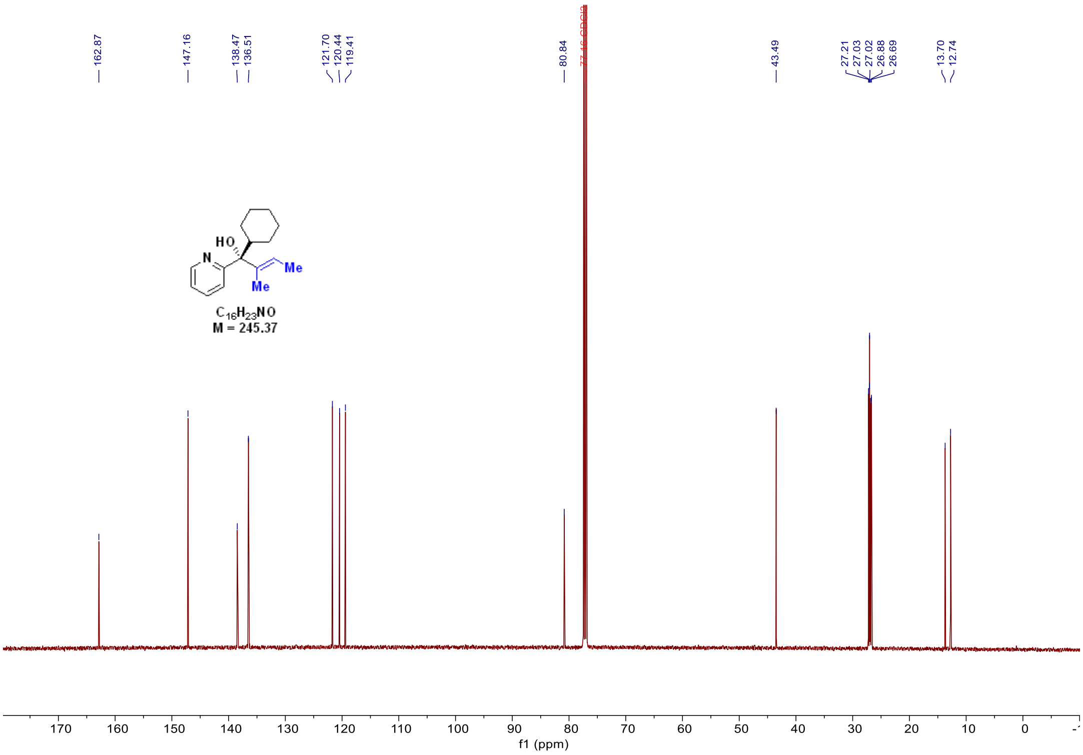

^{1}H NMR (500 MHz, CD_3Cl, 298 K) and ^{13}C NMR (125 MHz, CD_3Cl, 298 K) of **3qp**.

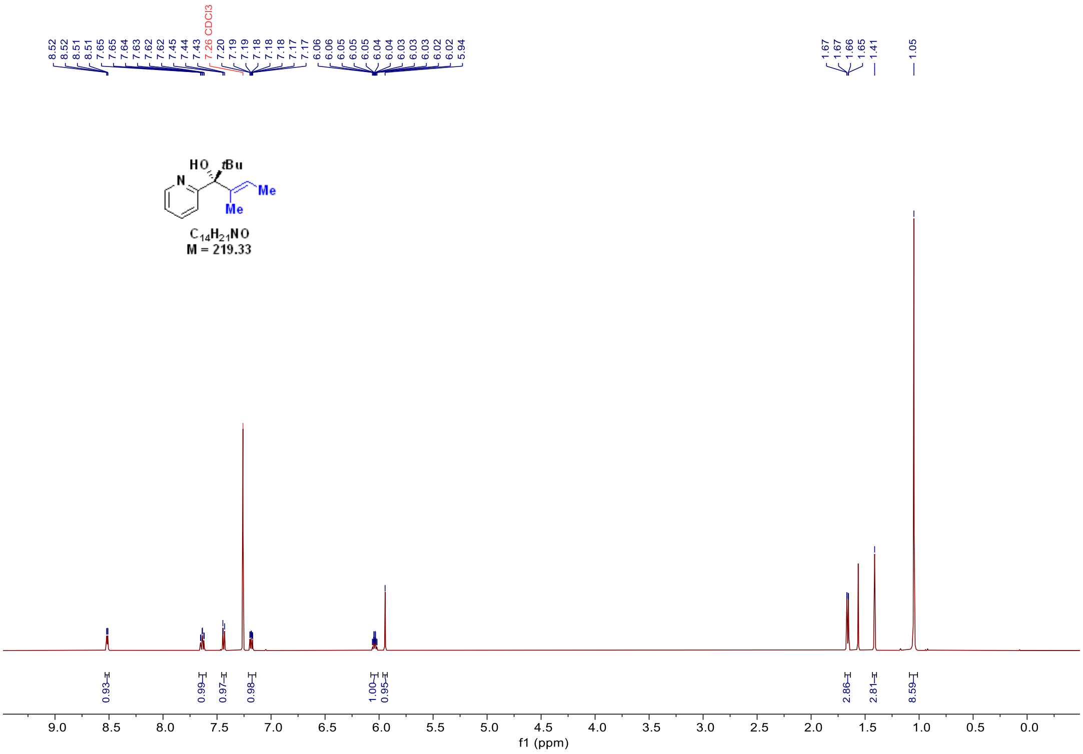

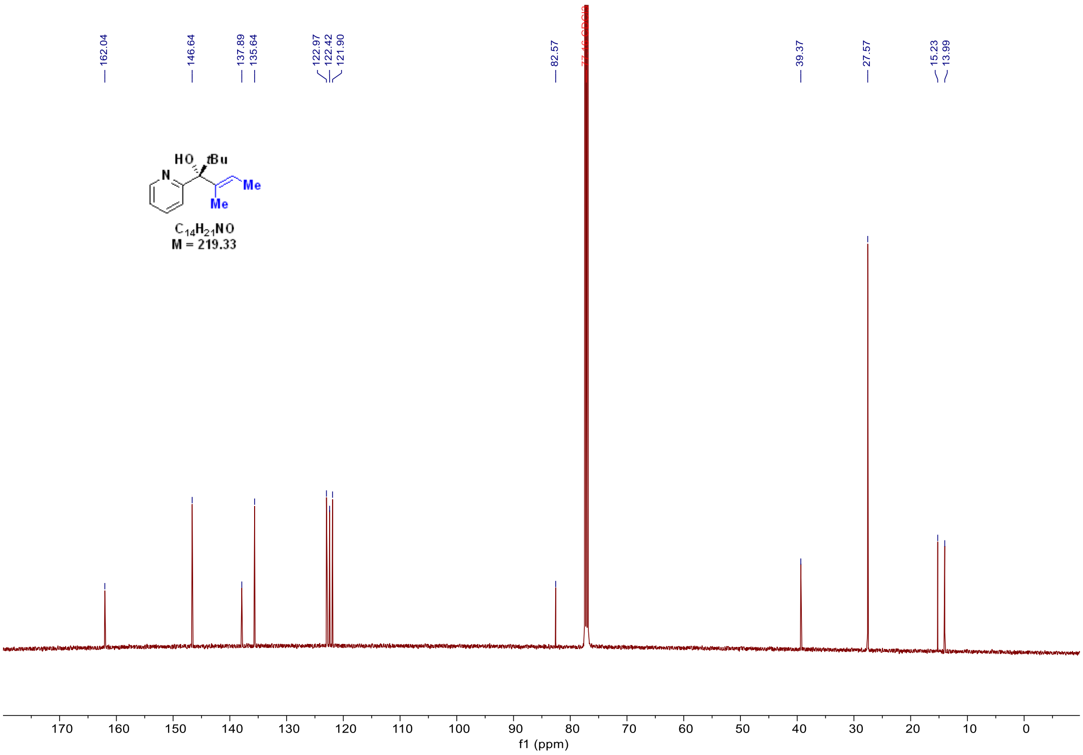

^{1}H NMR (500 MHz, CD_3Cl, 298 K) and ^{13}C NMR (125 MHz, CD_3Cl, 298 K) of **3ra**.

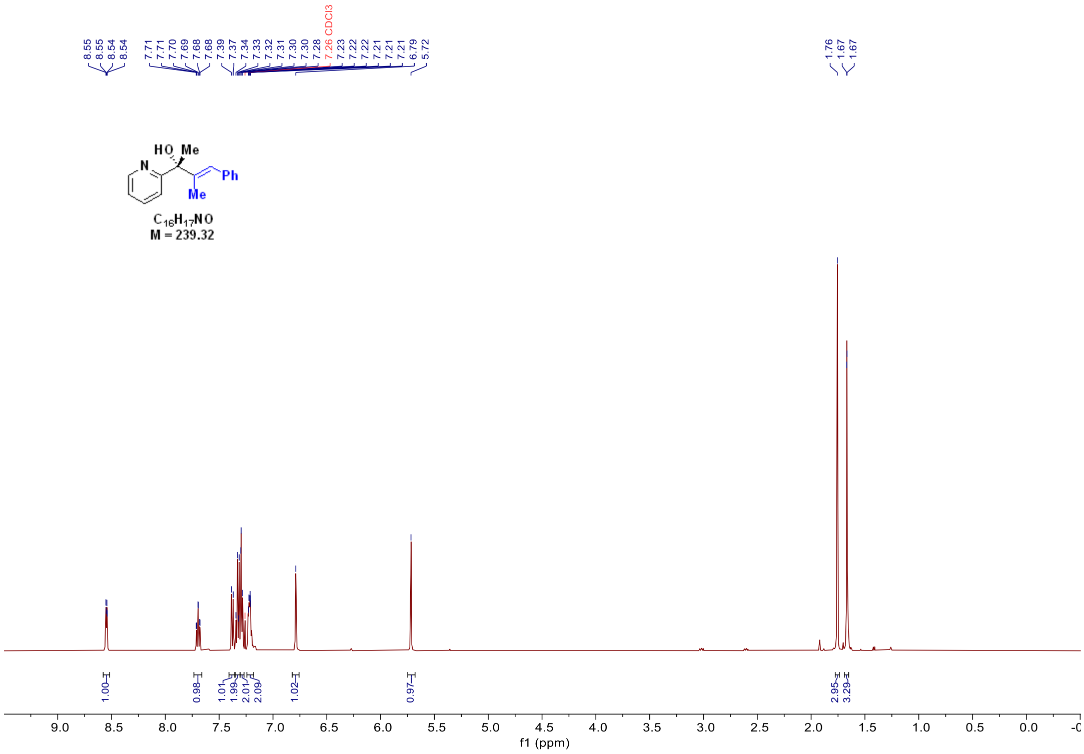

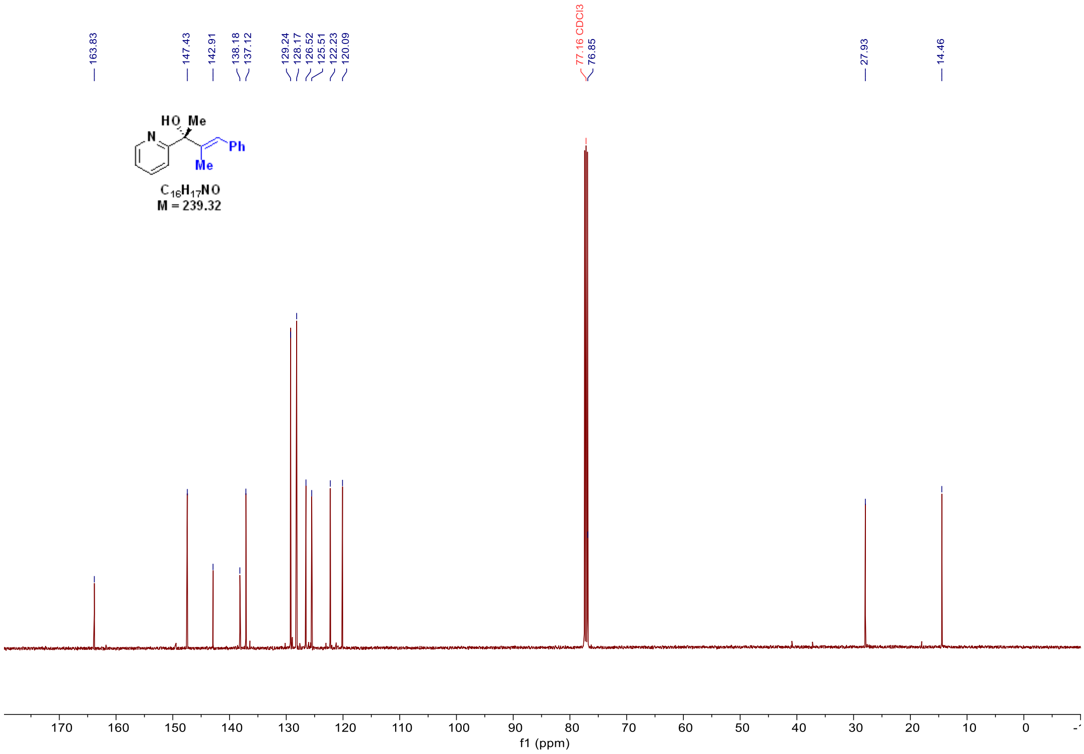

^{1}H NMR (500 MHz, CD_3Cl, 298 K), ^{13}C NMR (125 MHz, CD_3Cl, 298 K) and HMBC of **3sp**.

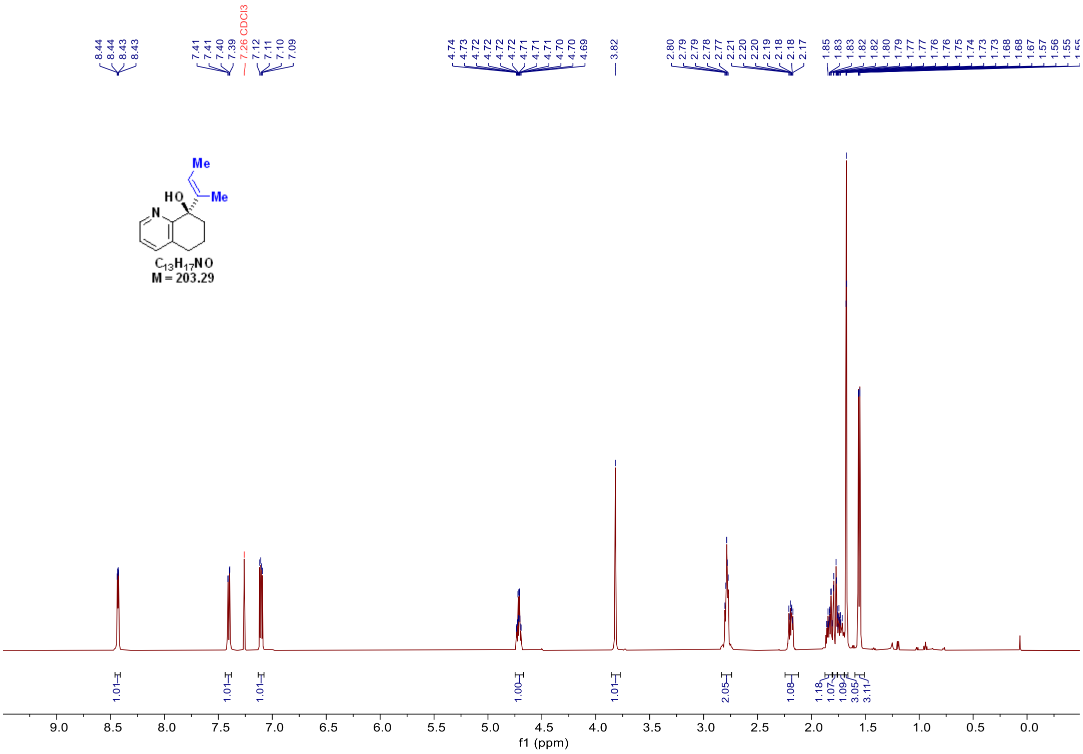

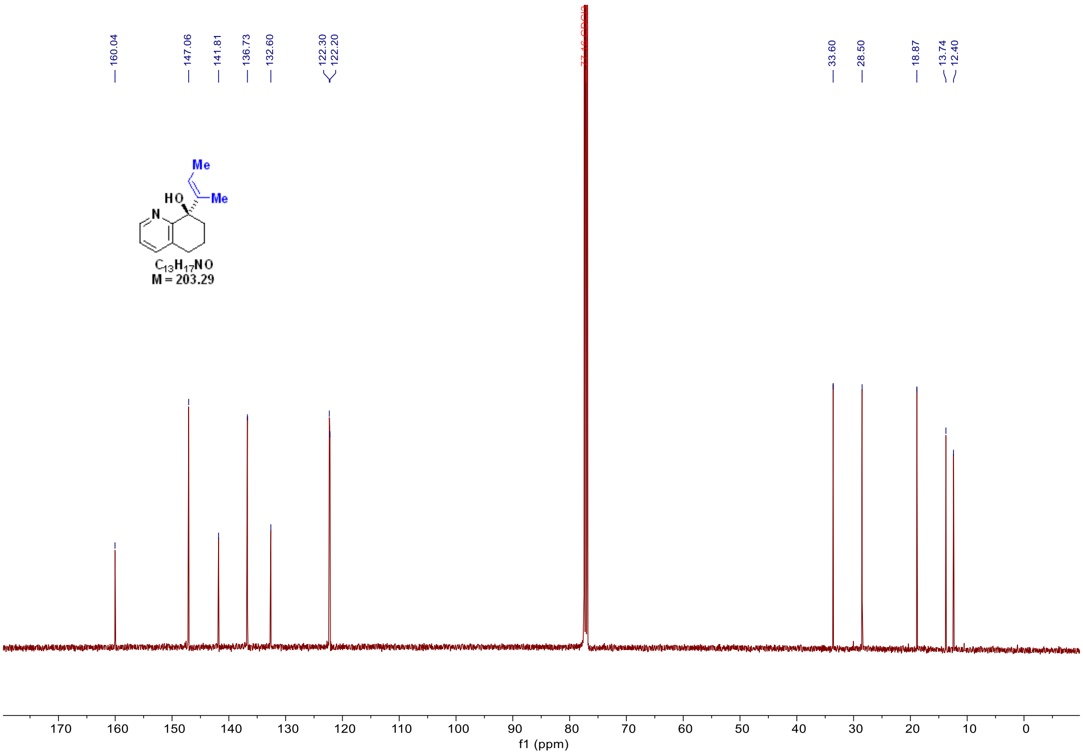

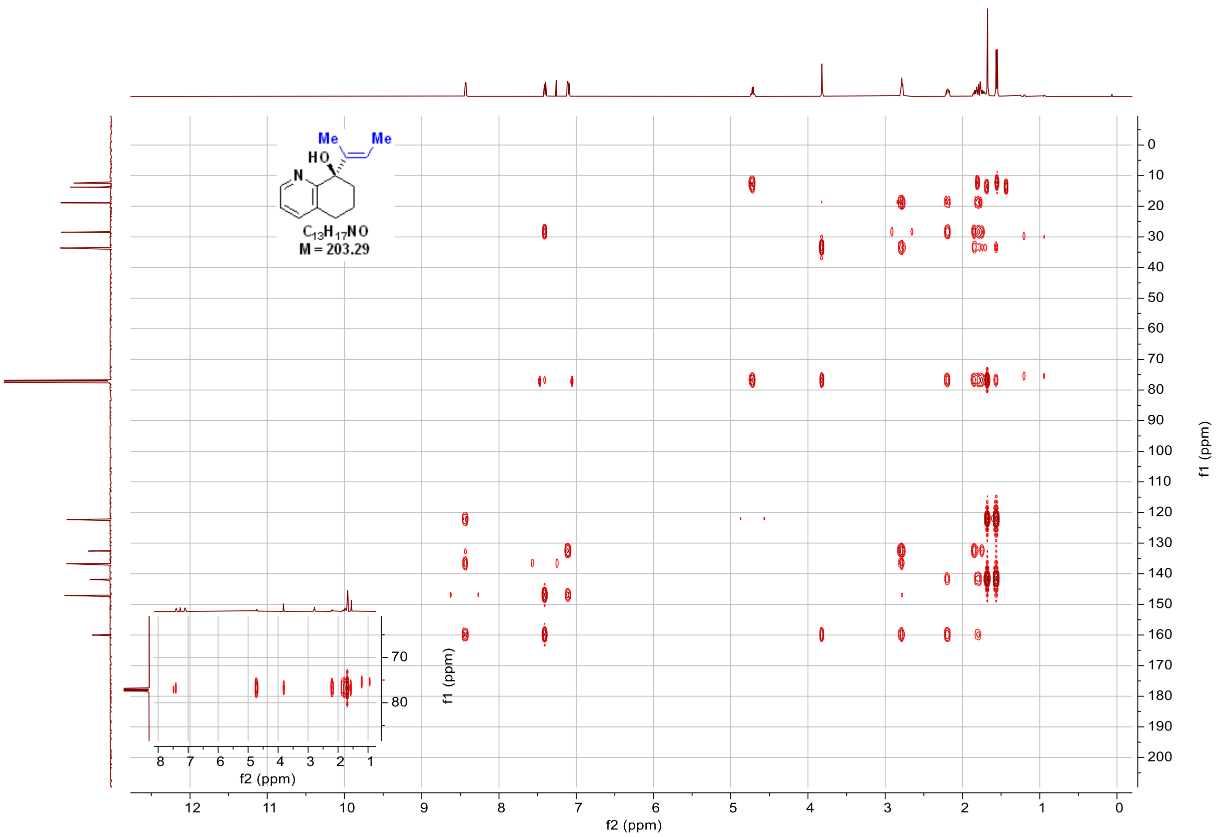

Me
Me
HO
N
$C_{13}H_{17}NO$
M = 203.29
f2 (ppm)
f1 (ppm)

^{1}H NMR (500 MHz, CD_3Cl, 298 K) and ^{13}C NMR (125 MHz, CD_3Cl, 298 K) of **3tp**.

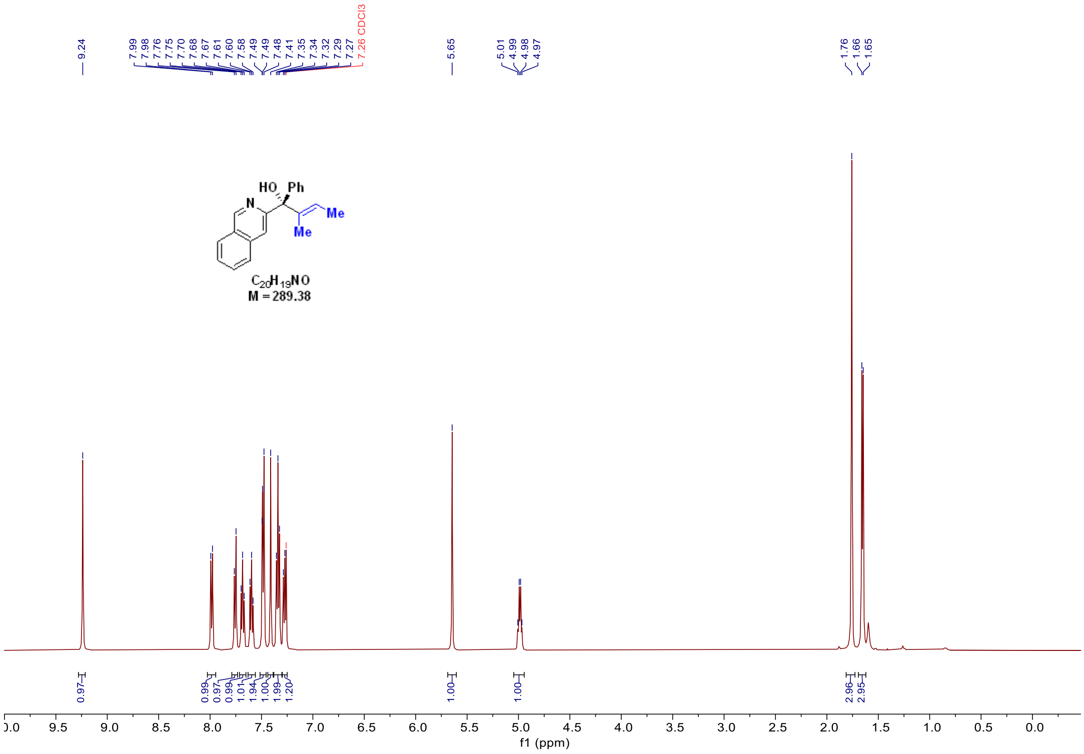

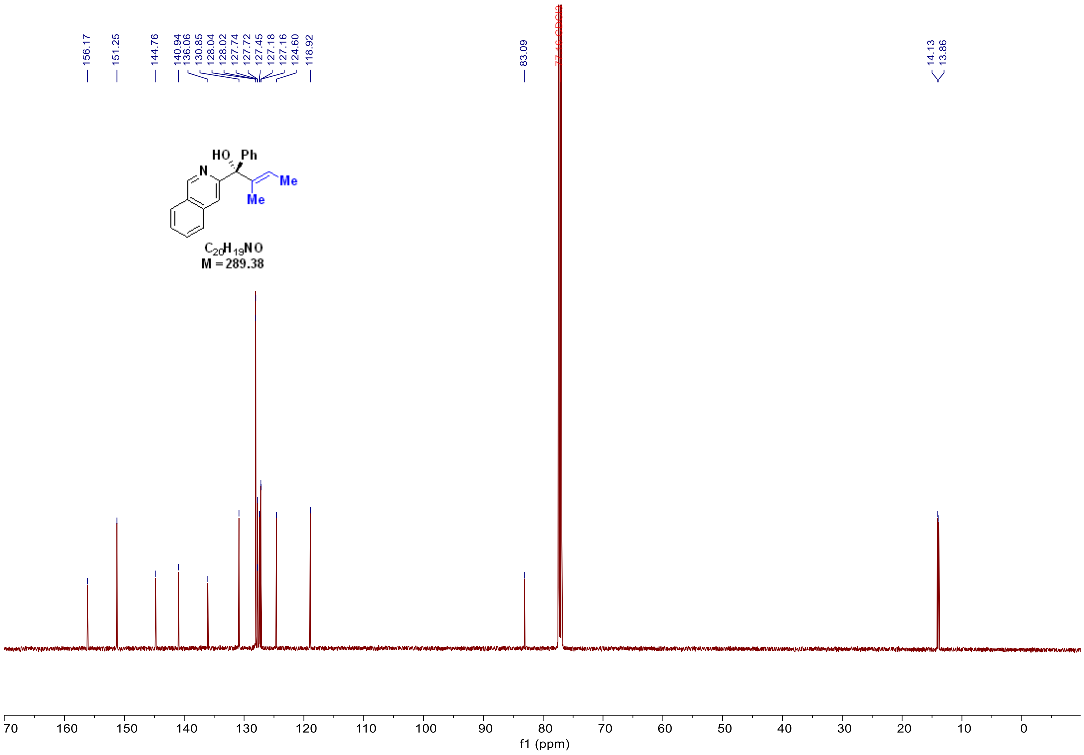

^{1}H NMR (500 MHz, CD_3Cl, 298 K) and ^{13}C NMR (125 MHz, CD_3Cl, 298 K) of **2-phenyl-1-(quinolin-2-yl)propan-1-one**.

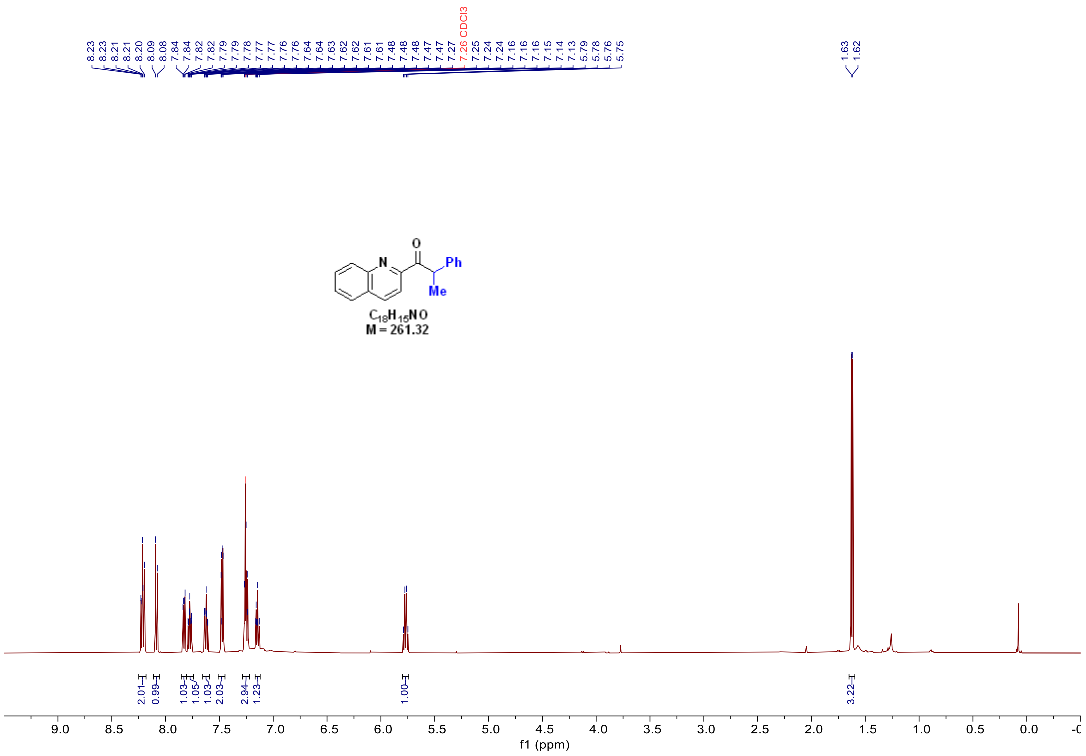

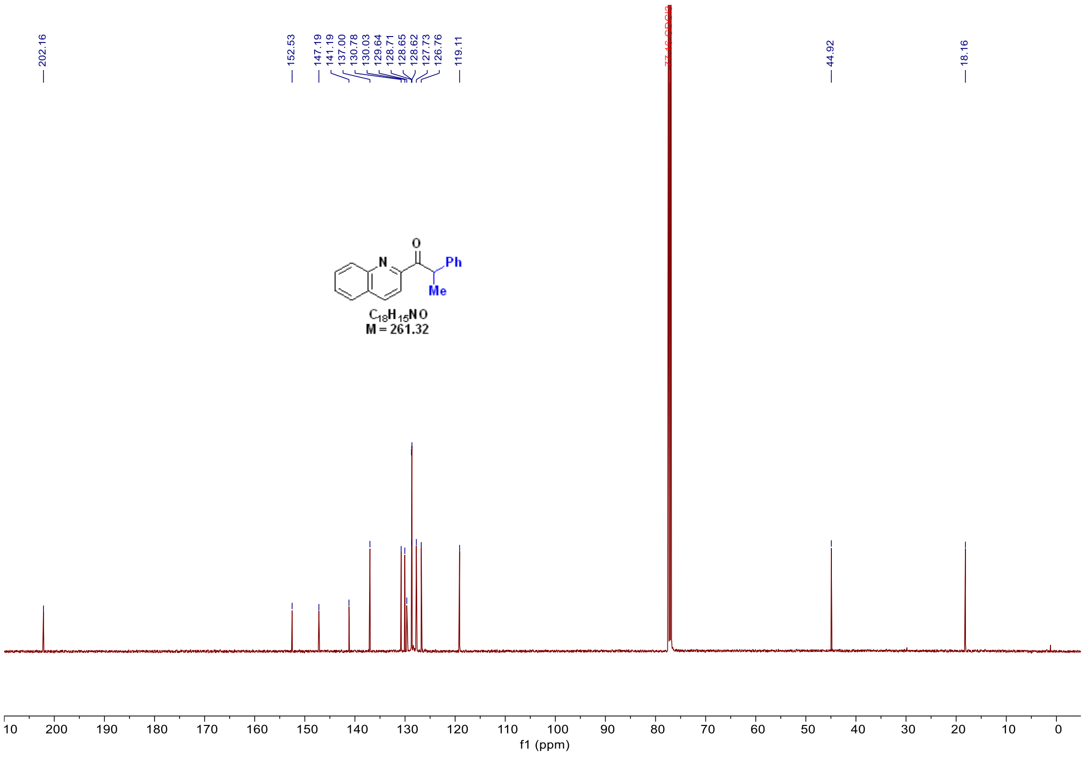

^{1}H NMR (500 MHz, CD_3Cl, 298 K) and ^{13}C NMR (125 MHz, CD_3Cl, 298 K) of **1-(quinolin-2-yl)ethan-1-one**.

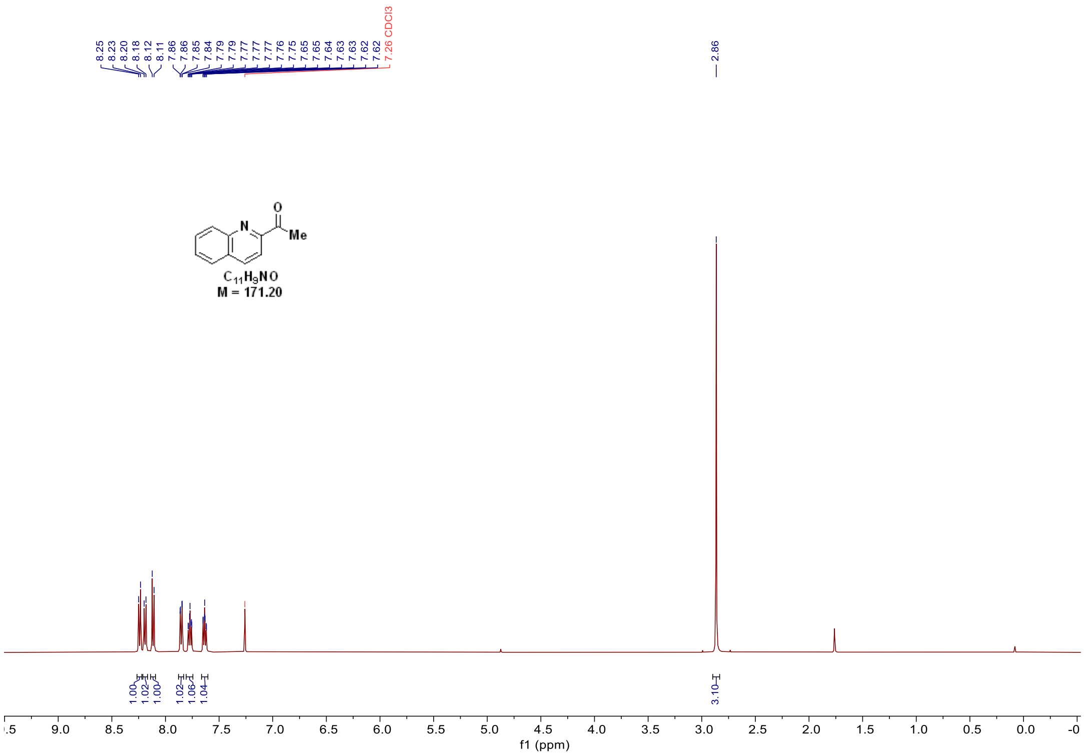

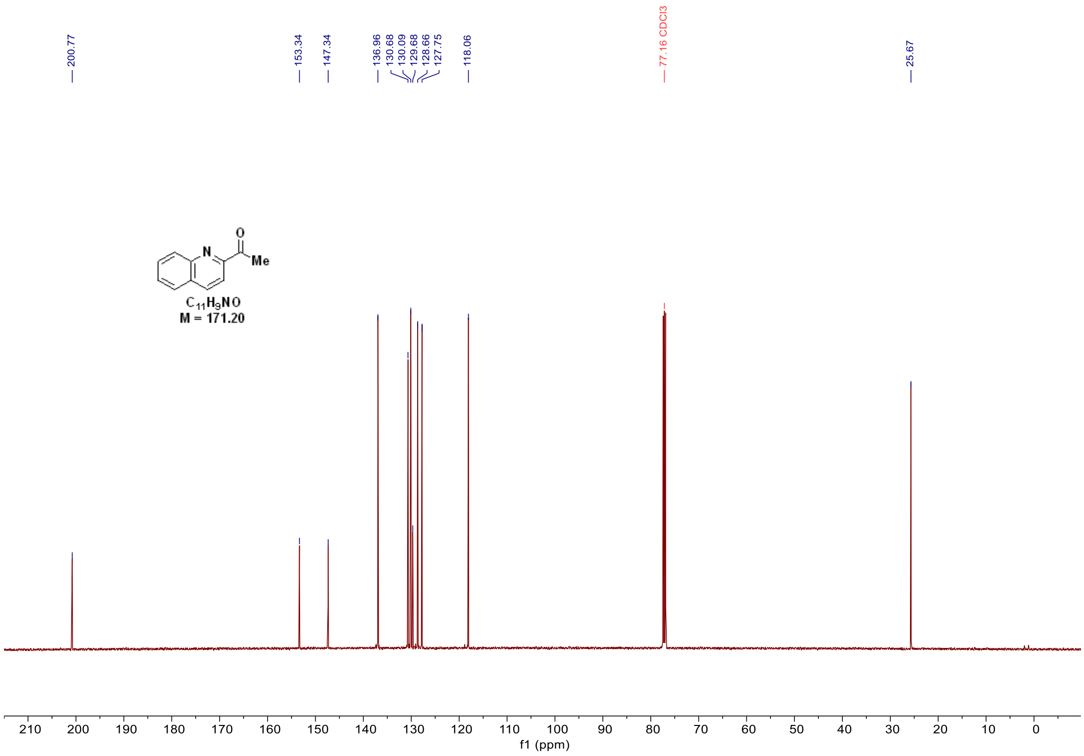

^{1}H NMR (500 MHz, CD_3Cl, 298 K) and ^{13}C NMR (125 MHz, CD_3Cl, 298 K) of **6**.

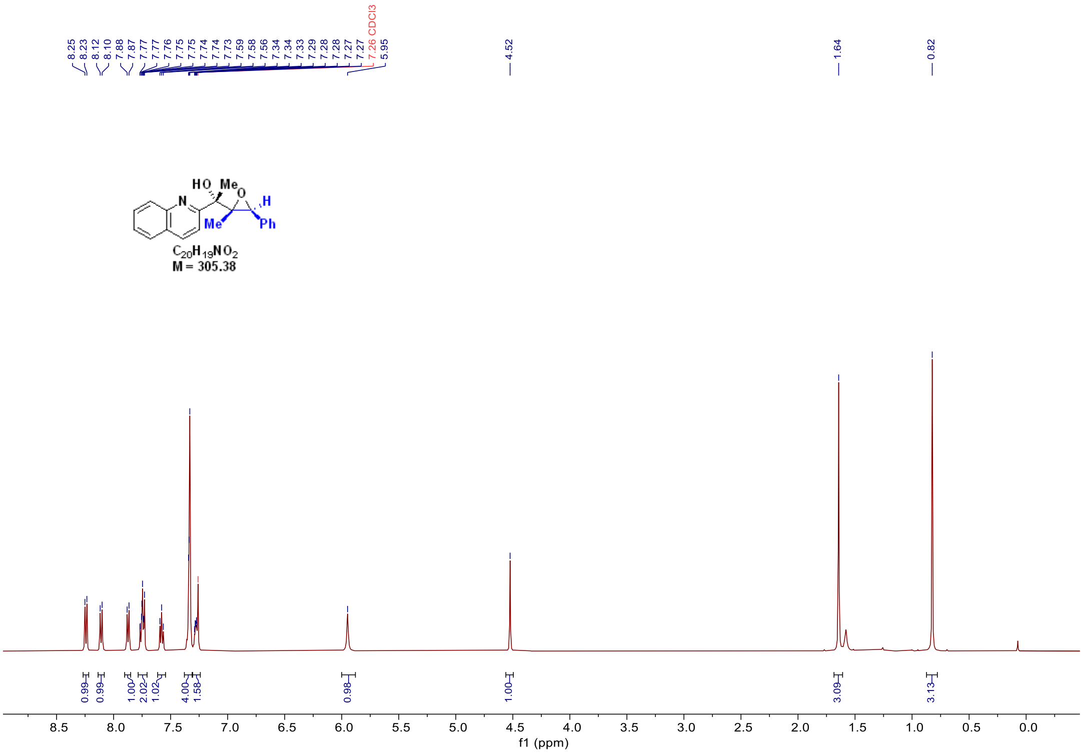

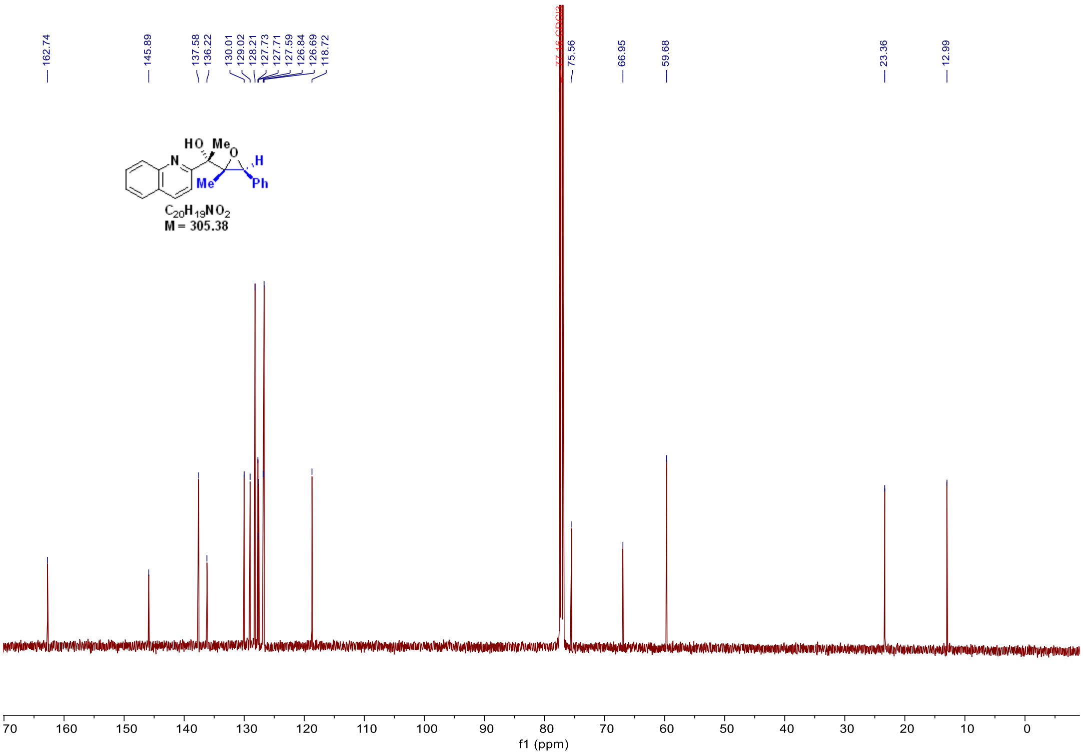

^{1}H NMR (500 MHz, CD_3Cl, 298 K) and ^{13}C NMR (125 MHz, CD_3Cl, 298 K) of **7a**.

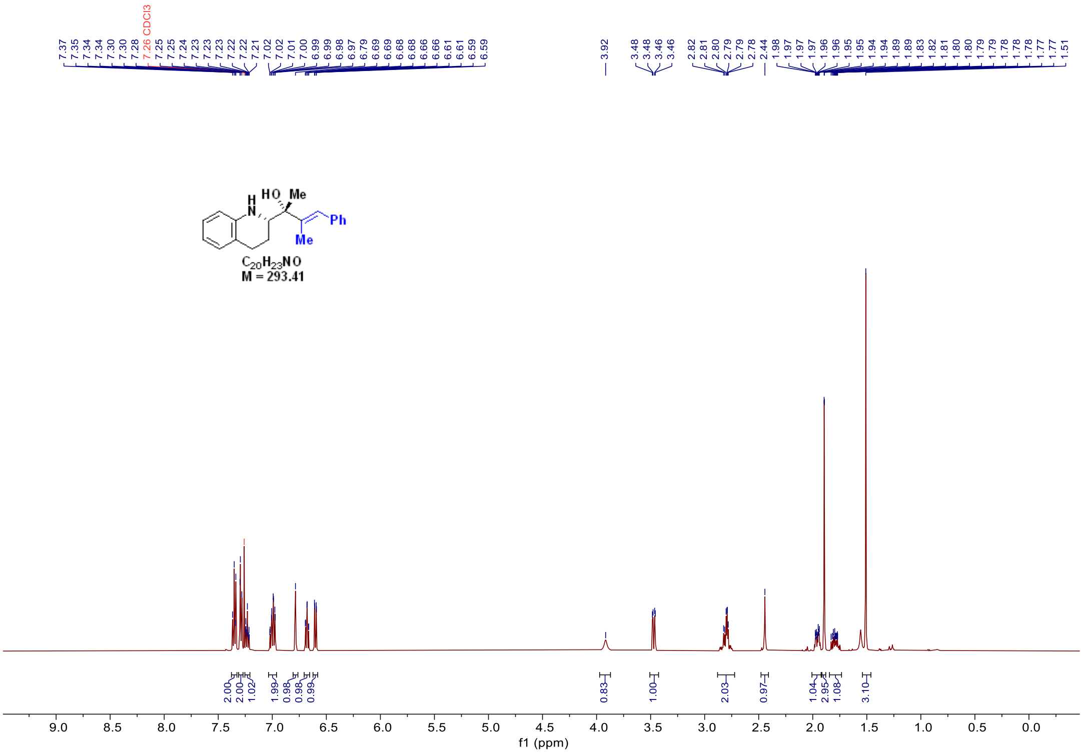

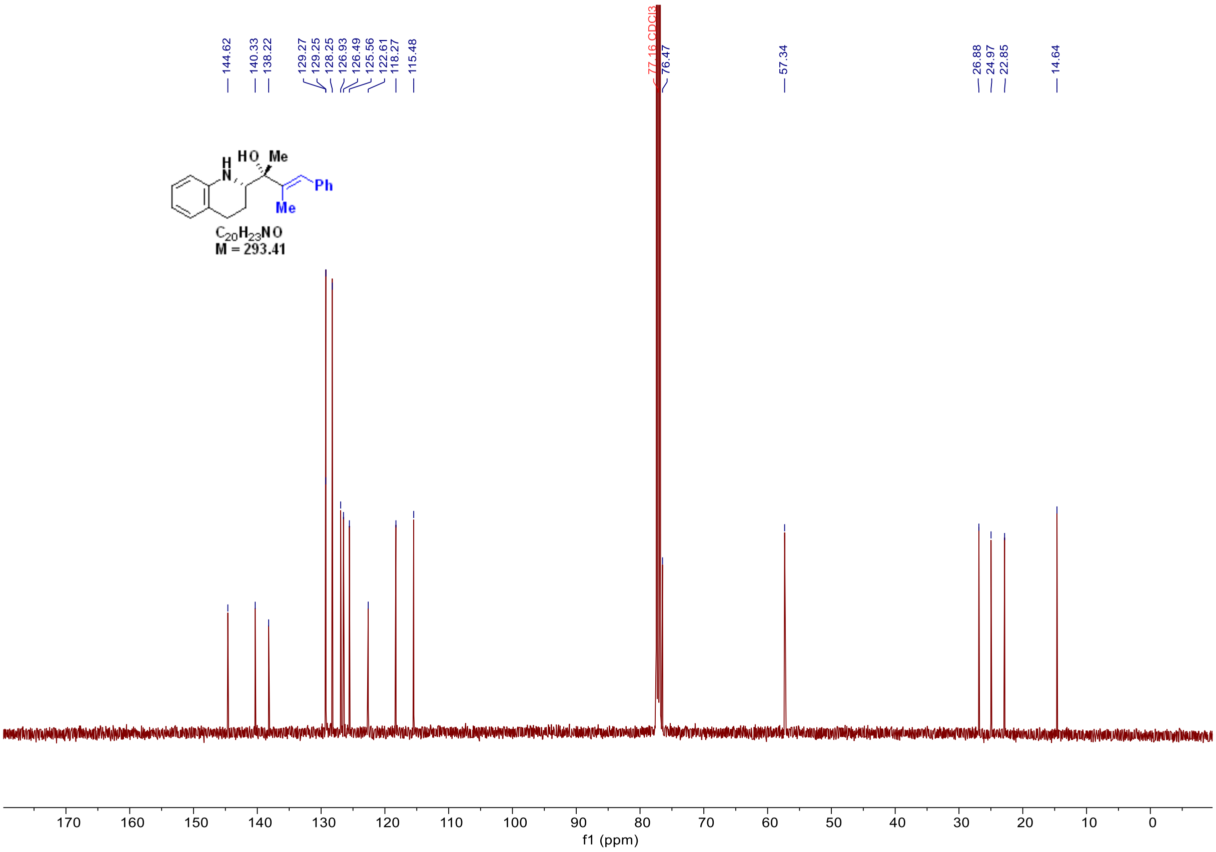

^{1}H NMR (500 MHz, CD_3Cl, 298 K) and ^{13}C NMR (125 MHz, CD_3Cl, 298 K) of **(3*R*,3a*S*)-3-methyl-3-((*E*)-1-phenylprop-1-en-2-yl)-3,3a,4,5-tetrahydro-1*H*-oxazolo[3,4-*a*]-quinolin-1-one**.

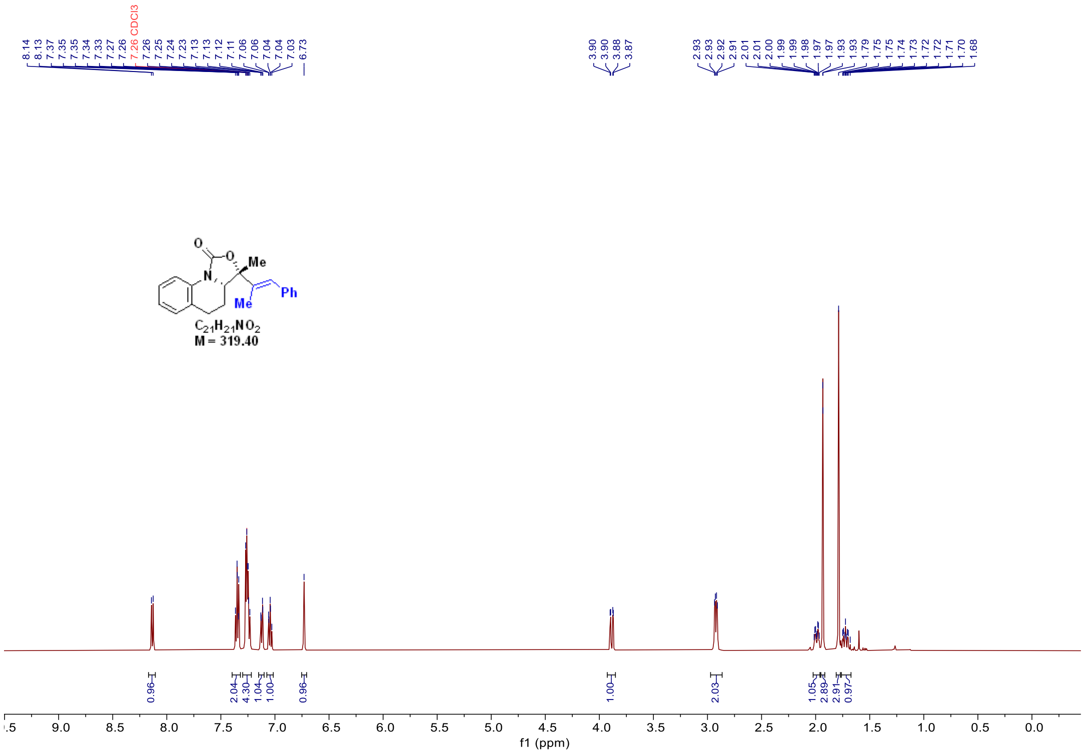

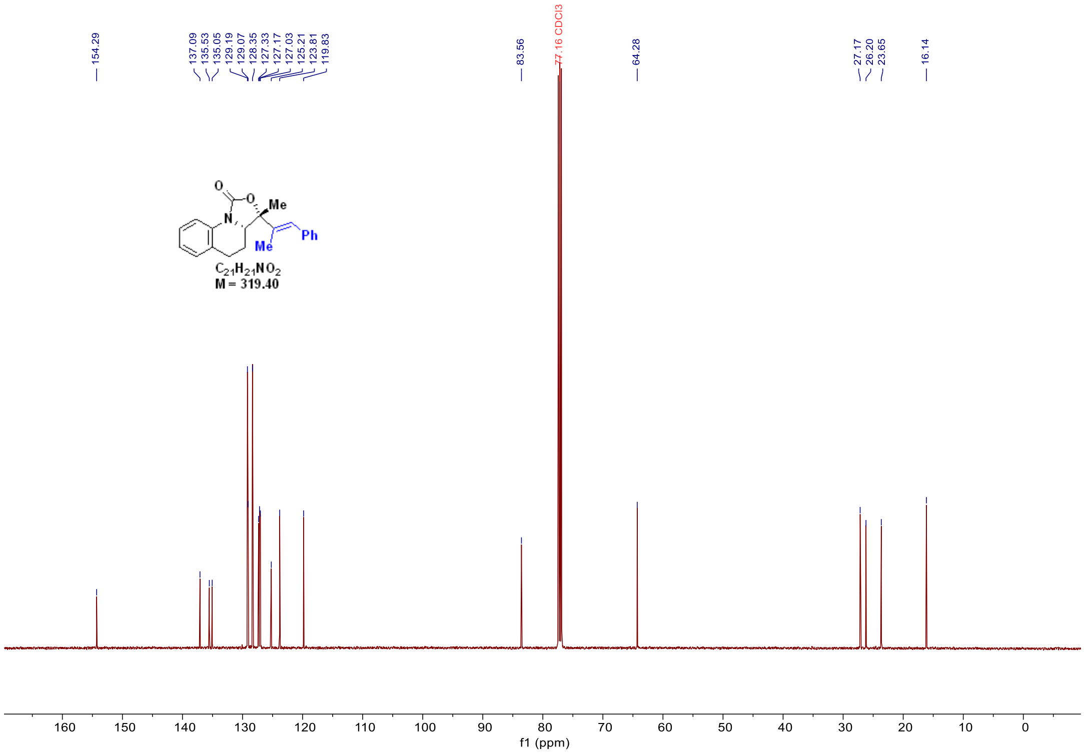

^{1}H NMR (500 MHz, CD_3Cl, 298 K) and ^{13}C NMR (125 MHz, CD_3Cl, 298 K) of **7b**.

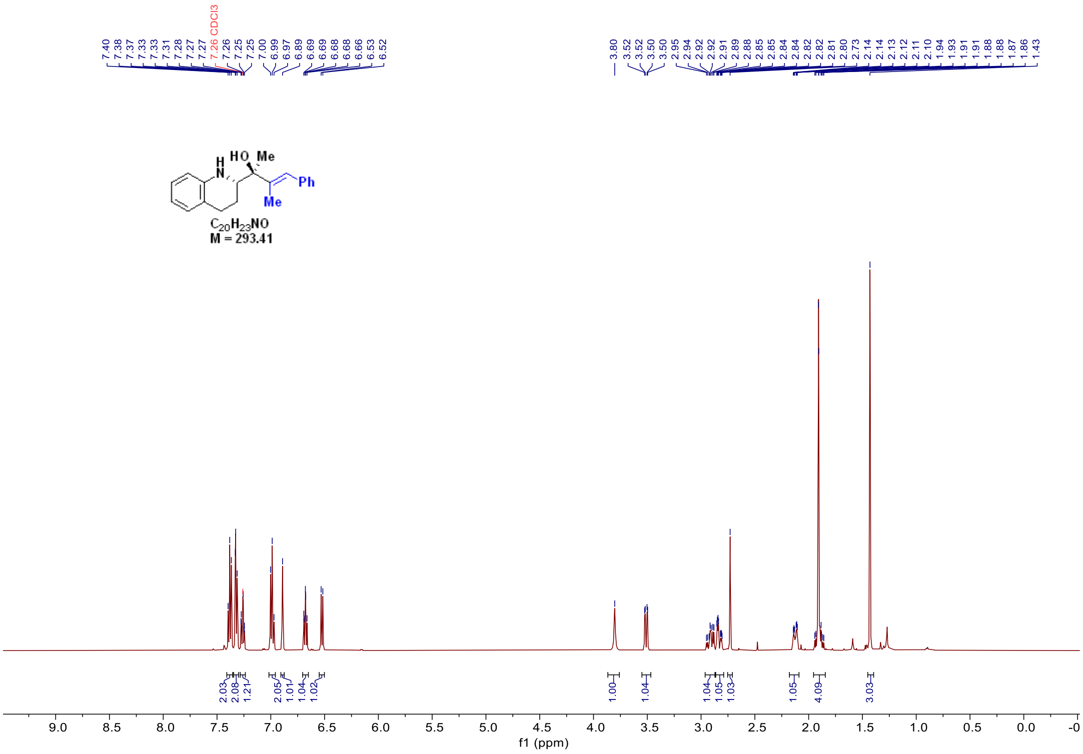

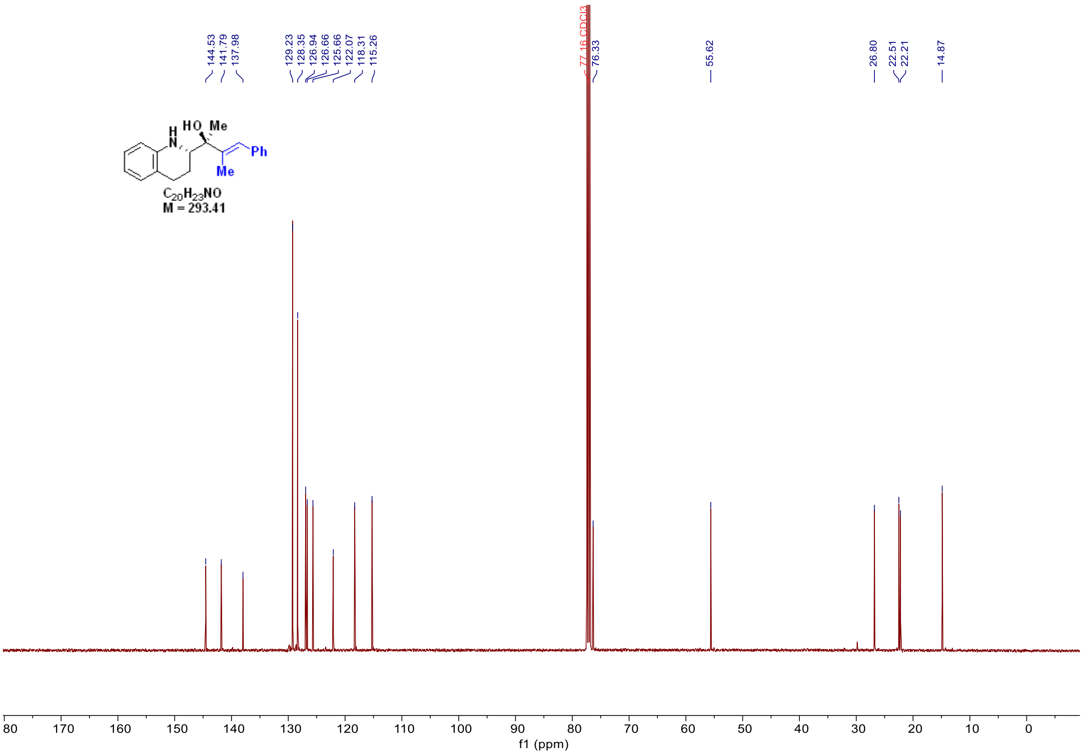

^{1}H NMR (500 MHz, CD_3Cl, 298 K) and ^{13}C NMR (125 MHz, CD_3Cl, 298 K) of ***trans*-3-methyl-3-((*E*)-1-phenylprop-1-en-2-yl)-3,3a,4,5-tetrahydro-1*H*-oxazolo[3,4-*a*]quinolin-1-one**.

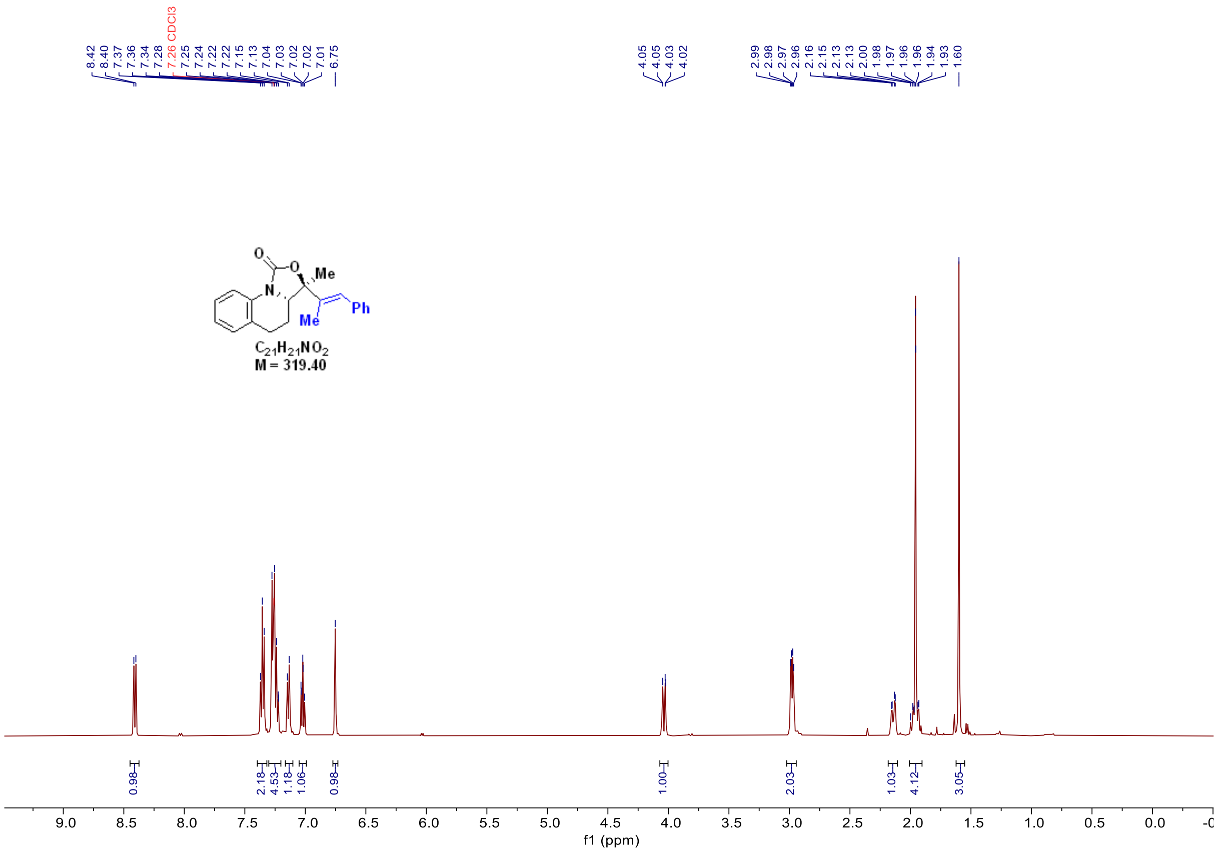

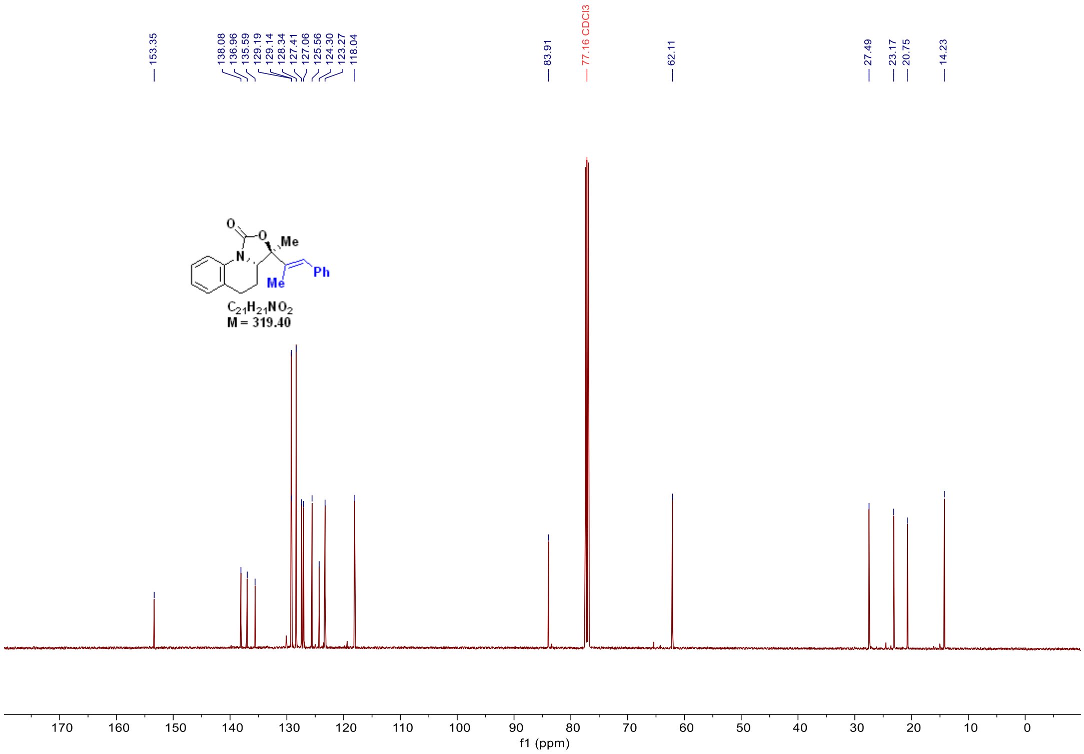

^{1}H NMR (500 MHz, CD_3Cl, 298 K) and ^{13}C NMR (125 MHz, CD_3Cl, 298 K) of **2-ethylnaphthalene**.

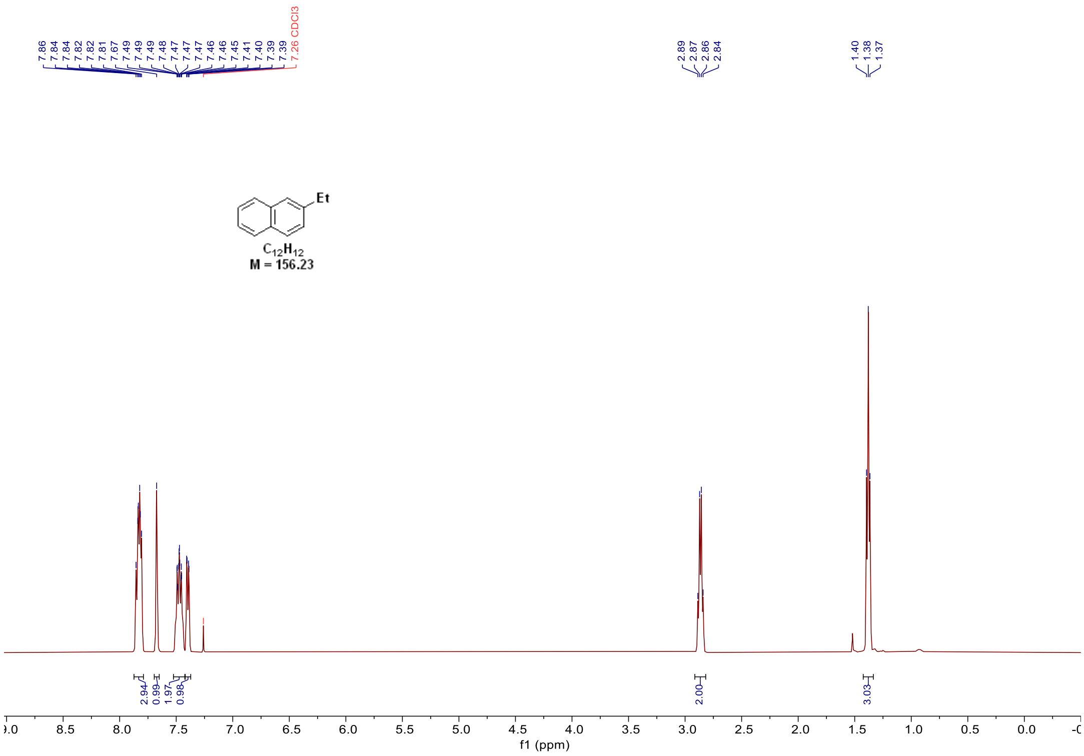

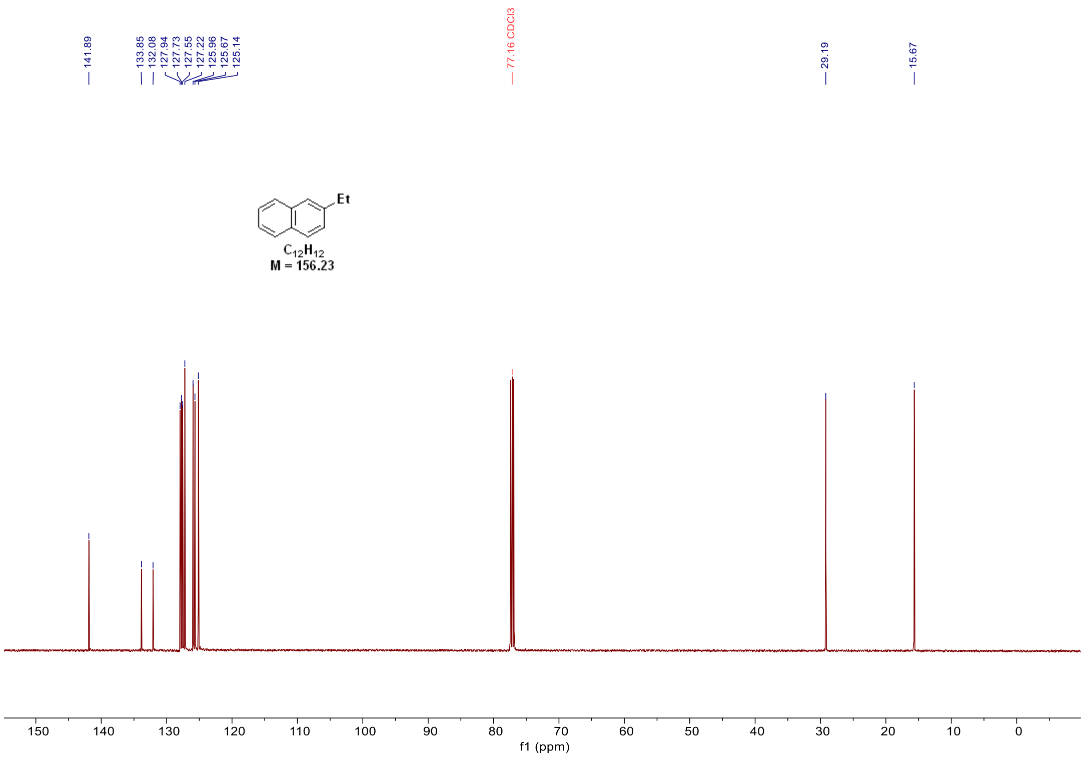

^{1}H NMR (500 MHz, CD_3Cl, 298 K) and ^{13}C NMR (125 MHz, CD_3Cl, 298 K) of **3ba**.

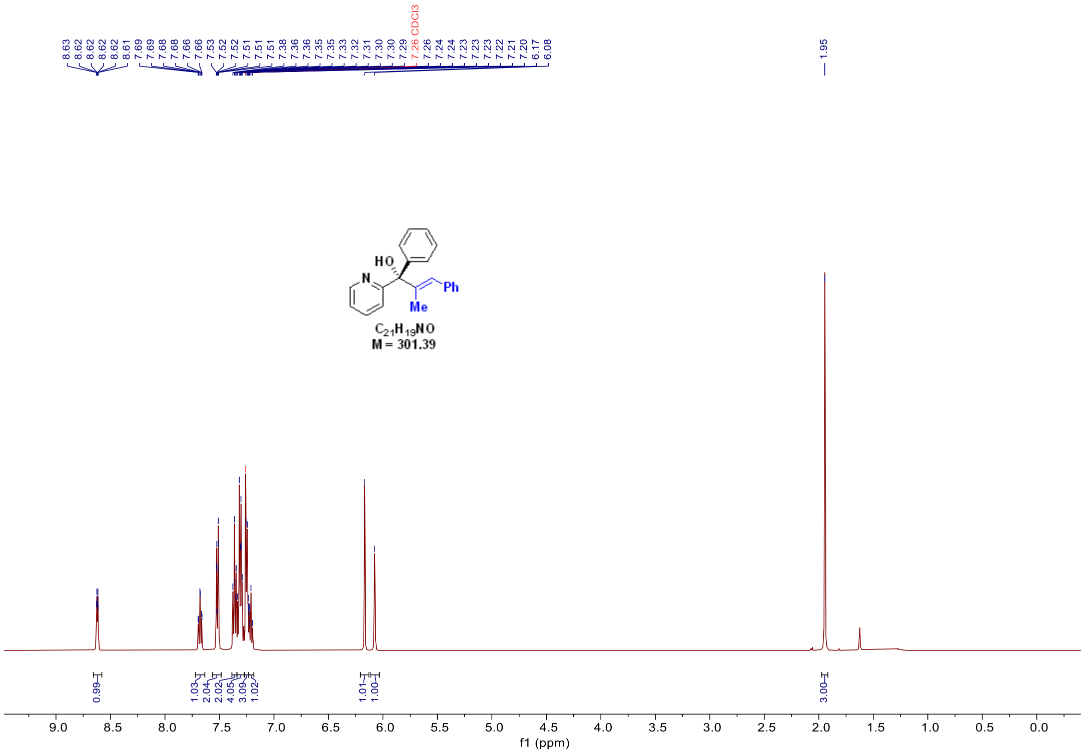

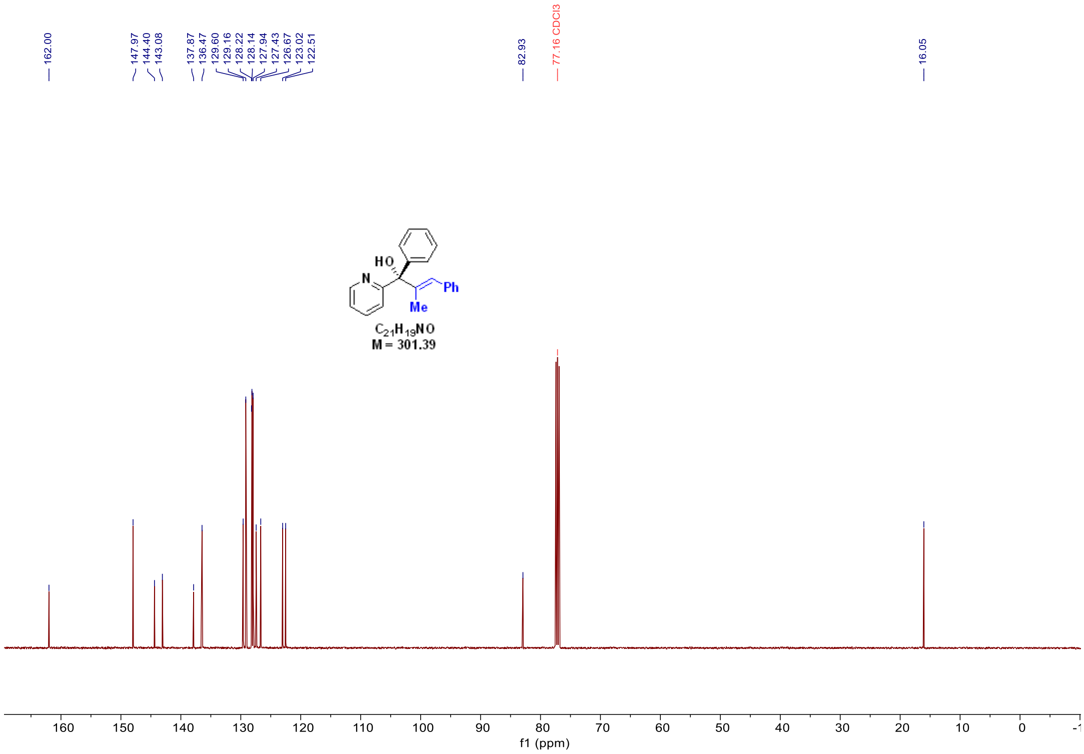

^{1}H NMR (500 MHz, CD_3Cl, 298 K) and ^{13}C NMR (125 MHz, CD_3Cl, 298 K) of *iso*-**3ba**.

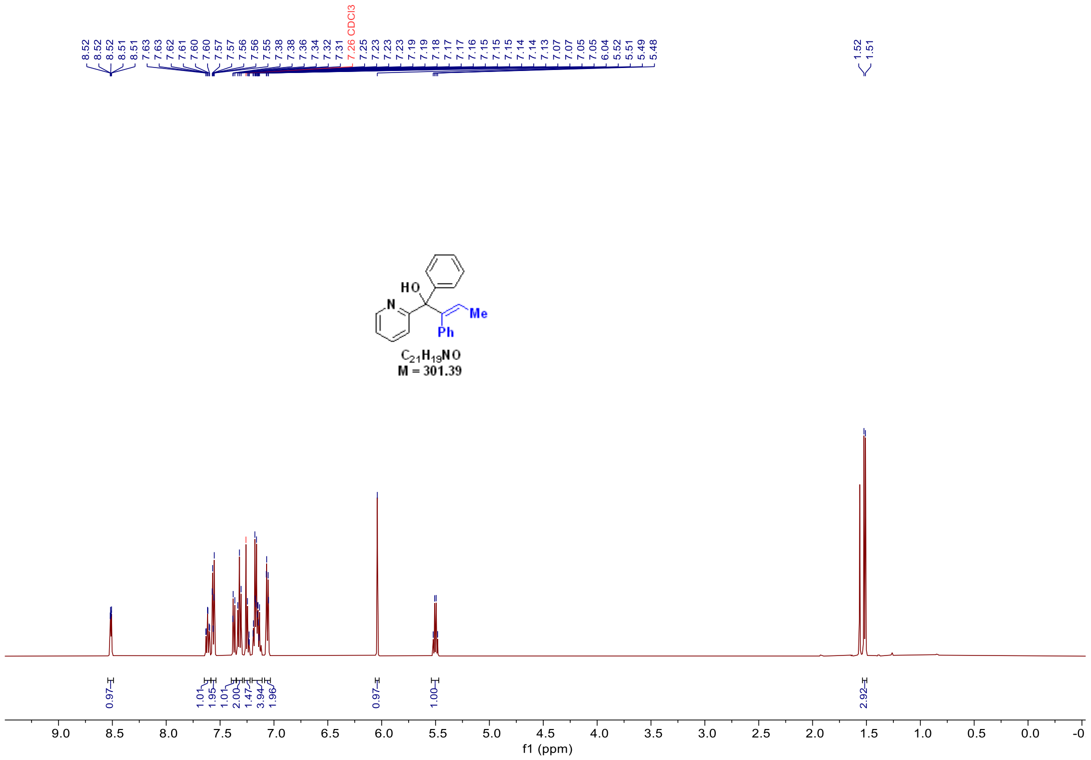

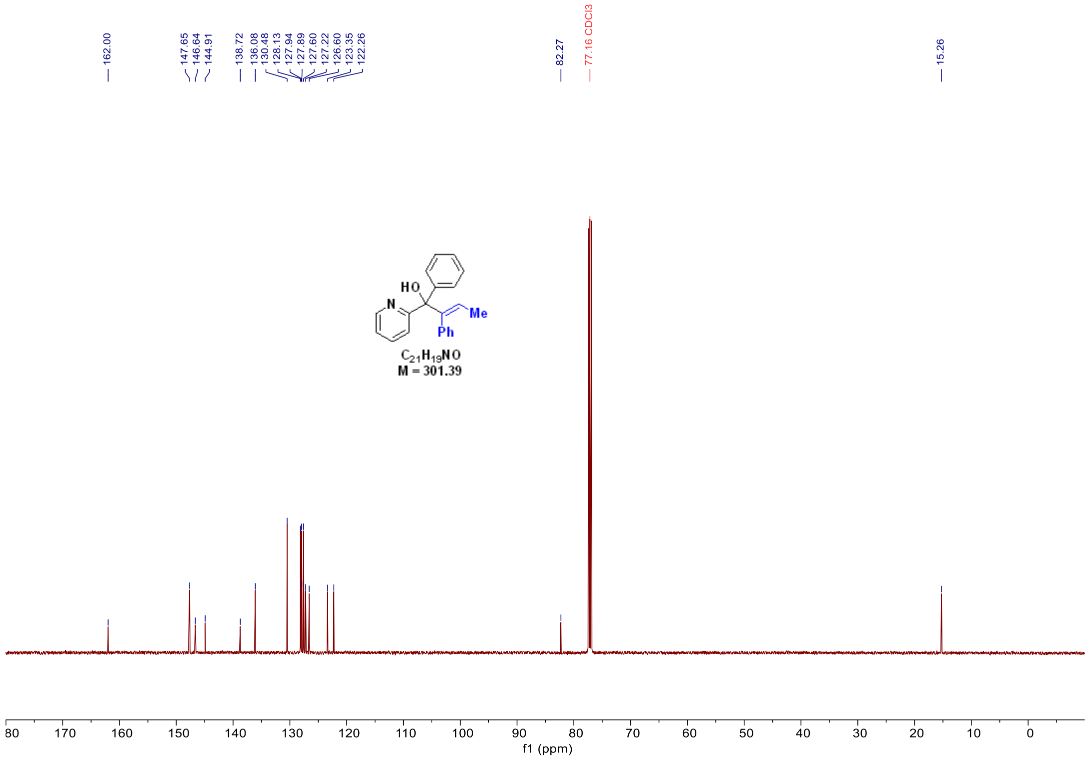

9 References

[1] Cao, Y.; Zhang, S.; Antilla, J. C. *ACS Catal*. **2020**, *10*, 10914–10919.

[2] (a) Gómez, I.; Alonso, E.; Ramón, D. J.; Yus, M. *Tetrahedron* **2000**, *56*, 4043–4052. (b) Fakhfakh, M. A.; Fournet, A.; Prina, E.; Mouscadet, J.-F.; Franck, X.; Hocquemillera, R.; Figadère, B. *Bioorg. Med. Chem*. **2003**, *11*, 5013–5023. (c) Chen, F.; He, D.; Chen, L.; Chang, X.; Wang, D. Z.; Xu, C.; Xing, X. *ACS Catal*. **2019**, *9*, 5562–5566. (d) Nian, S.; Ling, F Chen, J.; Wang, Z.; Shen, H.; Yi, X.; Yang, Y.-F.; She, Y.; Zhong, W. *Org. Lett*. **2019**, *21*, 5392–5396. (e) Yu, R.; Hao, F.; Zhang, X.; Fang, Z.; Jin, Z.; Liu, G.; Dai, G.; Wu, J. *J. Org. Chem*. **2023**, *88*, 8279–8285.

[3] Roy, S.; Das, S. K.; Chattopadhyay, B. *Angew. Chem. Int. Ed*. **2018**, *57*, 2238–2243.

[4] (a) Brown, E. V.; Shambhu, M. B. *J. Org. Chem*. **1971**, *36*, 2002–2004. (b) Wang, X.; Wang, D. Z. *Tetrahedron* **2011**, *67*, 3406–3411. (c) Yang, H.; Huo, N.; Yang, P.; Pei, H.; Lv, H.; Zhang, X. *Org. Lett*. **2015**, *17*, 4144–4147. (d) Wang, Z.; Zhao, X.; Wang, S.; Huang, A.; Wang, Y.; He, J.; Ling, F.; Zhong, W. *Org. Biomol. Chem*. **2021**, *19*, 9746–9751. (e) Xu, X.; You, Y.; Jin, M. Y.; Meng, F.-J.; Xu, C.; Xing, X. *Sci. China Chem*. **2023**, *66*, 1443–1449.

[5] Zhong, S.; Deng, G.-J.; Dai, Z.; Huang, H. *Org. Chem. Front*. **2021**, *8*, 4419–4425.

[6] Guanti, G.; Riva, R. *Tetrahedron*: *Asymmetry* **2001**, *12*, 1185–1200.

[7] (a) Lu, B.; Li, C.; Zhang, L. *J. Am. Chem. Soc*. **2010**, *132*, 14070–14072. (b) Cruz, F. A.; Dong, V. M. *J. Am. Chem. Soc*. **2017**, *139*, 1029–1032.

[8] (a) Tomita, R.; Koike, T.; Akita, M. *Angew. Chem. Int. Ed*. **2015**, *54*, 12923–12927. (b) Zhu, Z.-F.; Tu, J.-L.; Liu, F. *Chem. Commun*. **2019**, *55*, 11478–11481. (c) Davison, R. T.; Parker, P. D.; Hou, X.; Chung, C. P.; Augustine, S. A.; Dong, V. M. *Angew. Chem. Int. Ed*. **2021**, *60*, 4599–4603. (d) Wech, F.; Gellrich, U. *ACS Catal*. **2022**, *12*, 5388–5396. (e) Rayaroth, A.; Elikkottil, A.; Jayakumari, C. M.; Vennoli, K. A.; Vennapusa S. R.; Kaliyamoorthy, A. *Chem. Commun*. **2022**, *58*, 10671–10674.

[9] (a) Hötling, S.; Haberlag, B.; Tamm, M.; Collatz, J.; Mack, P.; Steidle, J. L. M.; Vences, M.; Schulz, S. *Chem. Eur. J.* **2014**, *20*, 3183–3191. (b) Connolly, T.; Wang, Z.; Walker, M. A.; McDonald, I. M.; Peese K. M. *Org. Lett*. **2014**, *16*, 4444–4447. (c) Börjesson, M. ; Moragas, T. ; Martin, R. *J. Am. Chem. Soc.* **2016**, *138*, 7504–7507.

[10] Crisenza, G. E. M.; McCreanor, N. G.; Bower, J. F. *J. Am. Chem. Soc*. **2014**, *136*, 10258–10261.

[11] Zhang, C.; Gao, A. Z.; Nie, X.; Ye, C.-X.; Ivlev, S. I.; Chen, S.; Meggers, E. *J*. *Am*. *Chem*. *Soc*. **2021**, *143*, 13393–13400.

[12] Guan, J.; Chen, J.; Luo, Y.; Guo, L.; Zhang, W. *Angew*. *Chem*. *Int*. *Ed*. **2023**, *62*, e202306380.

[13] (a) Tang, W.; Sun, Y.; Xu, L.; Wang, T.; Fan, Q.; Lamc, K.-H.; Chanc, A. S. C. *Org*. *Biomol*. *Chem*. **2010**, *8*, 3464–3471. (b) Cao, J.; Su, Y.-X.; Zhang, X.-Y.; Zhu, S.-F. *Angew*. *Chem*. *Int. Ed.* **2023**, *62*, e202212976.

[14] Bew, S. P.; Hiatt-Gipson, G. D.; Lovell, J. A.; Poullain, C. *Org*. *Lett*. **2012**, *14*, 456–459.

[15] Wang, F.-F.; Luo, C.-P.; Wang, Y.; Deng, G.; Yang, L. *Org. Biomol. Chem.* **2012**, *10*, 8605–8608.

[16] Nielsen, C. D. T.; Burés, J. *Chem. Sci.* **2019**, *10*, 348–353.
